# Device-measured physical activity, sedentary behaviour and cardiometabolic health and fitness across occupational groups: a systematic review and meta-analysis

**DOI:** 10.1186/s12966-019-0790-9

**Published:** 2019-04-02

**Authors:** Stephanie A. Prince, Cara G. Elliott, Kyle Scott, Sarah Visintini, Jennifer L. Reed

**Affiliations:** 1 Division of Cardiac Prevention and Rehabilitation, University of Ottawa Heart Institute, 40 Ruskin Street, Ottawa, Ontario K1Y 4W7 Canada; 2 Centre for Surveillance and Applied Research, Public Health Agency of Canada, Ottawa, Canada; 3 School of Human Kinetics, Faculty of Health Sciences, University of Ottawa, Ottawa, Canada; 4 Berkman Library, University of Ottawa Heart Institute, Ottawa, Canada; 5 School of Epidemiology and Public Health, Faculty of Medicine, University of Ottawa, Ottawa, Canada

**Keywords:** Occupation, Workplace, Motor activity, Sedentary behaviour, Systematic review

## Abstract

**Background:**

With approximately 8 hours of one’s waking day spent at work, occupational tasks and environments are important influencers on an individual’s physical activity (PA) and sedentary behaviours. Little research has compared device-measured physical activity, sedentary behaviour and cardiometabolic outcomes between occupational groups.

**Objective:**

To compare device-measured movement (sedentary time [ST], light intensity physical activity [LPA], moderate-to-vigorous intensity physical activity [MVPA], and steps) across occupations. The secondary objective was to examine whether cardiometabolic and fitness outcomes differed by occupation.

**Methods:**

Five bibliographic databases were searched to identify all studies which included working age, employed adults from high-income countries, and reported on device-measured movement within occupations. Risk of bias within and across studies was assessed. Results were synthesized using meta-analyses and narrative syntheses.

**Results:**

The review includes 132 unique studies with data from 15,619 participants. Working adults spent ~ 60% of their working and waking time engaged in sedentary behaviour; a very small proportion (~ 4%) of the day included MVPA. On average, workers accumulated 8124 steps/day. Office and call center workers’ steps/day were among the lowest, while those of postal delivery workers were highest. Office workers had the greatest ST and the lowest time in LPA both at work and during wakeful time. However, office workers had the greatest minutes sent in MVPA during wakeful hours. Laborers had the lowest ST and spent a significantly greater proportion of their work time in LPA and MVPA. Healthcare and protective services workers had higher levels of LPA at work compared to other occupations. Workers in driving-based occupations tended to have a higher body mass index and blood pressure.

**Conclusion:**

This review identifies that occupational and wakeful time PA and ST differed between occupations. Future studies are needed to assess whether patterns differ by age and sex, describe leisure-time movement and movement patterns, and the relationship with cardiometabolic health.

**Systematic review registration:**

PROSPERO CRD42017070448.

**Electronic supplementary material:**

The online version of this article (10.1186/s12966-019-0790-9) contains supplementary material, which is available to authorized users.

## Introduction

It is well established that regular physical activity (PA) prevents several chronic conditions (e.g. cardiovascular disease, diabetes, cancer, hypertension, obesity, depression, and osteoporosis) and premature all-cause mortality [1, 2]. The World Health Organization recommends that adults accumulate a minimum of 150 min of moderate-to-vigorous intensity PA (MVPA) per week for good health [3]. Reported adherence to these guidelines in high-income countries remains low (36.8%) [4] and the prevalence of inactivity is more than double that in low income countries [5]. Independent of MVPA, there is strong evidence that time spent in sedentary activities (i.e. sitting, reclining, lying down while awake) [6] increases one’s risk for cardiometabolic diseases, cancers, depression, and premature death [7–10]. Much of the evidence looking at the relationships between PA, sedentary behaviour and health outcomes has focused on behaviours that occur outside of work hours (e.g. recreational PA, TV or leisure-time screen use) [10, 11].

In the landmark London Transport Workers Study, Morris et al. found that men who worked more physically active jobs (i.e. conductors) had a lower incidence of heart disease compared to those with inactive jobs (i.e. drivers) [12]. Although this study was published more than 50 years ago, the relationship between movement behaviours and health outcomes across occupations remains poorly understood. The “physical activity paradox” suggests that PA undertaken at work differs in quality, quantity and health effects than PA performed during leisure-time [13]. For example, occupational PA carried out at lower intensities may not elicit substantial improvements in cardiorespiratory fitness, such as in the case of nurses who have been shown to spend a large proportion of their wakeful time in light intensity PA (LPA) [14]. Recent systematic review evidence also suggests that higher levels of occupational PA (even while controlling for leisure-time MVPA) are associated with an increased risk in premature mortality in men [15]. It is important to build on past research to understand the intensities of PA at which workers engage both within and outside of working hours and the associations with cardiometabolic health.

With approximately 8 hours of one’s waking day spent at work [16], occupational tasks and environments can heavily influence PA and sedentary time (ST). Occupational PA and ST are broadly defined as the amount of these behaviours accumulated while at work (typically within an 8-h timeframe), whereas leisure-time PA or ST occurs outside of work hours [17]. In the only known systematic review examining occupation and leisure-time PA, Kirk et al. found that white-collar workers accumulated higher amounts of leisure-time PA than blue-collar workers, and occupations with low occupational PA and long work hours were associated with lower leisure-time PA [18]. However, research has shown that individuals are more sedentary at work than during leisure-time, irrespective of occupation type [19]. Beyond white- vs. blue-collar classifications, little research has compared PA levels (i.e. ST, LPA, MVPA) and cardiometabolic and fitness outcomes between occupational groups. Identifying the specific occupations that enable higher rates of inactivity can inform workplace and lifestyle interventions aimed at improving workers’ PA levels and health status.

Most of the research examining occupational PA has been measured using self-report tools (e.g. questionnaires) [20], introducing considerable measurement errors (i.e. social desirability, recall bias, poor validity) [21, 22]. It is likely that individuals misperceive some of their occupational PA as MVPA when in fact, it is spent at lower intensities [23]. Activity monitors such as accelerometers, inclinometers, and pedometers have been validated for measuring ST (including posture in the case of inclinometers), steps, and PA of varying intensity, bouts, and duration [24, 25]. These devices allow movement to be continuously and more precisely monitored, thus effectively studied in many occupational environments.

No rigorous systematic investigations have examined device-measured PA across occupations. With higher income countries experiencing a transition towards more sedentary occupations compared to lower income countries, it is important to understand the PA levels experienced by these workers. Therefore, the primary objective of this systematic review was, to summarize and compare device-measured PA across occupations in adults from high-income countries. This includes step counts, ST, and, time spent in LPA, moderate intensity PA (MPA), vigorous intensity PA (VPA) and MVPA. The secondary objective was to examine whether cardiometabolic and fitness outcomes differ by occupation.

## Methods

The review was prospectively registered with PROSPERO (#CRD42017070448).

### Study inclusion criteria

#### Population

Working age, employed adults (mean age = 18–65 years) from high-income countries [26].

#### Exposure

PA and ST were examined across occupation groups. Eligible occupations included in the meta-analyses were either specific occupations described within individual studies (e.g. nurses, office workers, postal workers) or occupational groups, which were identified a priori by the authors based on similar occupational task characteristics. Occupational groups included: (1) drivers (bus, lorry, taxi, transit, truck); (2) laborers (agriculture, cleaners, construction workers, dry cleaners, farmers, freight mechanics); (3) protective services (army, firefighters, national guard, paramedics, police); (4) factory workers (extractive and precision, fabric machine operators, meat processing); (5) call center workers; (6) academics (researchers, scientists, lecturers); (7) school teachers; (8) healthcare (diagnosticians, nurses, nurses’ aides, physicians, physiotherapists); (9) office workers (administrative, civil servants, postal workers in offices, professionals/managers, records, secretaries, software); (10) customer service and hospitality (cooks, customer service, retail, servers); and, (11) postal delivery workers.

#### Outcomes

The primary outcome was device-measured (e.g. accelerometers, inclinometers, pedometers) physical behaviours within occupation groups, including: ST; LPA; MPA; VPA; and, MVPA. This included activity at work, leisure-time and during wakeful time. The secondary outcomes were cardiometabolic health indicators (self-report and objective measures), including: body mass index (BMI); waist circumference; waist-to-hip ratio (WHR); body fat percentage; resting systolic blood pressure (SBP) and diastolic blood pressure (DBP); total cholesterol; high density lipoprotein (HDL), low density lipoprotein (LDL); triglycerides; total cholesterol-to-HDL ratio (TC:HDL); blood glucose; HbA1c; HOMA-IR; and, fitness (V̇O_2_ peak). All units for cardiometabolic outcomes were standardized to the same units.

#### Study designs

Observational (e.g. cross-sectional, prospective cohort, retrospective cohort) and experimental (e.g. baseline data from randomized controlled trials or quasi-experimental trials) studies were included.

#### Publication status and language

Both published (peer-reviewed) and unpublished grey literature (e.g. abstracts) were eligible. No language restrictions were imposed on the search, but only papers published in English or French were included due to translation limitations.

### Search strategy

The search strategy was created by a medical librarian (SV) in discussion with the team (SAP, JLR, CGE). The search was first created in MEDLINE using a combination of index terms and keywords related to the workplace, PA, and device measurement tools. The search strategy was then peer-reviewed by a second medical librarian (NA). Once the search was finalized (see Additional file 1: Table S1 for MEDLINE search strategy), it was translated to the other bibliographic databases.

Searches were initially conducted from database inception to July 31, 2017 and then updated to include papers indexed up to and including December 12, 2018 in MEDLINE (Ovid, MEDLINE(R) Epub Ahead of Print, In-Process & Other Non-Indexed Citations, MEDLINE(R) Daily and MEDLINE(R)), EMBASE (Ovid, Embase Classic + Embase), CINAHL (EBSCO), SPORTDiscus (EBSCO), and Dissertations & Theses Global (Proquest). Search results were exported to EndNote X7 (Thompson Reuters, San Francisco, CA, USA) and duplicates removed through manual inspection using the EndNote duplicate identification function.

### Selection of studies

Following the initial search, titles and abstracts were exported from EndNote X7 into Microsoft Excel for screening. During the updated search the titles and abstracts were exported into Covidence software (Veritas Health Innovation, Melbourne, Australia). Two reviewers independently reviewed all titles and abstracts (SAP, JLR, CGE) and full texts (SAP, JLR, CGE, KS). A third reviewer was consulted if disagreements occurred. Reviewers were not blinded to the authors of the studies.

### Data extraction and analysis

Data extraction was completed by one reviewer and verified by a second. Information extracted included: publication details; participant characteristics; occupation; sample size analyzed; study design; PA intensity and cardiometabolic and fitness indicators examined; device; units of measurement; and, quantity of the movement intensity (e.g. minutes/day, proportion of wear time) and cardiometabolic indicators across occupational groups.

A narrative synthesis, including summary tables, was used to examine PA and sedentary behaviors and cardiometabolic outcomes across all studies and assess overall trends. Outcomes with sufficient number of studies per outcome per occupation group (≥3 studies/group) were compared using random effects meta-analyses to provide a summary measure of movement intensity (e.g. minutes/day of ST) overall and per each occupational sub-group with variance not assumed to be common among subgroups. Forest plots and meta-analyses were created using Comprehensive Meta Analysis Version 3 (Englewood, NJ, USA) to compare quantities of PA intensities, ST and cardiometabolic outcomes (in same units) and 95% confidence intervals (CIs). Sitting and ST were combined as a single outcome. Pairwise comparisons between groups were performed using a Microsoft Excel sheet supplied by Comprehensive Meta Analysis. Publication bias was conducted using Egger’s two-tailed test (when the number of studies was ≥10) whereby *p* < 0.10 indicated the possibility of bias [27].

### Risk of bias

We assessed the risk of bias of the individual studies using a modified version (observational studies) [28] of the Cochrane Collaboration’s Tool for Assessing Risk of Bias. Risk of bias graphs were created using Review Manager Version 5.3 (The Nordic Cochrane Centre, The Cochrane Collaboration, Copenhagen). Studies were assessed for potential biases including: selection bias (sampling methods); performance bias (measurement of exposure); attrition bias (incomplete follow-up + > 10% missing data), selective reporting bias (selective/incomplete reporting, rated high if secondary data analyses); and, other possible sources of bias (i.e. self-reported SB and not controlling for confounders). Risk of bias assessments were carried out by one assessor and verified by a second.

## Results

### Study characteristics

**Fig. 1:**
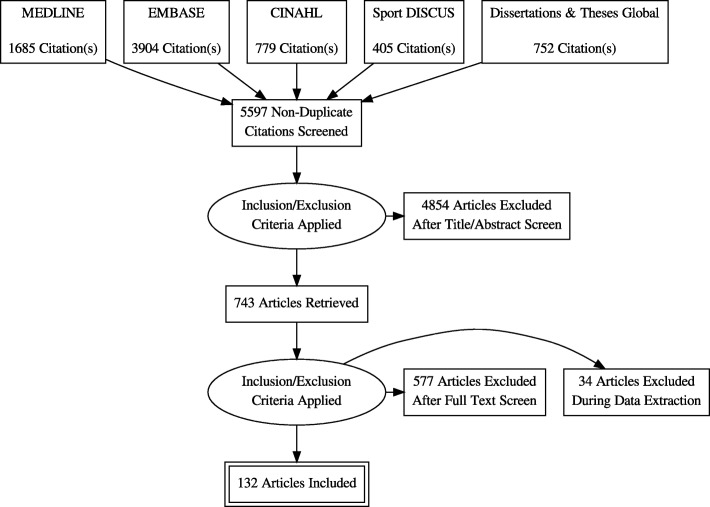
PRISMA flow diagram

Figure 1 provides a detailed flow diagram of the literature search and screening process. Of the 5597 originally identified non-duplicate publications, 132 unique studies [14, 29–160] met the eligibility criteria. The review includes data from 15,619 participants. Study characteristics are presented in Additional file 2: Table S2. The included studies were published from 1983 to 2018. The countries with the most studies included Australia (27%) and the Unites States (24%). Most (69%) were from smaller studies (*N* < 100 participants). The majority reported on office-based/desk-based occupations (referred henceforth as office workers). Older studies tended to use pedometers and early generation accelerometers, while more recent studies used newer accelerometers and inclinometers. Accelerometers were the most used devices (*N* = 70 studies) followed by inclinometers (*N* = 41 studies) and pedometers (*N* = 30 studies). Steps/day was the most reported behaviour outcome.

### Risk of bias

**Fig. 2:**
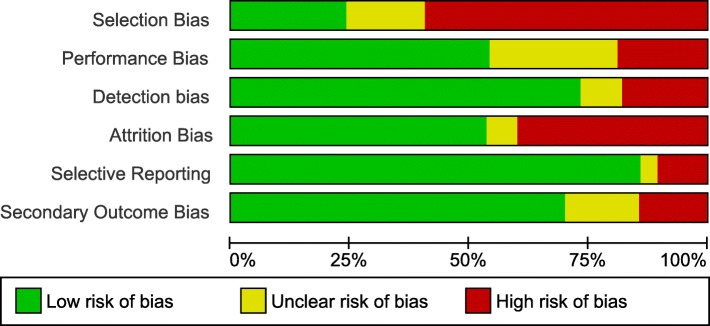
Summary of risk of bias scores

Risk of bias results are summarized in Fig. 2. More than half of the studies had a risk of selection bias largely due to convenience samples. Approximately 20% had no description of how the occupational group was defined (included no clear inclusion criteria; poor selection bias) and occupation definitions were unclear in an additional ~ 30% (performance bias). Most of the studies had a low risk of detection bias as most used validated cut-points to define movement intensity (if studies used an unsealed device they were rated as high). Just under 50% of the studies had a high or unclear risk of attrition bias; largely a result of having ≥10% missing data. Most studies had a low risk of selective reporting bias; most reported on all available data. Finally, most had a low risk of ‘other bias’ as they used objective measures of cardiometabolic outcomes.

### Physical behaviour outcomes

A summary of physical behaviour outcomes across occupational groups is shown in Additional file 3: Table S3. All meta-analyses and funnel plots for behaviour outcomes are available in Additional file 4: Figures S1-S6.

#### Sedentary time (ST)

**Fig. 3:**
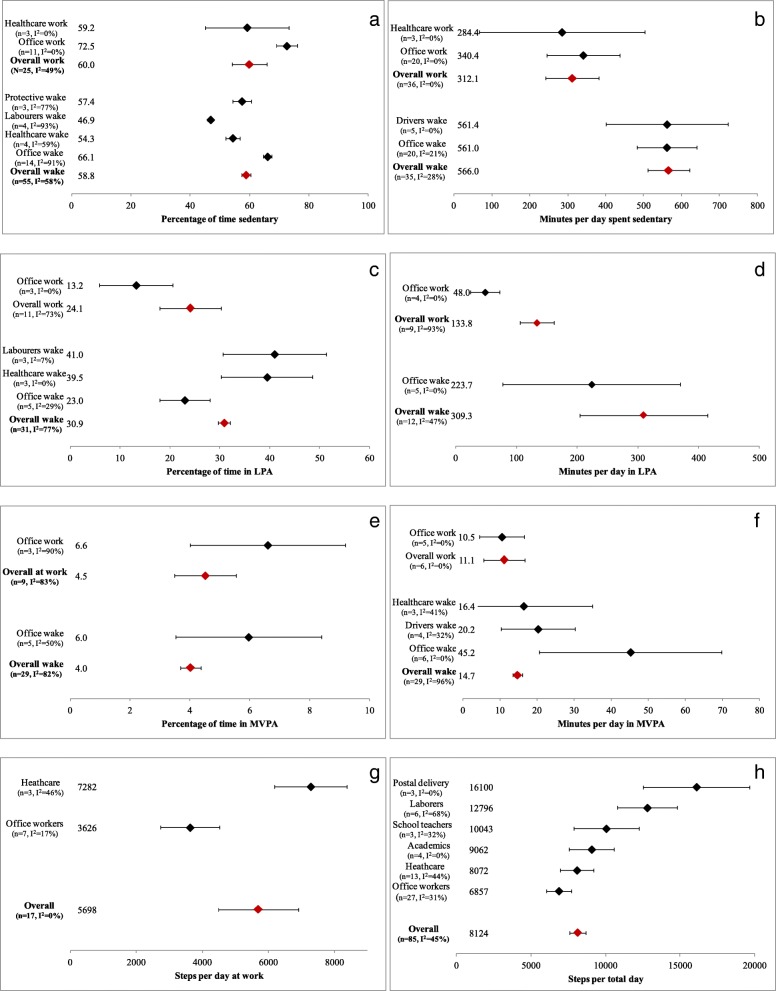
**a**-**h** Percentage and minutes of total wake time and time at work spent in sedentary (**a** and **b**), light- (**c** and **d**) and moderate-to-vigorous intensity physical activity (**e** and **f**) and steps at work (**g**) and during wakeful time (**h**) overall (red diamonds) and by occupation group (black diamonds). I^2^ - measure of heterogeneity, LPA - light intensity physical activity, MVPA – moderate-to-vigorous intensity physical activity, n - number of studies

A meta-analysis of the percentage of ST found that, on average, 60.0% (95% CI: 54.2–65.7%, I^2^ = 49%, Egger’s *p* = 0.0399) of time at work and 58.8% (95% CI: 56.7–59.8%, I^2^ = 58%, Egger’s *p* = 0.3240) of all workers’ wakeful time was spent being sedentary (Fig. 3a). The proportion of ST was significantly higher in office workers than all other occupations both at work (72.5% vs. 49.7%, Z = 3.9919, *p* = 0.0001) and during wakeful time (66.1% vs. 55.9%, Z = 6.9970, *p* = 0.0000) (Additional file 4: Figure S1c, e, j, l). Office workers appeared to spend a lower amount of their non-work time in ST; 58.5% (95% CI: 55.5–61.6%, *N* = 4) of their non-work days (including weekends) was spent sedentary (Additional file 4: Figure S1ff). Daily ST in office workers with access to sit-stand desks did not appear different from those without (qualitative assessment). Laborers’ proportion of ST was significantly lower than all other workers during wakeful time (46.9% vs. 59.6%, Z = 7.7113, *p* = 0.0000) (Additional file 4: Figure S1m-n) and appeared lower during work hours (qualitative assessment). Healthcare workers’ proportion of wakeful ST was significantly lower than all other workers (54.3% vs. 59.2%, Z = 3.2856, *p* = 0.001) (Additional file 4: Figure S1p-q).

On average, workers spent 312.1 min/day at work sedentary (95% CI: 242.2–381.9, I^2^ = 28%, Egger’s *p* = 0.00003) and 565.8 min/day sedentary (95% CI: 510.4–620.8, I^2^ = 0%, Egger’s *p* = 0.00037). No significant differences were found between occupational groups (Fig. 3b). In general, occupations that largely involve sitting (i.e. drivers, office workers, call center) engaged in greater amounts of ST at work than those in more active occupations (i.e. laborers, healthcare, protective services). There was evidence of publication bias across all measures of ST. Office workers were found to spend 593.1 min/day (95% CI: 541.2–645.0, *N* = 5) sedentary on their non-workdays/weekends (Additional file 4: Figure S1dd), which appeared similar to their ST on workdays (qualitative assessment).

#### Light intensity physical activity (LPA)

A meta-analysis of the percentage of time spent in LPA identified that, on average, workers spent 24.1% (95% CI: 17.9–30.2%, I^2^ = 73%, Egger’s *p* = 0.0005) of their work time and 30.9% (95% CI: 29.7–32.1%, I^2^ = 77%, Egger’s *p* = 0.9093) (Fig. 3c) of their wakeful time engaged in LPA. Office workers spent a lower proportion of their waking hours (23.0% vs. 32.3%, Z = 3.5769, *p* = 0.0003) and work time (13.2% vs. 28.3%, Z = 2.4714, *p* = 0.0135) in LPA when compared to all other workers (Additional file 4: Figure S2c, d, g, h). Laborers spent a greater proportion of their waking hours in LPA compared to all other workers (41.0% vs. 30.0%, Z = 2.0767, *p* = 0.0378) (Additional file 4: Figure S2k-l). Healthcare workers spent a significantly higher proportion of their wakeful hours engaged in LPA compared to all other workers (39.5% vs. 30.0%, Z = 2.0226, *p* = 0.0431) (Additional file 4: Figure S2i-j). Laborers, protective services, and healthcare workers appeared to spend a greater proportion of their work time in LPA compared to desk-based occupations.

On average, workers spent 133.8 (95% CI: 105.7–162.0, I^2^ = 73%, Egger’s *p* = 0.08696) and 309.3 (95% CI: 204.1–414.5, I^2^ = 47%, Egger’s *p* = 0.05699) minutes/day at work and during wakeful time in LPA, respectively (Fig. 3d). Office workers spent significantly fewer minutes at work engaged in LPA compared to all other workers (48.0 vs. 223.5, Z = 4.0153, *p* = 0.0001). Based on our qualitative assessment, it appears that laborers and healthcare workers tended to have higher minutes/day spent in LPA. Call center and office workers tended to have fewer minutes/day of LPA at work, while protective service and healthcare workers had greater minutes/day at work in LPA.

#### Moderate-to-vigorous intensity physical activity (MVPA)

A meta-analysis of the percentage of daily time spent in MVPA identified that, on average, workers spent 4.5% (95% CI: 3.5–5.6%, I^2^ = 83%, Egger’s *p* = 0.07633) of their work time and 4.0% (95% CI: 3.7–4.4%, I^2^ = 82%, Egger’s *p* = 0.08545) of their wakeful time in MVPA (Fig. 3e). No significant differences between-occupational groups were identified. However, with the removal of an outlier (office workers) [96], office and call center workers tended to spend a lower proportion of their work time in MVPA compared to other workers. A meta-analysis found that, on average, workers (largely office workers) obtained 11.1 (95% CI: 5.5–16.7) minutes at work and 14.7 min per wakeful time (95% CI: 13.5–16.0, I^2^ = 96%, Egger’s *p* = 0.00007) in MVPA (Fig. 3f). Office workers engaged in more minutes/day of MVPA compared to other workers (45.2 vs. 11.0, Z = 2.7224, *p* = 0.0065) (Additional file 4: Figure S3g, i).

A subset of studies reported on MPA and VPA separately. Meta-analytic results showed that workers obtained an average of 43.2 (95% CI: 31.9–545.5, I^2^ = 99%) minutes/day and 4.4% (95% CI: 3.4–5.3%, I^2^ = 86%) at work and 52.6 (95% CI: 43.4–61.7, I^2^ = 94%) minutes/day and 5.5% (95% CI: 3.6–7.3%, I^2^ = 91%) during wakeful time of MPA (Additional file 4: Figure S4a-h). Office workers engaged in significantly fewer minutes of MPA during wakeful time compared to all other (included laborers, call center workers, healthcare, and athletes) workers (32.2 vs. 82.1, Z = 3.9246, *p* = 0.0001) (Additional file 4: Figure S4i-j). Our qualitative analyses indicate that laborers and protective service workers tended to have greater wakeful and work-specific time spent in MPA. Workers also engaged in 0.7 (95% CI: 0.3–1.098) minutes/day and 0.4% (95% CI: 0.2–0.6%) while at work and 1.9 (95% CI: 0.7–3.1) minutes/day and 0.2% (95% CI: 0.1–0.3%) during wakeful time in VPA (Additional file 4: Figure S5a-h).

#### Steps

Occupational groups that were primarily desk-based accumulated fewer steps than those with more ‘active’ occupations both during work time and across the day. The meta-analysis of steps taken during work time found that, on average, workers took 5698 steps at work (95% CI: 4485–6911, I^2^ = 0%) (Fig. 3g). Office workers accumulated significantly fewer steps at work compared to healthcare workers (3626 vs. 7282, Z = 5.0676, *p* = 0.0000) and all other workers (3626 vs. 7124, Z = 5.0735, *p* = 0.0000) (Additional file 4: Figure S6b-d). Laborers appeared to have some of the highest number of steps at work.

A meta-analysis of steps/wakeful hours identified that, on day per day (95% CI: 7586–8661, I^2^ = 45%) (Fig. 3h). Steps/day were lowest amongst office workers (6857, 95% CI: 6023–7692) and highest among postal delivery workers (16,100, 95%CI: 12526–19,674). Office workers had significantly fewer total steps during wakeful time than all other workers (6857 vs. 8745, Z = 3.2677, *p* = 0.0011) (Additional file 4: Figure f-g). Our qualitative assessment suggests that call center workers have some of the lowest total steps/wakeful time.

### Cardiometabolic and fitness outcomes

A summary of cardiometabolic outcomes across occupational groups is shown in Additional file 5: Table S4. All meta-analyses and funnel plots for cardiometabolic and fitness outcomes are available in Additional file 4: Figures S7-S18.

#### Body mass index (BMI)

**Fig. 4:**
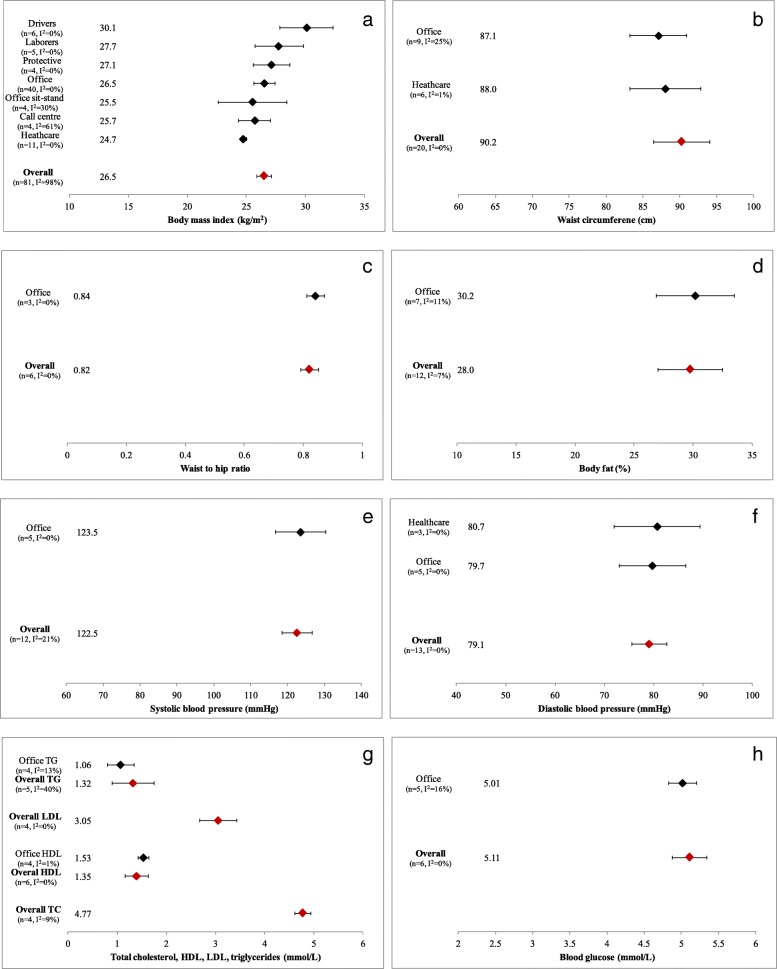
**a**-**h** Body mass index (**a**), waist circumference (**b**), waist-to-hip ratio (**c**), body fat percentage (**d**), systolic blood pressure (**e**), diastolic blood pressure (**f**), total cholesterol, high density lipoprotein, low density lipoprotein and triglycerides (**g**), and blood glucose (**h**) overall (red diamonds) and by occupation group (black diamonds). HDL - high density lipoprotein, I^2^ - measure of heterogeneity, LDL - low density lipoprotein, n - number of studies, TC - total cholesterol, TG – triglycerides

A meta-analysis of BMI found that workers had an average BMI of 26.5 kg/m^2^ (95% CI: 25.9–27.1, I^2^ = 98%, Egger’s *p* = 0.2176) (Fig. 4b, Additional file 4: Figure S7). Drivers had a significantly higher average BMI than office workers (30.1 vs. 26.5 kg/m^2^, Z = 2.9060, *p* = 0.0037), office workers with sit-stand desks (30.1 vs. 25.5 kg/m^2^, Z = 2.4322, *p* = .0150), call center workers (30.1 vs. 25.7 kg/m^2^, Z = 2.7118, *p* = 0.0067), and healthcare workers (30.1 vs. 24.7 kg/m^2^, Z = 4.6298, *p* = 0.0000) (Additional file 4: Figure S7c-j). Qualitatively, BMI tended to be higher among occupations with a greater proportion of men such as protective services (e.g. fire fighters, soldiers), laborers (e.g. construction workers, farmers) and drivers (e.g. taxi, truck drivers).

#### Waist circumference

A meta-analysis of waist circumference found that workers had an average waist circumference of 90.2 cm (95% CI: 86.4–94.0, I^2^ = 0%, Egger’s *p* = 0.17461) (Fig. 4b, Additional file 4: Figure S8a-d). No significant differences were found between office workers and healthcare workers. Qualitatively, waist circumference tended to be higher among laborers and drivers.

#### Waist-to-hip ratio (WHR)

A small (*N* = 6 [3 office]) meta-analysis (Fig. 4c) found that workers had an average WHR of 0.82 (95% CI: 0.79–0.85, I^2^ = 0%) (Additional file 4: Figure S9a-d). Office workers had a significant higher WHR than all other workers (0.84 vs. 0.80, Z = 2.4778, *p* = 0.0132) (Additional file 4: Figure S9c-d).

#### Body fat percentage

Twelve studies (9 office) reported on percentage of body fat. A meta-analysis found that workers had an average body fat percentage of 29.7% (95% CI: 27.0–32.5%, I^2^ = 7%, Egger’s *p* = 0.01855) (Fig. 4d). Office workers were no different than all other workers (30.2% vs. 29.2%, Z = 0.3, *p* = 0.7489) (Additional file 4: Figure S10c-d).

#### Blood pressure (BP)

Twelve studies had enough BP data to be included in a meta-analysis. Workers had an average SBP of 122.5 mmHg (95% CI: 118.4–126.6, I^2^ = 21%, Egger’s *p* = 0.03538) (Fig. 4e) and an average DBP of 79.1 mmHg (95% CI: 75.5–82.7, I^2^ = 0%, Egger’s *p* = 0.01109) (Fig. 4f). There is evidence of publication bias for these studies (Additional file 4: Figures S11b and S12b). Qualitative assessments indicate that occupations which rely heavily on driving vehicles appeared to have higher BPs and healthcare workers appeared to have lower BPs.

#### Blood lipids

Few studies provided sufficient total cholesterol (*N* = 4 [3 office]), triglycerides (*N* = 5 [4 office]), HDL (N = 6 [4 office]), or LDL (N = 4 [2 office]) data to be included in meta-analyses. Workers had an average total cholesterol level of 4.77 mmol/L (95% CI: 4.61–4.93, I^2^ = 9%), triglycerides of 1.32 mmol/L (95% CI: 0.89–1.75, I^2^ = 40%); HDL of 1.39 mmol/L (95% CI: 1.16–1.63, I^2^ = 0%) and, LDL of 3.05 mmol/L (95% CI: 2.67–3.43, I^2^ = 0%) (Fig. 4g, Additional file 4: Figures S13a-b, S14a-b, S15a-b, S16a-b).

#### Blood glucose, HbA1c and HOMA-IR

A limited number of studies provided adequate data to be included in the blood glucose meta-analyses; five of the six included data from office worker populations. The average blood glucose concentrations across workers were 5.11 mmol/L (95% CI: 4.88–5.34, I^2^ = 0%) (Fig. 4h, Additional file 4: Figure S17a-b). Single studies reported on HbA1c, [89] 2-h glucose [31] and HOMA-IR [31].

#### Peak V̇*O*_*2*_

Five studies (three in office workers) reported comparable data for peak V̇O_2_ and were included in a meta-analysis. Workers exhibited an overall peak V̇O_2_ of 32.7 mL/min/kg (95% CI: 30.5–34.9, I^2^ = 0%); there was no statistically significant difference between studies (Additional file 4: Figure S18a-b).

## Discussion

This systematic review examined and compared device-measured PA and ST, and cardiometabolic health and fitness indicators across occupation groups. Working adults spent about 60% of their working and waking time in sedentary behaviour; a very small proportion (~ 4%) of the day included MVPA. On average, workers fell within the 7000 to 11,000 steps/day range associated with good health [161]. Office and call center workers’ steps/day were among the lowest, while labourers and postal delivery workers had the most. We found several occupational group-related differences in levels of ST, LPA and MVPA at work and during wakeful time. Occupations that were desk-based (i.e. office workers) had the highest ST and the lowest number of steps, LPA (work and wakeful time) and MVPA (work). They did however, have more daily minutes of MVPA compared to all other workers. Laborers had the lowest ST and spent a significantly greater proportion of their work time in LPA and MVPA. Healthcare and protective services workers had higher levels of LPA at work (and wakeful time in healthcare) compared to other workers. Workers in driving-based occupations tended to have higher BMIs and BPs. While office workers had a significantly higher WHR than other workers, their percentage of body fat did not appear to differ.

As mentioned, similar investigations have been limited to blue vs. white-collar occupational classifications only. Such previous systematic review evidence (that included self-report and device-measured outcomes) identified that white-collar workers (e.g. desk-based professionals) were more likely to engage in higher amounts of occupational ST and lower levels of occupational PA [20]. White-collar workers have also been shown engage in higher leisure-time PA compared to blue-collar workers [18]. We also found that office workers had higher occupational ST and lower occupational PA compared to blue-collar workers (e.g. laborers). Similar to our findings, higher step counts have been observed in blue-collar workers compared to white-collar workers [163]. Our finding that office workers had greater minutes of MVPA during wakeful time suggests that they are likely spending a greater amount of time engaged in non-work (e.g. travel, leisure) MVPA. However, given that evidence suggests a substantial amount (60–75 min) of daily MVPA is required to overcome large amounts of ST [162], office workers remain at health risk due to their high ST.

Adults who are employed in occupations with long work hours, stressful conditions and who engage in low levels of occupational PA are at the greatest risk for lower leisure-time PA [18, 164]. Those participating in manual labor are more likely to engage in sedentary behaviour in their leisure-time, whereas office workers are less likely to engage in these behaviours in leisure-time [165]. Lunde et al. found that construction workers had a greater number of minutes/day of sitting in their leisure-time/outside of work than within (282 vs. 157 min/day) [94]. Mansoubi et al. found that office workers engaged in a greater proportion of MVPA and a lower proportion of ST outside of work hours than during [96].

Higher levels of occupational, leisure-time and total PA are shown to be associated with a reduced risk of all-cause mortality, especially among women [166]. Additionally, higher levels of occupational sitting have been associated with a greater risk for diabetes and mortality [9]. However, the “PA paradox” suggests that occupational PA may not confer the same benefits as leisure-time PA as it may: occur at too low of intensity; elevate BP as a result of heavy lifting and static postures; not provide adequate recovery time; and, be performed outside of a worker’s control (e.g. work tasks, protective clothing, stressors, surrounding environment) [13, 167].

Globally, occupation-related energy expenditure and PA has decreased substantially since the 1960s and is projected to continue to decline [168–171]. Given that the majority of working adults’ wakeful time is spent on the job, workplaces are opportune settings for PA. Previous research has shown that workplace interventions may increase MVPA, decrease ST and improve cardiometabolic health indicators among workers [172–176]. In addition, recent review evidence suggests that workplace interventions may improve mental health [177, 178], the ability to perform occupational tasks fully [179], and adverse musculoskeletal symptoms (e.g. neck and shoulder pain) [176, 180–182], though higher quality studies are still needed to identify the most promising interventions. Evidence has also shown that increasing PA levels may be advantageous to employers through reduced absenteeism [183, 184] and increased productivity [185]. The findings of this review therefore highlight the need for workplace and public health interventions to promote these behaviours both within and outside of work (i.e., active travel and leisure/recreation) and their cardiometabolic sequelae among working adults. It is also a call to action to recognize that more physically demanding occupations may not provide the same opportunity for health-enhancing PA. It is important that future research examines lower intensities of PA, the postural components of occupational work to understand the effects on health (e.g. prolonged standing, walking) and consider measuring sleep as part of the 24-h movement continuum.

Strengths of this review include a comprehensive search strategy developed with a research librarian (SV), an a priori established protocol with inclusion and exclusion criteria including device-measured movement intensities, cardiometabolic and fitness outcomes, the assessment of risk of bias, and the use of meta-analyses to provide overall summary measures across occupational groups. This review builds on previous research by focusing on device-measured outcomes, including varying intensities of PA and ST thereby removing sources of potential biases (recall and response bias). While device measures overcome biases, there was variation in the monitors used and cut-points/algorithms applied to determine intensity; we used a random effects model to account for such heterogeneity.

A limitation of this review is the small number of studies available for the meta-analyses of MPA, VPA, and the majority of the cardiometabolic outcomes. Further, there were insufficient studies to compare movement intensities across all specific occupations or occupational groups. There is a need for further studies to characterize the movement patterns (including comparisons between leisure- and work-specific movement and describing postural changes and bout duration of movement intensities) and cardiometabolic health and fitness in occupations, especially those that are not office workers. Future work would also benefit from examining the data in a more compositional manner looking at the 24-h daily period and how different behaviours at work might influence those outside. It is also important to recognize the limitations of many of the devices used which have the potential to misclassify behaviours. The cut-point thresholds employed, especially for the accelerometers, were often developed in controlled laboratory settings performing standard activities (e.g. walking on a treadmill) rather than during occupational tasks. This review included self-reported (largely BMI) and objectively measured cardiometabolic outcomes from studies which reported on the primary outcome of interest. Therefore, there is the potential for response bias. The meta-analytic software was unable to produce funnel plots and an Egger’s test for the steps meta-analyses due to large standard deviations and small sample sizes (S. Tarlow [Comprehensive Meta-Analysis support], personal communication, August 20, 2018). We were unable to perform a sex-specific analysis as most studies did not report on male and female data separately. Our review did not examine any of the reasons for why PA and ST differed by occupation. However, research suggests that the differences are likely affected by an individual’s work and leisure-time hours. During work hours, PA is most influenced by the type of tasks performed as part of the job, whereas, during leisure-time, socioeconomic background is an important determinant [186]. This is likely why there is a higher amount of leisure-time PA in white-collar workers. There was evidence of publication bias for many of the behaviour outcomes, as well as for body mass and BP, indicating that smaller studies had larger effect sizes. As most studies were smaller (*N* < 100) and non-representative, there is a need for larger, more representative studies examining device-measured PA and ST in occupational groups. Lastly, wide confidence intervals for many of the outcomes are indicative of heterogeneity among several occupation groups within each analysis and thereby reduce our confidence in the estimates. Finally, it is likely that our categorizations have misclassified some studies into the wrong occupation group. For example, we included nurses, nurses’ aides, physicians, diagnosticians and physiotherapists under healthcare workers. These groups may involve different types of work tasks.

## Conclusion

This review identified that work-based and wakeful PA and ST differed between occupations. Office workers spent the greatest amount of time at work and throughout wakeful time sedentary, but also spend the most minutes/day in MVPA. Healthcare workers engage in more LPA, and laborers and postal delivery workers in more work time-specific MVPA. Future studies are needed to assess whether these patterns differ between men and women within the same occupation, describe leisure-time specific PA and ST and contrast them to occupational-specific PA and ST, and whether they translate to different levels of risk for poor cardiometabolic health. Ultimately, awareness that these health behaviours differ between occupations calls for the need to include workplace interventions that promote health-enhancing PA both at work and during leisure-time.

## Additional files

Additional file 1: **Table S1.** Sample Ovid MEDLINE search strategy. (DOCX 24 kb)

Additional file 2: **Table S2.** Included study characteristics. (DOCX 82 kb)

Additional file 3: **Table S3.** Physical behaviour outcomes by occupation group. (DOCX 100 kb)

Additional file 4: **Figures S1–S18.** Individual meta-analyses and funnel plots for behaviour, cardiometabolic and fitness outcomes. (PDF 658 kb)

Additional file 5: **Table S4.** Cardiometabolic and fitness outcomes by occupation group. (DOCX 54 kb)
