# Supplementary material for: Device-measured physical activity, sedentary behaviour and cardiometabolic health and fitness across occupational groups: a systematic review and meta-analysis
Source: Int J Behav Nutr Phys Act. 2019 Apr 2;16:30. doi: 10.1186/s12966-019-0790-9 (PMC6444868; doi:10.1186/s12966-019-0790-9)
Supplement: Supplementary file 1 — Table S1. Sample Ovid MEDLINE search strategy. (DOCX 24 kb) [file 12966_2019_790_MOESM1_ESM.docx]

Table S1. Sample Ovid MEDLINE search strategy.

| 1 | | exp Occupations/ | |
| --- | --- | --- | --- |
| 2 | | Occupational Health/ | |
| 3 | | exp occupational groups/ | |
| 4 | | Work/ | |
| 5 | | Workplace/ | |
| 6 | | working.tw. | |
| 7 | | workplace?.tw. | |
| 8 | | occupation*.tw. | |
| 9 | | professional.tw. | |
| 10 | | job?.tw. | |
| 11 | | employ*.tw. | |
| 12 | | or/1-11 | |
| 13 | | Motor Activity/ | |
| 14 | | exp Exercise/ | |
| 15 | | exercis*.tw. | |
| 16 | | (low adj3 (activit* or intensit* or exertion or aerobic or fit or fitness* or train*)).tw. | |
| 17 | | (light adj3 (activit* or exertion or aerobic or fit or fitness* or train*)).tw. | |
| 18 | | (moderate adj3 (activit* or intensit* or exertion or aerobic or fit or fitness* or train*)).tw. | |
| 19 | | (vigorous adj3 (activit* or intensit* or exertion or aerobic or fit or fitness* or train*)).tw. | |
| 20 | | ("high intensity" adj3 (activit* or exertion or aerobic or fit or fitness* or train*)).tw. | |
| 21 | | (physical* adj3 (inactiv* or activ* or intens* or exertion or aerobic or fit or fitness* or train*)).tw. | |
| 22 | | (aerobic adj3 (activit* or intens* or exertion or fit or fitness* or train*)).tw. | |
| 23 | | (anaerobic adj3 (activit* or exercis* or intens* or exertion or fit or fitness* or train*)).tw. | |
| 24 | | (strength adj3 (activit* or exercis* or intens* or exertion or fit or fitness* or train*)).tw. | |
| 25 | | walk*.tw. | |
| 26 | | (step? adj2 (day? or week?)).tw. | |
| 27 | | sedentary.tw. | |
| 28 | | or/13-27 | |
| 29 | | Monitoring, Ambulatory/ | |
| 30 | | exp Accelerometry/ | |
| 31 | | acceleromet*.tw. | |
| 32 | | inclinomet*.tw. | |
| 33 | | activity monitor*.tw. | |
| 34 | | ((fitness or activity) adj1 track*).tw. | |
| 35 | | actigraph*.tw. | |
| 36 | | activpal*.tw. | |
| 37 | | actimet*.tw. | |
| 38 | | actical.tw. | |
| 39 | | actiheart.tw. | |
| 40 | | pedomet*.tw. | |
| 41 | | bodymedia.tw. | |
| 42 | | Geneactiv.tw. | |
| 43 | | objective* measure?.tw. | |
| 44 | | doubly label?ed water.tw. | |
| 45 | | (step? adj3 count*).tw. | |
| 46 | | or/29-45 | |
| 47 | | 12 and 28 and 46 | |
| 48 | | exp Child/ or Adolescent/ | |
| 49 | | exp Adult/ | |
| 50 | | 48 not 49 | |
| 51 | | 47 not 50 | |
