# Supplementary material for: Device-measured physical activity, sedentary behaviour and cardiometabolic health and fitness across occupational groups: a systematic review and meta-analysis
Source: Int J Behav Nutr Phys Act. 2019 Apr 2;16:30. doi: 10.1186/s12966-019-0790-9 (PMC6444868; doi:10.1186/s12966-019-0790-9)
Supplement: Supplementary file 2 — Table S2. Included study characteristics. (DOCX 82 kb) [file 12966_2019_790_MOESM2_ESM.docx]

Table S2. Included study characteristics

| **First author, year** | **Country** | **Occupation group(s)** | **Age**  **mean (SD) or range** | **Study design** | **N_analyzed_ (N or %)** | | | **Movement behaviour** | | | | | **Cardio-metabolic outcomes (SR or OM)** |
| --- | --- | --- | --- | --- | --- | --- | --- | --- | --- | --- | --- | --- | --- |
|  |  |  |  |  | **Women** | **Men** | **Total** | **Behaviour** | **Units** | **Monitor used (type + brand)** | **Wear time** | |  |
| Aandstad, 2016 | Norway | Home guard soldiers | 33 (5), 24-44 | C-S | 0 | 411 | 411 | MPA, VPA, steps | Mins/weekday, mins/weekend day, steps/weekday, steps/weekend day | Accelerometer (SenseWear Armband Pro 2) | | 2 week days and 1 weekend day, 24 hours/day | BMI (OM) |
| Abd, 2012 | USA | Physicians (general cardiologists, cardiothoracic surgeons, procedural cardiologists, cardiac anesthesiologists) | 39.5 (9.1) | C-S | 2 (7%) | 26 (93%) | 28 | Steps | Steps/workday | Pedometer, NR | | 2 weeks, work hours | BMI, WC, DBP, HR (SR) |
| Alkhajah*,* 2012 | Australia | Office workers | I: 33.5 (8.7); C: 39.9 (7.2), 20-65 | Quasi-experimental | 29 (91%) | 3 (9%) | 32 | ST | Mins/8 hr workday, mins/ 16 hr workday | Inclinometer (ActivPAL3) | | 7 days, 24 hours/day | BM, BMI, WC, TC, HDL, TG, glucose (OM) |
| Andersen, 2011 | Norway | Taxi drivers | 34.3 (7.7), 25-60 | C-S | 0 (0%) | 71 (100%) | 71 | ST, MVPA | Hours/day, mins/day | Accelerometer (MTI model 7164) | | 7 days, waking hours | BMI, WC, HDL-C, LDL-C, TG, glucose, glucose 2h, HOMA-IR, VO_2_ (OM) |
| Arias, 2015 | USA | Construction workers | 40 (11) | C-S | 0 (0%) | 55 (100%) | 55 | ST, LPA, MPA, VPA | Mins/day, % of day | Accelerometer (ActiGraph GT3X) | | 7 days, waking hours | BMI (NR) |
| Arias, 2017 | USA | Patient care workers | 42 (13) | C-S | 41 (87%) | 6 (13%) | 47 | ST, LPA, MPA, VPA | Mins/day, % of day | Accelerometer (ActiGraph GT3X) | | 7 days. waking hours | BMI (NR) |
| Arundell, 2018 | Australia | Office workers | 40.1 (85) | Pre-post | 101 (72%) | 39 (28%) | 140 | ST, LPA, MVPA | Mins/8 hr day | Accelerometer (ActiGraph GT3X) | | 5 working days, work hours | NR |
| Atkinson, 2005 | Scotland | Physicians | NR | C-S | NR | NR | 16 | Steps | Steps/day | Accelerometer (ActiGraph GT3X) | | 7 days. 8-12 hr shifts | NR |
| Badland, 2004 | New Zealand | Office workers | 24-45 | C-S | 29 (52%) | 27 (48%) | 56 | Steps | Steps/workday, steps/non- workday, total steps/ day | Pedometer (Yamax Digiwalker SW-700) | | 3 days; work hours & waking hours | NR |
| Balogh, 2004 | Sweden | Cleaners, office workers | NR | C-S | 0 (0%) | 48 cleaners, 41 office workers (100%) | 89 | ST | % of work day | Posimeter (Posimeter 100) | | 2 days, work hours | NR |
| Bassey, 1983 | United Kingdom | Factory workers | Women: 58.1 (0.2); Men: 57.7 (1.4) | C-S | NR | NR | 59 | Steps | Steps/ day | Pedometer, NR | | 7 days, waking hours | BM (NR) |
| Bergman, 2018 | Sweden | Office workers | I: 52.4 (6.8), C: 50.3 (6.7), 40-67 | RCT | 44 (55%) | 36 (45%) | 80 | ST, LPA, MVPA, steps | Mins/day, steps/day | Accelerometer (ActiGraph GT3X) and inclinometer (ActivPAL3) | | ActiGraph: 14 days, waking hours; ActivPAL: 7 days, 24 hours | NR |
| Bird, 2015 | Australia | University staff | 53 (7) | C-S | NR | NR | 10 | ST, LPA, MPA, VPA | % of workday | Accelerometer (Body Media SenseWear) | | 5 days, waking hours | BMI (SR) |
| Brakenridgem, 2016 | Australia | Office workers | 38.9 (8) | Quasi-experimental | 70 (46%) | 83 (54%) | 153 | ST, steps | Mins/10hr workday, mins/16hr day, steps/10hr workday, steps/16hr day | Inclinometer (ActivPAL3) | | 7 days, 24hours/day | BMI (OM) |
| Brett, 2017 | Scotland | Administrative workers, academic staff | NR | Pre-post | 16 (80%) | 4 (20%) | 20 (administrative: 10; academic: 10) | Steps | Steps/day | Pedometer (Silva Ex Step) | | 7 days, waking hours | NR |
| Brewer, 2016 | USA | Physical therapists | Outpatient: 30.65 (5.55); inpatient: 32.3 (9.01) | C-S | Outpatient: 11 (18%); inpatient: 27 (44%) | Outpatient: 20 (33%); inpatient: 3 (5%) | 61 (outpatient: 31; inpatient 30) | ST, steps, LPA, MVPA, | Steps/ workday, % of workday | Accelerometer (ActiGraph GT3XP) | | 4-5 days, work hours | BM, BMI (NR) |
| Brown*,* 2013 | Australia | Office workers | 40.7 (11.2) | C-S | 76 (70%) | 32 (30%) | 108 | ST, LPA, MVPA | Hours/day, % of day, mins/day (MVPA) | Accelerometer (ActiGraph GT3X+) | | 7 days, waking hours | BMI, WC (NR) |
| Carr, 2016 | USA | Office workers with & without access to sit-stand desks | 44.1 (10.7) | C-S | 74% | 26% | 69 (sit: 38; sit-stand: 31) | ST, steps | Hours/ day, steps/day | Inclinometer (activPAL3) | | 5 days, mins/18 hours | BM, BMI, WC, %BF, SBP, DBP, VO_2_ (OM) |
| Chae, 2015 | South Korea | Office workers | 38.2 (8.1) | Pre-post | 35 (50%) | 35 (50%) | 70 | Steps | Steps/day | Pedometer (Yamax Digiwalker SW-700) | | Unknown, waking hours | BMI, WHR (OM) |
| Chan, 2018 | New Zealand | Healthcare workers | 41.4 (11.6), 22-64 | Prospective | 36 (74%) | 13 (26%) | 49 | Steps | Steps/day | Pedometer (Omron HJ-203) | | 7 days, waking hours | BMI (SR) |
| Chappel, 2016 | Australia | Firefighters | 37.8 (10.9), 20-60 | C-S | 8 (24%) | 26 (76%) | 34 | ST, LPA, MPA, VPA | Mins/ workday | Accelerometer (Actical) | | 28 days, 24 hours/day | BM, BMI (SR) |
| Chastin, 2009 | United Kingdom | Postal workers (deliver & office) | Delivery:40 (9); office: 40 (7) | C-S | Delivery: 5 (13%); office: 6 (15%) | Delivery: 34 (87%); office: 33 (85%) | 78 (delivery: 39; office: 39) | Steps | Steps/day | Inclinometer (activPAL) | | 7 days, 24 hours/day | BM, BMI (SR) |
| Chau, 2014 | Australia | Office workers | 38 (11) | RCT | 36 (86%) | 6 (14%) | 38 | ST | Mins/day | Inclinometer (activPAL3) | | 7 days, work hours | BM (SR) |
| Chau, 2016 | Australia | Call center workers | 33 (10.8) | Quasi-experimental | 14 (45%) | 17 (55%) | 31 | ST, steps, LPA, MVPA | Mins/workday | Inclinometer (activPAL3) and accelerometer (ActiGraph GT3X) | | 7 days, work hours | BMI (SR) |
| Cheung, 2008 | Hong Kong | School teachers | I: 38.9 (10.8); C: 26.5 (1.9) | Quasi – experimental | 41 (79%) | 11 (21%) | 52 (I: 38; C: 14) | Steps | Steps/ day | Pedometer (Yamax Digiwalker SW-700) | | 5 days, waking hours | BM, BMI, WHR, %BF (OM) |
| Cheung, 2012 | Hong Kong | School teachers | 35.9 (10.8) | C-S | NR | NR | 71 (PE teacher: 18; non-PE: 53) | Steps | Steps/day | Pedometer (Yamax Digiwalker SW-700) | | 3 days, waking hours | BMI, WHR, %BF (OM) |
| Chitkara, 2014 | Canada | Call center workers | 50.5 (10.7) | C-S | 15 (79%) | 4 (21%) | 19 | ST, LPA, MPA, VPA | Min/day | Accelerometer (Actical) | | 7 days, waking hours | BM, BMI, WC (OM) |
| Choi, 2010 | South Korea | Navy submarine crew | 3.5 (7.7), 22-57 | C-S | 0 | 76 (100%) | 109 (submarine crew: 76; command personnel: 33) | Steps | Steps/day | Pedometer (DMC-03) | | 1 month, waking hours | NR |
| Clark, 2011 | Australia | Office workers, call center workers, submarine crew | Median 34.9 (IQR: 28.5-46.0) (office: 35.5 (IQR: 30.6-47.3); call centre: 29.2 (IQR: 25.0-40.1); customer service: 39.5 (IQR: 27.6-53.5)) | C-S | 73 (60%) (Office: 54 (66%); call centre: 10 (42%); customer service: 9 (60%)) | NR | 121 (office: 82; call centre: 24; customer service: 15) | ST | Hours/ workday | Accelerometer (ActiGraph GT1M) | | 7 days, work hours | BMI (OM) |
| Clays, 2017 | Denmark | Manufacturers, garbage collectors, mobile plant operators, construction workers | 44.9 (9.9) | C-S | 86 (43.4%) | 112 (56.6%) | 198 | MVPA | % of working day | Accelerometer (ActiGraph GT1M) | | 4 days, working hours | NR |
| Clemes*,* 2014a | UK | Office workers | 37 (13) | C-S | 60% | 40% | 72 | Steps | Steps at work, steps outside of work (on work days) | Pedometer (Yamax Digiwalker SW-700) | | 7 days, waking hours | BMI (OM) |
| Clemes, 2014b | UK | Office workers | 40.1 (12.7) | C-S | 70% | 30% | 170 | ST, LPA, MVPA | mins/day, % for workdays and non-workdays | Accelerometer (ActiGraphGT1M) | | 7 days, waking hours | BMI (OM) |
| Cole*,* 2015 | UK | Desk-based workers (software engineers) | NR | C-S | NR | NR | 5 | ST, steps, MVPA | Mins/day, steps/day | Accelerometer (NR) | | 1 week, NR | NR |
| Copeland, 2017 | USA | Acute care Nurses | 34.4 (9.8) | Pre-post | 24 (92%) | 2 (8%) | 26 | Steps | Steps/shift | Pedometer (NR) | | 3 months, working hours | NR |
| Croteau, 2017 | USA | Nurses and nurse support staff | 47.6 (7.1) | Pre-post | 19 (95%) | 1 (5%) | 20 | Steps | Steps/day, steps/workday, steps/non-workday | Pedometer (Accusplit AE120x) | | 7 days, waking hours | BMI, WC (NR) |
| Cuddy*,* 2015 | USA | Firefighters | 26 (3) | C-S | 3 (20%) | 12 (80%) | 15 | ST, LPA, MVPA | % of wear time | Accelerometer (Actical) | | 3 days, working hours | BM (OM) |
| Danquah, 2017 | Denmark, Greenland | Office workers with access to sit-stand desk | 46 (10), 19-64 | RCT | 210 (66%) | 107 (44%) | 317 | ST, steps, MVPA | workday/ leisure sitting time (mins/ 8 hr), steps (n/h), leisure MVPA (mins/ 8hr) | Accelerometer (ActiGraph GT3X+) | | 5 days, work hours | BMI, WC, %BF (OM) |
| De Jong, 2018 | USA | Office workers | 31.7 (1.3), 19-45 | Randomized cross-over | NR | NR | 21 | ST, LPA, steps | Hours/day (%), steps/non-workday, steps/workday | Inclinometer (activPAL3) and accelerometer (ActiGraph GT3X) | | 3 days, 24 hours/day | BMI, glucose, %BF (OM) |
| Dollman, 2016 | Australia | Farmers, office workers | Office: 46.9 (8.5); Farmer: 50.7 (9.7), 30-65 | C-S | 0 (0%) | 57 (100%) | 57 (farmers: 29; office: 28) | ST | Hours/ day | Inclinometer (activPAL) | | 3 days, work hours | BMI (OM) |
| Donath, 2015 | Switzerland | Office workers with access to sit-stand desk | I: 45 (12); C: 40 (10), 18-65 | RCT | 23 (74%) | 8 (26%) | 31 | ST | Hours/ week | Accelerometer (ActiGraph GT3X+) | | 5 days, work hours | BM, BMI, %BF (OM %BR, NR on all others) |
| Evans, 2012 | UK | Office workers | I: 49 (8); C: 39 (10) | RCT | 22 (79%) | 6 (21%) | 28 | ST | Hours/ day (%) | Inclinometer (activPAL) | | 5 days, work hours | BMI (NR) |
| Finkelstein, 2015 | Singapore | Office workers | Cash: 35.5 (8.4); Charity: 35.5 (8.6); Control: 35.6 (8.6); Fitbit Only: 35.4 (8.3), 21-65 | RCT | Cash: 113 (57.4%); Charity: 105 (52.8%); Control: 112 (55.7%); Fitbit only: 100 (49.3%) | Cash: 84 (42.6%); Charity: 94 (47.2%); Control: 89 (44.3%); Fitbit only: 103 (50.7%) | 800 (Cash: 197; Charity: 199; Control: 201; Fitbit only: 203) | ST, steps, LPA, MPA, VPA | Steps/ day, mins/ day | Accelerometer (ActiGraph GT3X+) | | 7 days, waking hours | BM, BMI, SBP, DBP, VO_2_ (OM) |
| Fisher, 2018 | UK | Office workers | 39.4 (10.7) | C-S | 54% | 42% | 131 | ST, steps | Mins/workday, steps/workday | Inclinometer (ActivPAL) | | 3 days, 24 hours/day | BMI (OR) |
| Foley, 2016 | Australia | Office workers | 38.1 (NR), 22-63 | Cross-over | 38 (43%) | 50 (57%) | 56 | ST, steps, LPA, MVPA | % time in behavior/ mean wear time per day, step counts per hr at work | Accelerometer (ActiGraph GT3X+) | | 5 days, work hours | BMI (SR) |
| French*,* 2007 | USA | Transit workers | 47.6 (10.2), 19-79 | RCT | 22% | 78% | 158 | ST, LPA, MVPA | Mins/ day | Accelerometer (ActiGraph GT3X+) | | 4 days, NR | BM, BMI (OM) |
| Gany*,* 2014 | USA | Taxi drivers | 47.9 (NR) | RCT | 0 (0%) | 47 (100%) | 47 | Steps | Steps/day | Pedometer (HJ-303 OMROM) | | 4, NR | SBP, DBP, TC, HDL-C, TC-HDL, glucose (OM) |
| Gao*,* 2016 | Finland | Office workers with sit-stand desks | 37.7 (10.5), 24-62 | C-S | 14 (58%) | 10 (42%) | 24 | ST, LPA, MVPA | % of day | EMG electrodes derived muscle unit (Myontec Ltd, Kuopio) | | 1 workday, NR | BM, BMI, WHR (OM) |
| Gilson, 2010 | Australia | Office workers | 44 (10.2) | Quasi-experimental | 32 (68%) | 15 (32%) | 47 | Steps | Steps/ day | Pedometer | | NR. NR | NR |
| Gilson*,* 2012a | Australia, Canada, Northern Ireland, USA | Office workers | 45.5 (10.8) | Pre-post | 288 (69%) | 102 (31%) | 330 | Steps | Steps/ day | Pedometer (Yamax SW-200) | | NR, NR | BMI (NR) |
| Gilson*.* 2012b | Australia | Office workers | 46.9 (9.8) | Quasi-experimental | 7 (64%) | 4 (36%) | 11 | ST, LPA, MVPA | % of day | Accelerometer (Body Media SenseWear Pro2) | | 1 week, waking hours | BMI (SR) |
| Gilson*,* 2016 | Australia | Office workers | 47 (11) | Quasi-experimental | 11 (20%) | 46 (80%) | 57 | ST, LPA, MVPA | Hours/ workday, mins/day, % of day | Accelerometer, sitpad (GENEActiv) | | One working week, 24 hours/day | BMI, WC, SBP, DBP (OM) |
| Gilson*,* 2017 | Australia | Truck drivers | 44.4 (10) | Quasi-experimental | 0 (0%) | 19 (100%) | 19 | ST | % of work time, % nonwork day, leisure time, work time | Accelerometer (GENEActive) | | One working week, 24 hours/day | BMI, WC, SBP, DBP (OM) |
| Gorman, 2013 | Canada | Office workers | 34.5 (8.1) | Pre-post | 75% | 25% | 27 | ST | Mins/ 8 hr workday | Inclinometer (activPAL3) | | 7 days, 24 hours | BM, % BF, HDL-C, TG, glucose (OM) |
| Gram*,* 2016 | Denmark | Construction workers | NR | RCT | NR | NR | NR | ST, LPA, MVPA | % work time, % leisure time | Accelerometer (Actiheart) | | 7 days, 24 hours | NR |
| Hadgraft*,* 2016 | Australia | Office workers | 45.5 (9.4) | RCT | 155 (69%) | 76 (31%) | 229 | ST, MVPA | % of time, mins/ day | Inclinometer (ActivPAL3), accelerometer (ActiGraph) | | 7 days, 24 hours | NR |
| Hallman*,* 2017 | Denmark | Workers from cleaning, transportation, & manufacturing sectors | 44.8 (9.8) | C-S | Cleaning (24%); manufacturing (61%); transportation ( 97%) | Cleaning ( 86%); manufacturing (39%) ; transportation (3%) | 625 | Leisure PA, occupational PA | % of time | Accelerometer (ActiGraph GT3X+) | | 4-5 days, 24 hours | BMI (OM) |
| Hallman, 2018 | Sweden | Office workers | 45.3 (8.6), 18-65 | Natural intervention | 50 | 60 | 110 | ST | % of working time | Accelerometer (ActiGraph GT3X) | | 5-8 days, waking hours | BMI (SR) |
| He, 2013 | USA | Nursing aids | 33.9 (11.5) (8-hr day: 34.5 (13.5); 8-hr evening: 27 (4.3); 8hr night: 32.5 (12.5); 12hr day: 34.7 (11.3); 12hr night: 42.1 (10.2)) | C-S | 22 (100%) | 0 (0%) | 22 | ST, steps | % time, steps/ hr, % of shift | Inclinometer (activPAL) | | 1 shift, 8 or 12 hours | BM, WC (OM) |
| Healy*,* 2013 | Australia | Office workers | 43.2 (10.3), 26-62 | Quasi-experimental | 23 (56%) | 19 (44%) | 43 | ST, steps, MVPA | Mins/8r workday day, MVPA MET mins/ hr workday, step n/8hr workday | Inclinometer (activPAL3) | | 7 days, 24 hours | BMI (OM) |
| Healy, 2016 | Australia | Office workers | 45.6 (9.4) | Cluster RCT | 158 (68%) | 73 (32%) | 229 | ST | % of work time, min/work time, min/wake time | Inclinometer (activPAL3) | | 7 days, 24 hours/day | BMI (OM) |
| Hogstedt Danquah, 2017 | Denmark & Greenland | Office workers with sit-stand desks | 46 (10) (I: 47 (10); C: 46 (11)) | RCT | 210 (66%) | 107 (34%) | 317 (I: 173; C: 144) | ST, steps, MVPA | Mins/ 8hr day, MVPA min/8hr leisure time, Step n/hours leisure time | Accelerometer (ActiGraph GT3X+) | | 5 days, 24 hours/day | BMI (NR) |
| James*,* 2009 | UK | General practitioners | NR | RCT | 22 (51%) | 21 (49%) | 43 | Steps | Steps/day | Pedometer (Omron HJ-104) | | 5 days, 24 hours/day | NR |
| Jancey*,* 2016 | Australia | Office workers | 40.3 (11.9) | Pre-post | 27 (64%) | 15 (36%) | 42 | ST, steps, LPA, MPA, VPA | Mins/ day, steps/ day, % of work time | Accelerometer (ActiGraph GT3X+) | | 5 days, workday | NR |
| Jirathananuwat*,* 2017 | Thailand | Nurse practitioners & nurse managers | 35. 8 (10.8) (NP: 26.8 (4.1); managers: 44.6 (7.6)) | C-S | 283 (97.9%) (NP: 136 (95.7%); managers: 147 (100%) | 6 (2%) (NP: 6 (2%); managers: 0 (0%)) | 289 | ST, LPA, MPA, VPA | Min/day | Accelerometer (FeelFit) | | 5 days, 24hours/day | BMI (OM) |
| Ju, 2011 | USA | Dry cleaner owners | 55.8 (7.6) | C-S | 48 (50%) | 48 (50%) | 96 | Steps | Steps/ day | Pedometer (New Lifestyles -800) | | 7 days, waking hours | NR |
| Julin, 2011 | Finland | Construction workers | 37 (NR) | C-S | 2 (8%) | 25 (92%) | 27 | Steps | Steps/day | Accelerometer (SenseWear Armband) | | 1 workday | BMI (NR) |
| Kirk, 2016 | Scotland | Office workers | 43 (12) | C-S | 27 (93%) | 2 (7%) | 29 | ST | Hours/day, % of day, steps/day | Inclinometer (activPAL) | | 7 days, waking hours | BMI (NR) |
| Kloster, 2017 | Denmark & Greenland | Office workers | 46 (10) | C-S | 201 (63%) | 102 (37%) | 317 | ST | Mins/ 8hr workday | Accelerometer (ActiGraph GT3X+) | | 5 days, 8hr workday | BMI, WC (OM) |
| Koepp*,* 2013 | USA | Office workers | 42 (9.9) | Prospective | 25 (69%) | 11 (31%) | 36 | ST | Mins/ day | Accelerometer (Actical) | | 7 days, waking hours | BM, WC, %BF, SBP, DBP, TC, HDL-C, LDL-C, TG, glucose, HbA1c (OM) |
|  |  |  |  |  |  |  |  |  |  |  | |  |  |
| Korshoj*,* 2013 | Denmark | Cleaners | 37.5 (13.4) | C-S | 20 (100%) | 0 (0%) | 20 | Steps | Steps/ day | Accelerometer (Actiheart) & pedometer (Yamax Digiwalker SW 801) | | 3 days, 24 hours/day | BM, BMI, WC, WHR, SBP, DBP, VO_2_ (OM) |
| Kozey-Keadle, 2012 | USA | Office workers | 46.5 (10.8) | Pre-post | 15 (75%) | 5 (25%) | 20 | ST, steps, MVPA | % of day, steps/day | Accelerometer (ActiGraph GT3X+) & Pedometer (OMROM HJ720-ITC) | | 7 days, waking hours | BMI (NR) |
| Lagersted-Olsen*,* 2014 | Denmark | Office workers | 40.9 (8.6) | C-S | 12 (46%) | 14 (54%) | 26 | ST | Hours/ day, % of workday | Accelerometer (ActiGraph GT3X+) | | 7 days, working hours | BM, BMI, WC, WHR, %BF, SBP, DBP, VO_2_ (OM) |
| Larouche, 2018 | USA | Office workers | 39.4 (10.7) | Randomized cross-over | 15 (79%) | 4 (21%) | 19 | ST, LPA, MVPA | Min/8-hr workday | Inclinometer (activPAL) | | 5 work days, working hours | BMI (SR) |
| Li, 2017 | Australia | Office workers | 42 (10) | RCT | 20 (77%) | 6 (23%) | 26 | ST, steps | % of wear time, steps/day | Inclinometer (activPAL) | | 3 days, 8hr workday | BM, BMI (NR) |
| Loef, 2018 | Netherlands | Healthcare workers | 18-65 | C-S | 83-88% | 12-17% | 479 | ST | % of leisure time, % of work time | Accelerometer (ActiGraph GT3X+) | | 7 days, waking hours | NR |
| Lunde*,* 2017 | Norway | Construction workers, healthcare workers | Construction: 39.9 (13.6); healthcare: 44.5 (9.6) | Prospective | Construction (1.6%) healthcare (73.8%) | Construction (98.4%); healthcare (22.2%) | 124 (construction: 51; healthcare: 63) | ST | Mins/day | Accelerometer (ActiGraph GT3X+) | | 3-4 days, workday | BMI (SR) |
| Mansi*,* 2015 | New Zealand | Meat processing workers | I: 43 (14.9); C: 40 (12.2) | RCT | I: 19 (65.5%); C: 15 (51.7%) | I: 10 (34.5%); C: 14 (48.3%) | 58 (I: 29; C: 29) | Steps | Steps/day | Pedometer (Yamax Digiwalker SW-200) | | 7 days, waking hours | BM, BMI, WC, %BF, SBP, DBP (OM) |
| Mansoubi, 2016 | UK | Office workers | Women: 32.3 (7.9); Men: 31.5 (8.6) | Longitudinal | 22 (55%) | 18 (45%) | 40 | ST, LPA, MVPA | % wear time, mins/day | Inclinometer (activPAL) & Accelerometer (ActiGraph) | | 2 weeks, waking hours | BM, BMI, WC, %BF (OM) |
| Martinez, 2013 | Switzerland | Obstetricians | Median: 33 | Prospective | 11 (61%) | 7 (39%) | 18 | Steps | Steps/day | Accelerometer (ActiGraph GT1M) | | NR, waking hours | BMI (NR) |
| Miller*,* 2004 | Australia | Managers, administrative workers, scientists, technicians, blue-collar workers | 18–29 yr: 20.5%; 30–44 yr: 40.5%; 45–59 yr: 36.8%; 60–75 yr: 2.2% | C-S | 111 (60%) | 74 (40%) | 185 (managers: 43; administrative: 42; scientists: 56; technicians: 33; blue-collar: 11) | Steps | Steps/day | Pedometer (Yamax Digiwalker SW700) | | 7 days, waking hours | NR |
| Mitsui*,* 2010 | Japan | Office workers | 43.6 (10.8) | Prospective | 0 (0%) | 50 (100%) | 50 | Steps | Steps/day | Pedometer (Yamasa EM-180) | | 7 days, waking hours | BM, BMI (OM) |
| Murphy*,* 2015 | Ireland | Radiologists & clinicians | NR | C-S | 22 (radiologists: 10; clinicians: 12) | 28 (radiologists: 15; clinicians: 13) | 50 (radiologists: 25; clinicians: 25) | Steps | Steps/day | Accelerometer (FitBit Ultra) | | 1 day, 8 hours | NR |
| Neil-Sztramko, 2017 | Canada | Physiotherapists | 40.8 (10.2) | C-S | 36 (94.7%) | 2 (5.3%) | 38 | ST, MVPA | Mins/ week, MVPA mins/ week, ST hours/ week | Accelerometer (ActiGraph GT3X+) | | 7 days, waking hours | BMI (SR) |
| Neuhaus, 2014 | Australia | Desk-based office workers | 42.5 (11.5) | C-S | 37 (84%) | 7 (16%) | 44 | ST, steps | Mins/ 8hr day, steps/8 hr workday | Inclinometer (activPAL3) | | 7 days, 24 hours/day | BMI (OM) |
| Noojen, 2018 | Sweden | Office workers | 41 (IQR: 35-48) | C-S | 65% | 35% | 311 | ST | Hours/day, % wear time | Inclinometer (activPAL3) | | 7 days, 24 hours/day | NR |
| Olsen, 2018 | Australia | Office workers | 39.5 (8.7), 26-59 | Pre-post | 67% | 33% | 24 | ST, LPA, MVPA | Mins/day | Accelerometer (ActiGraph GT3X+) | | 7 days, 24 hours/day | NR |
| Parry, 2013a | Australia | Office workers | 36.4 (8.6) | C-S | 42% | 58% | 50 | ST, LPA, MVPA | Mins/day, hours/day, % wear time | Accelerometer (Actical) | | 7 days, 24 hours/day | BMI (NR) |
| Parry*,* 2013b | Australia | Clerical workers, call centre, data processing workers | 43.5 (6.4) | RCT | 50 (80.6%) | 12 (19.4%) | 62 | ST, LPA, MVPA | % of wear time, % of work hours | Accelerometer (ActiGraph GT3X+), Pedometer (NR) | | 7 days, waking hours | BMI (OM) |
| Peavler, 2012 | USA | Desk-based workers | I: 42.6 (8.9); C: 47.6 (9.9) | RCT | I: 86.9%; C: 94.1% | I: 13.1%; C: 5.9% | 40 (I: 23; C: 17) | ST, LPA, MPA, VPA | Mins/ day, % wear time | Activity monitor (StepWatch) | | 7 days, waking hours | BM, BMI (OM) |
| Pedersen*,* 2016 | Denmark | Office workers | 46 (11) | RCT | 100 (74%) | 63 (26%) | 153 | ST, steps, MVPA | ST mins/day, steps/day, MVPA mins/day | Accelerometer (ActiGraph GT3X+) | | 5 week days, 24 hours/ day | BMI (NR) |
| Pedersen*,* 2016 | Australia | Desk-based workers | 45.6 (11.0), | C-S | 28 | 6 | 34 | ST | % wear time | Inclinometer (activPAL) | | 1 8-hr workday, work time | NR |
| Pontt, 2015 | Australia | Office workers & famers | 50.7 (9.7) | RCT | 0 (0%) | 58 (100%) | 58 (office: 29; farmers: 29) | ST, steps | Hours/day, steps/day | Inclinometer (activPAL) | | 3 days, 24 hours/day | BMI (NR) |
| Rafferty, 2016 | UK | Office workers | 38, (23-65) | C-S | 17 | 9 | 26 | Steps, MVPA | Min/day, steps/day | Inclinometer (activPAL) | | 7 days, waking hours | NR |
| Reed, 2018a | Canada | Nurses | 43 (12) | C-S | 23 (6%) | 387 (94%) | 410 | ST, LPA, MPA, VPA, MVPA | Mins/day, % of wear time | Accelerometer (ActiGraph GT3X+) | | 7 days, waking hours | BM, BMI, WC, SBP, DBP (OM) |
| Reed, 2018b | Canada | Nurses | 46 (11) | RCT | 74 (97%) | 2 (4%) | 76 | MVPA, steps | Mins/day, steps/day | Accelerometer (Tractivity) | | 1 week, waking hours | BM, BMI, WC, %BF, SBP, DBP, HR (OM) |
| Ryan*,* 2011 | Scotland | Office workers | 40 (10) | Observational | 58 (70%) | 25 (30%) | 83 | ST | Mins/day | Inclinometer (activPAL) | | 7 days, working hours | BM, BMI (NR) |
| Ryde, 2013 | Australia | Office workers | 40.9 (11.5) | C-S | 68 (64.8%) | 37 (35.2%) | 105 | ST, MVPA (total, at work) | Mins/ day | Accelerometer (ActiGraph GT3X+) | | 7 days, working hours | BMI, WC (OM) |
| Sawyer*,* 2017 | England | Office-based workers | 39.6 (10.5) | C-S | 61 (54%) | 52 (46%) | 115 | ST, steps | Hours/day, steps/day, steps/working hr | Inclinometer (activPAL) | | 5 days, working hours | BMI (OM) |
| Schnebly, 2017 | USA | Nurses | 35.7 (9.5) | C-S | 36 (97.3%) | 1 (2.7%) | 37 | Steps | Steps/ workday | Pedometer (Free-Step Counter and Walking Activity Tracker for Sport and Fitness by App Holdings) | | 3 days, working hours | NR |
| Schofield, 2005 | New Zealand | Office, retail, university academic, university allied, nurse/aid, blue collar (mechanics, green keepers, dry cleaners) | NR | C-S | 121 (67%) | 60 (33%) | 181 (office: 63; retail: 10; uni. academic: 40; uni. allied: 48; nurse/aid: 11; blue collar: 9) | Steps | Steps/day | Pedometer (Yamax Digiwalker SW-700) | | 3 days, waking hours & working hours | NR |
| Schulz, 2018 | USA | Farmers | 52.8 (14.7) | C-S | 0 | 40 (100%) | 40 | MPA | Mins/day | Accelerometer (ActiGraph GT3X) | | 7 days, waking hours | BMI (SR) |
| Schwartz, 2016 | Canada | Office workers | 45.7 (9.4) | C-S | 88.7% | 11.3% | 97 | ST, MVPA | % of wake time | Accelerometer (ActiGraph GT3X) | | 7 days, NR | BMI, BF% (OM) |
| Smith, 2002 | England, Northern Ireland, Scotland, Wales | Postmen, nurses, technicians, civil servants, professional engineers, administration, university researchers, teachers, lecturers, managers, secretaries, accounting, housewives, software programmers | Postmen: 28.4 (6.0); nurses: 32.5 (9.9); technicians: 39.7 (9.8); civil servants: 36.5 (10.3); professional engineers: 38.1 (12.4); administration: 38.5 (11.0); university researchers: 27.6 (2.4); teachers: 44.6 (12.2); lecturers: 47.8 (8.8); managers: 50.9 (6.1); secretaries: 53.4 (7.6); accounting: 47.8 (9.4); housewives: 58.4 (14.6); software programmers: 33.5 (11.6) | C-S | 152 | 141 | 293 (Postmen: 7; nurses: 15; technicians: 12; civil servants: 8; professional engineers: 16; administration: 13; university researchers: 12; teachers: 18; lecturers: 11; managers: 9; secretaries: 9; accounting: 7; housewives: 14; software programmers: 11) | Steps | Steps/day | Pedometer (NR) | | 2 weeks, waking hours | NR |
| Smith, 2015 | England | Office workers | 39 (10.6) | C-S | 55% | 45% | 164 | ST, steps | Hours/day, steps/day | Inclinometer (activPAL) | | 4 days, 24 hours/day | NR |
| Soh*,* 2006 | New Zealand | Anesthesiologists | NR | C-S | NR | NR | NR | Steps | Steps/ workday | Pedometer (Sportline 340) | | 5 days, working hours | NR |
| Steeves*,* 2015 | USA | *See note | NR, 20-60 | C-S | 477*** | 635** | 1112 | ST, LPA, MVPA | % of time | Accelerometer (ActiGraph AM-7164) | | 7 days, waking hours | NR |
| Steeves, 2018 | USA | †See note | 40.5 (SE = .5) | C-S | 45.1% | 54.9% | 1465†† | ST, MVPA, steps | Mins/week, % wake time, steps/day | Accelerometer (ActiGraph AM-7164) | | 7 days, waking hours | BMI (NR) |
| Straker*,* 2013 | Sweden | Call center workers | 34.3 (10) | C-S | 68 | 63 | 131 | ST | % of time | Inclinometer (Posimeter 100) | | 1 shift, working hours | BMI (NR) |
| Stephens, 2018 | Australia | Desk-based workers | 44.5 (9.0) | Cluster RCT | 88 (66%) | 46 (34%) | 121 | ST | Mins/8-hr workday | Inclinometer (activPAL3) | | 7 days, 24 hours/day | NR |
| Sudholz, 2018 | Australia | Office workers | 18+ | C-S | 32 (54%) | 27 (46%) | 59 | ST | Mins/day | Inclinometer (activPAL3) and accelerometer (ActiGraph GT3X) | | 8 days, 24 hours/day | BMI (SR) |
| Swartz, 2014 | USA | Office workers | 44.3 (11.1) | RCT | 68% | 32% | 60 | ST, steps | Mins/ workday, steps/ workday | Inclinometer (activPAL) | | 3 days, work hours | BM, BMI (OM) |
| Talbot, 2011 | USA | National Guard Personnel | FFL: 32.7 (10.1); TRAD: 32.8 (8.3) | RCT | FFL: 15 (31%); TRAD: 9 (19%) | FFL: 33 (68%); TRAD: 37 (80%) | 94 | Steps | Steps/day | Pedometer (Yamax SW 200) | | 7 days, waking hours | BMI, SBP, DBP, TC, HDL-C, LDL-C, TC-HDL (OM) |
| Taylor, 2016 | USA | Office workers | 43.4 | Cluster RCT | 82% | 18% | 175 | ST, steps | Mins/weekday, mins/weekend day, steps/week | Pedometer (Lifestyles Digiwalker SW200) | | 7 days, waking hours | BM, BMI, WC, TC, HDL-C, LDL-C, TG, SBP, DBP (OM) |
| Thorp, 2012 | USA | Office workers, customer service, and call centre workers | 37.3 (Office: 38.2 (10); call centre: 32.3 (10.3); customer service: 38.5 (12.6)) | C-S | 123 (office: 91 (70%); call centre: 25 (46%); customer service: 17 (74%)) | 64 (office: 40 (30%); call centre: 8 (54%); customer service: 6 (26%)) | 187 (office: 131; call centre: 33; customer service: 23) | ST, LPA, MVPA | % work day | Accelerometer (ActiGraph GT1M) | | 7 days, waking hours | NR |
| Tigbe, 2011 | UK | Delivery postal workers, office workers | Delivery:38 (9); office: 40 (7) | C-S | Delivery: 5 (9%); office; 10 (18%) | Delivery: 51 (91%); office: 46 (82%) | 112 (delivery: 56; office: 56) | ST, step | Hours/day, steps/day | Inclinometer (activPAL3) | | 7 days, 24 hours/day | BMI (NR) |
| Tobin*,* 2016 | Australia | Office workers | I: 34.8 (10.5); C: 34.3 (8.9) | Pre-post | 32 (86%) | 5 (14%) | 37 | ST, steps | Mins/day, % of day, steps/day | Inclinometer (activPAL3) | | 7 days, working hours | BMI (SR) |
| Toomingas*,* 2012 | Sweden | Call center operators | 34.4 (10.6), 18-62 | C-S | 97 (69%) | 43 (31%) | 140 | ST | % of day | Inclinometer (Posimeter 100) | | 1 day, working hours | BM, BMI (SR) |
| Torquati, 2018 | Australia | Nurses | 41.4 (12.1) | C-S | 41 (87%) | 6 (13%) | 47 | ST, LPA, MVPA, steps | Mins/day, % of wake time, steps/day | Accelerometer (ActiGraph GT3X+) | | 7 days, NR | BM, BMI, WC, SBP, DBP (OM) |
| Umukoro, 2013 | USA | Patient care workers (nurses, personal care assistants) | 42 (12) | C-S | 41 (85%) | 7 (15%) | 48 | ST, LPA, MPA, VPA | Mins/work time, % of work time, Mins/day | Accelerometer (ActiGraph GT3X) | | 7 days, waking hours | NR |
| Urda, 2016 | USA | Office workers | 48 (10) | Baseline data from RCT | 44 (100%) | 0 (0%) | 44 | ST | Hours/workday, % of workday | Inclinometer (activPAL3) | | 7 days, 24 hours/day | BMI (OM) |
| Van Dommelen, 2016 | Netherlands | Financial services, research institutes, construction workers | Financial: 42.8 (10.1); research: 47.4 (9.3); construction: 48.9 (9.1) | Baseline data from RCT | Financial: 31 (39%); research: 57 (66%); construction: 0 (0%) | Financial: 49 (61%); research: 30 (34%); construction:38 (100%) | 205 (financial: 80; research: 87; construction: 38) | ST, LPA, MPA, VPA, MVPA | % of wear time | Accelerometer (ActiGraph) | | 7 days, waking hours | BMI (SR) |
| Varela-Mato*,* 2016a | UK | Bus drivers | 44 (27) | C-S | NR | NR | 28 | ST | Mins/day, % of day | Inclinometer (activPAL3) | | 7 days, waking hours | BMI, WC, %BF, SBP, DBP, HR (OM) |
| Varela-Mato, 2016b | Australia | Truck driver | 49 (14) | C-S | 0 (0%) | 77 (100%) | 77 | ST, MVPA | Hours/day, mins/day | Inclinometer (activPAL3) & accelerometer (ActiGraph GT3X) | | 7 days, NR | BMI (OM) |
| Varela-Mato, 2017a | UK | Bus drivers | 43.9 (27) | C-S | 0 (0%) | 28 (100%) | 28 | ST | Mins/day | Inclinometer (activPAL3) & accelerometer (ActiGraph GT3X) | | 7 days, 24hours/day | BMI (OM) |
| Varela-Mato, 2017b | UK | Truck driver | Median: 50 (1QR: 25, 65) | C-S | 0 (0%) | 87 (100%) | 87 | ST, steps, LPA, MVPA | Mins/day, steps/day | Inclinometer (activPAL3) | | 7 days, 24 hours/day | BMI, WC, WHR, %BF, SBP, DBP, HR, TC, HDL-C, LDL-C, TG, glucose (OM) |
| Vincent*,* 2016 | Australia | Firefighters | 46.1 (11.4) | C-S | 9 (23%) | 31 (77%) | 40 | ST, LPA, MPA, VPA | Mins/ shift | Accelerometer (Actical) | | NR, work hours | BMI (SR) |
| Waters, 2016 | Singapore | Office workers | 43 (9) | C-S | 24 (60%) | 13 (40%) | 37 | ST, steps, LPA, MPA, MVPA | Mins/day, steps/day | Accelerometer (ActiGraph GT3X+) | | 7 days, 10 hours | BMI, WC (OM) |
| Weiler*,* 2015 | UK | Professional football players | 26.8 (4.4) | C-S | 0 (0%) | 25 (100%) | 25 | ST, LPA, MPA, VPA | Mins/ day | Accelerometer (GENEActiv triaxial wrist) | | 7 days, waking hours except training/ matches | BM, BMI (NR) |
| Wick, 2016 | Switzerland | Office workers with access to sit-stand desks | 40.8 (11.4), 18-65 | C-S | 30 (78.9%) | 8 (21.1%) | 28 | ST | % of day | Accelerometer (ActiGraph GT3X+) | | 7 days, work hours | BMI (OM) |
| Wieters, 2009 | USA | Office workers | >18 | Case-control | Urban: 153 (72.5%); suburban: 193 (73.1%) | Urban: 58 (27.5%); suburban: 71 (26.9%) | 475 (urban: 211; suburban: 264) | Steps | Steps/ day | Pedometer (Walk4Life Classic) | | 6 days, waking hours | NR |
| Wong, 2014 | Australia | Transportation drivers | 52.4 (9.69) | C-S | 0 (0%) | 23 (100%) | 23 | ST, LPA, MVPA | % of day, hours/ day | Accelerometer (ActiGraph GT3X) | | 7 days, waking hours | BMI (NR) |
| Ying*,* 2014 | USA | Office workers | 25-64 | C-S | 69% | 31% | 26 | ST, steps | % of day, steps/ day | Accelerometer (ActiGraph GT3X) | | 3 weekdays, work hours | NR |
| Zhu, 2018 | USA | Office workers | 39.1 (11.3) | C-S | 27 (75%) | 9 (25%) | 36 | ST, LPA, MVPA | Mins/8-hr work day | Inclinometer (activPAL3c) | | 7 days, 24-hours/day | BM, BMI, SBP, DBP, glucose, TC, HDL-C, LDL-C, TG |

Abbreviations: %BF – body fat percentage, BM – body mass, BMI – body mass index, BP – blood pressure, DBP – diastolic blood pressure, C-S – cross-sectional, HbA1c – haemoglobin A1c, HDL-C – high density lipoprotein cholesterol, HOMA-IR – homeostatic model assessment insulin resistance, HR – heart rate, LDL-C – low density lipoprotein cholesterol, LPA – light intensity physical activity, mins – minutes, MPA – moderate intensity physical activity, MVPA – moderate to vigorous intensity physical activity, NA – not applicable, NR – not reported, OM – objectively measured, PCS – prospective cohort study, RCT – randomized controlled trial, SBP – systolic blood pressure, SD – standard deviation, SR – self-report, ST – sedentary time, TC – total cholesterol, TC-HDL – total cholesterol to HDL ratio, TG – triglycerides, WC – waist circumference, WHR – waist to hip ratio, VO_2max_ – maximum rate of oxygen consumption, VPA – vigorous intensity physical activity

* Occupation Groups:

(1) Farm and nursery workers; (2) helpers, cleaners, hand packagers, laborers; (3) construction laborers; (4) agricultural, forestry, fishing; (5) cleaning and building service occupations; (6) construction trades; (7) Freight, stock, and material movers (hand); (8) Farm operators, managers and supervisors; (9) Textile, apparel, furnishings machine operators; (10) Machine operators, assorted materials; (11) Waiters and waitresses; (12) Other mechanics and repairers; (13) Motor vehicle operators; (14) Supervisors and proprietors, sales occupations; (15) Fabricators, assemblers, inspectors, and samplers; (16) Other transportation and material moving; (17) Private household occupations; (18) Vehicle and mobile equip. mechanics, repairers; (19) Material recording, scheduling, distributing clerks; (20) Cooks; (21) Miscellaneous food preparation and service; (22) Extractive and precision production occupations; (23) Laborers, except construction; (24) Sales workers, retail and personal services; (25) Health service occupations; (26) Sales reps., finance, business, & commodities; (27) Technicians and related support occupations; (28) Information clerks; (29) Health diagnosing, assessing and treating; (30) Executive, administrators, and managers; (31) Writers, artists, entertainers, and athletes; (32) Personal service occupations; (33) Management related occupations; (34) Teachers; (35) Protective service occupations; (36) Engineers, architects and scientists; (37) Miscellaneous administrative support occupations; (38) other professional specialty occupations; (39) Records processing occupations; (40) Secretaries, stenographers, and typists

** Total N (N or %):

1112 ((1) Farm and nursery workers: 6; (2) helpers, cleaners, hand packagers, laborers: 18; (3) construction laborers: 9; (4) agricultural, forestry, fishing: 21; (5) cleaning and building service occupations: 23; (6) construction trades: 58; (7) Freight, stock, and material movers (hand): 14; (8) Farm operators, managers and supervisors: 5; (9) Textile, apparel, furnishings machine operators: 8; (10) Machine operators, assorted materials: 22; (11) Waiters and waitresses: 7; (12) Other mechanics and repairers: 37; (13) Motor vehicle operators: 61; (14) Supervisors and proprietors, sales occupations: 27; (15) Fabricators, assemblers, inspectors, and samplers: 28; (16) Other transportation and material moving: 21; (17) Private household occupations: 9; (18) Vehicle and mobile equip. mechanics, repairers: 15; (19) Material recording, scheduling, distributing clerks: 19; (20) Cooks: 24; (21) Miscellaneous food preparation and service: 14; (22); Extractive and precision production occupations: 47; (23) Laborers, except construction: 3; (24) Sales workers, retail and personal services: 26; (25) Health service occupations: 38; (26) Sales reps., finance, business, & commodities: 29; (27) Technicians and related support occupations: 33; (28) Information clerks: 18; (29) Health diagnosing, assessing and treating: 42; (30) Executive, administrators, and managers: 112; (31) Writers, artists, entertainers, and athletes: 15; (32) Personal service occupations: 14; (33) Management related occupations: 49; (34) Teachers: 40; (35) Protective service occupations: 13; (36) Engineers, architects and scientists: 29; (37) Miscellaneous administrative support occupations: 65; (38) other professional specialty occupations: 39; (39) Records processing occupations: 33; (40) Secretaries, stenographers, and typists: 21)

*** Total women (N or %):

(1) Farm and nursery workers: 1; (2) helpers, cleaners, hand packagers, laborers: 6; (3) construction laborers: 0; (4) agricultural, forestry, fishing: 0; (5) cleaning and building service occupations: 8; (6) construction trades: 1; (7) Freight, stock, and material movers (hand): 3; (8) Farm operators, managers and supervisors: ; (9) Textile, apparel, furnishings machine operators: 5; (10) Machine operators, assorted materials: 5; (11) Waiters and waitresses: 5; (12) Other mechanics and repairers: 0; (13) Motor vehicle operators: 2; (14) Supervisors and proprietors, sales occupations: 8; (15) Fabricators, assemblers, inspectors, and samplers: 12; (16) Other transportation and material moving: 1; (17) Private household occupations: 9; (18) Vehicle and mobile equip. mechanics, repairers: 0; (19) Material recording, scheduling, distributing clerks: 7; (20) Cooks: 11; (21) Miscellaneous food preparation and service: 8; (22) Extractive and precision production occupations: 9; (23) Laborers, except construction: 2; (24) Sales workers, retail and personal services: 14; (25) Health service occupations: 31; (26) Sales reps., finance, business, & commodities: 13; (27) Technicians and related support occupations: 15; (28) Information clerks: 14; (29) Health diagnosing, assessing and treating: 34; (30) Executive, administrators, and managers: 45; (31) Writers, artists, entertainers, and athletes: 6; (32) Personal service occupations: 11; (33) Management related occupations: 26; (34) Teachers: 29; (35) Protective service occupations: 3; (36) Engineers, architects and scientists: 7; (37) Miscellaneous administrative support occupations: 49; (38) other professional specialty occupations: 23; (39) Records processing occupations: 33; (40) Secretaries, stenographers, and typists: 21)

**** Total men (N or %):

(1) Farm and nursery workers: 5; (2) helpers, cleaners, hand packagers, laborers: 12; (3) construction laborers: 9; (4) agricultural, forestry, fishing: 21; (5) cleaning and building service occupations: 15; (6) construction trades: 57; (7) Freight, stock, and material movers (hand): 11; (8) Farm operators, managers and supervisors : 4; (9) Textile, apparel, furnishings machine operators: 3; (10) Machine operators, assorted materials: 17; (11) Waiters and waitresses: 2; (12) Other mechanics and repairers: 37; (13) Motor vehicle operators: 59; (14) Supervisors and proprietors, sales occupations: 19; (15) Fabricators, assemblers, inspectors, and samplers: 16; (16) Other transportation and material moving: 20; (17) Private household occupations: 0; (18) Vehicle and mobile equip. mechanics, repairers: 15; (19) Material recording, scheduling, distributing clerks: 12; (20) Cooks: 13; (21) Miscellaneous food preparation and service: 6; (22) Extractive and precision production occupations: 38; (23) Laborers, except construction: 1; (24) Sales workers, retail and personal services: 12; (25) Health service occupations: 7; (26) Sales reps., finance, business, & commodities: 16; (27) Technicians and related support occupations: 18; (28) Information clerks: 4; (29) Health diagnosing, assessing and treating: 8; (30) Executive, administrators, and managers: 67; (31) Writers, artists, entertainers, and athletes: 9; (32) Personal service occupations: 3; (33) Management related occupations: 23; (34) Teachers: 11; (35) Protective service occupations: 10; (36) Engineers, architects and scientists: 22; (37) Miscellaneous administrative support occupations: 16; (38) other professional specialty occupations: 16; (39) Records processing occupations: 0; (40) Secretaries, stenographers, and typists: 0)

† Occupation Groups:

(1) Farming, fishery, forestry; (2) building & grounds cleaning, maintenance; (3) construction, extraction; (4) transportation, material moving; (5) food preparation, serving; (6) installation, maintenance, repair; (7) personal care, service; (8) production; (9) education, training, library; (10) arts, design, entertainment, sports, media; (11) protective service; (12) sales & related; (13) business, financial operations; (14) architecture, engineering; (15) management; (16) healthcare practitioner, technical; (17) office, administrative support; (18) life, physical, social science; (19) health care support; (20) computer, mathematical; (21) community, social services; (22) legal

†† Total N (N or %):

1465; (1) Farming, fishery, forestry: 6; (2) building & grounds cleaning, maintenance: 43; (3) construction, extraction: 127; (4) transportation, material moving: 117; (5) food preparation, serving: 47; (6) installation, maintenance, repair: 62; (7) personal care, service: 43; (8) production: 134; (9) education, training, library: 63; (10) arts, design, entertainment, sports, media: 19; (11) protective service: 43; (12) sales & related: 117; (13) business, financial operations: 68; (14) architecture, engineering: 36; (15) management: 120; (16) healthcare practitioner, technical: 69; (17) office, administrative support: 218; (18) life, physical, social science: 19; (19) health care support: 49; (20) computer, mathematical: 32; (21) community, social services: 21; (22) legal: 15
