# Supplementary material for: Device-measured physical activity, sedentary behaviour and cardiometabolic health and fitness across occupational groups: a systematic review and meta-analysis
Source: Int J Behav Nutr Phys Act. 2019 Apr 2;16:30. doi: 10.1186/s12966-019-0790-9 (PMC6444868; doi:10.1186/s12966-019-0790-9)
Supplement: Supplementary file 3 — Table S3. Physical behaviour outcomes by occupation group. (DOCX 100 kb) [file 12966_2019_790_MOESM3_ESM.docx]

Table S3. Physical behaviour outcomes by occupation group

| **First author, year** | **Occupation group(s)** | **Sample size analyzed** | **Sedentary time (mean ± SD or median [IQR])** | **Mean light PA (mean ± SD or median [IQR])** | **Mean moderate PA (mean ± SD or median [IQR])** | **Mean vigorous PA (mean ± SD or median [IQR])** | **Mean MVPA (mean ± SD or median [IQR])** | **Mean steps per day (mean ± SD or median [IQR]** |
| --- | --- | --- | --- | --- | --- | --- | --- | --- |
| **Academics** | | | | | | | | |
| Academic university staff | 10 | 68.1% [57.8-83.5%] of work day | 10.4% [8.5-26.8%] of work day | 8.0 [6.7-18.7%] of work day | 0.7 [0.1-2.1%] of work day | - | - |  |
| Academic/research staff | 10 | - | - | - | - | - | 9264.20 ± 3151.37 |  |
| University academic | 40 | - | - | - | - | - | **Steps/day at work:**   - University academic: 4422 ± 1380   **Steps/day outside of work:**   - 5280 ± 2885   **Total steps/day:**   - 9702 ± 3129 |  |
| University researchers, Lecturers | Researchers: 12, lecturers: 11 | - | - | - | - | - | **University researchers:** 7708 ± 2840  **Lecturers:** 6818 ± 2040 |  |
| **Call centre workers** | | | | | | | | |
| Chau, 2016 | Call centre workers | ActivPAL:  C: 6; I: 5 ActiGraph:  C: 1; I: 3 | **ActivPAL sitting time:**   - C: 345 ± 86 min work time - I: 294 ± 127 min work time   **ActiGraph ST:**   - C: 430 ± 56 min work time I: 427 ± NR min work time | - | - | - | **ActiGraph**   - I: 28 ± 1 min work time - C: 15 ± 5 min work time | **ActivPAL**   - C: 1916 ± 784 - I: 1167 ± 943   **ActiGraph:**   - C: 2193 ± 897 - I: 3651 ± NA |
| Chitkara, 2014 | Call centre workers | 19 | 1166.6 ± 106.5 min/day | 181.2 ± 76.9 min/day | 91.6 ± 44.9 min/day | 0.9 ± 3.3 min/day | - | 5361 ± 2509 |
| Clark, 2011 | Office workers, call centre workers, customer service workers | 24 | 6.63 ± 0.73 h/work day | - | - | - | - | - |
| Straker, 2013 | Call centre workers | 131 | - Sit-stand: 78.5% [64.2-84.2] - Sit: 83.8% [76.7-86.8] | - | - | - | - | - |
| Thorp, 2012 | Call centre employees | 33 | 71.2% ± 7.1% of work day | 4.6% ± 2.9% of work day | - | - | 1.8% ± 1.8% of work day | - |
| Toomingas, 2012 | Call centre operators | 140 | **Sitting:** 75% ± 16.6% | - | - | - | - | - |
| **Customer service - hospitality** | | | | | | | | |
| Clark, 2011 | Customer service workers | 121 | 5.79 ± 0.73h/work day | - | - | - | - | - |
| Schofield, 2005 | Retail | 10 | - | - | - | - | - | **Steps/day at work:**   - 8384 ± 3123   **Steps/day outside of work:**   - 5535 ± 2299   **Total steps/day:**   - 13919 ± 3696 |
| Steeves, 2015 | Waiters and waitresses  Cooks  Miscellaneous food preparation and service  Sales workers, retail and personal services  Sales reps., finance, business, & commodities | Waiters and waitresses 7;  Cooks 24;  Miscellaneous food preparation and service 14;  Sales workers, retail and personal services 26;  Sales reps., finance, business, & commodities 29 | **Waiters and waitresses:** 39.8% ± 3.2%  **Cooks**: 47.9% ± 2.6%  **Miscellaneous food preparation and service:** 46.7% ± 2.3%  **Sales workers, retail and personal services:** 52% ± 1.5%  **Sales reps., finance, business, & commodities:** 58.2% ± 1.9% | **Waiters and waitresses:** 43.4% ± 3.4%  **Cooks**: 39% ± 2.1%  **Miscellaneous food preparation and service:** 39.2% ± 2.3%  **Sales workers, retail and personal services:** 32.1% ± 0.9%  **Sales reps., finance, business, & commodities:** 27.7% ± 1.3% | - | - | **Waiters and waitresses:** 2.5% ± 0.9%  **Cooks:** 2.2% ± 0.4%  **Miscellaneous food preparation and service:** 2.1% ± 0.4%  **Sales workers, retail and personal services:** 3.1% ± 0. 3%  **Sales reps., finance, business, & commodities:** 3.5% ± 0.6% | - |
| Steeves, 2018 | Food preparation, serving | 47 | 43.8% ± 2.0% | - | - | - | 48.7 ± 14 min/week | 9388 ± 570 |
| Thorp, 2012 | Customer service | 23 | 62.1% ± 6.2% of workday | 4.8% ± 3.0% of workday | - | - | 1.9% ± 0.9% of workday | - |
| **Drivers** | | | | | | | | |
| Andersen, 2011 | Taxi drivers | 71 | 8.4 ± 1.4 h/day | - | - | - | 25 ± 18 min/day | - |
| French, 2007 | Transit workers | 158 | 342.8 ± 6.2 (SE) min/day | 232.5 ± 5.3 (SE) min/day | - | - | 16.7 ± 1.2 (SE) min/day | - |
| Gany, 2014 | Taxi drivers | 47 | - | - | - | - | - | - 3731.9 ± 1685.4 - 3241.4 (median) |
| Gilson, 2017 | Truck drivers | 19 | - Work time: 23% ± 16% - Workday non-work time: 63% ± 15% - Non-workday: 62% ± 8% | - | - | - | - | - |
| Steeves, 2015 | Motor vehicle operators; Other transportation and material moving | 1112 | - Motor vehicle operators: 50% ± 1.5% - Other transportation and material moving: 54.1% ± 2.2% | - Motor vehicle operators: 33.6% ± 1.1% - Other transportation and material moving: 28.8% ± 1.7% | - | - | - Motor vehicle operators: 4.1% ± 0.5% - Other transportation and material moving: 4.2% ± 0.3% | - |
| Steeves, 2018 | Transportation, material moving | 117 | 47.0 ± 1.3% | - | - | - | 44.4 ± 9 min/week | 9960 ± 443 |
| Varela-Mato, 2017 | Bus drivers | 28 | **ActivPAL:**   - Waking work days: 715 ± 123 min/day - Waking non-work days: 536 ± 203 min/day - Working workdays: 478 ± 106 min/day - Non-working workdays: 236 ± 65 min/day   **ActiGraph <50cpm:**   - Waking work days: 480 ± 92 min/day - Waking non-work days: 502 ± 169 min/day - Working workdays: 229 ± 52 min/day - Non-work workdays: 251 ± 75 min/day   **ActiGraph <100:**   - Waking work days: 572 ± 96 min/day - Waking non-work days: 594 ± 199 min/day - Working workdays: 298 ± 65 min/day - Non-work workdays: 274 ± 81 min/day   **ActiGraph <150 cpm:**   - Waking work days: 638 ± 95 min/day - Waking non-work days: 619 ± 205 min/day - Working workdays: 348 ± 68 min/day - Non-work workdays: 290 ± 84 min/day   **ActiGraph <200 cpm:**   - Waking work days: 690 ± 91 min/day - Waking non-work days: 641 ± 212 min/day - Working workdays: 388 ± 69 min/day - Non-work workdays: 302 ± 85 min/day   **ActiGraph <250 cpm:**   - Waking work days: 732 ± 84 min/day - Waking non-work days: 662 ± 216 min/day - Working workdays: 420 ± 70 min/day - Non-work workdays: 312 ± 86 min/day   **ActiGraph <300 cpm:**   - Waking work days: 768 ± 84 min/day - Waking non-work days: 663 ± 217 min/day - Working workdays: 445 ± 71 min/day - Non-work workdays: 323 ± 84 min/day | - | - | - | - | - |
| Varela-Mato, 2016 | Bus drivers | 28 | **Workdays:**   - 724 [112] min/day - 75% [9%]   **Non-workdays:**   - 528 [151] min/day - 62% [10%] | - | - | - | - | - |
| Varela-Mato, 2016 | Lorry drivers | 77 | **Sitting time workdays:**   - 13 ± 2.3 h/day   **Non-workdays:**   - 8.2 ± 3.0 h/day   **At work:**   - 8.0 ± 1.0 h/day   **Non-work:**   - 4.5 ± 1.3 h/day | - | - | - | **Workdays:** 44 ± 30 min/day  **Non-workdays:** 33 ± 39 min/day | - |
| Varela-Mato, 2017 | Lorry drivers | 87 | **On all days:**   - Workday: 749.5 [493.5, 1179.9] min/day - Non-workday: 463.1 [258.0, 787.9] min/day   **On work days only:**   - Working: 491.5 [148.6, 678.2] min/day - Non-working: 239.9 [101.8, 600.4] min/day | **On all days:**   - Workday: 85.3 [48.0, 169.2] min/day - Non-workday: 97.6 [27.2, 317.2] min/day   **On work days only:**   - Working: 39.7 [8.2, 95.8] min/day - Non-working on workdays: 47.0 [9.6, 118.6] min/day | - | - | **On all days:**   - Workday: 12.6 (1.4, 103.5] min/day - Non-workday: 6.0 [0.0, 84.4] min/day   **On workdays only:**   - Working: 4.4 [0.3, 26.2] min/day - Non-working workdays: 5.8 [0.0, 96.0] min/day | **On all days:**   - Workday: 8074.9 [13411.3, 21420.0] - Non-workday: 7854.8 [1008.0, 23308.0]   **On workdays only:**   - Working: 3659.9 [2148.4, 9177.9] - Non-working workdays: 4324.5 [634.0, 17469.0] |
| Wong , 2014 | Transport drivers | 23 | **Off-workdays:**   - 63.5% - 8.9 ± 2.5 h/day   **Workdays:**   - 52.4% - 7.8 ± 1.5 h/day | **Off-workdays:**   - 32.9% - 4.6 ± 1.6 h/day   **Workdays:**   - 43.9% - 6.6 ± 1.3 h/day | - | - | **Off-workdays:**   - 3.6% - 0.5 ± 0.5 h/day   **Workdays:**   - 3.7% - 0.6 ± 0.4 h/day | - |
| **Factory** | | | | | | | | |
| Bassey, 1983 | Factory workers | 59 | - | - | - | - | - | 11600 ± 500 (SE) |
| Mansi, 2015 | Meat processing workers | 58 | - | - | - | - | - | - I: 5993 ± 1234 - C: 5788 ± 1172 |
| Steeves, 2015 | Textile, apparel, furnishings machine operators  Machine operators, assorted materials  Fabricators, assemblers, inspectors, and samplers  Extractive and precision production occupations | Textile, apparel, furnishings machine operators 8;  Machine operators, assorted materials 22;  Fabricators, assemblers, inspectors, and samplers 28;  Extractive and precision production occupations 47 | **Textile, apparel, furnishings machine operators**: 48.9% ± 5.5%  **Machine operators, assorted materials:** 45.9% ± 1.5%  **Fabricators, assemblers, inspectors, and samplers:** 50.1% ± 3.6%  **Extractive and precision production occupations**: 52.4% ± 1.2% | **Textile, apparel, furnishings machine operators:** 31.4% ± 2.0%  **Machine operators, assorted materials:** 36.6% ± 1.5%  **Fabricators, assemblers, inspectors, and samplers:** 33.7% ± 2.0%  **Extractive and precision production occupations:** 31.7% ± 0.8% | - | - | **Textile, apparel, furnishings machine operators:** 5.4% ± 1.5%  **Machine operators, assorted materials:** 3.3% ± 0.6%  **Fabricators, assemblers, inspectors, and samplers:** 3.6% ± 0.5%  **Extractive and precision production occupations:** 3.1% ± 0.3% | - |
| **Healthcare workers** | | | | | | | | |
| Abd, 2012 | Physicians (general cardiologists, cardiothoracic surgeons, procedural cardiologists, cardiac anathesiologists) | 28 (cardiothoracic surgeons = 8, cardiacanathesiologists = 8, general cardiologists = 7, procedural cardiologists = 5) | - | - | - | - | - | **All physicians:** 6010.6 ± 1999.6 steps/day at work  **Cardiothoracic surgeons:** 6038.8 ± 2540 steps/day at work  **Cardiac anesthesiologists:** 5553.3 ± 1762 steps/day at work  **General cardiologists:** 6540.1 ± 2204.3 steps/day at work  **Procedural cardiologists:** 5910.4 ± 1388 steps/day at work |
| Atkinson, 2005 | Physicians | 16 | - | - | - | - | - | **Medical house officers:** 7907 ± 453 steps/shift  **Consultant physicians:** 4647 ± 1968 steps/shift  **Surgical house officers:** 5068 ± 542 steps/shift  **Surgical consultants**: 4822 ± 2174 steps/shift |
| Brewer, 2016 | Physical therapists | 61 | **Inpatient:** 73.8% ± 7.4% of work day  **Outpatient:** 69.2% ± 8.9% of work day | **Inpatient:** 20.5% ± 6.1% of work day  **Outpatient:** 23.1% ± 7.2% of work day | - | - | **Inpatient:** 5.7% ± 2.4% of work day  **Outpatient:** 7.7% ± 5.4% of work day | **Inpatient:** 4475.2 ± 1464.7 at work  **Outpatient:** 3195.0 ± 1333.1 at work |
| Chan, 2018 | Junior doctors, senior doctors, nurses, allied health, staff working in neurology, cardiology, endocrinology | 49 | - | - | - | - | - | 10620 ± 3141 |
| Copeland, 2017 | Acute care nurses | 26 | - | - | - | - | - | 8362.5 ± 2549.4 steps/shift |
| Croteau, 2017 | Nurses and nursing support staff | 20 | **-** | - | - | - | - | Steps/day: 8446 ± 2266  Steps/workday: 10398 ± 2931  Steps/non-work day: 7036 ± 2915 |
| He, 2013 | Nursing aids | 22 | **Percentage of shift:**   - 8 h day: 9.8% - 8 h evening: 17.6% - 8 h night: 34.6% - 12 h day: 21.6% - 12 h night: 31.3% | - | - | - | - | **Steps per hour at work:**   - 8 h day: 1704 - 8 h evening: 1356 - 8 h night: 1101 - 12 h day: 1388 - 12 h night: 763 |
| James, 2009 | General practitioners in pedometer intervention | 44 | - | - | - | - | - | **Men:** 10161 ± 3544  **Women**: 11537 ± 4356  **Total:** 10865 ± 3994 |
| Jirathananuwat, 2017 | Nurse clinical practitioners and nurse managers | 289 | **Total:**   - At work: 494.6 ± 150.8 min/day - Non-work: 258.2 ± 150.6 min/day   **Clinical practitioners:**   - At work: 500.6 ± 160.6 min/day - Non-work: 228.1 ± 153.2 min/day   **Managers:**   - At work: 488.6 ± 140.3 min/day - Non-work: 287.7 ± 142.1 min/day | **Total:**   - At work: 80.8 ± 41.2 min/day - Non-work: 41.8 ± 32.0 min/day   **Clinical practitioners:**   - At work: 89.0 ± 44.5 min/day - Non-work: 34.9 ± 29.8 min/day   **Managers:**   - At work: 72.7 ± 35.9 min/day - Non-work: 48.6 ± 32.6 min/day | **Total:**   - At work: 8.50 ± 7.81 min/day - Non-work: 7.49 ± 7.68 min/day   **Clinical practitioners:**   - At work: 10.24 ± 8.60 min/day - Non-work: 7.94 ± 7.82 min/day   **Managers:**   - At work: 6.79 ± 6.50 min/day - Non-work: 7.05 ± 7.52 min/day | **Total:**   - At work 0.01 ± 0.08 min/day - Non-work: 0.06 ± 1.19 min/day   **Clinical practitioners:**   - At work: 0.01 ± 0.11 min/day - Non-work: 0.08 ± 1.63 min/day   **Managers:**   - At work: 0.01 ± 0.05 min/day - Non-work: 0.04 ± 0.48 min/day | - | **Total work + non-work:**   - 11416.5 ± 4105.4   **Clinical practitioners:**   - 11611.4 ± 4305.3   **Managers:**   - 11229.7 ± 3898.1 |
|  |  |  |  |  |  |  |  |  |
| Loef, 2018 | Healthcare workers (shift and non-shift workers) | 479 | **Leisure time non-shift:**   - 59.5% ± 9.2%   **Leisure time shift:**   - 61.4% ± 9.7%   **Working time non-shift:**   - 63.5% ± 16.5%   **Working time shift:**   - 50% ± 13.7% | - | - | - | - | - |
| Lunde, 2017 | Healthcare workers | 124 | **Sitting at work:** 171.6 ± 93.8 min/day  **Sitting in leisure:** 274.0 ± 94.3 min/day | - | - | - | - | - |
| Martinez de Tejada, 2013 | Obstetricians | 18 | - |  | - | - | - | 7132 [5283-8649] |
| Murphy, 2015 | Radiologists and clinicians | 50 | - | - | - | - | - | Mean difference between clinicians and radiologists: 2985 steps/day, 95% CI: 1853-4117 |
| Neil-Sztramko, 2017 | Public and private practice physiotherapists | 38 | **All participants:**   - Total: 21.3 [15.3-34.5] h/week - Occupational: 4.3 [2.2-6.5] h/week - Non-occupational: 17.9 [11.7-25.4] h/week   **Public practice PTs:**   - Total: 23.4 [17.2-31.9] h/week - Occupational: 5.5 [35.-7.5 ] h/week - Non-occupational: 18.9 [12.8-27.3] h/week   **Private Practice PTs:**   - Total: 20.2 [16.2-23.7] h/week - Occupational: 3.1 [1.0-4.1] h/week - Non-occupational: 16.6 [13.3-24.0] h/week | - | - | - | **All participants:**   - Total: 390.9 [320-509.3] min/week - Occupational: 53.5 [32.3-120] min/week - Non-occupational: 325.5 [234.2-408.2] min/week   **Public practice pts:**   - Total: 458.8 [339.4-549.4] min/week - Occupational: 97.0 [34.3-59.6] min/week - Non-occupational: 361.1 [253.9-385.4] min/week   **Private Practice pts:**   - Total: 357.4 [313.1-465.6] min/week - Occupational: 44.2 [34.3-59.6] min/week - Non-occupational: 303.6 [253.9-385.4] min/week   **MVPA in bouts of 10min:**  **All participants:**   - Total: 158.0 [88.8-251.5] min/week - Occupational: 0.0 [0.0-19.3] min/week - Non-occupational: 153.0 [76.1-245.5] min/week   **Public practice:**   - Total: 188.9 [123.2-329.4] min/week - Occupational: 0.0 [0.0-29.3] min/week - Non-occupational: 177.3 [93.3-288.7] min/week   **Private practice:**   - Total: 165.2 [86.1-202.7] min/week - Occupational: 0.0 [0.0-6.3] min/week - Non-Occupational: 150.6 [78.0-210.8] min/week | - |
| Reed, 2018a | Nurses | 410 | - 445 ± 116 min/day - 49.5% of day | - 408 ± 79 min/day - 45.8% of day | - 38 ± 18 min/day - 4.3% of day | - 3 ± 5 min/day - 0.4 % of day | **In bouts of at least 10 min:**   - 14 ± 14 min/day; - 96 ± 100 min/week in bouts   **Continuous (not in bouts):**   - 288 ± 143 min/week | 8176 ± 2351 |
| Reed, 2018b | Nurses | 76 | - | - | - | - | 27.4 ± 49.1 min/week | 8885 ± 3596 |
| Schnebly, 2017 | Bedside nurses working 12 h shifts | 37 | - | - | - | - | - | 9369.7 ± 2786.5 steps/12 h work day |
| Soh, 2006 | Anaesthiologists | 30 | - | - | - | - | - | 4770 [1985-8922] steps at work |
| Steeves, 2015 | Health service occupations; Health diagnosing, assessing and treating | Health service occupations 38; Health diagnosing, assessing and treating 42 | **Health service occupations:**   - 50.4% ± 1.6%   **Health diagnosing, assessing and treating:**   - 55.2% ± 1.1% | **Health service occupations:**   - 34.6% ± 0.7%   **Health diagnosing, assessing and treating:**   - 32.5% ± 1.0% | - | - | **Health service occupations:**   - 2.1% ± 0.4%   **Health diagnosing, assessing and treating:**   - 2.7% ± 0.3% | - |
| Steeves, 2018 | Health care practitioner, technical  Health care support | Health care practitioner, technical: 69  Health care support: 49 | **Healthcare practitioner, technical:**   - 56.8% ± 1.1%   **Health care support**:   - 54.2% ± 2.1% | - | - | - | **Health care practitioner, technical:**   - 41.9 ± 12 min/week   **Health care support:**   - 22.2 ± 7 min/week | **Healthcare practitioner, technical:**   - 7126 ± 477   **Health care support:**   - 6574 ± 352 |
| Torquati, 2018 | Nurses | 47 | - 486.3 ± 107.7 min/day - 58.4% ± 8.5% | - 322.4 ± 79.4 min/day - 38.7% ± 8.5% | - | - | - 19.1 [24.6] min/day - 3.0% ±1.9% | 8496 ± 2528 |
| Umukoro, 2013 | Hospital patient care workers (nurses: 81%, personal care assistants: 19%) | 48 | - 1308 ± 485 min at work over 7 days - 54% ± 12% of work time | - 1084 ± 386 min at work over 7 days - 45% ± 11% of work time | - 30 ± 32 min at work over 7 days - 1% ± 1% of work time - 165 ± 95 min/day | - 0.4 ± 1.5 min at work over 7 days - 0% ± 0% of work time - 18 ± 44 min/day | - | - |
| **Laborers** | | | | | | | | |
| Arias, 2015 | Commercial construction | 55 | - 760 ± 677 min/day - 37% ± 18% of day | - 1055 ± 404 min/day - 51% ± 14% | - 243 ± 128 min/day - 12% ± 7% - Outside of work: 130 ± 123 - In 10 min bouts: 40 ± 50 - Outside of work in 10 min bouts: 93 ± 209 | - 2 ± 6 min/day - Outside of work: 5 ± 12 min/day - In 10 min bouts: 3 ± 11 min/day | - | - |
| Balogh, 2004 | Cleaners | 48 | 20% ± 18% of work day | - | - | - | - | - |
| Clays, 2017 | Construction workers, garbage collectors | NR | - | - | - | - | 33% of work | - |
| Dollman, 2016 | Farmers | 29 | 16.5 ± 1.8 h/day including time in bed | - | - | - | - | - |
| Gram, 2016 | Construction workers | 53 | - Work: 2% [0-13%] - Leisure time: 20% [3-50%] | - Work: 88% [2-97%] - Leisure: 72% [47-90%] | - | - | - Work: 2% [0-10%] - Leisure: 0% [0-2%] | - |
| Ju, 2011 | Self-employed Korean American married couples working at dry cleaners | 96 | - | - | - | - | - | **Total:** 8009.9 ± 2784.1  **Husbands:** 8381.8 ± 2594.5  **Wives:** 7312.9 ± 2814 |
| Julin 2011 | Construction | 27 | - | - | - | - | - | - Work time: 9820 (CI 95% 8431–11210) - Total: 15085 (95% CI:13213–16957) |
| Korshoj 2013 | Cleaners | 20 | - | - | - | - | - | 20198 ± 4627 |
| Lunde, 2017 | Construction | 124 | **Sitting at work:** 156.8 ± 114.2 min/day  **Sitting in leisure:** 282.0 ± 78.4 min/day | - | - | - | - | - |
| Pontt, 2015 | Rural farmers | 29 | - 4.5 ± 1.5 h/work time - 8.2 ± 1.6 h/total day | - | - | - | - | 11296 ± 3948 |
| Schulz, 2018 | Farmers | 40 | - | - | - | - | **Off-peak season:**   - 44.8 ± 24.8 min/day   **Peak season:**   - 54.8 ± 25.3 min/day | **-** |
| Schofield, 2005 | Blue collar (mechanics, green keepers, dry cleaners) | 181 | - | - | - | - | - | **Steps/day at work:**   - 10334 ± 5553   **Steps/day outside of work:**   - 4201± 4139   **Total steps/day:**   - 14535 ± 5368 |
| Steeves, 2015 | Farm and nursery workers  Helpers, cleaners, hand packagers, laborers  Construction laborers  Agricultural, forestry, fishing  Cleaning and building service occupations  Construction trades  Freight, stock, and material movers (hand)  Farm operators, managers and supervisors  Other mechanics and repairers  Vehicle and mobile equip. mechanics, repairers | Farm and nursery workers 6;  helpers, cleaners, hand packagers, laborers 18;  construction laborers 9;  agricultural, forestry, fishing 21;  cleaning and building service occupations 23;  construction trades 58; freight, stock, and material movers (hand) 14;  farm operators, managers and supervisors 5;  other mechanics and repairers 37; vehicle and mobile equip. mechanics, repairers 15 | **Farm and nursery workers:** 44.7% ± 5.4%  **Helpers, cleaners, hand packagers, laborers**: 42.4% ± 1.2%  **Construction laborers:** 45.3% ± 2%  **Agricultural, forestry, fishing:** 45.4% ± 1.3%  **Cleaning and building service occupations:** 45.6% ± 2.2%  **Construction trades:** 45.7% ± 1.3%  **Freight, stock, and material movers (hand):** 46.1% ± 2.7%  **Farm operators, managers and supervisors:** 46.3 %± 1.0%  **Other mechanics and repairers:** 49.5% ± 1.2%  **Vehicle and mobile equip. mechanics, repairers:** 49.8% ± 2.9% | **Farm and nursery workers:** 32.5% ± 2.1%  **Helpers, cleaners, hand packagers, laborers:** 36.1% ± 1.8%  **Construction laborers:** 30.5% ± 1.6%  **Agricultural, forestry, fishing:** 29.9% ± 1.7%  **Cleaning and building service occupations:** 31.3% ± 0.6%  **Construction trades:** 32.3% ± 0.7%  **Freight, stock, and material movers (hand):** 32.3% ± 1.4%  **Farm operators, managers and supervisors:** 32.1% ± 1.0%  **Other mechanics and repairers:** 32.1% ± 1.0%  **Vehicle and mobile equip. Mechanics, repairers:** 33.3% ± 1.4% | - | - | **Farm and nursery workers:** 5.7% ± 1.3%  **Helpers, cleaners, hand packagers, laborers:** 4.8% ± 0.8%  **Construction laborers:** 7.4% ± 1.0%  **Agricultural, forestry, fishing:** 7.7% ± 1.0%  **Cleaning and building service occupations:** 5.1% ± 0.4%  **Construction trades:** 5.6% ± 0.4%  **Freight, stock, and material movers (hand):** 4.4% ± 0.6%  **Farm operators, managers and supervisors:** 5.6% ± 0.8%  **Other mechanics and repairers:** 4.0% ± 0.5%  **Vehicle and mobile equip. Mechanics, repairers:** 2.9% ± 0.7% | - |
| Steeves, 2018 | Farming, fishing, forestry  Building and grounds cleaning, maintenance  Construction, extraction | Farming, fishing, forestry 6  Building and grounds cleaning, maintenance 43  Construction, extraction 127 | **Farming, fishing, forestry:** 45.2% ± 1.5%  **Building and grounds cleaning, maintenance:** 45.4% ± 2.0%  **Construction, extraction:** 46.8% ± 1.1% | **-** | - | - | **Farming, fishing, forestry:** 149.6 ± 51min/week  **Building and grounds cleaning, maintenance:** 97.8 ± 21min/week  **Construction, extraction:** 63.6 ± 11min/week | **Farming, fishing, forestry:** 11602 ± 521  **Building and grounds cleaning, maintenance:**10464 ± 983  **Construction, extraction:** 9453 ± 469 |
| **Office workers (traditional desks)** | | | | | | | | |
| Alkhajah, 2012 | Office workers | I: 18, C: 14 | **Minutes/8-h work day**   - I: 329 ± 55 - C: 377 ± 56   **Minutes/16-h day**   - I: 551 ± 75 - C: 607 ± 82 | - | - | - | - | - |
| Arundell, 2018 | Office workers | 140 (ABW: 79, C: 61) | - ABW: 387.5 ± 27.5 - Comparison: 379.8 ± 29.5 | - ABW: 74.3 ±22.5 - Comparison: 77.9 ± 24.7 | - | - | - ABW: 18.2 ± 8.4 - Comparison: 22.2 ± 10.8 | **-** |
| Badland, 2004 | Office workers | 56 |  | - | - | - | - | **Work day:**   - Men: 4704 ± 1331 - Women: 4986 ± 1499   **Non-work:**   - Men: 4166 ± 2531 - Women: 4177 ± 2305   **Total:**   - Men: 8870 ± 3065 - women: 9163 ± 2789 |
| Balogh, 2004 | Office workers | 41 | 70% ± 13% of work day | - | - | - | - | - |
| Bergman, 2018 | Office workers | 80 (I: 40, C: 40) | - I: 577 (95% CI: 545-610) - C: 540 (95% CI: 508-572) | - I: 340 (95% CI: 319-361) - C: 345 (95% CI: 324-365) | - | - | - I: 59 (95% CI: 52-66) - C: 50 (95% CI: 43-57) | - I: 9183 (95% CI: 8270-10097) - C: 8799 (95% CI: 7898-9700) |
| Brakenridge, 2016 | Office workers | 153 | - 446.0 ± 58.2 min/10-h workday - 622.1 ± 68.9/16-h day | - | - | - | - | - 2286.8 ± 774.3 steps/10 h workday - 5004.0 ± 1431.3 steps/16 h day |
| Brett, 2017 | Administrative workers | 10 | - | - | - | - | - | - Administrative/Support: 10554.76 ± 3818.15 |
| Brown, 2013 | Office workers | 108 | **Total sample (hours/day, % of day):**   - Total: 8.6 [1.6], 57.4% - During work: 4.3 [1.0] - Before and after work: 3.6 [1.2] - Non-workday: 7.2 [1.7]   **Men (hours/day, % of day):**   - Total: 9.4 [1.4] - During work: 4.6 [1.0] - Before and after work: 4.2 [0.9] - Non-workday: 7.9 [2.0]   **Women (hours/day, % of day):**   - Total: 8.3 [1.4] - During work: 4.3 [0.9] - Before and after work: 3.4 [1.0] - Non-workday: 7.1 [1.5] | **Total sample (hours/day, % of day):**   - Total: 5.7 [1.3]; 37.5% - During work: 2.1 [1.0] - Before and after work: 2.6 [1.2] - Non-workday: 5.8 [2.0]   **Men (hours/day, % of day):**   - Total: 5.4 [1.4] - During work: 1.7 [1.0] - Before and after work: 2.7 [0.9] - Non-workday: 5.6 [2.0]   **Women** **(hours/day, % of day):**   - Total: 5.8 [1.4] - During work: 2.3 [0.9] - Before and after work: 2.65 [1.0] - Non-workday: 5.9 [1.5] | - | - | **Total sample (mins/day, % of day):**   - Total: 39.1 [31.0]; 5.1% - During work: 7.0 [8.95] - Before and after work: 22.0 [27.4] - Non-workday: 35.0 [35.6]   **Men (mins/day, % of day):**   - Total: 39.2 [32.2] - During work: 7.5 [10.9] - Before and after work: 20.0 [24.9] - Non-workday: 40.3 (50.1)   **Women (mins/day, % of day):**   - Total: 38.7 [31.4] - During work: 7.0 [8.47] - Before and after work: 24.2 [27.5]   Non-workday: 34.0 [32.3] | - |
| Carr, 2016 | Office workers | 38 | 7.3 [6.4-8.1] h/day | - | - | - | - | 4407 [2950-5589] |
| Chae, 2015 | Office workers | 70 | - | - | - | - | - | 5403.7 ± 1799.1 |
| Chastin, 2009 | Postal workers (office-based) | 39 | - | - | - | - | - | 11007 ± 4000 |
| Chau, 2014 | Office workers | 32 | 347 ± 59 min/day | - | - | - | - | - |
| Clark, 2011 | Office workers | 82 | 6.74 ± 0.97 h/work day | - | - | - | - | - |
| Clemes, 2014a | Office workers | 72 | - | - | - | - | - | At work: 3742 ± 2493  Outside work: 5159 ± 2474 |
| Clemes, 2014b | Office workers | 170 | **Workdays**   - 580 [101] min/day - 68% ± 9% of work day   **Non-workdays**   - 460 [105] min/day - 60% ± 14% of day | **Workdays**   - 246 (90) min/day - 28% (14%)   **Non-workdays**   - 278 (126) min/day - 36% (14%) | - | - | **Workdays**   - 32 (26) min/day; 4% (3%)   **Non-workdays**   - 28 (33) min/day; 4% (4%) | - |
| Cole, 2015 | Desk-based workers (software engineers) | 5 | 401.9 [377.30-421.90] min/day | - | - | - | 36.47 [27.39-48.08] min/day | 3134.46 [2430.8-3887.3] |
| De Jong, 2018 | Office workers | 21 | **Non-work days:**   - 9.8 h ± 2.0h - 66.6% ±14.2%   **Work-days:**   - 10.6 h ± 2.3h - 68.4% ± 13.5% | **Work days:** 1.1 h/day | - | - | - | **Non-work days:**   - 6409 ± 2843   **Work-days:**   - 7125 ± 2554 |
| Dollman, 2016 | Office workers | 28 | 17.9 ± 1.4 h/day (including bed) | - | - | - | - | - |
| Evans, 2012 | Office workers | I: 14, C: 14 | - I: 5.9 ± 1.0 h/day at work (78.1% ± 6.9% of h/day) - C: 5.6 ± 1.0 h/day at work (74.0% ± 11.0% of h/day) | - | - | - | - | - |
| Finkelstein, 2015 | Desk-based office workers | 800 | Cash: 803.9 ± 84.9 min/day  Charity: 812.1 ± 98.2 min/day  Control: 815.8 ± 94.7 min/day  Fitbit only: 797.2 ± 73.3 min/day | - | Cash: 43.7 ± 19.0 min/day  Charity: 43.1 ± 18.9 min/day  Control: 43.3 ± 19.3 min/day  Fitbit only: 44.3 ± 20.1 min/day | Cash: 3.5 ± 7.07 min/day  Charity: 2.4 ± 4.1 min/day  Control: 2.8 ± 4.5 min/day  Fitbit only: 3.4 ± 6.6 min/day | - | Cash: 8298 ± 2500  Charity: 7777 ± 2256  Control: 8032 ± 2501  Fitbit only: 7999 ± 2410 |
| Fisher, 2018 | Office workers | 131 | At work: 5.46 h ±1.62h | - | - | - | - | At work: 3412 ± 1919 |
| Foley, 2016 | Office workers | 56 | 80.28% | 9.08% | - | - | 10.68% | 449.43 steps/h at work |
| Gao, 2016 | Office workers with traditional sit workstations | 10 | 80.9% ± 6.4% | 14.9% ± 6.3% | - | - | 4.2% ± 2.1% | - |
| Gilson, 2012 | University office workers | 330 | - | - | - | - | - | 5892 ± 2016 |
| Gilson, 2010 | Office workers | 47 | - | - | - | - | - | - I: 7919 ± 2117 - C: 7473 ± 2342 |
| Gilson, 2012 | Office workers | 11 | 75.8% ± 10.3% | 15% ± 4.9% | - | - | 9.2% ± 6.8% | - |
| Gilson, 2016 | Office workers (administrative and clerical workers) | 57 | **Sedentary:**   - 9.7 ± 2 h/day - I: 68% ± 14% - C: 74% ± 7%   **Sitting time (sit pad):**   - 371 ± 71 min/day - I: 370 ± 84 min/day - C: 372 ± 53 min/day | - 5 ± 1.7 h/day - I: 25% ± 11% - C: 19% ± 7% | - | - | - 1.6 ± 0.7 h/day - I: 7 % ± 4% - C: 7% ± 3% | - |
| Gorman, 2013 | Office workers | 27 (faculty (n=4), staff (n=7), and graduate students (n=13) | 364.1 ± 43 min/8 h workday | - | - | - | - | - |
| Hadgraft, 2016 | Office workers (managers, professional, clerical, sales & services) | 229 | **Workplace:** 78.8% ± 9.5%  **Workdays:** 69.4 ± 8 min/day  **Non-workdays:** 55.9 ± 13 min/day  **Overall:** 64.6 ± 8.4 min/day | - | - | - | 23 ± 17 min/day | - |
| Hallman, 2018 | Office workers | I=79, C=31 | **Sitting time at work:**   - I = 70.6% ± 15% - C = 65.8% ± 18.5% | - | - | - | - | - |
| Healy, 2013 | Office workers | I=18, C=18 | **Work time:**   - I: 338.5 ± 35.3 min/day - C: 334.7 ± 52.4 min/day | - | - | - | - I: 19.7 ± 15.4 - C:13.5 ± 19   (MET Minutes/8hr day) | - I: 1997 ± 678 steps at work - C: 1924 ± 549 steps at work |
| Healy, 2016 | Office workers (managers, administrators, professionals, clerical/sales/service) | 229 | **Work:**   - 378.3 ± 45.6 min/day - 78.8% ± 9.5%   **All day:**   - 620.6 ± 80.7 min /day - 64.6% ± 8.4% | - | - | - | - | **-** |
| Hogstedt, 2017 | Office workers | I: 173, C: 144 | - 340 ± 57 min/8 h workday - 290 ± 51 min/8 h leisure | - | - | - | 45 ± 21 min/8 h leisure | **Steps/hour at work**   - 464 ± 161   **Steps per hour in leisure**   - 706 ± 297 |
| Jancey, 2016 | Office workers | 42 | - 401.30 ± 39.45 min/day - 84.88% ± 4.71% | - 35.13 ± 12.33 min/day - 11.20% ± 3.68% | - 36.13 ± 13.53 min/day - 3.85% ± 1.80% | - 0.33 ± 0.63 min/day - 0.07% ± 0.13% | - | 3238.17 ± 1310.23 |
| Kirk, 2016 | Office workers | 29 | - Weekday sitting time: 8.9 h/day (62%) - Weekend day sitting: 8.2 h/day (58%) | - | - | - | - | - Weekday: 9508 - Weekend day: 8159 |
| Kloster, 2017 | Office workers | 317 | - Workday sitting time: 347 [312-378] min/8h | - | - | - | - | - |
| Koepp, 2013 | Office workers | 36 | 1020 ± 66 min/day | - | - | - | - | - |
| Kozey-Keadle, 2012 | Office workers | 20 | - 67.0% ± 13.3% of day (ActivPAL all days) - 69.4% ± 11.1% (ActivPAL Weekday) - 61.0% ± 16.3% (ActivPAL Weekend) - 68.8% ± 8.5 (ActivPAL Weekday) - 66.4% ± 10.2% (ActiGraph Weekday (100 counts/min)) - 70.5 ± 9.4 (ActiGraph Weekday (150 cpm)) - 60.4 ± 15.6 (ActivPAL Weekend) - 62.7 ± 8.9 (ActiGraph Weekend (100 cpm)) - 66.7 ± 9.0 (ActiGraph Weekend (150 cpm)) | - | - | - | - 16.1 min/day | - All days: 6417 ± 3366 - Weekday: 6121 ± 2495 - Weekend: 7132 ± 4871 |
| Lagersted-Olsen, 2014 | Employees with mainly administrative work | 26 | - 5.6 ± 1.1 h/day - 73.1% of workday | - | - | - | - | - |
| Larouche, 2018 | Office workers (executive, professional, clerical) | 19 | **Minutes / 8-h work day**   - 267.9 ± 68 | **Minutes / 8-h work day**   - 35.9 ± 15.5 | - | - | **Minutes / 8-h work day**   - 6 ± 2.8 | **-** |
| Li, 2017 | Office workers | 26 | **Minutes sitting/day (95% CI):**   - Group 1: 362 (320,403) - Group 2: 355 (271,440) - Group 3: 356 (243,469) - Group 4: 410 (355,465)   **% of wear time sitting (95% CI):**   - Group 1: 69 (62,76) - Group 2: 64 (47,81) - Group 3: 62 (43,81) - Group 4: 75 (72,79) | - | - | - | - | **Steps/day mean (95% CI):**   - Group 1: 5753 (3863, 7643) - Group 2: 4855 (2416, 7293) - Group 3: 4547 (2843, 6252) - Group 4: 3741 (2619, 4864) |
| Mansoubi, 2016 | Office workers | 40 | **ActivPAL sitting:**   - Work time on workdays: 76% ± 13%; 299 ± 85 min/day - Non-working on workdays: 60 ± 11; 307 ± 82 min/day   **ActiGraph sedentary:**   - Working on workdays: 82% ± 5%; 333 ± 40 min/day - Non-working on workdays: 70% ± 7%; 316 ± 42 min/day | **ActiGraph:**   - Working on workdays: 14% ± 4%, 53 ± 18 min/day - Non-working on workdays: 21% ± 5%, 96 ± 29 min/day | - | - | **ActiGraph:**   - Working time on workdays: 4% ± 1%; 16 ± 8 min/day - Nonworking hours on workdays: 9% ± 5%; 32 ± 19 min/day | - |
| Miller, 2004 | Professional/ managerial; administrative | Professional/ managerial: 43, administrative: 42 | **Weekday sitting at work:**   - Professional/managerial: 6.2 ± 1.6 h/day - Administrative: 5.7 ± 1.3 h/day   **Work time spent sitting:**   - Professional/managerial: 75.0% ± 19.0% - Administrative: 75.7% ± 16.8%   **Total weekday sitting:**   - Professional/managerial: 10.6 ± 2.3 h/day - Administrative: 10.3 ± 2.1 h/day | - | - | - | - | **Weekday:**   - Professional/managerial: 7883 ± 2427 - Administrative: 7207 ± 2666 - Scientists: 10147 ± 2868   **Weekend day:**   - Professional/managerial: 8599 ± 2287 - Administrative: 7544 ± 3600 |
| Mitsui, 2010 | Office workers | 50 | - | - | - | - | - | **Summer:**   - Workdays: 6560 ± 2600 - Holidays: 7016 ± 4679   **Winter:**   - Workdays: 5236 ± 2253 - Holidays: 4770 ± 3039 |
| Neuhaus, 2014 | Desk-based office workers | I1=16, I2=14, C=14 | - **Total:** 368 ± 46 min/8h workday - **Multi-component:** 366 ± 49 min/8h workday - **Workstation-only:** 373 ± 36 min/8h - **Comparison:** 365 ± 54 min/8r day - **Overall:** 77% ± 10% of time at workplace | - | - | - | - | **Steps/8-hr workday:**   - **Multi-component:** 1548 ± 525 - **Workstation-only:** 1920 ± 568 - **Comparison:** 1789 ± 1015 - **Overall:** 1742 ± 786 |
| Nooijen, 2018 | Office workers | 311 | **Workplace:**   - 5.5 ± 1.4 h - 64% ±15%   **Working days:**   - 10.2 ± 2.5 h - 61% ±15%   **Non-working days:**   - 8.8 ± 2.6 h - 57% ± 17%   **Overall:**   - 9.7 ± 1.4 h - 60% ± 8% | - | - | - | - | - |
| Olsen, 2018 | Office workers (financial services) | 24 | 673.8 min/day | 137.8 min/day | - | - | 36.7 min/day | - |
| Parry, 2013 a | Office workers | 50 | - Work day: 76% of wear time - Non-workday: 70% of wear time - Work time on work day: 82% of wear time - Non-work time on work day: 67% of wear time - Total non-work: 69% of wear time - 11.3 h/day on workdays - 676.0 ± 58.7 min/day on workdays - 9.3 h on non-work days - 570.5 ± 88.0 min/day on non-work days | - Work day: 20% of wear time - Non-workday: 27% of wear time - Work hours on work day: 15% of wear time - Non-work hours on work day: 26% of wear time - Total non-work: 27% of wear time - Work day: 176.9 ± 52.6 min/day - Non-work day: 224.4 ± 78.3 min/day | - | - | - Work day: 4% of wear time - Non-workday: 3% of wear time - Work hours on work day: 3% of wear time - Non-work hours on work day: 7% of wear time - Total non-work: 4% of wear time - Work days: 39.5 ± 18.7 min/day - Non-work day: 25.7 ± 25.7 min/day | - |
| Parry, 2013 b | Office (clerical, call centre, and data processing workers) | 62 | - Workdays = 72.85% ± 7.06% - Work hours = 78.29% ± 8.41% | Workdays = 23.85% ± 6.37%   - Work hours = 19.14% ± 7.75% | - | - | - Workdays = 3.29% ± 1.83% - Work hours = 2.57% ± 1.83% | - |
| Peavler, 2012 | Employees working at desk dependent jobs | I: 23, C=17 | - C: 544.2 ± 76.9 min/day - I: 584.9 ± 136.1 min/day - C: 65.7% ± 7.5% - I: 67.6% ± 7.2% | - C: 265.7 ± 84.0 min/day - I: 263.9 ± 69.5 min/day - C: 31.9% ± 8.1% - I: 30.6% ± 8.2% | - C: 18.6 ± 25.2 min/day - I: 14.5 ± 18.5 min/day - C: 2.3% ± 3.2% - I: 1.5% ± 1.5% | - C: 1.2 ± 2.6 min/day - I: 2.7 ± 6.4 min/day - C: 0.14% ± 0.32% - I: 0.27% ± 0.60% | - | - |
| Pedersen, 2016 | Government desk-based workers | 34 | Sitting: 65.7% ± 21.8% |  |  |  |  |  |
| Pontt, 2015 | Rural office workers and rural farmers | 58 | - 6.4 ± 1.4 h/work time - 10.0 ± 1.6 h/day | - | - | - | - | 8886 ± 3077 |
| Rafferty, 2016 | Office workers | 26 | - | - | - | - | **Total:** 32.7 ± 17.1 min/day  **Work:**3.5 ± 3.8 min/day | **Total:** 11008 ± 2999  **Work:** 3564 ± 1599 |
| Ryan, 2011 | Office workers | 83 | - Overall sitting time at work: 66% ± 12% - Workday sitting: 5.3 ± 1.0 h/day | - | - | - | - | - |
| Ryde, 2013 | Office-based employees | 105 | 5.8 ± 1.2 h/workday | - | - | - | - Total: 44.3 ± 25.3 min/day - At work: 16.8 ± 13.2 min/day | - |
| Sawyer, 2017 | Office-based workers (managerial, professional, and administrative) | 115 | Sitting: 0.7 ± 0.14 h/day | - | - | - | - | 433 ± 186 steps/working hour |
| Schofield, 2005 | Office workers | 63 | - | - | - | - | - | **Steps/day at work:**   - 5380 ± 2730   **Steps/day outside of work:**   - 3820 ± 2384   **Total steps/day:**   - 9200 ± 3970 |
| Schwartz, 2016 | Office workers | 97 | 50.1% ± 6.2% | - | - | - | 11.5% ± 4.6% | - |
| Smith, 2015 | Office workers (15% managerial role, 45% professional role) | 164 | - Daily: 10.6 ± NR h/day - Weekday: 10.6 ± 2.1 h/day - Weekend day: 10.6 ± 2.5 h/day | - | - | - | - | - Daily: 9737 ± 3517 - Weekday: 9682 ± 3872 - Weekend day: 9518 ± 4615 |
| Smith, 2002 | Civil servants  Administration  Managers  Secretaries  Accounting  Software programmers | Civil servants: 8 admin: 13, Managers: 9, Secretaries: 9, Accounting: 7, Software programmers: 11 | - | - | - | - | - | **Civil servants:** 8131 ± 1818  **Administration:** 7844 ± 3318  **Managers:** 6810 ± 2430  **Secretaries:** 6457 ± 1939  **Accounting:** 5991 ± 2303  **Software programmers:** 4768 ± 1109 |
| Steeves, 2015 | Information clerks  Executive, administrators, and managers  Miscellaneous administrative support occupations  Records processing occupations  Secretaries, stenographers, and typists | Information clerks: 18  Executive, administrators, managers: 112  Miscellaneous admin support occupations: 65  Records processing: 33  Secretaries, stenographers, typists: 21 | **Information clerks:** 52.5% ± 1.8%  **Executive, administrators, and managers:** 58.6% ± 1.1%;  **Miscellaneous administrative support occupations:** 57.5% ± 1.4%  **Records processing occupations:** 58.8% ± 1.6%  **Secretaries, stenographers, and typists:** 58.7% ± 2.3% | **Information clerks:** 36.1% ± 1.8%  **Executive, administrators, and managers:** 27.8% ± 0.8%  **Miscellaneous administrative support occupations**: 29.7% ± 0.9%  **Records processing occupations:** 30.2% ± 1.3%  **Secretaries, stenographers, and typists:** 30.5% ± 1.9% | - | - | **Information clerks:** 1.9% ± 0.6%  **Executive, administrators, and managers:** 3.2% ± 0.2%  **Miscellaneous administrative support occupations:** 3.0% ± 0.3%  **Records processing occupations:** 1.8% ± 0.2%  **Secretaries, stenographers, and typists:** 1.7% ± 0.4% | - |
| Steeves, 2018 | Management  Office, administrative support | Management: 120  Office, administrative support: 218 | **Management**: 59.6% ± 1.5%  **Office, administrative support:** 58.0% ± 0.8% | - | - | - | **Management**: 40.7 ± 8 min/week  **Office, administrative support:** 39.3 ± 5 min/week | **Management:** 7187 ± 571  **Office, administrative support:** 6797± 238 |
| Stephens, 2018 | Desk-based workers | 121 | 381 ± 49 min/8-h day | - | - | - | - | - |
| Sudholz, 2018 | Sitting-based occupations | 59 | **activPAL3:**   - 346.8 [284.5, 414.3] min/day   **ActiGraph:**   - 430.9 [395.1, 451.4] min/day | - | - | - | - | - |
| Swartz, 2014 | Office workers (clerical positions) | 60 | 376.5 ± 7.9 min/workday | - | - | - | - | 3574 ± 213 steps/work day |
| Taylor, 2016 | Office workers | 175 | **Usual breaks:**   - 3069 (95% CI: 1801, 4338) min/weekday - 950 (95% CI: 426,1473) min/weekend day   **Computer prompts:**   - 2917 (95% CI: 2193, 3641) min/weekday - 602 (95% CI: 464, 740) min/weekend day   **Booster breaks:**   - 3226 (95% CI: 2336, 4117) min/weekday - 794 (95% CI: 620, 969) min/weekend day | - | - | - | - | **Usual breaks:**   - 43558 (95% CI: 37693, 49424) steps/week   **Computer prompts:**   - 46860 (95% CI: 41311, 52408) steps/week   **Booster breaks:**   - 45633 (95% CI: 39335, 51932) steps/week |
| Thorp, 2012 | Office | 131 | 68.3% ± 7.2% of workday | 6.5% ± 3.0% of workday | - | - | 3.5% ± 2.2% of workday | - |
| Tigbe, 2011 | Office postal workers | 112 | 17.6 ± 1.2 h/day | - | - | - | - | 10853 ± 2919 |
| Tobin, 2016 | Office workers | I=18, C=19 | - Sitting: 392 [79] min/day - 77% of working day | - | - | - | - | 2453 ± 1026 |
| Urda, 2016 | Office workers (university staff and administrators) | I: 22, C=22 | - C: 6.02 ± 0.66 h/workday - I: 5.54 ± 1.47 h/workday - Total: 68% of workday | - | - | - | - | - |
| Waters, 2016 | Office workers (non-academic University workers) | 37 | **Workdays:**   - 667.0 [174.5] min/day - 76.9%   **Non-workdays:**   - 557.0 [158.0] min/day - 69.5% | **Workdays:**   - 177.0 [79.0] min/day - 19.7%   **Non-workdays:**   - 214.0 [107] min/day - 26.4% | **Workdays:**   - 26.0 [29.5] min/day - 2.9%   **Non-workdays:**   - 19.0 [33.0] min/day - 2.2% | - | **Workdays:**   - 27.0 [31.0] min/day - 3.0%   **Non-workdays:**   - 21.0 [39.0] min/day - 2.8% | **Workdays:**   - 7494 [4415.5]   **Non-workdays:**   - 7427 [5428] |
| Wieters, 2009 | Office workers | 475 | - | - | - | - | - | Urban: 4932 ± 2494  Suburban: 4348 ± 2398 |
| Ying, 2014 | Office workers | 26 | 80.67% ± 5.42% | - | - | - | - | 4708 ± 1966 steps/3 days |
| Zhu, 2018 | Office workers | 36 | Intervention: 337.1 ± 58.3 min/8-h workday  Control: 314.2 ± 102.1 min/8-h workday | Intervention: 24.9 ± 13.6 min/8-h workday  Control: 30.8 ± 12.0 min/8-h workday | - | - | Intervention: 6.6 ± 4.9 min/8-h workday  Control: 6.8 ± 4.6 min/8-h workday | - |
| **Office workers with sit-stand desks** | | | | | | | | |
| Carr, 2016 | Office workers with access to sit-stand desks | 31 | 6.2 [5.1-7.6] h/day | - | - | - | - | 4589 [3598-5498] |
| Danquah, 2017 | office workers with access to a sit-stand desk | 317 | **Workday sitting time:** 340 ± 57 min/8 h workday (total sample)  **Leisure sitting time**:   - I: 291 (SE= 4.1) min/8 h leisure; - C: 289 (SE=4.2) min/8 h leisure | - | - | - | **Min/8 h leisure:**   - I: 45 ± 1.7 - C: 44 ± 1.7 | - |
| Donath, 2015 | office workers with access to a sit-stand desk | C=16, I=15 | **Sitting:**   - I: 29.4 ± 6.5 h/week - C: 27.7 ± 9.5 h/week | - | - | - | - | - |
| Gao, 2016 | office workers with sit-stand workstations | 14 | 66.2% ± 17.1% | 26.1% ± 12.3% | - | - | 7.7% ± 7.4% | - |
| Hogstedt, 2017 | office workers with sit-stand desks  *Note same study as Danquah 2017 | 317 | **Min/8h workday:**   - I: 345 ± 54 - C: 335 ± 59 - All: 340 ± 57   **Min/8h leisure:**   - I: 291 ± 53 - C: 289 ± 49 - All: 290 ± 51 | - | - | - | **Min/8 hour leisure**   - I = 45 ± 22 - C = 44 ± 19 - All = 45 ± 21 | **steps/h work**   - I = 482 ± 172 - C: 442 ± 144 - All: 464 ± 161   **steps/h leisure**   - I = 709 ± 312 - C = 702 ± 278 - All = 706 ± 297 |
| Wick, 2016 | Office workers with access to sit-stand desks | 38 | **Sitting:** 77.1% ± 11.10% | - | - | - | - | - |
| **Postal delivery** | | | | | | | | |
| Chastin, 2009 | Postal workers delivery | 39 | - | - | - | - | - | 17065 ± 4000 |
| Smith, 2002 | Postmen | 7 | - | - | - | - | - | 12713 ± 1463 |
| Tigbe, 2011 | Delivery postal workers | 56 | 16.0 ± 3.9 h/day | - | - | - | - | 18554 ± 4706 |
| **Protective services** | | | | | | | | |
| Aandstad, 2016 | Home guard soldiers | 411 | - | - | - weekdays 153 [105-239] - weekend days 140 [93-209] | - weekdays 17 [8-33] - weekend days 15 [5-29] | - | - weekdays 10448 (95% CI: 10037-10859) - weekend days 9209 (95% CI: 8780-9638) |
| Chappel, 2016 | Firefighters | 34 | 39.8 ± 32.2 min/whole shift | - 430.1 ± 111.6 min/whole shift - 69% | 152.6 ± 67.2 min/whole shift | 0.2 ± 0.5 min/whole shift | - | - |
| Choi, 2010 | Navy submarine crew | 109 | - | - | - | - | - | - **Deployed:** 2211 ± 121 - **Stationed:** 8181 ± 269 - **Command personnel:** 7773 ± 2198 |
| Cuddy, 2015 | Firefighters | 15 | 49% ± 8% | 39% ± 6% | - | - | 12% ± 2% | - |
| Steeves, 2015 | Protective service occupations | 13 | 59.7% ± 2.4% | 26.5% ± 1.8% | - | - | 3.4% ± 1.0% | - |
| Steeves, 2018 | Protective service | 43 | 60.3% ± 1.5% | - | - | - | 51.9± 11 min/week | 7109 ± 521 |
| Talbot, 2011 | National Guard Personnel (part-time) | 94 | - | - | - | - | - | - I: 6415 ± 2858 - C: 7300 ± 4064 |
| Vincent, 2016 | Firefighters | 40 | - 78 ± 79 min/shift - 11.3% ± 10.5% | - 453 ± 150 min/shift - 65.6% ± 12.7% | - 156 ± 99 min/shift - 23.0% ± 13.9% | - 1 ± 5 min/shift - 0.1% ± 0.7% | - | - |
| **School teachers** | | | | | | | | |
| Cheung, 2008 | School teachers | I: 38, C: 14 | - | - | - | - | - | - Total: 11399 ± 2450 - Work time: 7223 ± 2179 |
| Cheung, 2012 | School teachers (primary school) | 71 | - | - | - | - | - | **Total**   - At work: 7394 ± 1963 - Whole day: 11351 ± 2138   **PE teachers:**   - At work: 8858 ± 1294 - Whole day: 12720 ± 2138   **Non-PE teachers:**   - At work: 6897 ± 1909 - Whole day: 10886 ± 1943 |
| Smith, 2002 | Teachers | 18 | - | - | - | - | - | 7285 ± 1996 |
| Steeves, 2015 | Teachers | 40 | 57.5% ± 1.7% | 29.9% ± 1.2% | - | - | 3.0% ± 0.5% | - |
| **Other workers*** | | | | | | | | |
| Bird, 2015 | Academic university staff (mix of academics and professional staff) | 10 | 68.1% [57.8-83.5%] of workday | 10.4% [8.5-26.8%] of workday | 8.0 [6.7-18.7%] of workday | 0.7 [0.1-2.1%] of workday | - | - |
| Miller, 2004 | Technicians; blue-collar workers | 185 | **Weekday sitting h/day at work:**   - Technicians: 3.3 ± 1.5 - Blue-collar workers: 1.6 ± 1.9   **Work time spent sitting (%):**   - Technicians: 43.0 ± 19.6 - Blue-collar workers: 22.0 ± 20.8   **Total weekday sitting h/day:**   - Technicians: 7.8 ± 2.3 - Blue-collar workers: 6.5 ± 1.8 | - | - | - | - | **Weekday:**   - Technicians: 10731 2330 - Blue-collar workers: 11784 ± 4183   **Weekend day:**   - Technicians: 8432 ± 3665 - Blue-collar workers: 9246 ± 2383 |
| Schofield, 2005 | Allied university | 48 | - | - | - | - | - | **Steps/day at work:**   - 4790 ± 2038   **Steps/day outside of work:**   - 4708 ± 2278   **Total steps/day:**   - 4988 ± 3127 |
| Smith, 2002 | Technicians, Professional engineers, Housewives | Technicians: 12, Professional engineers: 16, Housewives 14 | - | - | - | - | - | **Technicians:** 8866 ± 2752  **Professional engineers:** 7947 ± 2484  **Housewives:** 5674 ± 2894 |
| Steeves, 2015 | Supervisors and proprietors, sales occupations  Private household occupations  Material recording, scheduling, distributing clerks  Sales reps., finance, business, & commodities  Technicians and related support occupations  Writers, artists, entertainers, and athletes  Personal service occupations;  Management related occupations  Engineers, architects and scientists  Other professional specialty occupations | Supervisors and proprietors, sales occupations 27;  Private household occupations 9;  Material recording, scheduling, distributing clerks 19;  Sales reps., finance, business, & commodities 29;  Technicians and related support occupations 33;  Writers, artists, entertainers, and athletes 15;  Personal service occupations 14;  Management related occupations 49;  Engineers, architects and scientists 29;  Other professional specialty occupations 39 | **Supervisors and proprietors, sales occupations:** 49% ± 3.0%  **Private household occupations:** 51.7% ± 9.1%  **Material recording, scheduling, distributing clerks:** 52.3% ± 4.4%  **Sales reps., finance, business, & commodities:** 58.2% ± 1.9%  **Technicians and related support occupations:** 58.5% ± 2.5%  **Writers, artists, entertainers, and athletes:** 58.9% ± 2.6%  **Personal service occupations:** 54.5% ± 2.5%  **Management related occupations:** 60.3% ± 1.2%  **Engineers, architects and scientists**: 65% ± 1.4%  **Other professional specialty occupations:** 59.9% ± 1.8% | **Supervisors and proprietors, sales occupations:** 33.1% ± 1.6%  **Private household occupations:** 31.2% ± 4.9%  **Material recording, scheduling, distributing clerks:** 30.2% ± 0.9%  **Sales reps., finance, business, & commodities**: 27.7% ± 1.3%  **Technicians and related support occupations:** 28.6% ± 1.8%  **Writers, artists, entertainers, and athletes:** 27.5% ± 1.4%  **Personal service occupations:** 33.7% ± 2.0%  **Management related occupations:** 26.4% ± 0.8%  **Engineers, architects and scientists:** 23% ± 0.8%  **Other professional specialty occupations:** 26.9% ± 1.1% | - | - | **Supervisors and proprietors, sales occupations:** 3.1% ± 0.5%  **Private household occupations:** 4.3% ± 1.5%  **Material recording, scheduling, distributing clerks:** 3.3% ± 1.0%  **Sales reps., finance, business, & commodities**: 3. 5% ± 0.6%  **Technicians and related support occupations:** 3.6% ± 0.4%  **Writers, artists, entertainers, and athletes:** 3.5% ± 0.7%  **Personal service occupations:** 1.6% ± 0.4%  **Management related occupations:** 3.5% ± 0.4%  **Engineers, architects and scientists:** 4.0% ± 0.7%  **Other professional specialty occupations:** 2.9% ± 0.3% | - |
| Steeves, 2018 | Business, financial operations  Computer, mathematical  Legal  Installation, maintenance, repair  Personal care, service  Production  Education, training, library  Sales & related  Architecture, engineering  Arts, design, entertainment, sports, media  Life, physical, social science  Community social services | Business, financial operations: 68  Computer, mathematical: 32  Legal: 12  Installation, maintenance, repair: 62  Personal care, service: 43  Production: 134  Education, training, library: 63  Sales & related: 117  Architecture, engineering: 36  Arts, design, entertainment, sports, media: 19  Life, physical, social science: 19  Community social services: 21 | **Business, financial operations:** 61.1% ± 0.8%  **Computer, mathematical:** 65.9% ± 1.9%  **Legal:** 65.9% ± 1.8%  **Installation, maintenance, repair:** 51.2% ±2.0%  **Personal care, service:** 50.9% ± 2.4%  **Production:** 52.7% ± 1.3%  **Education, training, library:** 55.3% ± 1.5%  **Sales & related:** 55.4% ± 1.6%  **Architecture, engineering:** 63.3% ± 2.0%  **Arts, design, entertainment, sports, media:** 62.4% ± 1.6%  **Life, physical, social science:** 64.8% ± 1.5%  **Community social services:** 64.2% ± 1.6% | - | - | - | **Business, financial operations:** 70.9 ± 13 min/week  **Computer, mathematical:** 72.0 ± 27 min/week  **Legal**: 53.6 ± 33 min/week  **Installation, maintenance, repair:** 37.4 ± 13 min/week  **Personal care, service:** 56.5 ± 24 min/week  **Production:** 24.4 ± 5 min/week  **Education, training, library:** 67.7 ± 15 min/week  **Sales & related:** 42.0 ± 10 min/week  **Architecture, engineering:** 66.5 ± 15 min/week  **Arts, design, entertainment, sports, media**: 66.1 ± 23 min/week  **Life, physical, social science:** 66.4 ± 22 min/week  **Community social services**: 12.1 ± 6 min/week | **Business, financial operations:** 7215 ± 391  **Computer, mathematical:** 6199 ± 411  **Legal**: 5804 ± 1066  **Installation, maintenance, repair:** 9004 ± 857  **Personal care, service:** 8339 ± 753  **Production:** 8151 ± 283  **Education, training, library:** 7890 ± 347  **Sales & related:** 7243 ± 333  **Architecture, engineering:** 7119 ± 462  **Arts, design, entertainment, sports, media:** 6552 ± 423  **Life, physical, social science:** 6196 ± 510  **Community social services**: 5684 ± 516 |
| van Dommelen, 2016 | Financial services | 80 | **Total time:**   - Men: 70.0% ± 5.2 % - Women: 67.4% ± 6.9%   **Occupational time:**   - Men: 78.5%, ± 5.6% - Women: 79.5% ± 5.9% | **Total % of wear time:**   - Men: 26.0% ± 5.2% - Women: 29.2% ± 6.6%   **Occupational time:**   - Men: 17.7% ± 5.2% - Women: 17.6% ± 5.4% | **Total % of wear time:**   - Men: 3.6% ± 1.1% - Women: 3.1% ± 1.2%   **Occupational time:**   - Men: 3.5% ± 2.0% - Women: 2.4% ± 1.4% | **Total % of wear time:**   - Men: 0.3% ± 0.5% - Women: 0.4% ± 0.6%   **Occupational time:**   - Men: 0.3% ± 0.9% - Women: 1.1% ± 1.1% | **Total % of wear time:**   - Men: 4.0% ± 1.3% - Women: 3.4% ± 1.4%   **Occupational time:**   - Men: 3.8% ± 2.2% - Women: 2.8% ± 1.9% | - |
| Weiler, 2015 | Professional football players | 25 | 500.6 ± 59 min/day | 55.7 ± 28.8 min/day | 74.1 ± 28.1 min/day | 2.1 ± 3.9 min/day | - | - |

*Other workers = (included more than one group, insufficient information for grouping, or does not fit categories)

C – Control, cpm – counts per minute, h – hours, I – Intervention, IQR – interquartile range, min – minutes, MVPA – moderate-to-vigorous intensity physical activity, PA – physical activity, PE – physical education, PT – physiotherapist, SD – standard deviation, SE – standard error
