# Supplementary material for: Device-measured physical activity, sedentary behaviour and cardiometabolic health and fitness across occupational groups: a systematic review and meta-analysis
Source: Int J Behav Nutr Phys Act. 2019 Apr 2;16:30. doi: 10.1186/s12966-019-0790-9 (PMC6444868; doi:10.1186/s12966-019-0790-9)

Supplemental Figures

Individual forest plots and funnel plots for sub-groups

Supplemental Figures 1a-dd

Sedentary Time (ST)

Supplemental Figure 1a. Percentage of ST at work across all occupations

Outcome	Study name	Comparison	Mean	Lower limit	Upper limit	Standard error	Subgroup within study
SED % work	Balogh, 2004 - L	Laborers	20.000	17.171	22.829	1.443	Cleaners
SED % work	Balogh, 2004 - O	Office workers	70.000	66.021	73.979	2.030	Blank
SED % work	Brewer, 2016	Healthcare workers	71.874	69.854	73.894	1.031	Combined
SED % work	Gilson 2017	Drivers	23.000	15.806	30.194	3.671	Blank
SED % work	Hadgraft 2016	Office workers	78.800	77.570	80.030	0.628	Blank
Sitting % work	Hallman 2018	Office workers	69.616	66.667	72.565	1.505	Combined
Sitting % work	Healy 2016	Office workers	77.800	76.570	79.030	0.628	Blank
SED % work	Li 2017	Office workers	73.669	71.588	75.750	1.062	Combined
SED % work	Loef 2018	Healthcare workers	51.596	50.337	52.855	0.642	Combined
SED % work	Mansoubi 2016	Office workers	76.000	71.971	80.029	2.055	Blank
SED % work	Mller 2004 - A	Academics	63.200	58.012	68.388	2.647	Scientists
SED % work	Mller 2004 - BC	Blue-collar workers	22.000	9.702	34.298	6.274	Blank
SED % work	Mller 2004 - O	Office workers	75.390	71.610	79.170	1.929	Combined
SED % work	Mller 2004 - tech	Technicians	43.000	36.326	49.674	3.405	Blank
SED % work	Nooijen 2018	Office workers	64.000	62.333	65.667	0.851	Blank
SED % work	Parry 2013 b	Office workers	78.290	76.197	80.383	1.068	Blank
Sitting % work	Ryan 2011	Office workers	66.000	63.418	68.582	1.317	Blank
SED % work	Thorp 2012 - C	Call centre	71.200	68.778	73.622	1.236	Blank
SED % work	Thorp 2012 - CS	Customer service	62.100	59.566	64.634	1.293	Blank
SED % work	Thorp 2012 - O	Office workers	68.300	67.067	69.533	0.629	Blank
SED % work	Umukoro 2013	Healthcare workers	54.000	50.605	57.395	1.732	Nurses and aides
SED % work	van Dommelen 2016 - A	Academics	76.550	74.968	78.132	0.807	Combined
SED % work	van Dommelen 2016 - F	Financial services	78.863	77.612	80.114	0.638	Combined
SED % work	van Dommelen 2016 - L	Laborers	43.600	38.227	48.973	2.742	Men
SED % work	Vincent 2016	Protective services	11.300	8.046	14.554	1.660	Blank
			59.972	54.201	65.743	2.945	

Mean and 95% CI

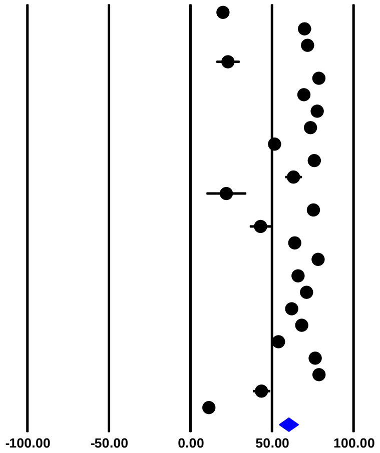

Sedentary time during work time (%)

Supplemental figure 1b. Funnel plot for ST (%) at work across all occupations

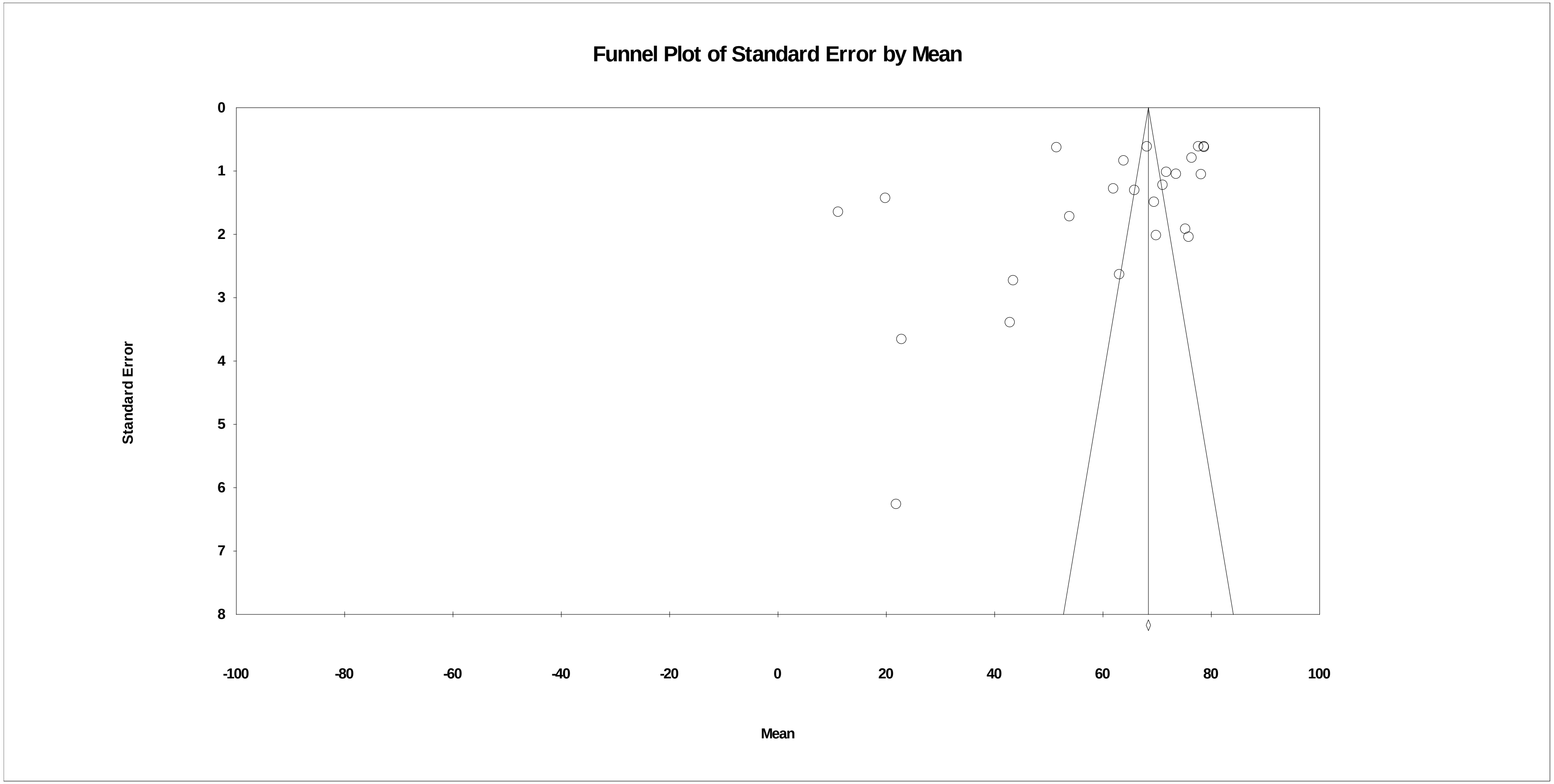

Supplemental figure 1c. Percentage of ST at work in office workers

Outcome	Study name	Comparison	Statistics for each study				Subgroup within study
			Mean	Lower limit	Upper limit	Standard error	
SED % work	Balogh, 2004 - O	Office workers	70.000	66.021	73.979	2.030	Blank
SED % work	Hadgraft 2016	Office workers	78.800	77.570	80.030	0.628	Blank
Sitting % work	Hallman 2018	Office workers	69.616	66.667	72.565	1.505	Combined
Sitting % work	Healy 2016	Office workers	77.800	76.570	79.030	0.628	Blank
SED % work	Li 2017	Office workers	73.669	71.588	75.750	1.062	Combined
SED % work	Mansoubi 2016	Office workers	76.000	71.971	80.029	2.055	Blank
SED % work	Mller 2004 - O	Office workers	75.390	71.610	79.170	1.929	Combined
SED % work	Nooijen 2018	Office workers	64.000	62.333	65.667	0.851	Blank
SED % work	Parry 2013 b	Office workers	78.290	76.197	80.383	1.068	Blank
Sitting % work	Ryan 2011	Office workers	66.000	63.418	68.582	1.317	Blank
SED % work	Thorp 2012 - O	Office workers	68.300	67.067	69.533	0.629	Blank
			72.531	68.981	76.081	1.811	

Mean and 95% CI

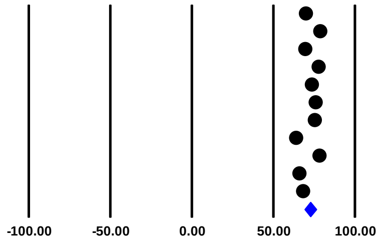

Sedentary time during work time (%)

Supplemental figure 1d. Funnel plot for ST (%) during work time in office workers

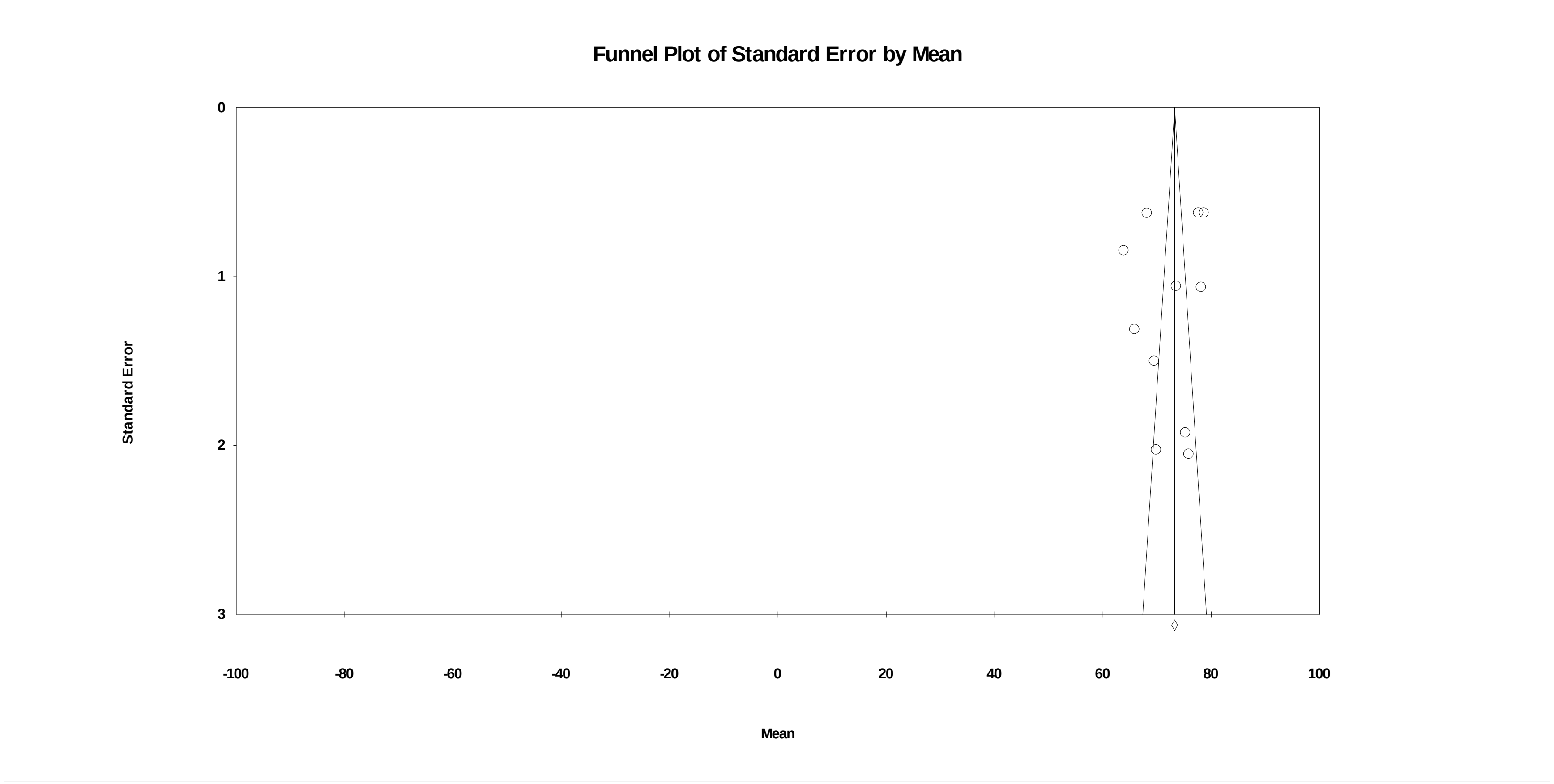

Supplemental figure 1e. Percentage of ST at work in all occupations except office workers

Outcome	Study name	Comparison	Mean	Lower limit	Upper limit	Standard error	Subgroup within study
SED % work	Balogh, 2004 - L	Laborers	20.000	17.171	22.829	1.443	Cleaners
SED % work	Brewer, 2016	Healthcare workers	71.874	69.854	73.894	1.031	Combined
SED % work	Gilson 2017	Drivers	23.000	15.806	30.194	3.671	Blank
SED % work	Loef 2018	Healthcare workers	51.596	50.337	52.855	0.642	Combined
SED % work	Mller 2004 - A	Academics	63.200	58.012	68.388	2.647	Scientists
SED % work	Mller 2004 - BC	Blue-collar workers	22.000	9.702	34.298	6.274	Blank
SED % work	Mller 2004 - tech	Technicians	43.000	36.326	49.674	3.405	Blank
SED % work	Thorp 2012 - C	Call centre	71.200	68.778	73.622	1.236	Blank
SED % work	Thorp 2012 - CS	Customer service	62.100	59.566	64.634	1.293	Blank
SED % work	Umukoro 2013	Healthcare workers	54.000	50.605	57.395	1.732	Nurses and aides
SED % work	van Dommelen 2016 - A	Academics	76.550	74.968	78.132	0.807	Combined
SED % work	van Dommelen 2016 - F	Financial services	78.863	77.612	80.114	0.638	Combined
SED % work	van Dommelen 2016 - L	Laborers	43.600	38.227	48.973	2.742	Men
SED % work	Vincent 2016	Protective services	11.300	8.046	14.554	1.660	Blank
			49.702	39.154	60.250	5.382	

Mean and 95% CI

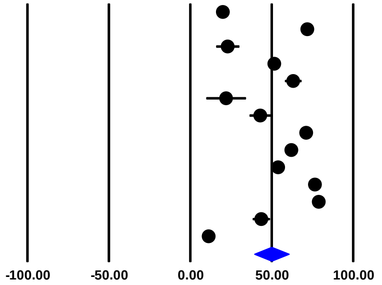

Sedentary time during work time (%)

Supplemental figure 1f. Percentage of ST at work in healthcare workers

Outcome	Study name	Comparison	Statistics for each study				Subgroup within study
			Mean	Lower limit	Upper limit	Standard error	
SED % work	Brewer, 2016	Healthcare workers	71.874	69.854	73.894	1.031	Combined
SED % work	Loef 2018	Healthcare workers	51.596	50.337	52.855	0.642	Combined
SED % work	Umukoro 2013	Healthcare workers	54.000	50.605	57.395	1.732	Nurses and aides
			59.167	45.141	73.194	7.156	

Mean and 95% CI

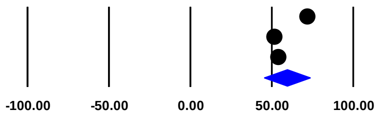

Sedentary time during work time (%)

Supplemental figure 1g. Percentage of ST at work in all occupations except healthcare workers

Outcome	Study name	Comparison	Statistics for each study: Mean	Lower limit	Upper limit	Standard error	Subgroup within study
SED % work	Balogh, 2004 - L	Laborers	20.000	17.171	22.829	1.443	Cleaners
SED % work	Balogh, 2004 - O	Office workers	70.000	66.021	73.979	2.030	Blank
SED % work	Gilson 2017	Drivers	23.000	15.806	30.194	3.671	Blank
SED % work	Hadgraft 2016	Office workers	78.800	77.570	80.030	0.628	Blank
Sitting % work	Hallman 2018	Office workers	69.616	66.667	72.565	1.505	Combined
Sitting % work	Healy 2016	Office workers	77.800	76.570	79.030	0.628	Blank
SED % work	Li 2017	Office workers	73.669	71.588	75.750	1.062	Combined
SED % work	Mansoubi 2016	Office workers	76.000	71.971	80.029	2.055	Blank
SED % work	Mller 2004 - A	Academics	63.200	58.012	68.388	2.647	Scientists
SED % work	Mller 2004 - BC	Blue-collar workers	22.000	9.702	34.298	6.274	Blank
SED % work	Mller 2004 - O	Office workers	75.390	71.610	79.170	1.929	Combined
SED % work	Mller 2004 - tech	Technicians	43.000	36.326	49.674	3.405	Blank
SED % work	Nooijen 2018	Office workers	64.000	62.333	65.667	0.851	Blank
SED % work	Parry 2013 b	Office workers	78.290	76.197	80.383	1.068	Blank
Sitting % work	Ryan 2011	Office workers	66.000	63.418	68.582	1.317	Blank
SED % work	Thorp 2012 - C	Call centre	71.200	68.778	73.622	1.236	Blank
SED % work	Thorp 2012 - CS	Customer service	62.100	59.566	64.634	1.293	Blank
SED % work	Thorp 2012 - O	Office workers	68.300	67.067	69.533	0.629	Blank
SED % work	van Dommelen 2016 - A	Academics	76.550	74.968	78.132	0.807	Combined
SED % work	van Dommelen 2016 - F	Financial services	78.863	77.612	80.114	0.638	Combined
SED % work	van Dommelen 2016 - L	Laborers	43.600	38.227	48.973	2.742	Men
SED % work	Vincent 2016	Protective services	11.300	8.046	14.554	1.660	Blank
			60.091	53.994	66.189	3.111	

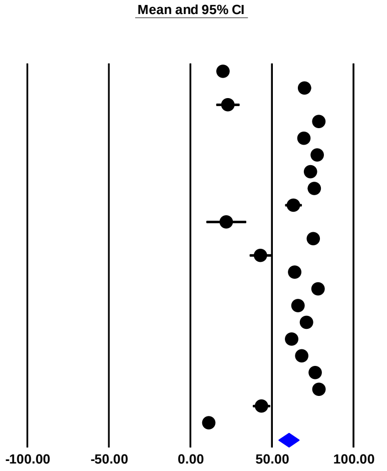

Sedentary time during work time (%)

Supplemental figure 1h. Percentage of ST during wake time across all occupations

Outcome	Study name	Comparison	Statistics for each study: Mean	Lower limit	Upper limit	Standard error	Subgroup within study
SED % wake	Arias, 2015	Laborers	37.000	32.243	41.757	2.427	Blank
SED % wake	Cuddy, 2015	Protective services	49.000	44.952	53.048	2.066	Blank
Sitting % wake	Evans, 2012	Office workers	76.942	73.880	80.004	1.562	Combined
SED % wake	Gao 2016 - O	Office workers	80.200	76.233	84.167	2.024	Blank
SED % wake	Gao 2016 - OSS	Office workers with sit-stand desk	66.200	57.243	75.157	4.570	Blank
SED % wake	Gilson 2012b	Office workers	75.800	69.713	81.887	3.106	Blank
SED % wake	Hadgraft 2016	Office workers	64.600	63.512	65.688	0.555	Blank
Sitting % wake	Healy 2016	Office workers	64.600	63.512	65.688	0.555	Blank
SED % wake	Kozey-Keadley 2012	Office workers	67.000	61.171	72.829	2.974	Blank
SED % wake	Nooijen 2018	Office workers	60.000	59.111	60.889	0.454	Blank
SED % wake	Parry 2013 b	Office workers	72.850	71.093	74.607	0.897	Blank
SED % wake	Peavler 2012	Office workers	66.830	64.561	69.100	1.158	Combined
Sitting % wake	Pedersen 2016	Office workers	65.720	58.406	73.034	3.732	Blank
SED % wake	Reed 2018 a	Healthcare workers	49.520	48.614	50.426	0.462	Nurses
SED % wake	Schwartz 2016	Office workers	50.100	48.866	51.334	0.630	Blank
SED % wake	Steeves 2015 - CS	Customer service - hospitality	49.997	49.541	50.454	0.233	Combined
SED % wake	Steeves 2015 - D	Drivers	50.566	50.216	50.915	0.178	Combined
SED % wake	Steeves 2015 - EAS	Engineers, architects and scientists	65.000	64.490	65.510	0.260	Blank
SED % wake	Steeves 2015 - EPP	Extractive and precision production occupations	52.400	52.057	52.743	0.175	Blank
SED % wake	Steeves 2015 - F	Factory workers	46.709	46.148	47.270	0.286	Combined
SED % wake	Steeves 2015 - H	Healthcare workers	53.762	53.484	54.041	0.142	Combined
SED % wake	Steeves 2015 - house	Private household occupations	51.700	45.755	57.645	3.033	Blank
SED % wake	Steeves 2015 - L	Laborers	46.319	46.124	46.514	0.100	Combined
SED % wake	Steeves 2015 - Mgt	Management related occupations	60.300	59.964	60.636	0.171	Blank
SED % wake	Steeves 2015 - MRSD	Material recording, scheduling, distributing clerks	52.300	50.322	54.278	1.009	Blank
SED % wake	Steeves 2015 - O	Office workers	58.145	57.984	58.306	0.082	Combined
SED % wake	Steeves 2015 - Other	other professional speciality occupations	59.900	59.335	60.465	0.288	Blank
SED % wake	Steeves 2015 - personal	Personal service occupations	54.500	53.190	55.810	0.668	Blank
SED % wake	Steeves 2015 - PS	Protective services	59.700	58.395	61.005	0.666	Protective service occupation
SED % wake	Steeves 2015 - sales	Sales reps., finance, business, & commodities	58.200	57.508	58.892	0.353	Blank
SED % wake	Steeves 2015 - ST	School teachers	57.500	56.973	58.027	0.269	Blank
SED % wake	Steeves 2015 - sups	Supervisors and proprietors, sales occupations	49.000	47.868	50.132	0.577	Blank
SED % wake	Steeves 2015 - Tech	Technicians and related support occupations	58.500	57.647	59.353	0.435	Blank
SED % wake	Steeves 2015 - WAEA	Writers, artists, entertainers, and athletes	58.900	57.584	60.216	0.671	Blank
SED % wake	Steeves 2018 - ADE	Blank	62.400	61.681	63.119	0.367	Arts, design, entertainment, sports, media
SED % wake	Steeves 2018 - Arch	Blank	63.300	62.647	63.953	0.333	Architecture, engineering
SED % wake	Steeves 2018 - BF	Blank	61.100	60.910	61.290	0.097	Business, financial operations
SED % wake	Steeves 2018 - C	Customer service - hospitality	43.800	43.228	44.372	0.292	Food preparation, serving
SED % wake	Steeves 2018 - CM	Blank	65.900	65.242	66.558	0.336	Computer, mathematical
SED % wake	Steeves 2018 - CSS	Blank	64.200	63.516	64.884	0.349	Community, social services
SED % wake	Steeves 2018 - D	Drivers	47.000	46.764	47.236	0.120	Transportation, material moving
SED % wake	Steeves 2018 - Educ	Blank	55.300	54.930	55.670	0.189	Education, training, library
SED % wake	Steeves 2018 - H	Healthcare workers	56.376	56.139	56.613	0.121	Combined
SED % wake	Steeves 2018 - IMR	Blank	51.200	50.702	51.698	0.254	Installation, maintenance, repair
SED % wake	Steeves 2018 - L	Laborers	46.637	46.457	46.817	0.092	Combined
SED % wake	Steeves 2018 - Legal	Blank	65.900	64.882	66.918	0.520	Legal
SED % wake	Steeves 2018 - LPSS	Blank	64.800	64.126	65.474	0.344	Life, physical, social science
SED % wake	Steeves 2018 - O	Office workers	58.217	58.118	58.315	0.050	Combined
SED % wake	Steeves 2018 - P	Protective services	60.300	59.852	60.748	0.229	Protective service
SED % wake	Steeves 2018 - PCS	Blank	50.900	50.183	51.617	0.366	Personal care service
SED % wake	Steeves 2018 - Prod	Blank	52.700	52.480	52.920	0.112	Production
SED % wake	Steeves 2018 - Sales	Blank	55.400	55.110	55.690	0.148	Sales & related
SED % wake	Torquati 2018	Healthcare workers	58.400	55.970	60.830	1.240	Nurses
SED % wake	van Dommelen 2016 - A	Academics	64.537	63.235	65.839	0.664	Combined
SED % wake	van Dommelen 2016 - F	Financial services	69.313	68.064	70.562	0.637	Combined
SED % wake	van Dommelen 2016 - L	Laborers	55.500	52.543	58.457	1.509	Men
SED % wake	Wick 2016	Office workers with sit-stand desk	77.100	73.571	80.629	1.801	Blank
SED % wake	Ying 2014	Office workers	80.670	78.587	82.753	1.063	Blank
			58.792	57.284	60.301	0.770	

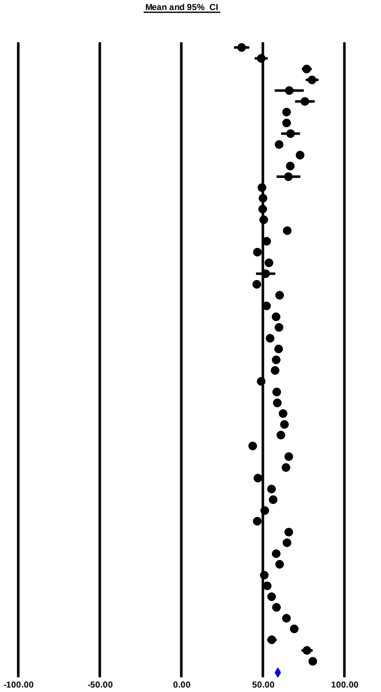

Sedentary time during wake time (%)

Supplemental figure 1i. Funnel plot for ST (%) during wake time across all occupations

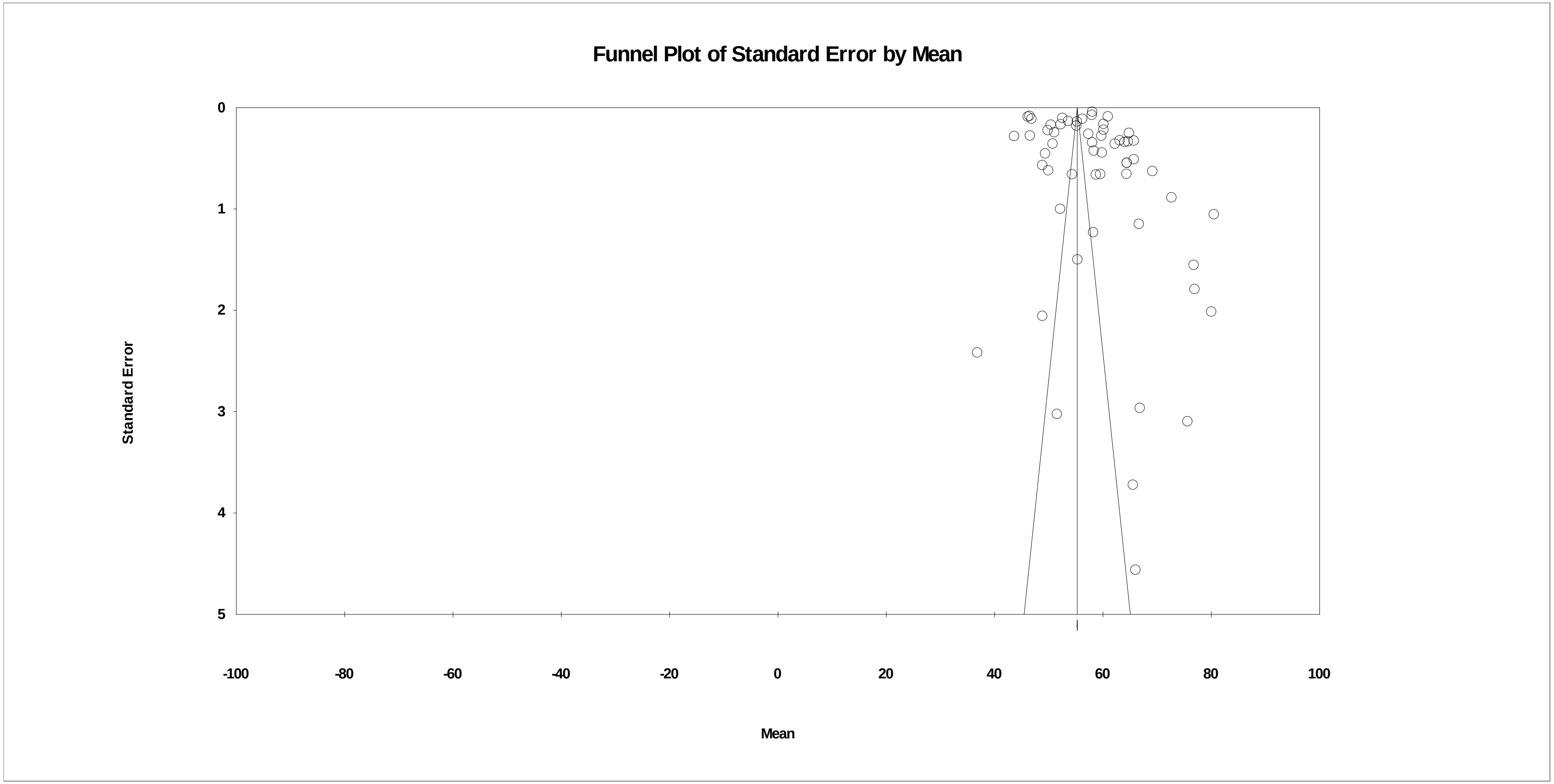

Supplemental figure 1j. Percentage of ST during wake time in office workers

Outcome	Study name	Comparison	Mean	Lower limit	Upper limit	Standard error	Subgroup within study
			Statistics for each study				
Sitting % wake	Evans, 2012	Office workers	76.942	73.880	80.004	1.562	Combined
SED % wake	Gao 2016 - O	Office workers	80.200	76.233	84.167	2.024	Blank
SED % wake	Gilson 2012b	Office workers	75.800	69.713	81.887	3.106	Blank
SED % wake	Hadgraft 2016	Office workers	64.600	63.512	65.688	0.555	Blank
Sitting % wake	Healy 2016	Office workers	64.600	63.512	65.688	0.555	Blank
SED % wake	Kozey-Keadley 2012	Office workers	67.000	61.171	72.829	2.974	Blank
SED % wake	Nooijen 2018	Office workers	60.000	59.111	60.889	0.454	Blank
SED % wake	Parry 2013 b	Office workers	72.850	71.093	74.607	0.897	Blank
SED % wake	Peavler 2012	Office workers	66.830	64.561	69.100	1.158	Combined
Sitting % wake	Pedersen 2016	Office workers	65.720	58.406	73.034	3.732	Blank
SED % wake	Schwartz 2016	Office workers	50.100	48.866	51.334	0.630	Blank
SED % wake	Steeves 2015 - O	Office workers	58.145	57.984	58.306	0.082	Combined
SED % wake	Steeves 2018 - O	Office workers	58.217	58.118	58.315	0.050	Combined
SED % wake	Ying 2014	Office workers	80.670	78.587	82.753	1.063	Blank
			66.056	64.575	67.536	0.755	

Mean and 95% CI

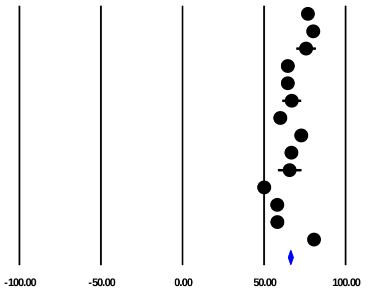

Sedentary time during wake time (%)

Supplemental figure 1k. Funnel plot for ST (%) during wake time across office workers

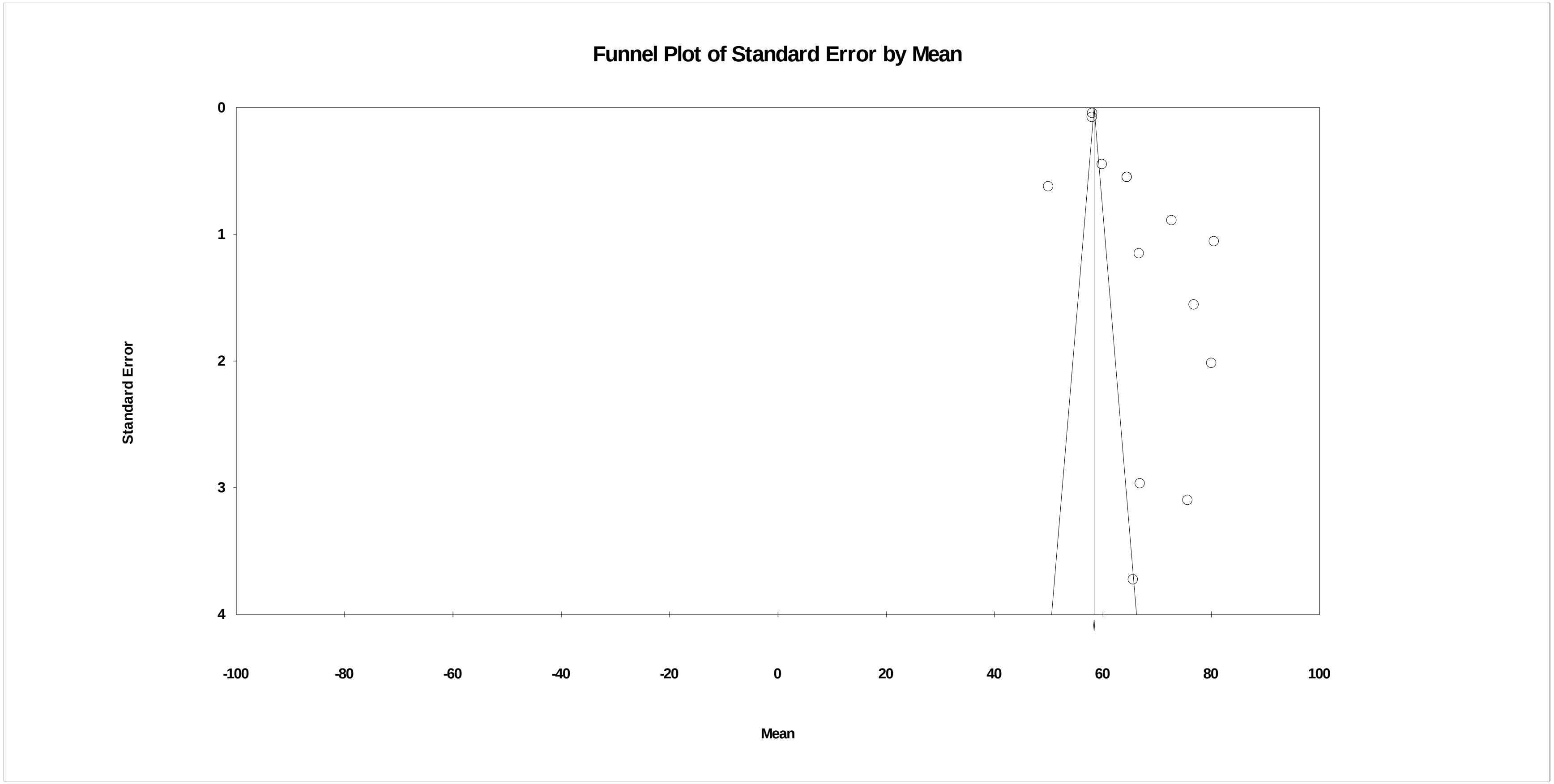

Supplemental figure 1l. Percentage of ST during wake time in all occupations except office workers

Outcome	Study name	Comparison	Mean	Lower limit	Upper limit	Standard error	Subgroup within study
			Statistics for each study				
SED % wake	Arias, 2015	Laborers	37.000	32.243	41.757	2.427	Blank
SED % wake	Cuddy, 2015	Protective services	49.000	44.952	53.048	2.066	Blank
SED % wake	Gao 2016 - OSS	Office workers with sit-stand desk	66.200	57.243	75.157	4.570	Blank
SED % wake	Reed 2018 a	Healthcare workers	49.520	48.614	50.426	0.462	Nurses
SED % wake	Steeves 2015 - CS	Customer service - hospitality	49.997	49.541	50.454	0.233	Combined
SED % wake	Steeves 2015 - D	Drivers	50.566	50.216	50.915	0.178	Combined
SED % wake	Steeves 2015 - EAS	Engineers, architects and scientists	65.000	64.490	65.510	0.260	Blank
SED % wake	Steeves 2015 - EPP	Extractive and precision production occupations	52.400	52.057	52.743	0.175	Blank
SED % wake	Steeves 2015 - F	Factory workers	46.709	46.148	47.270	0.286	Combined
SED % wake	Steeves 2015 - H	Healthcare workers	53.762	53.484	54.041	0.142	Combined
SED % wake	Steeves 2015 - house	Private household occupations	51.700	45.755	57.645	3.033	Blank
SED % wake	Steeves 2015 - L	Laborers	46.319	46.124	46.514	0.100	Combined
SED % wake	Steeves 2015 - Mgt	Management related occupations	60.300	59.964	60.636	0.171	Blank
SED % wake	Steeves 2015 - MRSD	Material recording, scheduling, distributing clerks	52.300	50.322	54.278	1.009	Blank
SED % wake	Steeves 2015 - Other	other professional speciality occupations	59.900	59.335	60.465	0.288	Blank
SED % wake	Steeves 2015 - personal	Personal service occupations	54.500	53.190	55.810	0.668	Blank
SED % wake	Steeves 2015 - PS	Protective services	59.700	58.395	61.005	0.666	Protective service occupation
SED % wake	Steeves 2015 - sales	Sales reps., finance, business, & commodities	58.200	57.508	58.892	0.353	Blank
SED % wake	Steeves 2015 - ST	School teachers	57.500	56.973	58.027	0.269	Blank
SED % wake	Steeves 2015 - sups	Supervisors and proprietors, sales occupations	49.000	47.868	50.132	0.577	Blank
SED % wake	Steeves 2015 - Tech	Technicians and related support occupations	58.500	57.647	59.353	0.435	Blank
SED % wake	Steeves 2015 - WAEA	Writers, artists, entertainers, and athletes	58.900	57.584	60.216	0.671	Blank
SED % wake	van Dommelen 2016 - A	Academics	64.537	63.235	65.839	0.664	Combined
SED % wake	van Dommelen 2016 - F	Financial services	69.313	68.064	70.562	0.637	Combined
SED % wake	van Dommelen 2016 - L	Laborers	55.500	52.543	58.457	1.509	Men
SED % wake	Wick 2016	Office workers with sit-stand desk	77.100	73.571	80.629	1.801	Blank
			55.847	53.399	58.294	1.249	

Mean and 95% CI

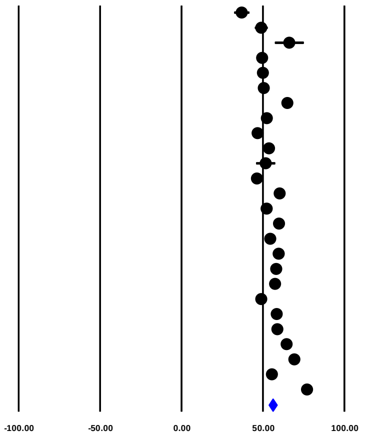

Sedentary time during wake time (%)

Supplemental figure 1m. Percentage of ST during wake time in laborers

Outcome	Study name	Comparison	Mean	Lower limit	Upper limit	Standard error	Subgroup within study
SED % wake	Arias, 2015	Laborers	37.000	32.243	41.757	2.427	Blank
SED % wake	Steeves 2015 - L	Laborers	46.319	46.124	46.514	0.100	Combined
SED % wake	Steeves 2018 - L	Laborers	46.637	46.457	46.817	0.092	Combined
SED % wake	van Dommelen 2016 - L	Laborers	55.500	52.543	58.457	1.509	Men
			46.869	45.950	47.788	0.469	

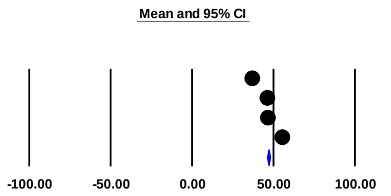

Sedentary time during wake time (%)

Supplemental figure 1n. Percentage of ST during wake time in all occupations except laborers

Outcome	Study name	Comparison	Statistics for each study				Subgroup within study
			Mean	Lower limit	Upper limit	Standard error	
SED % wake	Cuddy, 2015	Protective services	49.000	44.952	53.048	2.066	Blank
Sitting % wake	Evans, 2012	Office workers	76.942	73.880	80.004	1.562	Combined
SED % wake	Gao 2016 - O	Office workers	80.200	76.233	84.167	2.024	Blank
SED % wake	Gao 2016 - OSS	Office workers with sit-stand desk	66.200	57.243	75.157	4.570	Blank
SED % wake	Gilson 2012b	Office workers	75.800	69.713	81.887	3.106	Blank
SED % wake	Hadgraft 2016	Office workers	64.600	63.512	65.688	0.555	Blank
SED % wake	Kozey-Keadley 2012	Office workers	67.000	61.171	72.829	2.974	Blank
SED % wake	Parry 2013 b	Office workers	72.850	71.093	74.607	0.897	Blank
SED % wake	Peavler 2012	Office workers	66.830	64.561	69.100	1.158	Combined
SED % wake	Reed 2018 a	Healthcare workers	49.520	48.614	50.426	0.462	Nurses
SED % wake	Steeves 2015 - CS	Customer service - hospitality	49.997	49.541	50.454	0.233	Combined
SED % wake	Steeves 2015 - D	Drivers	50.566	50.216	50.915	0.178	Combined
SED % wake	Steeves 2015 - EAS	Engineers, architects and scientists	65.000	64.490	65.510	0.260	Blank
SED % wake	Steeves 2015 - EPP	Extractive and precision production occupations	52.400	52.057	52.743	0.175	Blank
SED % wake	Steeves 2015 - F	Factory workers	46.709	46.148	47.270	0.286	Combined
SED % wake	Steeves 2015 - H	Healthcare workers	53.762	53.484	54.041	0.142	Combined
SED % wake	Steeves 2015 - house	Private household occupations	51.700	45.755	57.645	3.033	Blank
SED % wake	Steeves 2015 - Mgt	Management related occupations	60.300	59.964	60.636	0.171	Blank
SED % wake	Steeves 2015 - MRSD	Material recording, scheduling, distributing clerks	52.300	50.322	54.278	1.009	Blank
SED % wake	Steeves 2015 - O	Office workers	58.145	57.984	58.306	0.082	Combined
SED % wake	Steeves 2015 - Other	other professional speciality occupations	59.900	59.335	60.465	0.288	Blank
SED % wake	Steeves 2015 - personal	Personal service occupations	54.500	53.190	55.810	0.668	Blank
SED % wake	Steeves 2015 - PS	Protective services	59.700	58.395	61.005	0.666	Protective service occupation
SED % wake	Steeves 2015 - sales	Sales reps., finance, business, & commodities	58.200	57.508	58.892	0.353	Blank
SED % wake	Steeves 2015 - ST	School teachers	57.500	56.973	58.027	0.269	Blank
SED % wake	Steeves 2015 - sups	Supervisors and proprietors, sales occupations	49.000	47.868	50.132	0.577	Blank
SED % wake	Steeves 2015 - Tech	Technicians and related support occupations	58.500	57.647	59.353	0.435	Blank
SED % wake	Steeves 2015 - WAEA	Writers, artists, entertainers, and athletes	58.900	57.584	60.216	0.671	Blank
SED % wake	van Dommelen 2016 - A	Academics	64.537	63.235	65.839	0.664	Combined
SED % wake	van Dommelen 2016 - F	Financial services	69.313	68.064	70.562	0.637	Combined
SED % wake	Wick 2016	Office workers with sit-stand desk	77.100	73.571	80.629	1.801	Blank
SED % wake	Ying 2014	Office workers	80.670	78.587	82.753	1.063	Blank
			60.844	59.020	62.668	0.930	

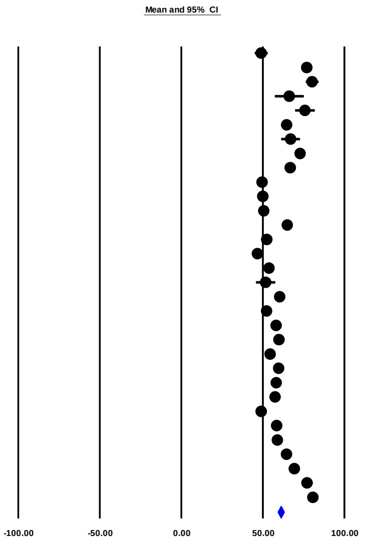

Sedentary time during wake time (%)

Supplemental figure 1o. Percentage of ST during wake time in protective services workers

Outcome	Study name	Comparison	Mean	Lower limit	Upper limit	Standard error	Subgroup within study
SED % wake	Cuddy, 2015	Protective services	49.000	44.952	53.048	2.066	Blank
SED % wake	Steeves 2015 - PS	Protective services	59.700	58.395	61.005	0.666	Protective service occupation
SED % wake	Steeves 2018 - P	Protective services	60.300	59.852	60.748	0.229	Protective service
			57.409	54.315	60.502	1.578	

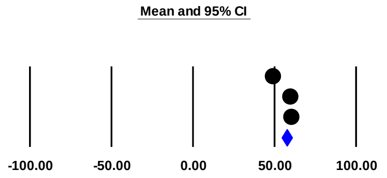

Sedentary time during wake time (%)

Supplemental figure 1p. Percentage of ST during wake time in health care workers

Outcome	Study name	Comparison	Statistics for each study				Subgroup within study
			Mean	Lower limit	Upper limit	Standard error	
SED % wake	Reed 2018 a	Healthcare workers	49.520	48.614	50.426	0.462	Nurses
SED % wake	Steeves 2015 - H	Healthcare workers	53.762	53.484	54.041	0.142	Combined
SED % wake	Steeves 2018 - H	Healthcare workers	56.376	56.139	56.613	0.121	Combined
SED % wake	Torquati 2018	Healthcare workers	58.400	55.970	60.830	1.240	Nurses
			54.333	51.984	56.683	1.199	

Mean and 95% CI

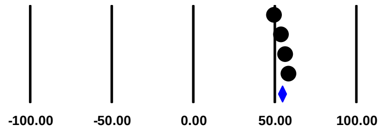

Sedentary time during wake time (%)

Supplemental figure 1q. Percentage of ST during wake time in all occupations except health care workers

Outcome	Study name	Comparison	Mean	Lower limit	Upper limit	Standard error	Subgroup within study
			Statistics for each study				
SED % wake	Arias, 2015	Laborers	37.000	32.243	41.757	2.427	Blank
SED % wake	Cuddy, 2015	Protective services	49.000	44.952	53.048	2.066	Blank
Sitting % wake	Evans, 2012	Office workers	76.942	73.880	80.004	1.562	Combined
SED % wake	Gao 2016 - O	Office workers	80.200	76.233	84.167	2.024	Blank
SED % wake	Gao 2016 - OSS	Office workers with sit-stand desk	66.200	57.243	75.157	4.570	Blank
SED % wake	Gilson 2012b	Office workers	75.800	69.713	81.887	3.106	Blank
SED % wake	Hadgraft 2016	Office workers	64.600	63.512	65.688	0.555	Blank
Sitting % wake	Healy 2016	Office workers	64.600	63.512	65.688	0.555	Blank
SED % wake	Kozey-Keadley 2012	Office workers	67.000	61.171	72.829	2.974	Blank
SED % wake	Nooijen 2018	Office workers	60.000	59.111	60.889	0.454	Blank
SED % wake	Parry 2013 b	Office workers	72.850	71.093	74.607	0.897	Blank
SED % wake	Peavler 2012	Office workers	66.830	64.561	69.100	1.158	Combined
Sitting % wake	Pedersen 2016	Office workers	65.720	58.406	73.034	3.732	Blank
SED % wake	Schwartz 2016	Office workers	50.100	48.866	51.334	0.630	Blank
SED % wake	Steeves 2015 - CS	Customer service - hospitality	49.997	49.541	50.454	0.233	Combined
SED % wake	Steeves 2015 - D	Drivers	50.566	50.216	50.915	0.178	Combined
SED % wake	Steeves 2015 - EAS	Engineers, architects and scientists	65.000	64.490	65.510	0.260	Blank
SED % wake	Steeves 2015 - EPP	Extractive and precision production occupations	52.400	52.057	52.743	0.175	Blank
SED % wake	Steeves 2015 - F	Factory workers	46.709	46.148	47.270	0.286	Combined
SED % wake	Steeves 2015 - house	Private household occupations	51.700	45.755	57.645	3.033	Blank
SED % wake	Steeves 2015 - L	Laborers	46.319	46.124	46.514	0.100	Combined
SED % wake	Steeves 2015 - Mgt	Management related occupations	60.300	59.964	60.636	0.171	Blank
SED % wake	Steeves 2015 - MRSD	Material recording, scheduling, distributing clerks	52.300	50.322	54.278	1.009	Blank
SED % wake	Steeves 2015 - O	Office workers	58.145	57.984	58.306	0.082	Combined
SED % wake	Steeves 2015 - Other	other professional speciality occupations	59.900	59.335	60.465	0.288	Blank
SED % wake	Steeves 2015 - personal	Personal service occupations	54.500	53.190	55.810	0.668	Blank
SED % wake	Steeves 2015 - PS	Protective services	59.700	58.395	61.005	0.666	Protective service occupation
SED % wake	Steeves 2015 - sales	Sales reps., finance, business, & commodities	58.200	57.508	58.892	0.353	Blank
SED % wake	Steeves 2015 - ST	School teachers	57.500	56.973	58.027	0.269	Blank
SED % wake	Steeves 2015 - sups	Supervisors and proprietors, sales occupations	49.000	47.868	50.132	0.577	Blank
SED % wake	Steeves 2015 - Tech	Technicians and related support occupations	58.500	57.647	59.353	0.435	Blank
SED % wake	Steeves 2015 - WAEA	Writers, artists, entertainers, and athletes	58.900	57.584	60.216	0.671	Blank
SED % wake	Steeves 2018 - ADE	Blank	62.400	61.681	63.119	0.367	Arts, design, entertainment, sports, media
SED % wake	Steeves 2018 - Arch	Blank	63.300	62.647	63.953	0.333	Architecture, engineering
SED % wake	Steeves 2018 - BF	Blank	61.100	60.910	61.290	0.097	Business, financial operations
SED % wake	Steeves 2018 - C	Customer service - hospitality	43.800	43.228	44.372	0.292	Food preparation, serving
SED % wake	Steeves 2018 - CM	Blank	65.900	65.242	66.558	0.336	Computer, mathematical
SED % wake	Steeves 2018 - CSS	Blank	64.200	63.516	64.884	0.349	Community, social services
SED % wake	Steeves 2018 - D	Drivers	47.000	46.764	47.236	0.120	Transportation, material moving
SED % wake	Steeves 2018 - Educ	Blank	55.300	54.930	55.670	0.189	Education, training, library
SED % wake	Steeves 2018 - IMR	Blank	51.200	50.702	51.698	0.254	Installation, maintenance, repair
SED % wake	Steeves 2018 - L	Laborers	46.637	46.457	46.817	0.092	Combined
SED % wake	Steeves 2018 - Legal	Blank	65.900	64.882	66.918	0.520	Legal
SED % wake	Steeves 2018 - LPSS	Blank	64.800	64.126	65.474	0.344	Life, physical, social science
SED % wake	Steeves 2018 - O	Office workers	58.217	58.118	58.315	0.050	Combined
SED % wake	Steeves 2018 - P	Protective services	60.300	59.852	60.748	0.229	Protective service
SED % wake	Steeves 2018 - PCS	Blank	50.900	50.183	51.617	0.366	Personal care service
SED % wake	Steeves 2018 - Prod	Blank	52.700	52.480	52.920	0.112	Production
SED % wake	Steeves 2018 - Sales	Blank	55.400	55.110	55.690	0.148	Sales & related
SED % wake	van Dommelen 2016 - A	Academics	64.537	63.235	65.839	0.664	Combined
SED % wake	van Dommelen 2016 - F	Financial services	69.313	68.064	70.562	0.637	Combined
SED % wake	van Dommelen 2016 - L	Laborers	55.500	52.543	58.457	1.509	Men
SED % wake	Wick 2016	Office workers with sit-stand desk	77.100	73.571	80.629	1.801	Blank
SED % wake	Ying 2014	Office workers	80.670	78.587	82.753	1.063	Blank
			59.135	57.498	60.772	0.835	

Mean and 95% CI

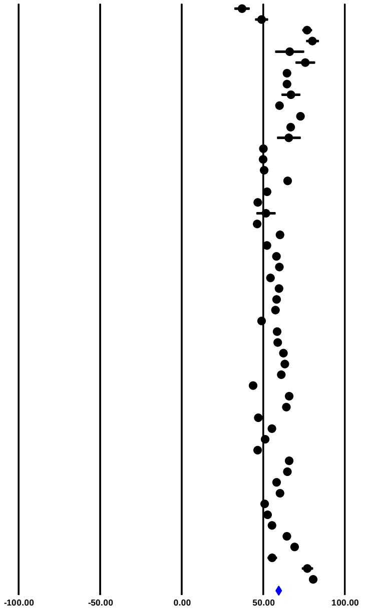

Sedentary time during wake time (%)

Supplemental figure 1r. ST, minutes/day during wake time across all occupations

Outcome	Study name	Comparison	Statistics for each study: Mean	Lower limit	Upper limit	Standard error	Subgroup within study
SED - wake	Alkajah, 2012	Office workers	575.005	555.799	594.210	9.799	Combined
SED - wake	Andersen, 2011	Drivers	504.000	484.461	523.539	9.969	Blank
SED - wake	Arias, 2015	Laborers	760.000	581.081	938.919	91.287	Blank
SED - wake	Bergman 2018	Office workers	558.214	535.412	581.016	11.634	Combined
SED - wake	Brakenridge, 2016	Office workers	622.100	611.183	633.017	5.570	Blank
SED - wake	Chau, 2014	Office workers	347.000	326.558	367.442	10.430	Blank
SED - wake	Chitkara, 2014	Call centre workers	1166.600	687.726	1645.474	244.328	Blank
Sitting - wake	Evans, 2012	Office workers	345.000	322.776	367.224	11.339	Combined
SED - wake	French 2007	Drivers	342.800	330.648	354.952	6.200	Blank
SED - wake	Gilson 2016	Office workers	582.000	550.848	613.152	15.894	Blank
Sitting - wake	Healy 2016	Office workers	620.600	610.148	631.052	5.333	Blank
SED - wake	Jancey 2016	Office workers	401.300	389.369	413.231	6.087	Blank
SED - wake	Koepp 2013	Office workers	1020.000	998.440	1041.560	11.000	Blank
SED - wake	Lagersted-Olsen 2014	Office workers	336.000	310.631	361.369	12.944	Blank
SED - wake	Miller 2004 - A	Academics	564.000	535.399	592.601	14.592	Scientists
SED - wake	Miller 2004 - BC	Blue-collar workers	390.000	326.886	453.114	32.201	Blank
SED - wake	Miller 2004 - O	Office workers	626.177	597.893	654.461	14.431	Combined
SED - wake	Miller 2004 - tech	Technicians	468.000	421.326	514.674	23.814	Blank
SED - wake	Nooijen 2018	Office workers	582.000	572.664	591.336	4.763	Blank
SED - wake	Parry 2013 a	Office workers	676.000	659.729	692.271	8.301	Blank
SED - wake	Peavler 2012	Office workers	556.477	525.928	587.025	15.586	Combined
SED - wake	Pontt 2015 - L	Laborers	492.000	457.060	526.940	17.827	Blank
SED - wake	Pontt 2015 - O	Office workers	600.000	565.060	634.940	17.827	Blank
SED - wake	Reed 2018 a	Healthcare workers	445.000	433.772	456.228	5.729	Nurses
SED - wake	Ryde 2013	Office workers	348.000	334.228	361.772	7.026	Blank
SED - wake	Smith 2015	Office workers	636.000	616.716	655.284	9.839	Blank
SED - wake	Swartz 2014	Office workers	376.500	374.501	378.499	1.020	Blank
SED - wake	Tigbe 2011 - O	Office workers	1056.000	1037.142	1074.858	9.621	Blank
SED - wake	Tigbe 2011 - PD	Postal workers - delivery	960.000	898.713	1021.287	31.270	Blank
SED - wake	Torquati 2018	Healthcare workers	486.300	455.510	517.090	15.710	Nurses
SED - wake	Urda 2016	Office workers	356.368	341.273	371.464	7.702	Combined
SED - wake	Vareka Mato 2016	Drivers	780.000	749.177	810.823	15.727	Blank
SED - wake	Varela Mato 2017	Drivers	715.000	669.441	760.559	23.245	Blank
SED - wake	Weiler 2015	Athletes	500.600	477.472	523.728	11.800	Blank
SED - wake	Wong 2014	Drivers	468.000	430.238	505.762	19.267	Blank
			565.571	510.389	620.752	28.154	

Mean and 95% CI

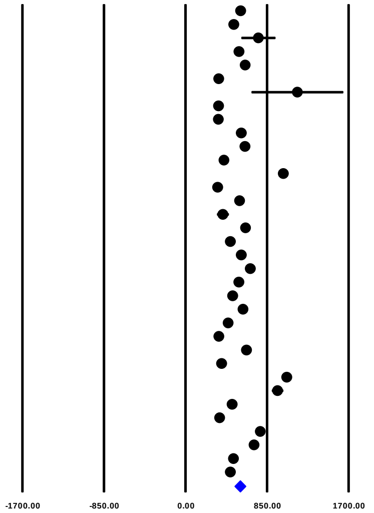

Sedentary time during wake time (min/day)

Supplemental figure 1s. Funnel plot for ST (min/day) at work across all occupations

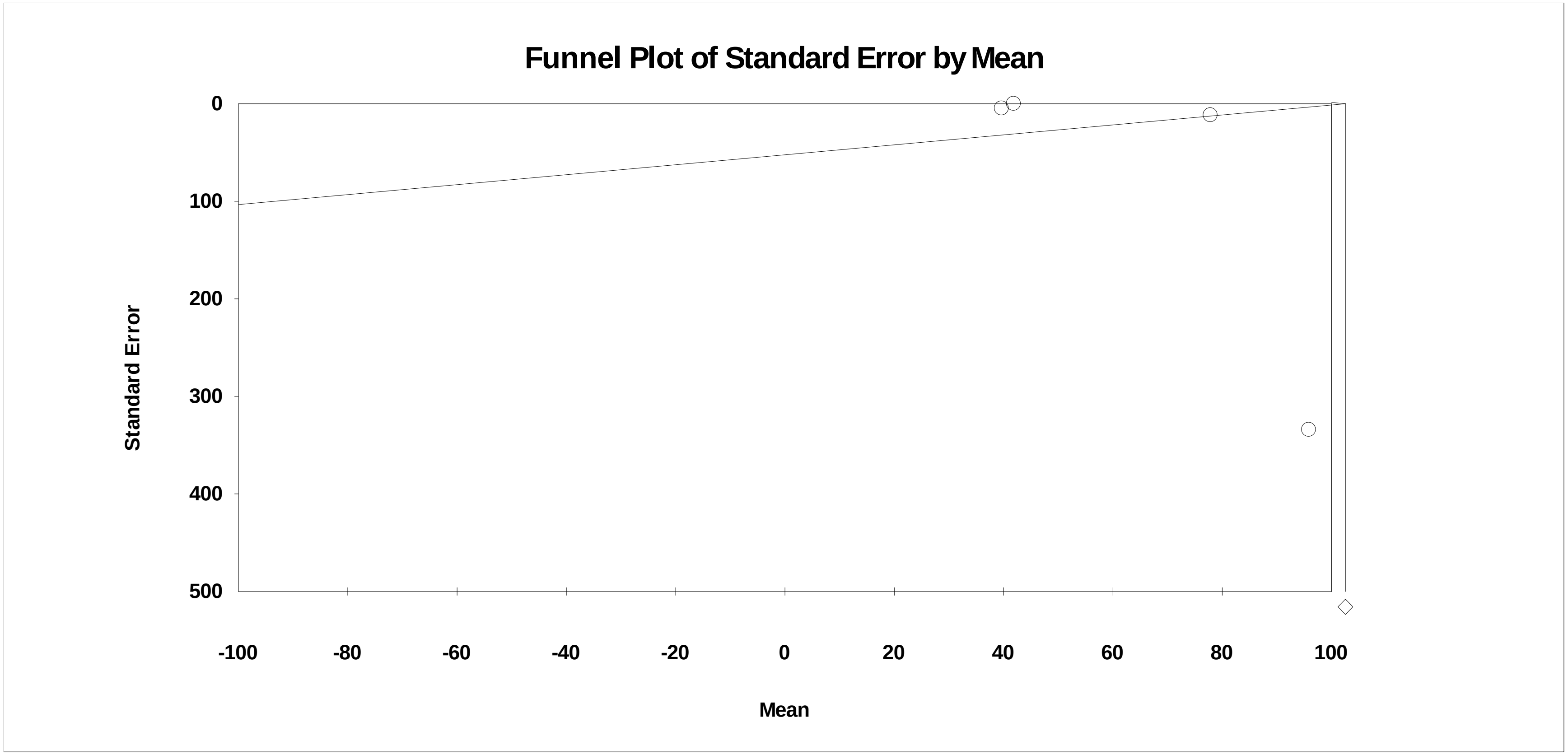

Supplemental figure 1t. ST, minutes/day during wake time across all office workers

Outcome	Studyname	Comparison	Statistics for each study				Subgroup within study
			Mean	Lower limit	Upper limit	Standard error	
SED - wake	Alkajah, 2012	Office workers	575.005	555.799	594.210	9.799	Combined
SED - wake	Bergman 2018	Office workers	558.214	535.412	581.016	11.634	Combined
SED - wake	Brakenridge, 2016	Office workers	622.100	611.183	633.017	5.570	Blank
SED - wake	Chau, 2014	Office workers	347.000	326.558	367.442	10.430	Blank
Sitting - wake	Evans, 2012	Office workers	345.000	322.776	367.224	11.339	Combined
SED - wake	Gilson 2016	Office workers	582.000	550.848	613.152	15.894	Blank
Sitting - wake	Healy 2016	Office workers	620.600	610.148	631.052	5.333	Blank
SED - wake	Jancey 2016	Office workers	401.300	389.369	413.231	6.087	Blank
SED - wake	Koepp 2013	Office workers	1020.000	998.440	1041.560	11.000	Blank
SED - wake	Lagersted-Olsen 2014	Office workers	336.000	310.631	361.369	12.944	Blank
SED - wake	Miller 2004 - O	Office workers	626.177	597.893	654.461	14.431	Combined
SED - wake	Nooijen 2018	Office workers	582.000	572.664	591.336	4.763	Blank
SED - wake	Parry 2013 a	Office workers	676.000	659.729	692.271	8.301	Blank
SED - wake	Peavler 2012	Office workers	556.477	525.928	587.025	15.586	Combined
SED - wake	Pontt 2015 - O	Office workers	600.000	565.060	634.940	17.827	Blank
SED - wake	Ryde 2013	Office workers	348.000	334.228	361.772	7.026	Blank
SED - wake	Smith 2015	Office workers	636.000	616.716	655.284	9.839	Blank
SED - wake	Swartz 2014	Office workers	376.500	374.501	378.499	1.020	Blank
SED - wake	Tigbe 2011 - O	Office workers	1056.000	1037.142	1074.858	9.621	Blank
SED - wake	Urda 2016	Office workers	356.368	341.273	371.464	7.702	Combined
			560.984	482.149	639.819	40.223	

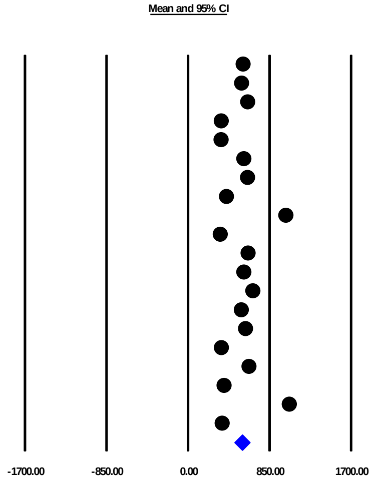

Sedentary time during wake time (min/day)

Supplemental figure 1u. ST, minutes/day during wake time across all occupations except office workers

Outcome	Study name	Comparison	Mean	Lower limit	Upper limit	Standard error	Subgroup within study
				Statistics for each study			
SED - wake	Andersen, 2011	Drivers	504.000	484.461	523.539	9.969	Blank
SED - wake	Arias, 2015	Laborers	760.000	581.081	938.919	91.287	Blank
SED - wake	Chitkara, 2014	Call centre workers	1166.600	687.726	1645.474	244.328	Blank
SED - wake	French 2007	Drivers	342.800	330.648	354.952	6.200	Blank
SED - wake	Miller 2004 - A	Academics	564.000	535.399	592.601	14.592	Scientists
SED - wake	Miller 2004 - BC	Blue-collar workers	390.000	326.886	453.114	32.201	Blank
SED - wake	Miller 2004 - tech	Technicians	468.000	421.326	514.674	23.814	Blank
SED - wake	Pontt 2015 - L	Laborers	492.000	457.060	526.940	17.827	Blank
SED - wake	Reed 2018 a	Healthcare workers	445.000	433.772	456.228	5.729	Nurses
SED - wake	Tigbe 2011 - PD	Postal workers - delivery	960.000	898.713	1021.287	31.270	Blank
SED - wake	Torquati 2018	Healthcare workers	486.300	455.510	517.090	15.710	Nurses
SED - wake	Vareka Mato 2016	Drivers	780.000	749.177	810.823	15.727	Blank
SED - wake	Varela Mato 2017	Drivers	715.000	669.441	760.559	23.245	Blank
SED - wake	Weiler 2015	Athletes	500.600	477.472	523.728	11.800	Blank
SED - wake	Wong 2014	Drivers	468.000	430.238	505.762	19.267	Blank
			565.996	499.163	632.830	34.099	

Mean and 95% CI

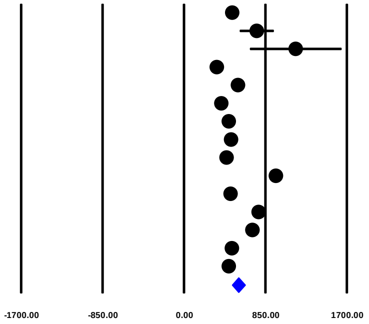

Sedentary time during wake time (min/day)

Supplemental figure 1v. ST, minutes/day during wake time in drivers

Outcome	Study name	Comparison	Mean	Lower limit	Upper limit	Standard error	Subgroup within study
SED - wake	Andersen, 2011	Drivers	504.000	484.461	523.539	9.969	Blank
SED - wake	French 2007	Drivers	342.800	330.648	354.952	6.200	Blank
SED - wake	Vareka Mato 2016	Drivers	780.000	749.177	810.823	15.727	Blank
SED - wake	Varela Mato 2017	Drivers	715.000	669.441	760.559	23.245	Blank
SED - wake	Wong 2014	Drivers	468.000	430.238	505.762	19.267	Blank
			561.441	400.404	722.478	82.163	

Mean and 95% CI

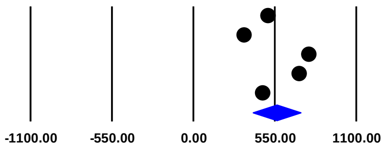

Sedentary time during wake time (min/day)

Supplemental figure 1w. ST, minutes/day during work time across all occupations

Outcome	Study name	Comparison	Mean	Lower limit	Upper limit	Standard error	Subgroup within study
SED - work	Alkajah, 2012	Office workers	347.921	320.953	374.888	13.759	Combined
SED - work	Arundell 2018	Office workers	384.408	379.717	389.099	2.393	Combined
SED - work	Brakenridge, 2016	Office workers	446.000	436.778	455.222	4.705	Blank
SED - work	Chappel, 2016	Protective services	39.800	28.977	50.623	5.522	Blank
SED - work	Chau, 2016	Call centre workers	330.900	272.367	389.432	29.864	Combined
SED - work	Clark 2011a	Call centre workers	404.400	386.877	421.923	8.941	Blank
SED - work	Clark 2011b	Customer service - hospitality	397.800	375.635	419.965	11.309	Blank
SED - work	Clark 2011c	Office workers	396.000	383.403	408.597	6.427	Blank
SED - work	Donath 2015	Office workers with sit-stand desk	346.006	313.770	378.242	16.447	Combined
Sitting - work	Fisher 2018	Office workers	327.600	310.955	344.245	8.492	Blank
SED - work	Gilson 2016	Office workers	371.000	352.568	389.432	9.404	Blank
SED - work	Gorman 2013	Office workers	364.100	346.897	381.303	8.777	Blank
SED - work	Healy 2013	Office workers	337.314	323.789	350.839	6.901	Combined
Sitting - work	Healy 2016	Office workers	378.300	372.394	384.206	3.013	Blank
SED - work	Jirathananuwat 2017	Healthcare workers	494.560	477.177	511.943	8.869	Blank
Sitting - work	Larouche 2018	Office workers	267.900	237.324	298.476	15.600	Blank
SED - work	Li 2017	Office workers	375.932	352.166	399.698	12.126	Combined
SED - work	Lunde 2017 - H	Healthcare workers	171.600	148.438	194.762	11.818	Blank
SED - work	Lunde 2017 - L	Laborers	156.800	128.142	185.458	14.622	Blank
SED - work	Mansoubi 2016	Office workers	299.000	272.659	325.341	13.440	Blank
SED - work	Miller 2004 - A	Academics	294.000	267.442	320.558	13.550	Scientists
SED - work	Miller 2004 - BC	Blue-collar workers	96.000	30.404	161.596	33.468	Blank
SED - work	Miller 2004 - O	Office workers	354.672	336.606	372.738	9.217	Combined
SED - work	Miller 2004 - tech	Technicians	198.000	168.112	227.888	15.249	Blank
SED - work	Neuhaus 2014	Office workers	368.000	354.408	381.592	6.935	Blank
SED - work	Nooijen 2018	Office workers	330.000	320.664	339.336	4.763	Blank
SED - work	Pontt 2015 - L	Laborers	270.000	237.244	302.756	16.713	Blank
SED - work	Pontt 2015 - O	Office workers	384.000	353.428	414.572	15.598	Blank
SED - work	Ryan 2011	Office workers	318.000	305.092	330.908	6.586	Blank
SED - work	Sawyer 2007	Office workers	42.000	40.465	43.535	0.783	Blank
Sitting - work	Stephens 2018	Office workers	381.000	372.269	389.731	4.455	Desk-based workers
SED - work	Umukoro 2013	Healthcare workers	186.860	167.258	206.462	10.001	Nurses and aides
SED - work	Vareka Mato 2016	Drivers	480.000	466.598	493.402	6.838	Blank
SED - work	Varela Mato 2017	Drivers	478.000	438.738	517.262	20.032	Blank
SED - work	Vincent 2016	Protective services	78.000	53.518	102.482	12.491	Blank
Sitting - work	Zhu 2018	Office workers	333.890	312.262	355.518	11.035	Combined
			312.063	242.228	381.898	35.631	

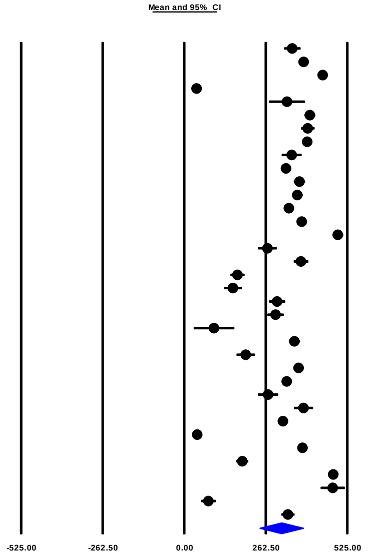

Sedentary time during work time (min/day)

Supplemental figure 1x. Funnel plot for ST (min/day) during wake time in all occupations

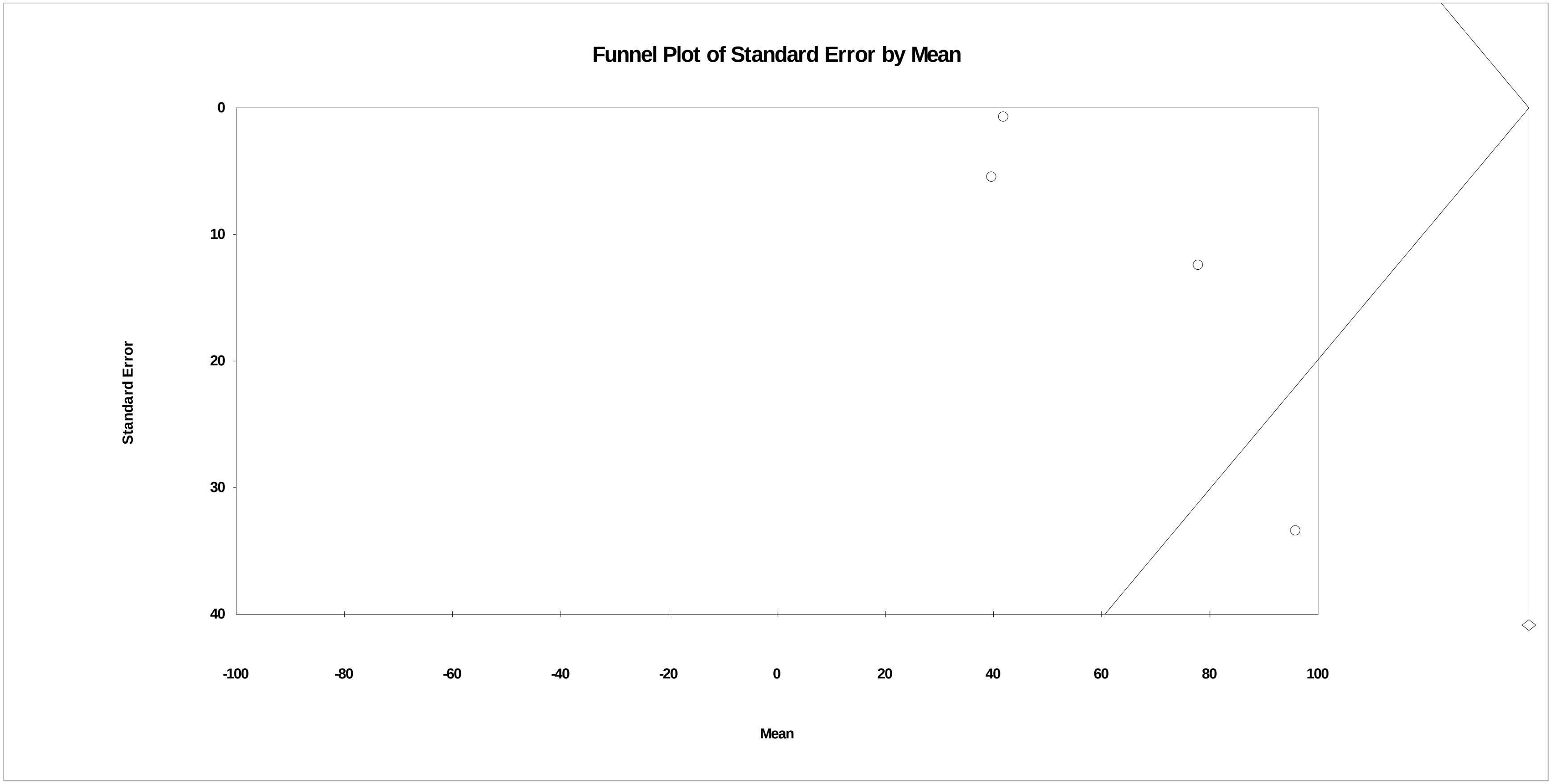

Supplemental figure 1y. ST, minutes/day during work time in office workers

Outcome	Study name	Comparison	Statistics for each study: Mean	Lower limit	Upper limit	Standard error	Subgroup within study
SED - work	Alkajah, 2012	Office workers	347.921	320.953	374.888	13.759	Combined
SED - work	Arundell 2018	Office workers	384.408	379.717	389.099	2.393	Combined
SED - work	Brakenridge, 2016	Office workers	446.000	436.778	455.222	4.705	Blank
SED - work	Clark 2011c	Office workers	396.000	383.403	408.597	6.427	Blank
Sitting - work	Fisher 2018	Office workers	327.600	310.955	344.245	8.492	Blank
SED - work	Gilson 2016	Office workers	371.000	352.568	389.432	9.404	Blank
SED - work	Gorman 2013	Office workers	364.100	346.897	381.303	8.777	Blank
SED - work	Healy 2013	Office workers	337.314	323.789	350.839	6.901	Combined
Sitting - work	Healy 2016	Office workers	378.300	372.394	384.206	3.013	Blank
Sitting - work	Larouche 2018	Office workers	267.900	237.324	298.476	15.600	Blank
SED - work	Li 2017	Office workers	375.932	352.166	399.698	12.126	Combined
SED - work	Mansoubi 2016	Office workers	299.000	272.659	325.341	13.440	Blank
SED - work	Miller 2004 - O	Office workers	354.672	336.606	372.738	9.217	Combined
SED - work	Neuhaus 2014	Office workers	368.000	354.408	381.592	6.935	Blank
SED - work	Nooijen 2018	Office workers	330.000	320.664	339.336	4.763	Blank
SED - work	Pontt 2015 - O	Office workers	384.000	353.428	414.572	15.598	Blank
SED - work	Ryan 2011	Office workers	318.000	305.092	330.908	6.586	Blank
SED - work	Sawyer 2007	Office workers	42.000	40.465	43.535	0.783	Blank
Sitting - work	Stephens 2018	Office workers	381.000	372.269	389.731	4.455	Desk-based workers
Sitting - work	Zhu 2018	Office workers	333.890	312.262	355.518	11.035	Combined
			340.350	244.032	436.667	49.142	

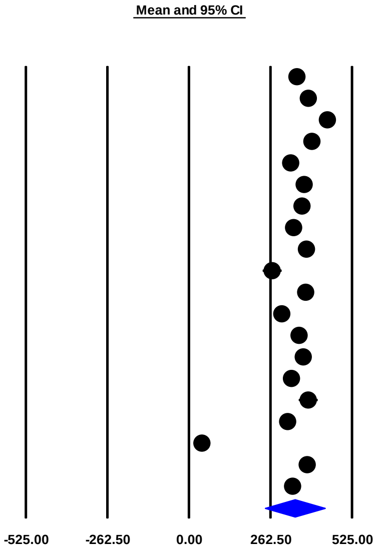

Sedentary time during work time (min/day)

Supplemental figure 1z. ST, minutes/day during work time across all occupations except office workers

Outcome	Study name	Comparison	Mean	Lower limit	Upper limit	Standard error	Subgroup within study
			Statistics for each study				
SED - work	Chappel, 2016	Protective services	39.800	28.977	50.623	5.522	Blank
SED - work	Chau, 2016	Call centre workers	330.900	272.367	389.432	29.864	Combined
SED - work	Clark 2011a	Call centre workers	404.400	386.877	421.923	8.941	Blank
SED - work	Clark 2011b	Customer service - hospitality	397.800	375.635	419.965	11.309	Blank
SED - work	Donath 2015	Office workers with sit-stand desk	346.006	313.770	378.242	16.447	Combined
SED - work	Jirathananuwat 2017	Healthcare workers	494.560	477.177	511.943	8.869	Blank
SED - work	Lunde 2017 - H	Healthcare workers	171.600	148.438	194.762	11.818	Blank
SED - work	Lunde 2017 - L	Laborers	156.800	128.142	185.458	14.622	Blank
SED - work	Miller 2004 - A	Academics	294.000	267.442	320.558	13.550	Scientists
SED - work	Miller 2004 - BC	Blue-collar workers	96.000	30.404	161.596	33.468	Blank
SED - work	Miller 2004 - tech	Technicians	198.000	168.112	227.888	15.249	Blank
SED - work	Pontt 2015 - L	Laborers	270.000	237.244	302.756	16.713	Blank
SED - work	Umukoro 2013	Healthcare workers	186.860	167.258	206.462	10.001	Nurses and aides
SED - work	Vareka Mato 2016	Drivers	480.000	466.598	493.402	6.838	Blank
SED - work	Varela Mato 2017	Drivers	478.000	438.738	517.262	20.032	Blank
SED - work	Vincent 2016	Protective services	78.000	53.518	102.482	12.491	Blank
			276.600	184.040	369.160	47.225	

Mean and 95% CI

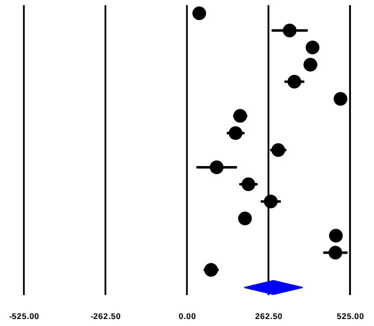

Sedentary time during work time (min/day)

Supplemental figure 1aa. ST, minutes/day during work time across healthcare workers

Outcome	Study name	Comparison	Mean	Lower limit	Upper limit	Standard error	Subgroup within study
SED - work	Jirathananuwat 2017	Healthcare workers	494.560	477.177	511.943	8.869	Blank
SED - work	Lunde 2017 - H	Healthcare workers	171.600	148.438	194.762	11.818	Blank
SED - work	Umukoro 2013	Healthcare workers	186.860	167.258	206.462	10.001	Nurses and aides
			284.420	66.208	502.633	111.335	

Statistics for each study

Mean and 95% CI

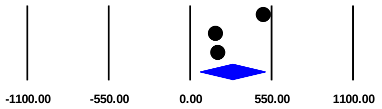

Sedentary time during work time (min/day)

Supplemental figure 1bb. ST, minutes/day during work time in all occupations except healthcare workers

Outcome	Study name	Comparison	Statistics for each study				Subgroup within study
			Mean	Lower limit	Upper limit	Standard error	
SED - work	Alkajah, 2012	Office workers	347.921	320.953	374.888	13.759	Combined
SED - work	Arundell 2018	Office workers	384.408	379.717	389.099	2.393	Combined
SED - work	Brakenridge, 2016	Office workers	446.000	436.778	455.222	4.705	Blank
SED - work	Chappel, 2016	Protective services	39.800	28.977	50.623	5.522	Blank
SED - work	Chau, 2016	Call centre workers	330.900	272.367	389.432	29.864	Combined
SED - work	Clark 2011a	Call centre workers	404.400	386.877	421.923	8.941	Blank
SED - work	Clark 2011b	Customer service - hospitality	397.800	375.635	419.965	11.309	Blank
SED - work	Clark 2011c	Office workers	396.000	383.403	408.597	6.427	Blank
SED - work	Donath 2015	Office workers with sit-stand desk	346.006	313.770	378.242	16.447	Combined
Sitting - work	Fisher 2018	Office workers	327.600	310.955	344.245	8.492	Blank
SED - work	Gilson 2016	Office workers	371.000	352.568	389.432	9.404	Blank
SED - work	Gorman 2013	Office workers	364.100	346.897	381.303	8.777	Blank
SED - work	Healy 2013	Office workers	337.314	323.789	350.839	6.901	Combined
Sitting - work	Healy 2016	Office workers	378.300	372.394	384.206	3.013	Blank
Sitting - work	Larouche 2018	Office workers	267.900	237.324	298.476	15.600	Blank
SED - work	Li 2017	Office workers	375.932	352.166	399.698	12.126	Combined
SED - work	Lunde 2017 - L	Laborers	156.800	128.142	185.458	14.622	Blank
SED - work	Mansoubi 2016	Office workers	299.000	272.659	325.341	13.440	Blank
SED - work	Miller 2004 - A	Academics	294.000	267.442	320.558	13.550	Scientists
SED - work	Miller 2004 - BC	Blue-collar workers	96.000	30.404	161.596	33.468	Blank
SED - work	Miller 2004 - O	Office workers	354.672	336.606	372.738	9.217	Combined
SED - work	Miller 2004 - tech	Technicians	198.000	168.112	227.888	15.249	Blank
SED - work	Neuhaus 2014	Office workers	368.000	354.408	381.592	6.935	Blank
SED - work	Nooijen 2018	Office workers	330.000	320.664	339.336	4.763	Blank
SED - work	Pontt 2015 - L	Laborers	270.000	237.244	302.756	16.713	Blank
SED - work	Pontt 2015 - O	Office workers	384.000	353.428	414.572	15.598	Blank
SED - work	Ryan 2011	Office workers	318.000	305.092	330.908	6.586	Blank
SED - work	Sawyer 2007	Office workers	42.000	40.465	43.535	0.783	Blank
Sitting - work	Stephens 2018	Office workers	381.000	372.269	389.731	4.455	Desk-based workers
SED - work	Vareka Mato 2016	Drivers	480.000	466.598	493.402	6.838	Blank
SED - work	Varela Mato 2017	Drivers	478.000	438.738	517.262	20.032	Blank
SED - work	Vincent 2016	Protective services	78.000	53.518	102.482	12.491	Blank
Sitting - work	Zhu 2018	Office workers	333.890	312.262	355.518	11.035	Combined
			314.580	241.380	387.780	37.348	

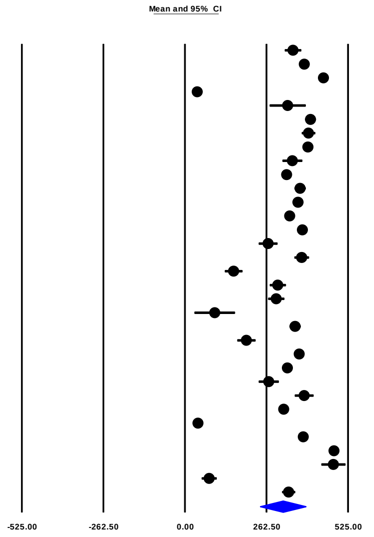

Sedentary time during work time (min/day)

Supplemental figure 1cc. Percentage of non-work days spent in ST, in office workers

Outcome	Study name	Comparison	Statistics for each study				Subgroup within study
			Mean	Lower limit	Upper limit	Standard error	
SED % nonwork day	De Jong 2018	Office workers	66.600	60.215	72.985	3.258	Control
SED % nonwork day	Hadgraft 2016	Office workers	55.900	54.216	57.584	0.859	Blank
SED % weekend day	Kozey-Keadley 2012	Office workers	61.000	53.856	68.144	3.645	Blank
SED % nonwork days	Nooijen 2018	Office workers	57.000	55.111	58.889	0.964	Blank
			58.521	55.462	61.580	1.561	

Mean and 95% CI

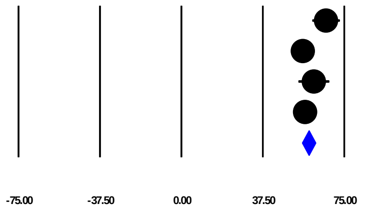

Proportion of non-work day sedentary

Supplemental figure 1dd. ST, minutes/day during non-work days in office workers

Outcome	Studyname	Comparison	Mean	Lower limit	Upper limit	Standard error	Subgroup within study
			Statistics for each study				
SED - nonwork day	De Jong 2018	Office workers	588.000	534.042	641.958	27.530	Control
SED - nonwork days	Nooijen 2018	Office workers	528.000	510.662	545.338	8.846	Blank
SED - nonwork day	Parry 2013 a	Office workers	570.500	546.108	594.892	12.445	Blank
SED - weekend day	Smith 2015	Office workers	636.000	613.043	658.957	11.713	Blank
SED - weekend day	Taylor 2016	Office workers	686.990	583.105	790.875	53.003	Combined
			593.095	541.239	644.951	26.458	

Mean and 95% CI

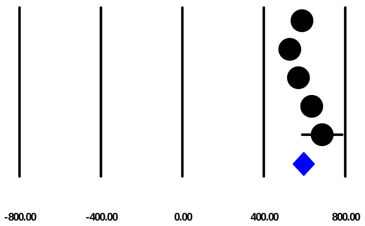

Sedentary time on non-work days (min/day)

Supplemental figures 2a-t

Light intensity physical activity (LPA)

Supplemental figure 2a. Percentage of time in LPA at work across all occupations

Outcome	Study name	Comparison	Mean	Lower limit	Upper limit	Standard error	Subgroup within study
LPA % work	Brewer, 2016	Healthcare workers	21.630	19.982	23.278	0.841	Combined
LPA % work	Mansoubi 2016	Office workers	14.000	12.760	15.240	0.632	Blank
LPA % work	Parry 2013 b	Office workers	19.140	17.211	21.069	0.984	Blank
LPA % work	Thorp 2012 - C	Call centre	4.600	3.611	5.589	0.505	Blank
LPA % work	Thorp 2012 - CS	Customer service	4.800	3.574	6.026	0.626	Blank
LPA % work	Thorp 2012 - O	Office workers	6.500	5.986	7.014	0.262	Blank
LPA % work	Umukoro 2013	Healthcare workers	45.000	41.888	48.112	1.588	Nurses and aides
LPA % work	van Dommelen 2016 - A	Academics	19.509	18.110	20.908	0.714	Combined
LPA % work	van Dommelen 2016 - F	Financial services	17.663	16.507	18.819	0.590	Combined
LPA % work	van Dommelen 2016 - L	Laborers	49.200	44.685	53.715	2.304	Men
LPA % work	Vincent 2016	Protective services	65.600	61.664	69.536	2.008	Blank
			24.068	17.900	30.236	3.147	

Mean and 95% CI

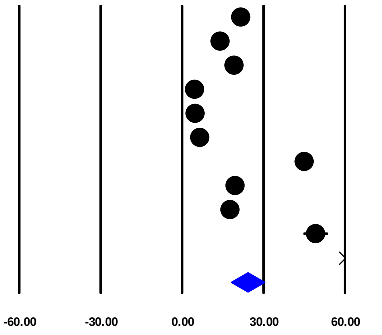

Light intensity physical activity during work time (%)

Supplemental figure 2b. Funnel plot for LPA (%) at work across all occupations

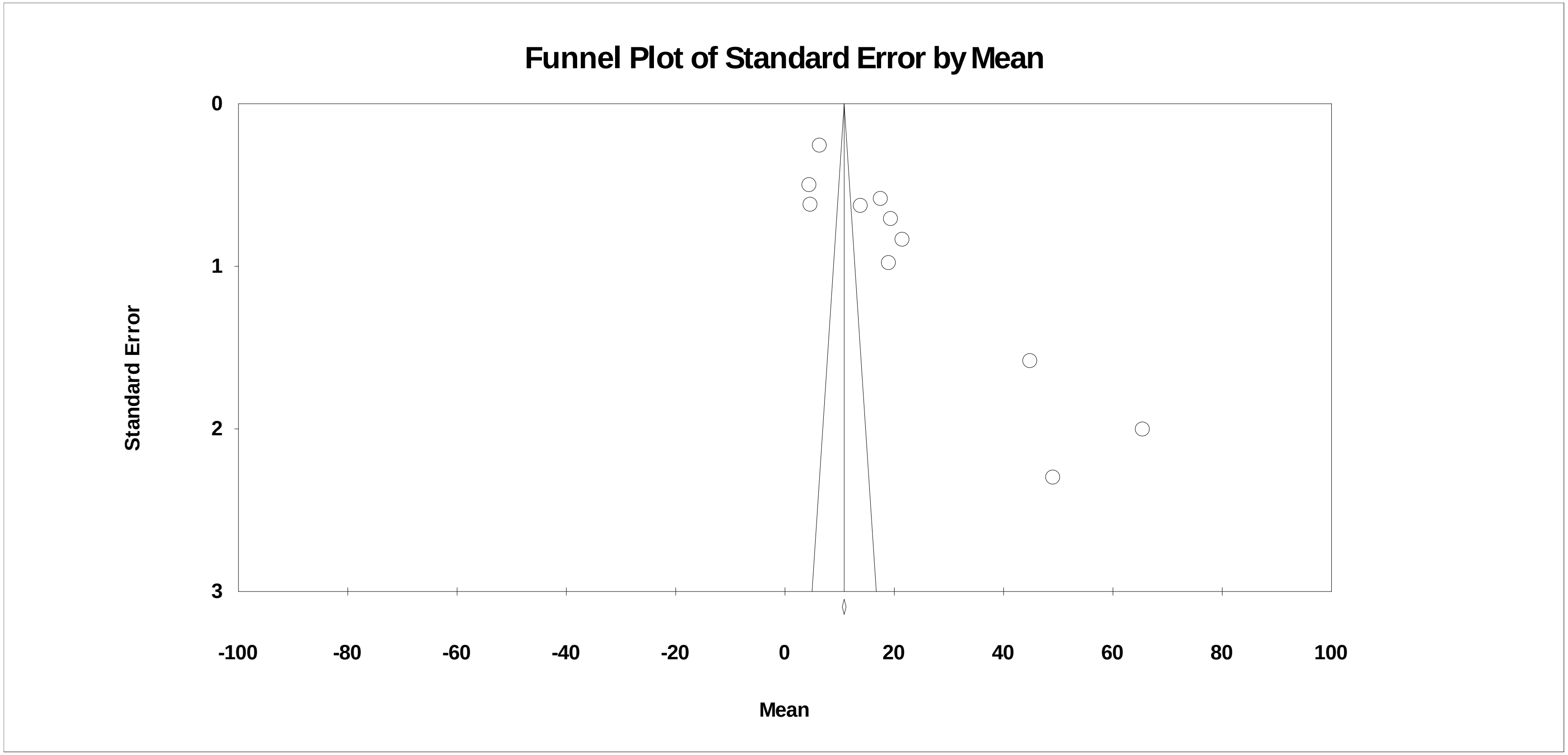

Supplemental figure 2c. Percentage of time in LPA during work in office workers

Outcome	Study name	Comparison	Mean	Lower limit	Upper limit	Standard error	Subgroup within study
LPA % work	Mansoubi 2016	Office workers	14.000	12.760	15.240	0.632	Blank
LPA % work	Parry 2013 b	Office workers	19.140	17.211	21.069	0.984	Blank
LPA % work	Thorp 2012 - O	Office workers	6.500	5.986	7.014	0.262	Blank
			13.169	5.839	20.499	3.740	

Mean and 95% CI

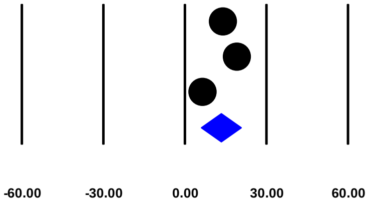

Light intensity physical activity during work time (%)

Supplemental figure 2d. Percentage of time in LPA at work in all occupations except office workers

Outcome	Study name	Comparison	Statistics for each study				Subgroup within study
			Mean	Lower limit	Upper limit	Standard error	
LPA %work	Brewer, 2016	Healthcare workers	21.630	19.982	23.278	0.841	Combined
LPA %work	Thorp 2012 - C	Call centre	4.600	3.611	5.589	0.505	Blank
LPA %work	Thorp 2012 - CS	Customer service	4.800	3.574	6.026	0.626	Blank
LPA %work	Umukoro 2013	Healthcare workers	45.000	41.888	48.112	1.588	Nurses and aides
LPA %work	van Dommelen 2016 - A	Academics	19.509	18.110	20.908	0.714	Combined
LPA %work	van Dommelen 2016 - F	Financial services	17.663	16.507	18.819	0.590	Combined
LPA %work	van Dommelen 2016 - L	Laborers	49.200	44.685	53.715	2.304	Men
LPA %work	Vincent 2016	Protective services	65.600	61.664	69.536	2.008	Blank
			28.319	18.858	37.779	4.827	

Mean and 95% CI

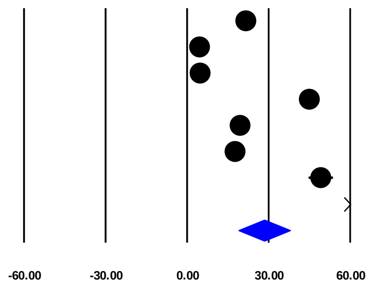

Light intensity physical activity during work time (%)

Supplemental figure 2e. Percentage of time in LPA during wake time across all occupations

Outcome	Study name	Comparison	Statistics for each study				Subgroup within study
			Mean	Lower limit	Upper limit	Standard error	
LPA % wake	Arias, 2015	Laborers	51.000	47.300	54.700	1.888	Blank
LPA % wake	Cuddy, 2015	Protective services	39.000	35.964	42.036	1.549	Blank
LPA % wake	Gao 2016 - O	Office workers	14.900	10.995	18.805	1.992	Blank
LPA % wake	Gao 2016 - OSS	Office workers with sit-stand desk	26.100	19.657	32.543	3.287	Blank
LPA % wake	Gilson 2012b	Office workers	15.000	12.104	17.896	1.477	Blank
LPA % wake	Parry 2013 b	Office workers	23.850	22.264	25.436	0.809	Blank
LPA % wake	Peavler 2012	Office workers	31.160	28.632	33.688	1.290	Combined
LPA % wake	Reed 2018 a	Healthcare workers	45.840	44.995	46.685	0.431	Nurses
LPA % wake	Steeves 2015 - CS	Customer service - hospitality	33.649	33.342	33.956	0.157	Combined
LPA % wake	Steeves 2015 - D	Drivers	32.995	32.737	33.253	0.132	Combined
LPA % wake	Steeves 2015 - EAS	Engineers, architects and scientists	23.000	22.709	23.291	0.149	Blank
LPA % wake	Steeves 2015 - EPP	Extractive and precision production occupations	31.700	31.471	31.929	0.117	Blank
LPA % wake	Steeves 2015 - F	Factory workers	34.965	34.513	35.417	0.231	Combined
LPA % wake	Steeves 2015 - H	Healthcare workers	33.862	33.683	34.041	0.091	Combined
LPA % wake	Steeves 2015 - house	Private household occupations	31.200	27.999	34.401	1.633	Blank
LPA % wake	Steeves 2015 - L	Laborers	32.040	31.917	32.162	0.062	Combined
LPA % wake	Steeves 2015 - Mgt	Management related occupations	26.400	26.176	26.624	0.114	Blank
LPA % wake	Steeves 2015 - MRSD	Material recording, scheduling, distributing clerks	30.200	29.795	30.605	0.206	Blank
LPA % wake	Steeves 2015 - O	Office workers	28.842	28.727	28.957	0.059	Combined
LPA % wake	Steeves 2015 - Other	other professional speciality occupations	26.900	26.555	27.245	0.176	Blank
LPA % wake	Steeves 2015 - personal	Personal service occupations	33.700	32.652	34.748	0.535	Blank
LPA % wake	Steeves 2015 - PS	Protective services	26.500	25.522	27.478	0.499	Protective service occupation
LPA % wake	Steeves 2015 - sales	Sales reps., finance, business, & commodities	27.700	27.227	28.173	0.241	Blank
LPA % wake	Steeves 2015 - ST	School teachers	29.900	29.528	30.272	0.190	Blank
LPA % wake	Steeves 2015 - sups	Supervisors and proprietors, sales occupations	33.100	32.496	33.704	0.308	Blank
LPA % wake	Steeves 2015 - Tech	Technicians and related support occupations	28.600	27.986	29.214	0.313	Blank
LPA % wake	Steeves 2015 - WAEA	Writers, artists, entertainers, and athletes	27.500	26.792	28.208	0.361	Blank
LPA % wake	Torquati 2018	Healthcare workers	38.700	36.270	41.130	1.240	Nurses
LPA % wake	van Dommelen 2016 - A	Academics	31.486	30.257	32.714	0.627	Combined
LPA % wake	van Dommelen 2016 - F	Financial services	26.902	25.669	28.136	0.629	Combined
LPA % wake	van Dommelen 2016 - L	Laborers	40.200	37.529	42.871	1.363	Men
			30.894	29.694	32.094	0.612	

Mean and 95% CI

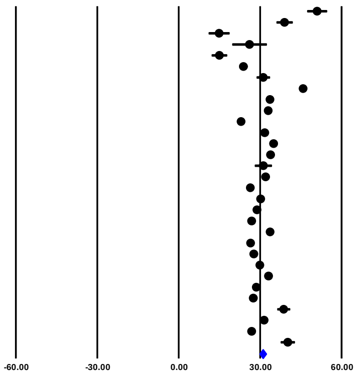

Sedentary time during work time (min/day)

Supplemental figure 2f. Funnel plot for LPA (%) during wake time in all occupations

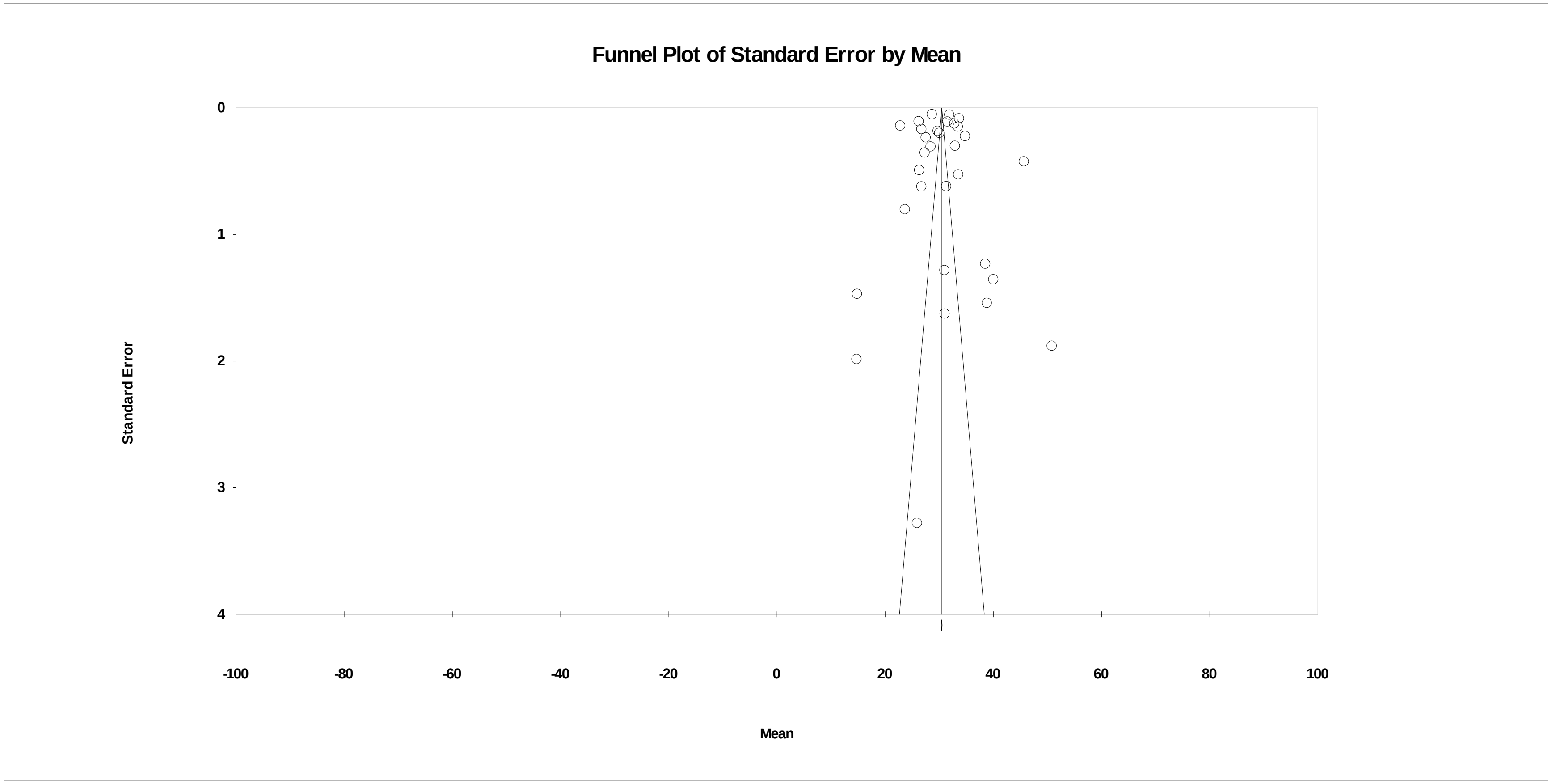

Supplemental figure 2g. Percentage of time in LPA during wake time in office workers

Outcome	Study name	Comparison	Statistics for each study				Subgroup within study
			Mean	Lower limit	Upper limit	Standard error	
LPA % wake	Gao 2016 - O	Office workers	14.900	10.995	18.805	1.992	Blank
LPA % wake	Gilson 2012b	Office workers	15.000	12.104	17.896	1.477	Blank
LPA % wake	Parry 2013 b	Office workers	23.850	22.264	25.436	0.809	Blank
LPA % wake	Peavler 2012	Office workers	31.160	28.632	33.688	1.290	Combined
LPA % wake	Steeves 2015 - O	Office workers	28.842	28.727	28.957	0.059	Combined
			22.952	17.968	27.937	2.543	

Mean and 95% CI

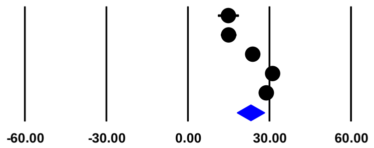

Light intensity physical activity during wake time (%)

Supplemental figure 2h. Percentage of time in LPA during wake time in all occupations except office workers

Outcome	Study name	Comparison	Mean	Lower limit	Upper limit	Standard error	Subgroup within study
LPA % wake	Arias, 2015	Laborers	51.000	47.300	54.700	1.888	Blank
LPA % wake	Cuddy, 2015	Protective services	39.000	35.964	42.036	1.549	Blank
LPA % wake	Gao 2016 - OSS	Office workers with sit-stand desk	26.100	19.657	32.543	3.287	Blank
LPA % wake	Reed 2018 a	Healthcare workers	45.840	44.995	46.685	0.431	Nurses
LPA % wake	Steeves 2015 - CS	Customer service - hospitality	33.649	33.342	33.956	0.157	Combined
LPA % wake	Steeves 2015 - D	Drivers	32.995	32.737	33.253	0.132	Combined
LPA % wake	Steeves 2015 - EAS	Engineers, architects and scientists	23.000	22.709	23.291	0.149	Blank
LPA % wake	Steeves 2015 - EPP	Extractive and precision production occupations	31.700	31.471	31.929	0.117	Blank
LPA % wake	Steeves 2015 - F	Factory workers	34.965	34.513	35.417	0.231	Combined
LPA % wake	Steeves 2015 - H	Healthcare workers	33.862	33.683	34.041	0.091	Combined
LPA % wake	Steeves 2015 - house	Private household occupations	31.200	27.999	34.401	1.633	Blank
LPA % wake	Steeves 2015 - L	Laborers	32.040	31.917	32.162	0.062	Combined
LPA % wake	Steeves 2015 - Mgt	Management related occupations	26.400	26.176	26.624	0.114	Blank
LPA % wake	Steeves 2015 - MRSD	Material recording, scheduling, distributing clerks	30.200	29.795	30.605	0.206	Blank
LPA % wake	Steeves 2015 - Other	other professional speciality occupations	26.900	26.555	27.245	0.176	Blank
LPA % wake	Steeves 2015 - personal	Personal service occupations	33.700	32.652	34.748	0.535	Blank
LPA % wake	Steeves 2015 - PS	Protective services	26.500	25.522	27.478	0.499	Protective service occupation
LPA % wake	Steeves 2015 - sales	Sales reps., finance, business, & commodities	27.700	27.227	28.173	0.241	Blank
LPA % wake	Steeves 2015 - ST	School teachers	29.900	29.528	30.272	0.190	Blank
LPA % wake	Steeves 2015 - sups	Supervisors and proprietors, sales occupations	33.100	32.496	33.704	0.308	Blank
LPA % wake	Steeves 2015 - Tech	Technicians and related support occupations	28.600	27.986	29.214	0.313	Blank
LPA % wake	Steeves 2015 - WAEA	Writers, artists, entertainers, and athletes	27.500	26.792	28.208	0.361	Blank
LPA % wake	Torquati 2018	Healthcare workers	38.700	36.270	41.130	1.240	Nurses
LPA % wake	van Dommelen 2016 - A	Academics	31.486	30.257	32.714	0.627	Combined
LPA % wake	van Dommelen 2016 - F	Financial services	26.902	25.669	28.136	0.629	Combined
LPA % wake	van Dommelen 2016 - L	Laborers	40.200	37.529	42.871	1.363	Men
			32.289	30.878	33.700	0.720	

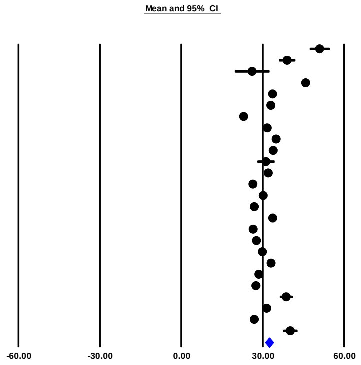

Light intensity physical activity during wake time (%)

Supplemental figure 2i. Percentage of time in LPA during wake time in healthcare workers

Outcome	Study name	Comparison	Statistics for each study				Subgroup within study
			Mean	Lower limit	Upper limit	Standard error	
LPA % wake	Reed 2018 a	Healthcare workers	45.840	44.995	46.685	0.431	Nurses
LPA % wake	Steeves 2015 - H	Healthcare workers	33.862	33.683	34.041	0.091	Combined
LPA % wake	Torquati 2018	Healthcare workers	38.700	36.270	41.130	1.240	Nurses
			39.468	30.312	48.623	4.671	

Mean and 95% CI

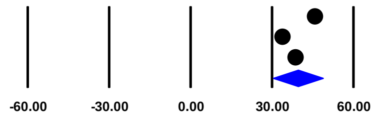

Sedentary time during work time (min/day)

Supplemental figure 2j. Percentage of time in LPA during wake time in all occupations except healthcare workers

Outcome	Study name	Comparison	Statistics for each study: Mean	Lower limit	Upper limit	Standard error	Subgroup within study
LPA % wake	Arias, 2015	Laborers	51.000	47.300	54.700	1.888	Blank
LPA % wake	Cuddy, 2015	Protective services	39.000	35.964	42.036	1.549	Blank
LPA % wake	Gao 2016 - O	Office workers	14.900	10.995	18.805	1.992	Blank
LPA % wake	Gao 2016 - OSS	Office workers with sit-stand desk	26.100	19.657	32.543	3.287	Blank
LPA % wake	Gilson 2012b	Office workers	15.000	12.104	17.896	1.477	Blank
LPA % wake	Parry 2013 b	Office workers	23.850	22.264	25.436	0.809	Blank
LPA % wake	Peavler 2012	Office workers	31.160	28.632	33.688	1.290	Combined
LPA % wake	Steeves 2015 - CS	Customer service - hospitality	33.649	33.342	33.956	0.157	Combined
LPA % wake	Steeves 2015 - D	Drivers	32.995	32.737	33.253	0.132	Combined
LPA % wake	Steeves 2015 - EAS	Engineers, architects and scientists	23.000	22.709	23.291	0.149	Blank
LPA % wake	Steeves 2015 - EPP	Extractive and precision production occupations	31.700	31.471	31.929	0.117	Blank
LPA % wake	Steeves 2015 - F	Factory workers	34.965	34.513	35.417	0.231	Combined
LPA % wake	Steeves 2015 - house	Private household occupations	31.200	27.999	34.401	1.633	Blank
LPA % wake	Steeves 2015 - L	Laborers	32.040	31.917	32.162	0.062	Combined
LPA % wake	Steeves 2015 - Mgt	Management related occupations	26.400	26.176	26.624	0.114	Blank
LPA % wake	Steeves 2015 - MRSD	Material recording, scheduling, distributing clerks	30.200	29.795	30.605	0.206	Blank
LPA % wake	Steeves 2015 - O	Office workers	28.842	28.727	28.957	0.059	Combined
LPA % wake	Steeves 2015 - Other	other professional speciality occupations	26.900	26.555	27.245	0.176	Blank
LPA % wake	Steeves 2015 - personal	Personal service occupations	33.700	32.652	34.748	0.535	Blank
LPA % wake	Steeves 2015 - PS	Protective services	26.500	25.522	27.478	0.499	Protective service occupation
LPA % wake	Steeves 2015 - sales	Sales reps., finance, business, & commodities	27.700	27.227	28.173	0.241	Blank
LPA % wake	Steeves 2015 - ST	School teachers	29.900	29.528	30.272	0.190	Blank
LPA % wake	Steeves 2015 - sups	Supervisors and proprietors, sales occupations	33.100	32.496	33.704	0.308	Blank
LPA % wake	Steeves 2015 - Tech	Technicians and related support occupations	28.600	27.986	29.214	0.313	Blank
LPA % wake	Steeves 2015 - WAEA	Writers, artists, entertainers, and athletes	27.500	26.792	28.208	0.361	Blank
LPA % wake	van Dommelen 2016 - A	Academics	31.486	30.257	32.714	0.627	Combined
LPA % wake	van Dommelen 2016 - F	Financial services	26.902	25.669	28.136	0.629	Combined
LPA % wake	van Dommelen 2016 - L	Laborers	40.200	37.529	42.871	1.363	Men
			29.945	28.784	31.106	0.592	

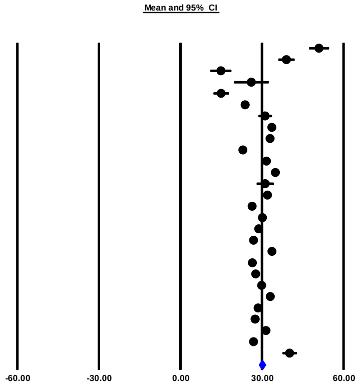

Light intensity physical activity during wake time (%)

Supplemental figure 2k. Percentage of time in LPA during wake time in laborers

Outcome	Study name	Comparison	Mean	Lower limit	Upper limit	Standard error	Subgroup within study
LPA % wake	Arias, 2015	Laborers	51.000	47.300	54.700	1.888	Blank
LPA % wake	Steeves 2015 - L	Laborers	32.040	31.917	32.162	0.062	Combined
LPA % wake	van Dommelen 2016 - L	Laborers	40.200	37.529	42.871	1.363	Men
			40.946	30.575	51.318	5.292	

(Column group heading over Mean, Lower limit, Upper limit, Standard error: Statistics for each study)

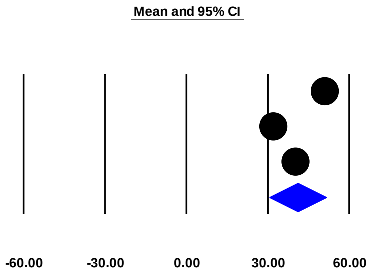

Light intensity physical activity during wake time (%)

Supplemental figure 2l. Percentage of time in LPA during wake time in all occupations except laborers

Outcome	Study name	Comparison	Mean	Lower limit	Upper limit	Standard error	Subgroup within study
LPA % wake	Cuddy, 2015	Protective services	39.000	35.964	42.036	1.549	Blank
LPA % wake	Gao 2016 - O	Office workers	14.900	10.995	18.805	1.992	Blank
LPA % wake	Gao 2016 - OSS	Office workers with sit-stand desk	26.100	19.657	32.543	3.287	Blank
LPA % wake	Gilson 2012b	Office workers	15.000	12.104	17.896	1.477	Blank
LPA % wake	Parry 2013 b	Office workers	23.850	22.264	25.436	0.809	Blank
LPA % wake	Peavler 2012	Office workers	31.160	28.632	33.688	1.290	Combined
LPA % wake	Reed 2018 a	Healthcare workers	45.840	44.995	46.685	0.431	Nurses
LPA % wake	Steeves 2015 - CS	Customer service - hospitality	33.649	33.342	33.956	0.157	Combined
LPA % wake	Steeves 2015 - D	Drivers	32.995	32.737	33.253	0.132	Combined
LPA % wake	Steeves 2015 - EAS	Engineers, architects and scientists	23.000	22.709	23.291	0.149	Blank
LPA % wake	Steeves 2015 - EPP	Extractive and precision production occupations	31.700	31.471	31.929	0.117	Blank
LPA % wake	Steeves 2015 - F	Factory workers	34.965	34.513	35.417	0.231	Combined
LPA % wake	Steeves 2015 - H	Healthcare workers	33.862	33.683	34.041	0.091	Combined
LPA % wake	Steeves 2015 - house	Private household occupations	31.200	27.999	34.401	1.633	Blank
LPA % wake	Steeves 2015 - Mgt	Management related occupations	26.400	26.176	26.624	0.114	Blank
LPA % wake	Steeves 2015 - MRSD	Material recording, scheduling, distributing clerks	30.200	29.795	30.605	0.206	Blank
LPA % wake	Steeves 2015 - O	Office workers	28.842	28.727	28.957	0.059	Combined
LPA % wake	Steeves 2015 - Other	other professional speciality occupations	26.900	26.555	27.245	0.176	Blank
LPA % wake	Steeves 2015 - personal	Personal service occupations	33.700	32.652	34.748	0.535	Blank
LPA % wake	Steeves 2015 - PS	Protective services	26.500	25.522	27.478	0.499	Protective service occupation
LPA % wake	Steeves 2015 - sales	Sales reps., finance, business, & commodities	27.700	27.227	28.173	0.241	Blank
LPA % wake	Steeves 2015 - ST	School teachers	29.900	29.528	30.272	0.190	Blank
LPA % wake	Steeves 2015 - sups	Supervisors and proprietors, sales occupations	33.100	32.496	33.704	0.308	Blank
LPA % wake	Steeves 2015 - Tech	Technicians and related support occupations	28.600	27.986	29.214	0.313	Blank
LPA % wake	Steeves 2015 - WAEA	Writers, artists, entertainers, and athletes	27.500	26.792	28.208	0.361	Blank
LPA % wake	Torquati 2018	Healthcare workers	38.700	36.270	41.130	1.240	Nurses
LPA % wake	van Dommelen 2016 - A	Academics	31.486	30.257	32.714	0.627	Combined
LPA % wake	van Dommelen 2016 - F	Financial services	26.902	25.669	28.136	0.629	Combined
			29.947	28.581	31.314	0.697	

Mean and 95% CI

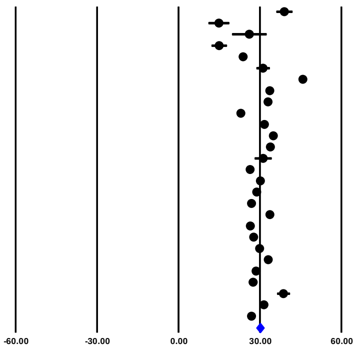

Sedentary time during work time (min/day)

Supplemental figure 2m. LPA, minutes/day at work across all occupations

Outcome	Study name	Comparison	Statistics for each study				Subgroup within study
			Mean	Lower limit	Upper limit	Standard error	
LPA - work	Arundell 2018	Office workers	75.706	71.832	79.579	1.976	Combined
LPA - work	Chappel, 2016	Protective services	430.100	392.588	467.612	19.139	Blank
LPA - work	Chau, 2016	Call centre workers	20.000	9.816	30.184	5.196	Blank
LPA - work	Jirathananuwat 2017	Healthcare workers	80.800	76.050	85.550	2.424	Blank
LPA - work	Larouche 2018	Office workers	35.900	28.930	42.870	3.556	Blank
LPA - work	Mansoubi 2016	Office workers	53.000	47.422	58.578	2.846	Blank
LPA - work	Umukoro 2013	Healthcare workers	154.857	139.257	170.457	7.959	Nurses and aides
LPA - work	Vincent 2016	Protective services	453.000	406.515	499.485	23.717	Blank
LPA - work	Zhu 2018	Office workers	27.207	22.961	31.453	2.166	Combined
			133.841	105.707	161.975	14.354	

Mean and 95% CI

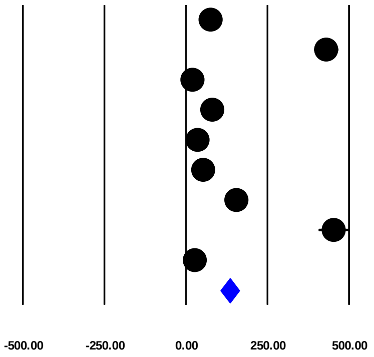

Light intensity physical activity during work time (min/day)

Supplemental figure 2n. Funnel plot for LPA min/day at work across all occupations

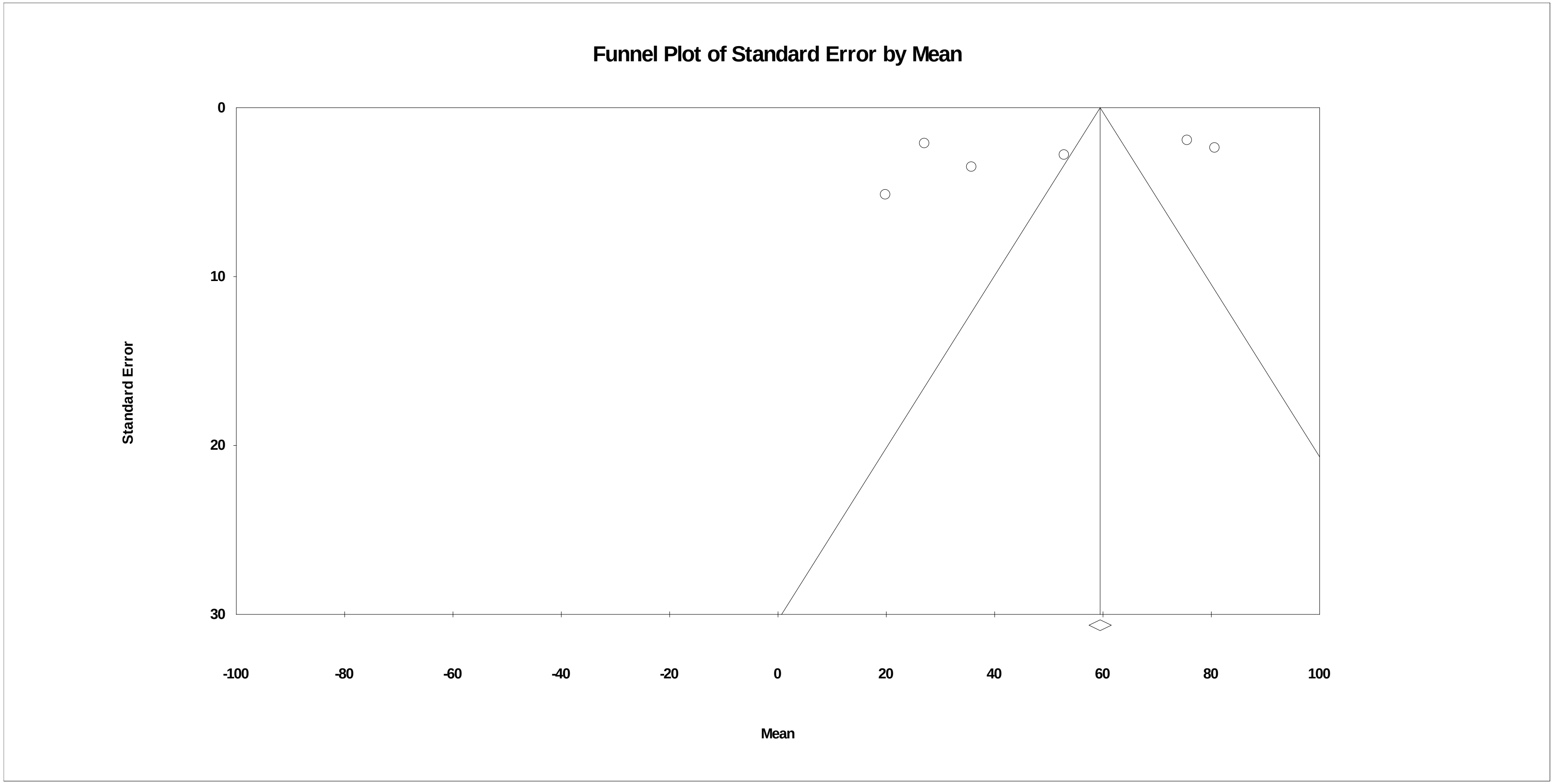

Supplemental figure 2o. LPA, minutes/day at work across office workers

Outcome	Study name	Comparison	Statistics for each study: Mean	Lower limit	Upper limit	Standard error	Subgroup within study
LPA - work	Arundell 2018	Office workers	75.706	71.832	79.579	1.976	Combined
LPA - work	Larouche 2018	Office workers	35.900	28.930	42.870	3.556	Blank
LPA - work	Mansoubi 2016	Office workers	53.000	47.422	58.578	2.846	Blank
LPA - work	Zhu 2018	Office workers	27.207	22.961	31.453	2.166	Combined
			47.993	23.526	72.461	12.484	

Mean and 95% CI

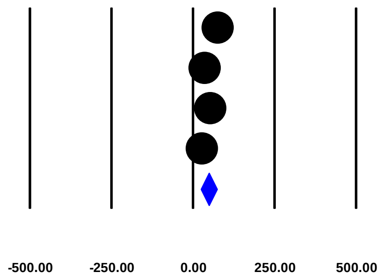

Light intensity physical activity during work time (min/day)

Supplemental figure 2p. LPA, minutes/day at work across all occupations except office workers

Outcome	Study name	Comparison	Statistics for each study				Subgroup within study
			Mean	Lower limit	Upper limit	Standard error	
LPA - work	Chappel, 2016	Protective services	430.100	392.588	467.612	19.139	Blank
LPA - work	Chau, 2016	Call centre workers	20.000	9.816	30.184	5.196	Blank
LPA - work	Jirathananuwat 2017	Healthcare workers	80.800	76.050	85.550	2.424	Blank
LPA - work	Umukoro 2013	Healthcare workers	154.857	139.257	170.457	7.959	Nurses and aides
LPA - work	Vincent 2016	Protective services	453.000	406.515	499.485	23.717	Blank
			223.479	141.388	305.570	41.884	

Mean and 95% CI

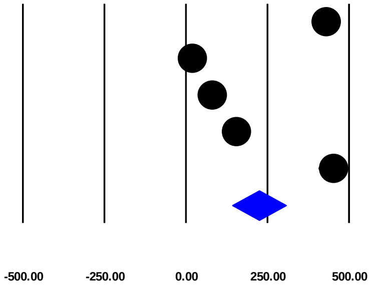

Light intensity physical activity during work time (min/day)

Supplemental figure 2q. LPA, minutes/day during wake time across all occupations

Outcome	Study name	Comparison	Mean	Lower limit	Upper limit	Standard error	Subgroup within study
LPA - wake	Arias, 2015	Laborers	1055.000	948.230	1161.770	54.475	Blank
LPA - wake	Bergman 2018	Office workers	342.560	327.891	357.229	7.484	Combined
LPA - wake	Chitkara, 2014	Call centre workers	181.200	146.622	215.778	17.642	Blank
LPA - wake	French 2007	Drivers	232.500	222.112	242.888	5.300	Blank
LPA - wake	Gilson 2016	Office workers	300.000	273.520	326.480	13.510	Blank
LPA - wake	Jancey 2016	Office workers	35.130	31.401	38.859	1.903	Blank
LPA - wake	Parry 2013 a	Office workers	176.900	162.320	191.480	7.439	Blank
LPA - wake	Peavler 2012	Office workers	264.505	241.360	287.650	11.809	Combined
LPA - wake	Reed 2018 a	Healthcare workers	408.000	400.353	415.647	3.902	Nurses
LPA - wake	Torquati 2018	Healthcare workers	322.400	299.700	345.100	11.582	Nurses
LPA - wake	Weiler 2015	Athletes	55.700	44.411	66.989	5.760	Blank
LPA - wake	Wong 2014	Drivers	396.000	364.123	427.877	16.264	Blank
			309.295	204.100	414.489	53.672	

Mean and 95% CI

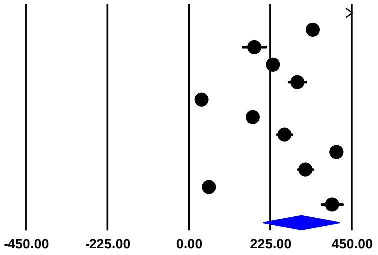

Light intensity physical activity during wake time (min/day)

Supplemental figure 2r. Funnel plot for LPA min/day during wake time across all occupations

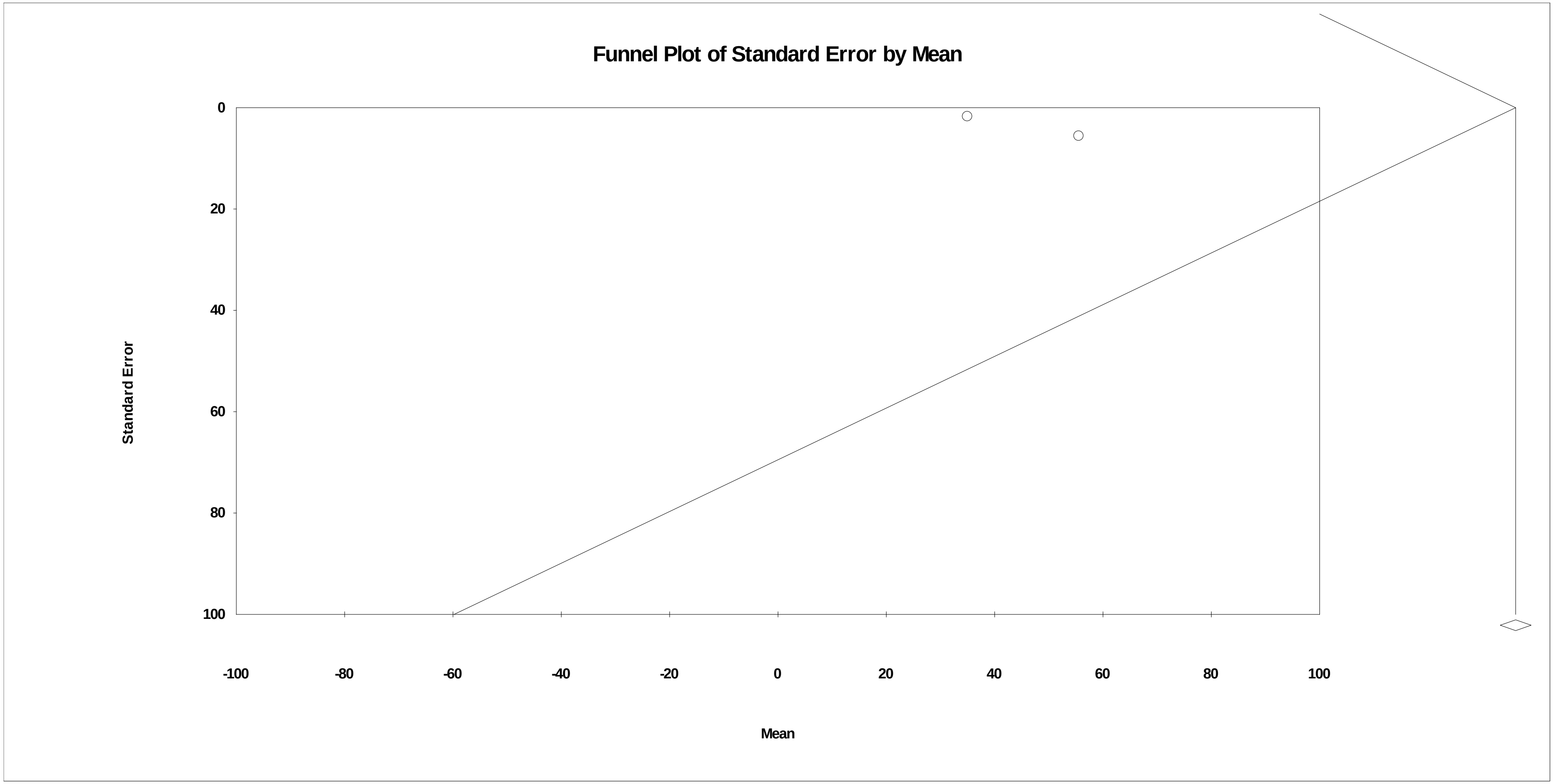

Supplemental figure 2s. LPA, minutes/day during wake time in office workers

Outcome	Study name	Comparison	Statistics for each study				Subgroup within study
			Mean	Lower limit	Upper limit	Standard error	
LPA - wake	Bergman 2018	Office workers	342.560	327.891	357.229	7.484	Combined
LPA - wake	Gilson 2016	Office workers	300.000	273.520	326.480	13.510	Blank
LPA - wake	Jancey 2016	Office workers	35.130	31.401	38.859	1.903	Blank
LPA - wake	Parry 2013 a	Office workers	176.900	162.320	191.480	7.439	Blank
LPA - wake	Peavler 2012	Office workers	264.505	241.360	287.650	11.809	Combined
			223.654	77.605	369.702	74.516	

Mean and 95% CI

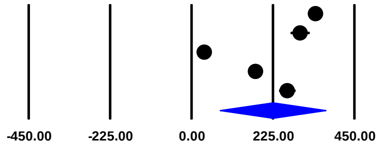

Light intensity physical activity during wake time (min/day)

Supplemental figure 2t. LPA, minutes/day during wake time in all workers except office workers

Outcome	Study name	Comparison	Statistics for each study				Subgroup within study
			Mean	Lower limit	Upper limit	Standard error	
LPA - wake	Arias, 2015	Laborers	1055.000	948.230	1161.770	54.475	Blank
LPA - wake	Chitkara, 2014	Call centre workers	181.200	146.622	215.778	17.642	Blank
LPA - wake	French 2007	Drivers	232.500	222.112	242.888	5.300	Blank
LPA - wake	Reed 2018 a	Healthcare workers	408.000	400.353	415.647	3.902	Nurses
LPA - wake	Torquati 2018	Healthcare workers	322.400	299.700	345.100	11.582	Nurses
LPA - wake	Weiler 2015	Athletes	55.700	44.411	66.989	5.760	Blank
LPA - wake	Wong 2014	Drivers	396.000	364.123	427.877	16.264	Blank
			370.021	242.239	497.802	65.196	

Mean and 95% CI

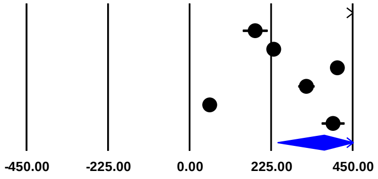

Light intensity physical activity during wake time (min/day)

Supplemental figures 3a-j

Moderate-to-vigorous intensity physical activity (MVPA)

Supplemental figure 3a. Percentage of time in MVPA at work across all occupations

Outcome	Study name	Comparison	Mean	Lower limit	Upper limit	Standard error	Subgroup within study
MVPA % work	Brewer, 2016	Healthcare workers	6.019	5.225	6.814	0.405	Combined
MVPA % work	Mansoubi 2016	Office workers	16.000	13.521	18.479	1.265	Blank
MVPA % work	Parry 2013 b	Office workers	2.570	2.114	3.026	0.232	Blank
MVPA % work	Thorp 2012 - C	Call centre	1.800	1.186	2.414	0.313	Blank
MVPA % work	Thorp 2012 - CS	Customer service	1.900	1.532	2.268	0.188	Blank
MVPA % work	Thorp 2012 - O	Office workers	3.500	3.123	3.877	0.192	Blank
MVPA % work	van Dommelen 2016 - A	Academics	3.552	3.081	4.022	0.240	Combined
MVPA % work	van Dommelen 2016 - F	Financial services	3.341	2.888	3.794	0.231	Combined
MVPA % work	van Dommelen 2016 - L	Laborers	7.200	5.547	8.853	0.844	Men
			4.517	3.482	5.552	0.528	

Mean and 95% CI

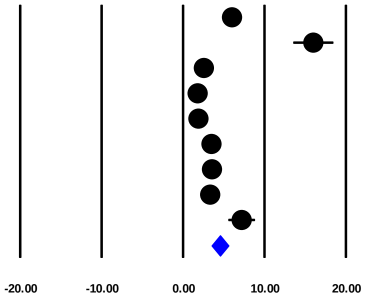

MVPA during work time (%)

Supplemental figure 3b. Funnel plot for MVPA (%) at work across all occupations

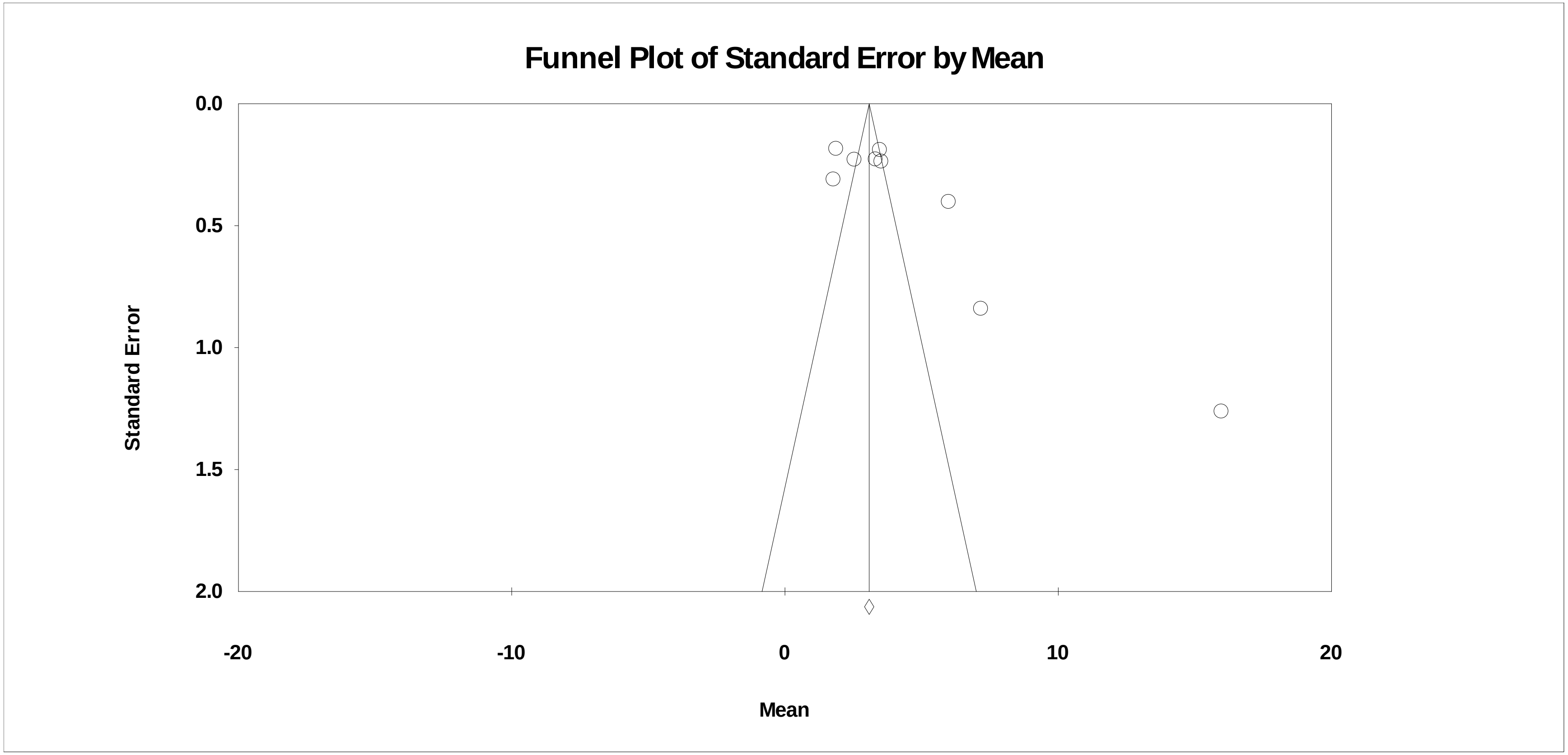

Supplemental figure 3c. Percentage of time in MVPA during wake time across all occupations

Outcome	Study name	Comparison	Mean	Lower limit	Upper limit	Standard error	Subgroup within study
			Statistics for each study				
MVPA % wake	Cuddy, 2015	Protective services	12.000	10.988	13.012	0.516	Blank
MVPA % wake	Gao 2016 - O	Office workers	4.200	2.898	5.502	0.664	Blank
MVPA % wake	Gao 2016 - OSS	Office workers with sit-stand desk	7.700	3.824	11.576	1.978	Blank
MVPA % wake	Gilson 2012b	Office workers	9.200	5.182	13.218	2.050	Blank
MVPA % wake	Parry 2013 b	Office workers	3.290	2.834	3.746	0.232	Blank
MVPA % wake	Reed 2018 a	Healthcare workers	4.640	4.416	4.864	0.114	Nurses
MVPA % wake	Schwartz 2016	Office workers	11.500	10.585	12.415	0.467	Blank
MVPA % wake	Steeves 2015 - CS	Customer service - hospitality	2.674	2.590	2.759	0.043	Combined
MVPA % wake	Steeves 2015 - D	Drivers	4.149	4.059	4.239	0.046	Combined
MVPA % wake	Steeves 2015 - EAS	Engineers, architects and scientists	4.000	3.745	4.255	0.130	Blank
MVPA % wake	Steeves 2015 - EPP	Extractive and precision production occupations	3.100	3.014	3.186	0.044	Blank
MVPA % wake	Steeves 2015 - F	Factory workers	3.532	3.385	3.680	0.075	Combined
MVPA % wake	Steeves 2015 - H	Healthcare workers	2.498	2.424	2.571	0.038	Combined
MVPA % wake	Steeves 2015 - house	Private household occupations	4.300	3.320	5.280	0.500	Blank
MVPA % wake	Steeves 2015 - L	Laborers	4.943	4.875	5.011	0.035	Combined
MVPA % wake	Steeves 2015 - Mgt	Management related occupations	3.500	3.388	3.612	0.057	Blank
MVPA % wake	Steeves 2015 - MRSD	Material recording, scheduling, distributing clerks	3.300	2.850	3.750	0.229	Blank
MVPA % wake	Steeves 2015 - O	Office workers	2.855	2.826	2.884	0.015	Combined
MVPA % wake	Steeves 2015 - Other	other professional speciality occupations	2.900	2.806	2.994	0.048	Blank
MVPA % wake	Steeves 2015 - personal	Personal service occupations	1.600	1.390	1.810	0.107	Blank
MVPA % wake	Steeves 2015 - PS	Protective services	3.400	2.856	3.944	0.277	Protective service occupation
MVPA % wake	Steeves 2015 - sales	Sales reps., finance, business, & commodities	3.500	3.282	3.718	0.111	Blank
MVPA % wake	Steeves 2015 - ST	School teachers	3.000	2.845	3.155	0.079	Blank
MVPA % wake	Steeves 2015 - sups	Supervisors and proprietors, sales occupations	3.100	2.911	3.289	0.096	Blank
MVPA % wake	Steeves 2015 - Tech	Technicians and related support occupations	3.600	3.464	3.736	0.070	Blank
MVPA % wake	Steeves 2015 - WAEA	Writers, artists, entertainers, and athletes	3.500	3.146	3.854	0.181	Blank
MVPA % wake	van Dommelen 2016 - A	Academics	3.899	3.460	4.338	0.224	Combined
MVPA % wake	van Dommelen 2016 - F	Financial services	3.788	3.495	4.081	0.149	Combined
MVPA % wake	van Dommelen 2016 - L	Laborers	4.300	3.537	5.063	0.389	Men
			4.025	3.692	4.357	0.170	

Mean and 95% CI

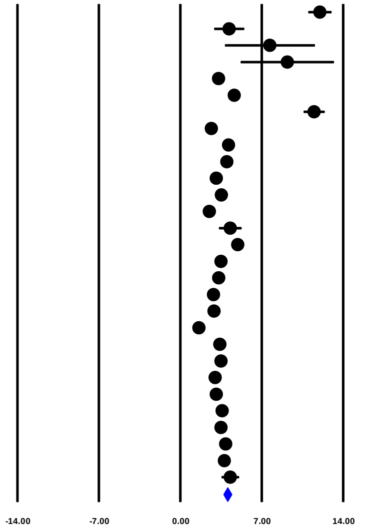

MVPA during wake time (%)

Supplemental figure 3d. Funnel plot for MVPA (%) during wake time across all occupations

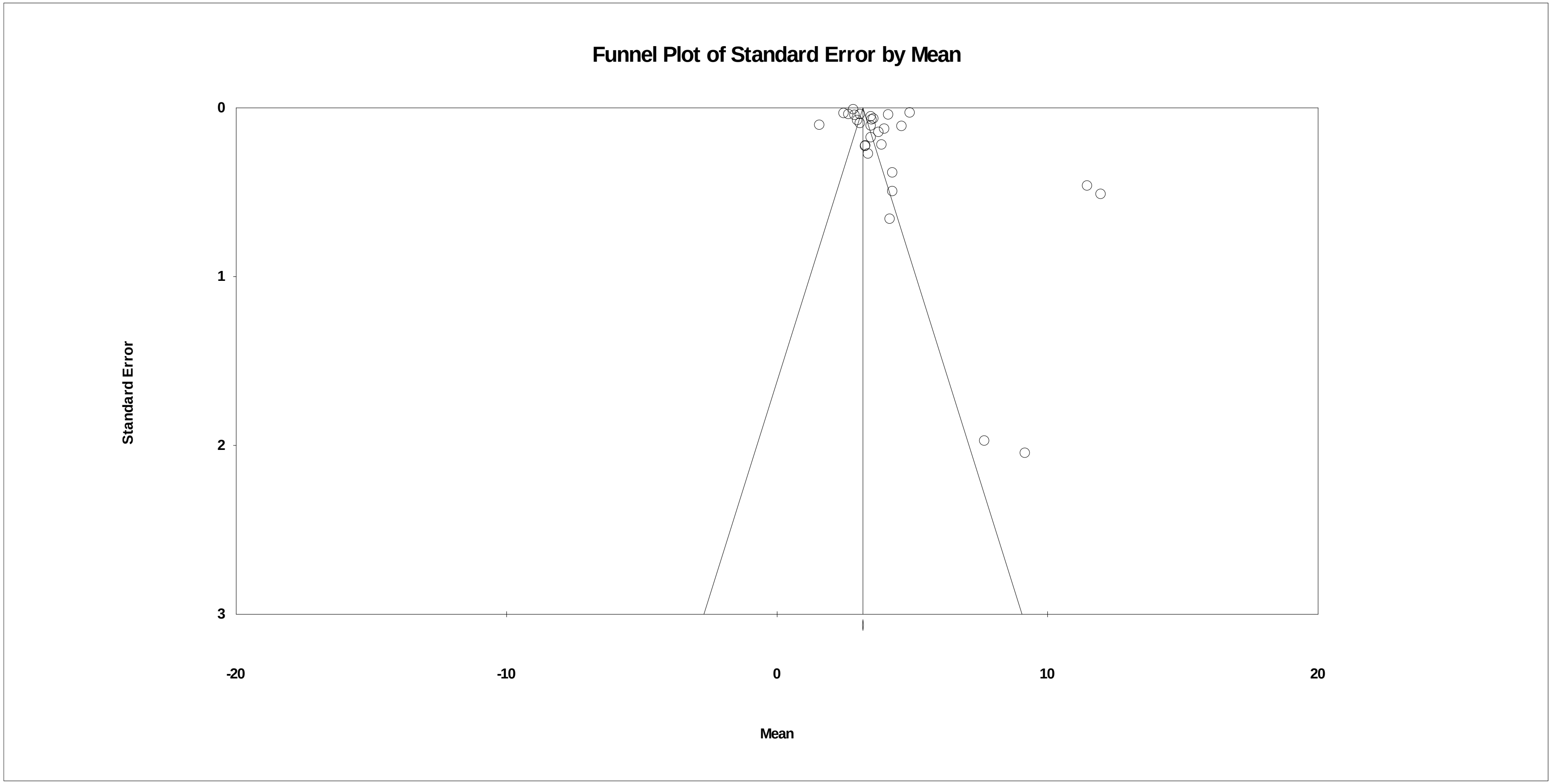

Supplemental figure 3e. Percentage of time in MVPA during wake time across all office workers

Outcome	Study name	Comparison	Statistics for each study				Subgroup within study
			Mean	Lower limit	Upper limit	Standard error	
MVPA % wake	Gao 2016 - O	Office workers	4.200	2.898	5.502	0.664	Blank
MVPA % wake	Gilson 2012b	Office workers	9.200	5.182	13.218	2.050	Blank
MVPA % wake	Parry 2013 b	Office workers	3.290	2.834	3.746	0.232	Blank
MVPA % wake	Schwartz 2016	Office workers	11.500	10.585	12.415	0.467	Blank
MVPA % wake	Steeves 2015 - O	Office workers	2.855	2.826	2.884	0.015	Combined
			5.956	3.525	8.388	1.241	

Mean and 95% CI

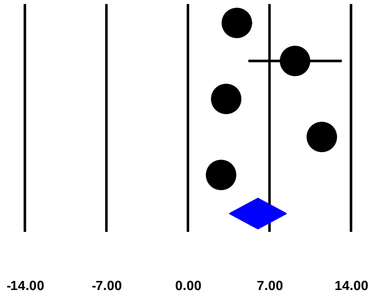

MVPA during wake time (%)

Supplemental figure 3f. Percentage of time in MVPA during wake time across all occupations except office workers

Outcome	Study name	Comparison	Statistics for each study				Subgroup within study
			Mean	Lower limit	Upper limit	Standard error	
MVPA % wake	Cuddy, 2015	Protective services	12.000	10.988	13.012	0.516	Blank
MVPA % wake	Gao 2016 - OSS	Office workers with sit-stand desk	7.700	3.824	11.576	1.978	Blank
MVPA % wake	Reed 2018 a	Healthcare workers	4.640	4.416	4.864	0.114	Nurses
MVPA % wake	Steeves 2015 - CS	Customer service - hospitality	2.674	2.590	2.759	0.043	Combined
MVPA % wake	Steeves 2015 - D	Drivers	4.149	4.059	4.239	0.046	Combined
MVPA % wake	Steeves 2015 - EAS	Engineers, architects and scientists	4.000	3.745	4.255	0.130	Blank
MVPA % wake	Steeves 2015 - EPP	Extractive and precision production occupations	3.100	3.014	3.186	0.044	Blank
MVPA % wake	Steeves 2015 - F	Factory workers	3.532	3.385	3.680	0.075	Combined
MVPA % wake	Steeves 2015 - H	Healthcare workers	2.498	2.424	2.571	0.038	Combined
MVPA % wake	Steeves 2015 - house	Private household occupations	4.300	3.320	5.280	0.500	Blank
MVPA % wake	Steeves 2015 - L	Laborers	4.943	4.875	5.011	0.035	Combined
MVPA % wake	Steeves 2015 - Mgt	Management related occupations	3.500	3.388	3.612	0.057	Blank
MVPA % wake	Steeves 2015 - MRSD	Material recording, scheduling, distributing clerks	3.300	2.850	3.750	0.229	Blank
MVPA % wake	Steeves 2015 - Other	other professional speciality occupations	2.900	2.806	2.994	0.048	Blank
MVPA % wake	Steeves 2015 - personal	Personal service occupations	1.600	1.390	1.810	0.107	Blank
MVPA % wake	Steeves 2015 - PS	Protective services	3.400	2.856	3.944	0.277	Protective service occupation
MVPA % wake	Steeves 2015 - sales	Sales reps., finance, business, & commodities	3.500	3.282	3.718	0.111	Blank
MVPA % wake	Steeves 2015 - ST	School teachers	3.000	2.845	3.155	0.079	Blank
MVPA % wake	Steeves 2015 - sups	Supervisors and proprietors, sales occupations	3.100	2.911	3.289	0.096	Blank
MVPA % wake	Steeves 2015 - Tech	Technicians and related support occupations	3.600	3.464	3.736	0.070	Blank
MVPA % wake	Steeves 2015 - WAEA	Writers, artists, entertainers, and athletes	3.500	3.146	3.854	0.181	Blank
MVPA % wake	van Dommelen 2016 - A	Academics	3.899	3.460	4.338	0.224	Combined
MVPA % wake	van Dommelen 2016 - F	Financial services	3.788	3.495	4.081	0.149	Combined
MVPA % wake	van Dommelen 2016 - L	Laborers	4.300	3.537	5.063	0.389	Men
			3.827	3.433	4.222	0.201	

Mean and 95% CI

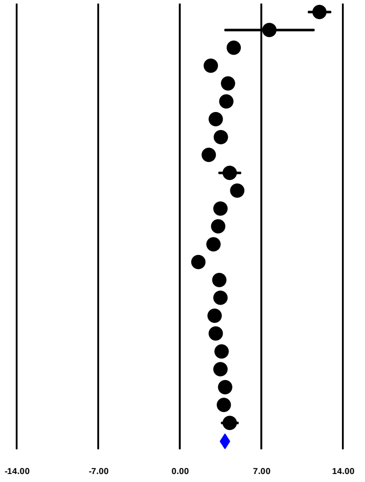

MVPA during wake time (%)

Supplemental figure 3g. Minuets/day of MVPA during wake time across all occupations

Outcome	Study name	Comparison	Statistics for each study				Subgroup within study
			Mean	Lower limit	Upper limit	Standard error	
MVPA - wake	Andersen, 2011	Drivers	25.000	20.813	29.187	2.136	Blank
MVPA - wake	Bergman 2018	Office workers	54.500	49.548	59.452	2.527	Combined
MVPA - wake	French 2007	Drivers	16.700	14.348	19.052	1.200	Blank
MVPA - wake	Gilson 2016	Office workers	96.000	85.097	106.903	5.563	Blank
MVPA - wake	Parry 2013 a	Office workers	39.500	34.317	44.683	2.645	Blank
MVPA - wake	Rafferty 2016	Office workers	32.700	26.127	39.273	3.354	Blank
MVPA - wake	Reed 2018 a	Healthcare workers	41.100	39.125	43.075	1.007	Nurses
MVPA - wake	Reed 2018b	Healthcare workers	3.914	2.337	5.491	0.805	Nurses
MVPA - wake	Ryde 2013	Office workers	44.300	39.461	49.139	2.469	Blank
MVPA - wake	Schulz 2018	Laborers	54.800	46.960	62.640	4.000	Farmers - peak season
MVPA - wake	Steeves 2018 - ADE	Blank	9.440	7.962	10.918	0.754	Arts, design, entertainment, sports, media
MVPA - wake	Steeves 2018 - Arch	Blank	9.500	8.801	10.199	0.357	Architecture, engineering
MVPA - wake	Steeves 2018 - BF	Blank	10.130	9.689	10.571	0.225	Business, financial operations
MVPA - wake	Steeves 2018 - C	Customer service - hospitality	6.960	6.388	7.532	0.292	Food preparation, serving
MVPA - wake	Steeves 2018 - CM	Blank	10.290	8.954	11.626	0.682	Computer, mathematical
MVPA - wake	Steeves 2018 - CSS	Blank	1.729	1.362	2.096	0.187	Community, social services
MVPA - wake	Steeves 2018 - D	Drivers	6.340	6.107	6.573	0.119	Transportation, material moving
MVPA - wake	Steeves 2018 - Educ	Blank	9.670	9.142	10.198	0.270	Education, training, library
MVPA - wake	Steeves 2018 - H	Healthcare workers	4.084	3.854	4.314	0.117	Combined
MVPA - wake	Steeves 2018 - IMR	Blank	5.340	4.878	5.802	0.236	Installation, maintenance, repair
MVPA - wake	Steeves 2018 - L	Laborers	9.528	9.267	9.789	0.133	Combined
MVPA - wake	Steeves 2018 - Legal	Blank	7.660	4.993	10.327	1.361	Legal
MVPA - wake	Steeves 2018 - LPSS	Blank	9.490	8.077	10.903	0.721	Life, physical, social science
MVPA - wake	Steeves 2018 - O	Office workers	5.810	5.605	6.015	0.104	Management
MVPA - wake	Steeves 2018 - P	Protective services	7.410	6.940	7.880	0.240	Protective service
MVPA - wake	Steeves 2018 - PCS	Blank	8.070	7.045	9.095	0.523	Personal care service
MVPA - wake	Steeves 2018 - Prod	Blank	3.490	3.369	3.611	0.062	Production
MVPA - wake	Steeves 2018 - Sales	Blank	6.000	5.741	6.259	0.132	Sales & related
MVPA - wake	Wong 2014	Drivers	36.000	26.682	45.318	4.754	Blank
			14.728	13.452	16.004	0.651	

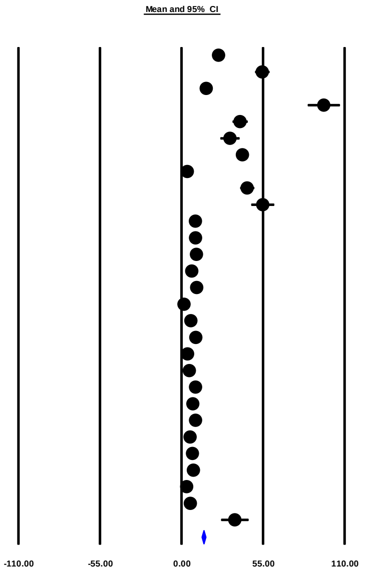

MVPA during wake time (min/day)

Supplemental figure 3h. Funnel plot for MVPA (min/day) during wake time across all occupations

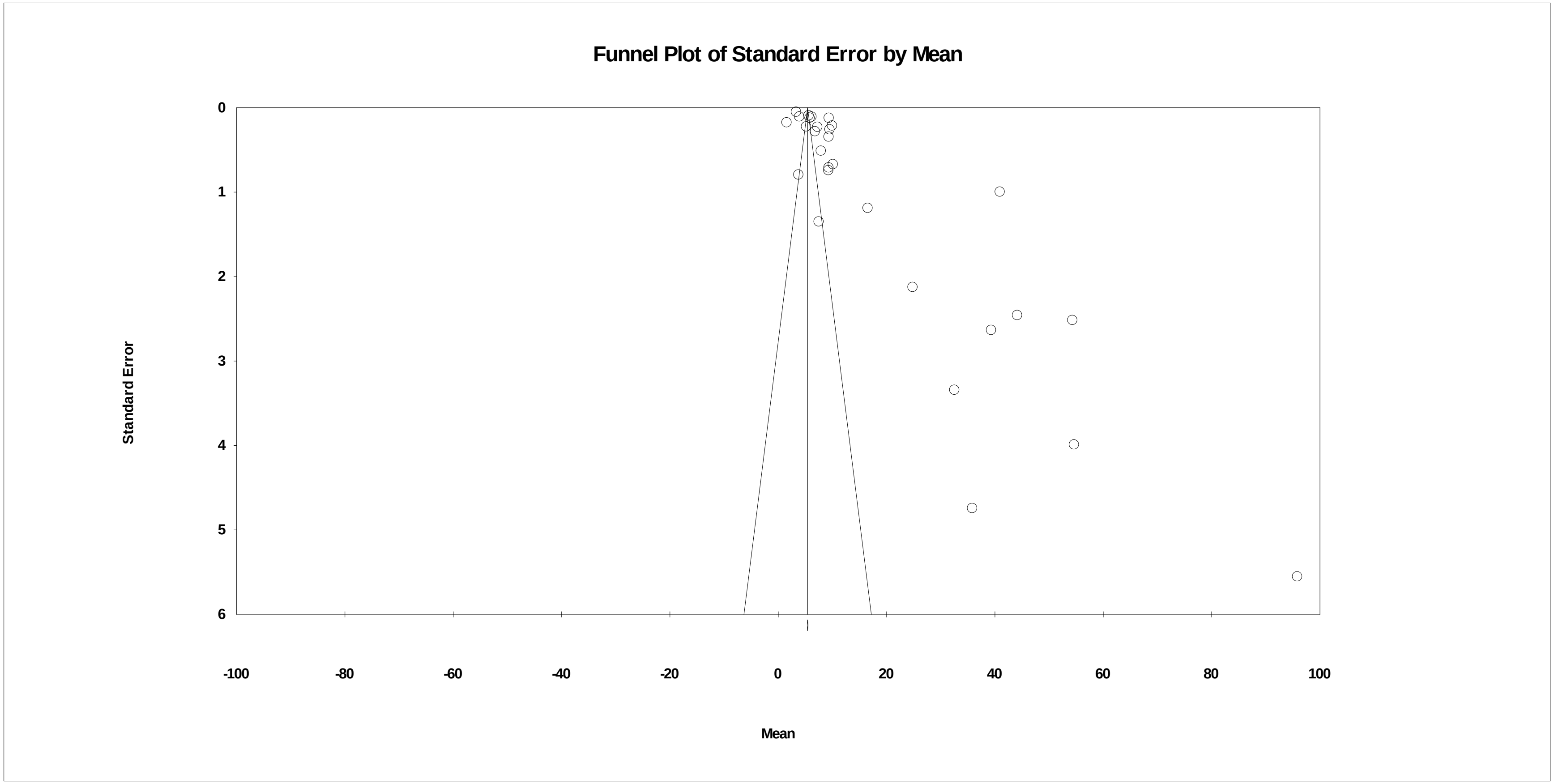

Supplemental figure 3g. Minutes/day of MVPA during wake time across in office workers

Outcome	Study name	Comparison	Mean	Lower limit	Upper limit	Standard error	Subgroup within study
MVPA - wake	Bergman 2018	Office workers	54.500	49.548	59.452	2.527	Combined
MVPA - wake	Gilson 2016	Office workers	96.000	85.097	106.903	5.563	Blank
MVPA - wake	Parry 2013 a	Office workers	39.500	34.317	44.683	2.645	Blank
MVPA - wake	Rafferty 2016	Office workers	32.700	26.127	39.273	3.354	Blank
MVPA - wake	Ryde 2013	Office workers	44.300	39.461	49.139	2.469	Blank
MVPA - wake	Steeves 2018 - O	Office workers	5.810	5.605	6.015	0.104	Management
			45.220	20.587	69.854	12.568	

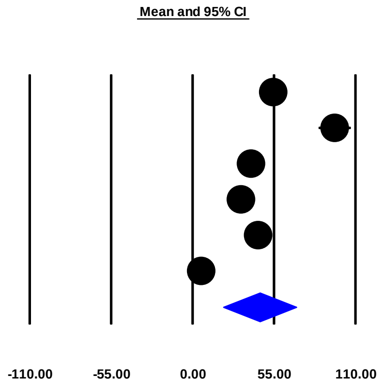

MVPA during wake time (min/day)

Supplemental figure 3h. Minutes/day of MVPA during wake time across in drivers

Outcome	Study name	Comparison	Statistics for each study				Subgroup within study
			Mean	Lower limit	Upper limit	Standard error	
MVPA - wake	Andersen, 2011	Drivers	25.000	20.813	29.187	2.136	Blank
MVPA - wake	French 2007	Drivers	16.700	14.348	19.052	1.200	Blank
MVPA - wake	Steeves 2018 - D	Drivers	6.340	6.107	6.573	0.119	Transportation, material moving
MVPA - wake	Wong 2014	Drivers	36.000	26.682	45.318	4.754	Blank
			20.227	10.243	30.211	5.094	

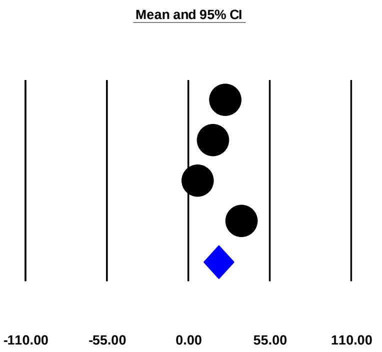

MVPA during wake time (min/day)

Supplemental figure 3h. Minutes/day of MVPA during wake time in healthcare workers

Outcome	Study name	Comparison	Mean	Lower limit	Upper limit	Standard error	Subgroup within study
MVPA - wake	Reed 2018 a	Healthcare workers	41.100	39.125	43.075	1.007	Nurses
MVPA - wake	Reed 2018b	Healthcare workers	3.914	2.337	5.491	0.805	Nurses
MVPA - wake	Steeves 2018 - H	Healthcare workers	4.084	3.854	4.314	0.117	Combined
			16.345	-2.264	34.955	9.495	

(Column group "Statistics for each study" spans Mean, Lower limit, Upper limit, Standard error.)

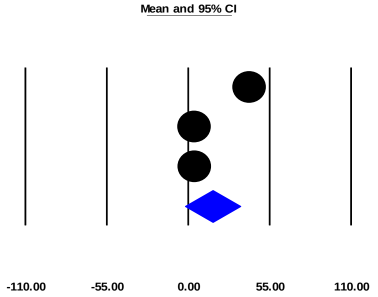

MVPA during wake time (min/day)

Supplemental figure 3i. Minutes/day of MVPA during wake time in all occupations except office workers

Outcome	Study name	Comparison	Statistics for each study				Subgroup within study
			Mean	Lower limit	Upper limit	Standard error	
MVPA - wake	Andersen, 2011	Drivers	25.000	20.813	29.187	2.136	Blank
MVPA - wake	French 2007	Drivers	16.700	14.348	19.052	1.200	Blank
MVPA - wake	Reed 2018 a	Healthcare workers	41.100	39.125	43.075	1.007	Nurses
MVPA - wake	Reed 2018b	Healthcare workers	3.914	2.337	5.491	0.805	Nurses
MVPA - wake	Schulz 2018	Laborers	54.800	46.960	62.640	4.000	Farmers - peak season
MVPA - wake	Steeves 2018 - ADE	Blank	9.440	7.962	10.918	0.754	Arts, design, entertainment, sports, media
MVPA - wake	Steeves 2018 - Arch	Blank	9.500	8.801	10.199	0.357	Architecture, engineering
MVPA - wake	Steeves 2018 - BF	Blank	10.130	9.689	10.571	0.225	Business, financial operations
MVPA - wake	Steeves 2018 - C	Customer service - hospitality	6.960	6.388	7.532	0.292	Food preparation, serving
MVPA - wake	Steeves 2018 - CM	Blank	10.290	8.954	11.626	0.682	Computer, mathematical
MVPA - wake	Steeves 2018 - CSS	Blank	1.729	1.362	2.096	0.187	Community, social services
MVPA - wake	Steeves 2018 - D	Drivers	6.340	6.107	6.573	0.119	Transportation, material moving
MVPA - wake	Steeves 2018 - Educ	Blank	9.670	9.142	10.198	0.270	Education, training, library
MVPA - wake	Steeves 2018 - H	Healthcare workers	4.084	3.854	4.314	0.117	Combined
MVPA - wake	Steeves 2018 - IMR	Blank	5.340	4.878	5.802	0.236	Installation, maintenance, repair
MVPA - wake	Steeves 2018 - L	Laborers	9.528	9.267	9.789	0.133	Combined
MVPA - wake	Steeves 2018 - Legal	Blank	7.660	4.993	10.327	1.361	Legal
MVPA - wake	Steeves 2018 - LPSS	Blank	9.490	8.077	10.903	0.721	Life, physical, social science
MVPA - wake	Steeves 2018 - P	Protective services	7.410	6.940	7.880	0.240	Protective service
MVPA - wake	Steeves 2018 - PCS	Blank	8.070	7.045	9.095	0.523	Personal care service
MVPA - wake	Steeves 2018 - Prod	Blank	3.490	3.369	3.611	0.062	Production
MVPA - wake	Steeves 2018 - Sales	Blank	6.000	5.741	6.259	0.132	Sales & related
MVPA - wake	Wong 2014	Drivers	36.000	26.682	45.318	4.754	Blank
			10.952	9.586	12.317	0.697	

Mean and 95% CI

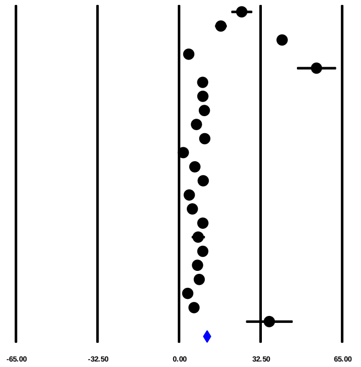

MVPA during wake time (min/day)

Supplemental figure 3j. Minutes/day of MVPA during work time in all occupations

Outcome	Study name	Comparison	Statistics for each study				Subgroup within study
			Mean	Lower limit	Upper limit	Standard error	
MVPA - work	Arundell 2018	Office workers	19.474	17.944	21.003	0.780	Combined
MVPA - work	Chau, 2016	Call centre workers	15.000	9.342	20.658	2.887	Blank
MVPA - work	Larouche 2018	Office workers	6.000	4.741	7.259	0.642	Blank
MVPA - work	Rafferty 2016	Office workers	3.500	2.039	4.961	0.745	Blank
MVPA - work	Ryde 2013	Office workers	16.800	14.275	19.325	1.288	Blank
MVPA - work	Zhu 2018	Office workers	6.672	5.107	8.238	0.799	Combined
			11.126	5.518	16.735	2.862	

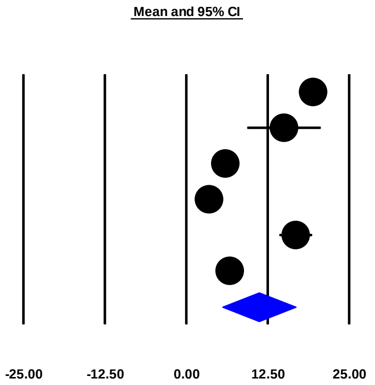

MVPA during work time (min/day)

Supplemental figure 3k. Minutes/day of MVPA during work time across healthcare workers

Outcome	Study name	Comparison	Statistics for each study				Subgroup within study
			Mean	Lower limit	Upper limit	Standard error	
MVPA - work	Arundell 2018	Office workers	19.474	17.944	21.003	0.780	Combined
MVPA - work	Larouche 2018	Office workers	6.000	4.741	7.259	0.642	Blank
MVPA - work	Rafferty 2016	Office workers	3.500	2.039	4.961	0.745	Blank
MVPA - work	Ryde 2013	Office workers	16.800	14.275	19.325	1.288	Blank
MVPA - work	Zhu 2018	Office workers	6.672	5.107	8.238	0.799	Combined
			10.458	4.345	16.570	3.119	

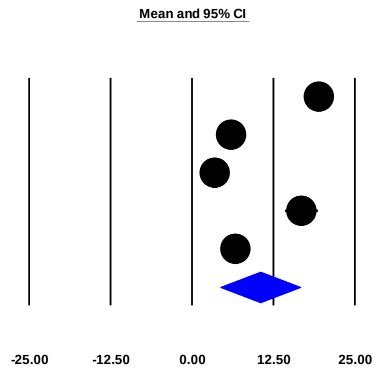

MVPA during work time (min/day)

Supplemental figure 4a-j

Moderate intensity physical activity (MPA)

Supplemental figure 4a. Percentage of time in MPA at work across all occupations

Outcome	Study name	Comparison	Statistics for each study				Subgroup within study
			Mean	Lower limit	Upper limit	Standard error	
MPA % work	Umukoro 2013	Healthcare workers	1.000	0.717	1.283	0.144	Nurses and aides
MPA % work	van Dommelen 2016 - A	Academics	3.454	3.036	3.872	0.213	Combined
MPA % work	van Dommelen 2016 - F	Financial services	2.880	2.510	3.250	0.189	Combined
MPA % work	van Dommelen 2016 - L	Laborers	7.100	5.447	8.753	0.844	Men
MPA % work	Vincent 2016	Protective services	23.000	18.692	27.308	2.198	Blank
			5.463	3.633	7.292	0.934	

Mean and 95% CI

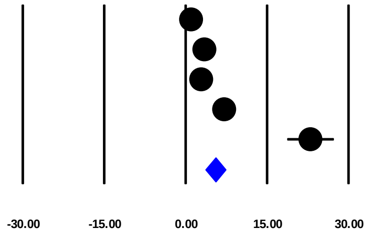

MPA during work time (%)

Supplemental figure 4b. Funnel plot for MPA (%) at work across all occupations

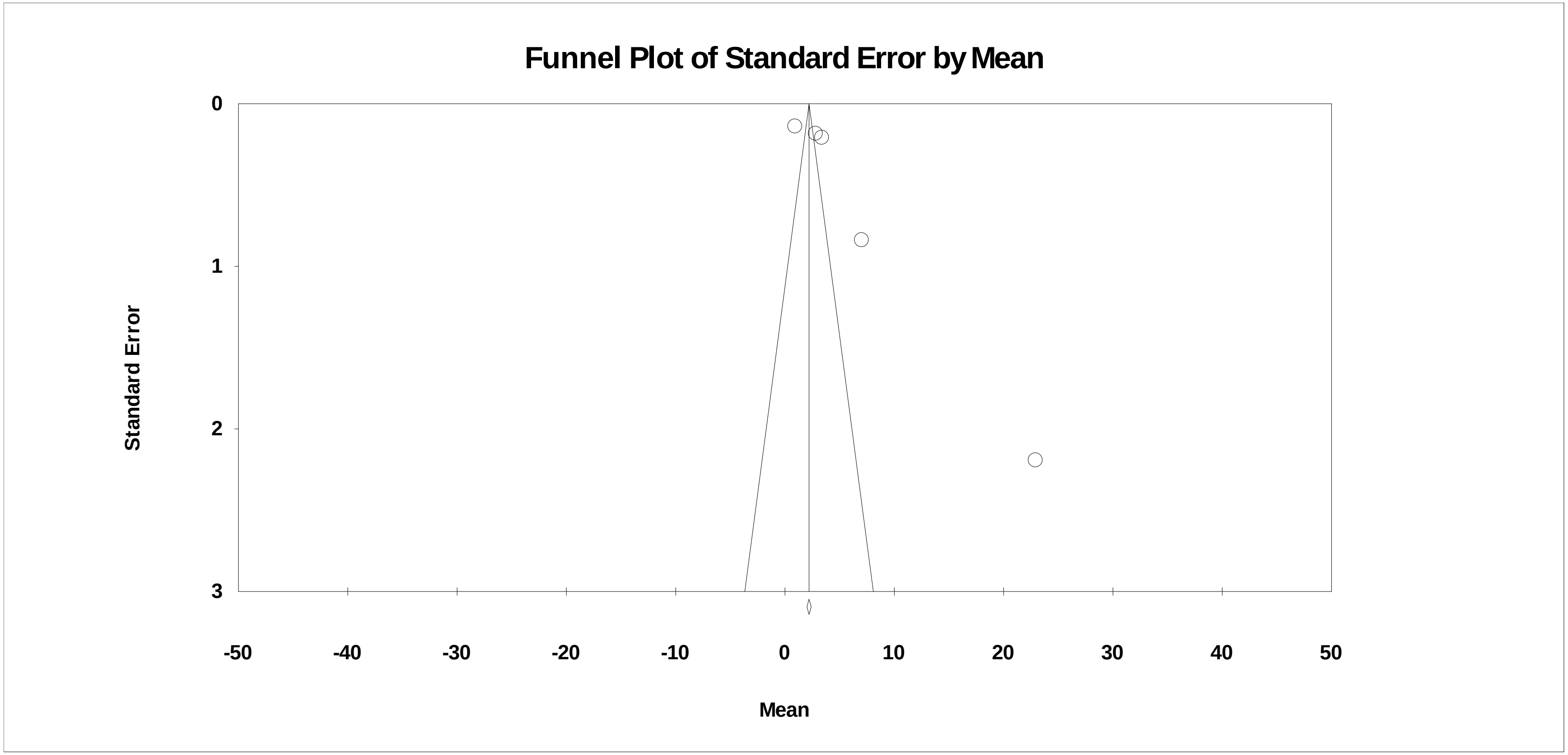

Supplemental figure 4c. Percentage of time in MPA during wake time across all occupations

Outcome	Study name	Comparison	Statistics for each study				Subgroup within study
			Mean	Lower limit	Upper limit	Standard error	
MPA % wake	Arias, 2015	Laborers	12.000	10.150	13.850	0.944	Blank
MPA % wake	Peavler 2012	Office workers	1.620	1.052	2.189	0.290	Combined
MPA % wake	Reed 2018 a	Healthcare workers	4.260	4.061	4.459	0.102	Nurses
MPA % wake	van Dommelen 2016 - A	Academics	3.641	3.223	4.059	0.213	Combined
MPA % wake	van Dommelen 2016 - F	Financial services	3.426	3.178	3.675	0.127	Combined
MPA % wake	van Dommelen 2016 - L	Laborers	4.200	3.437	4.963	0.389	Men
			4.355	3.425	5.285	0.475	

Mean and 95% CI

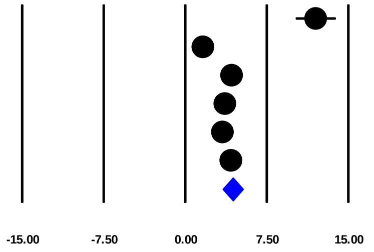

MPA during wake time (%)

Supplemental figure 4d. Funnel plot for MPA (%) during wake time across all occupations

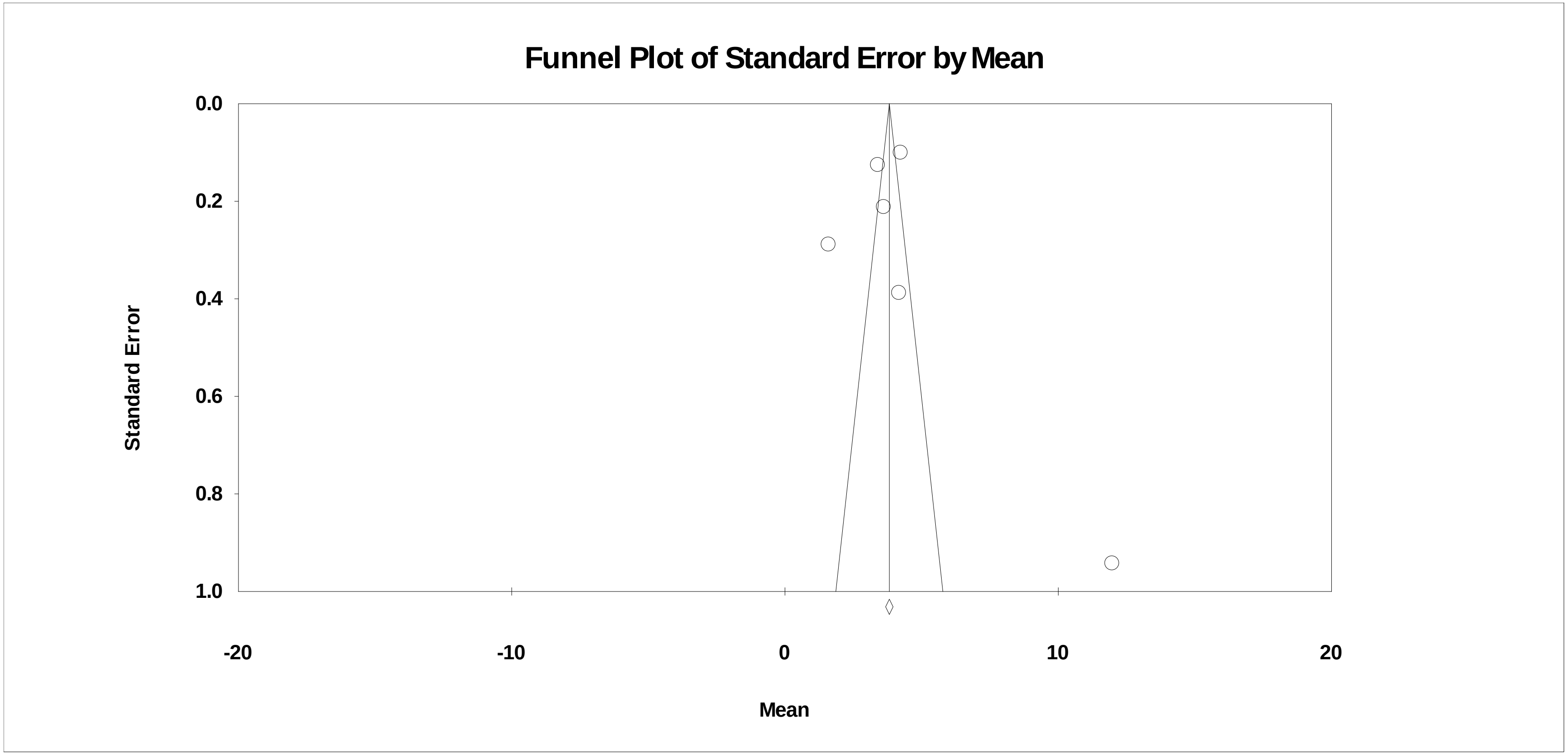

Supplemental figure 4e. Minutes/day of MPA at work across all occupations

Outcome	Study name	Comparison	Statistics for each study				Subgroup within study
			Mean	Lower limit	Upper limit	Standard error	
MPA - work	Chappel, 2016	Protective services	152.600	130.012	175.188	11.525	Blank
MPA - work	Jirathananuwat 2017	Healthcare workers	8.500	7.600	9.400	0.459	Blank
MPA - work	Umukoro 2013	Healthcare workers	4.286	2.992	5.579	0.660	Nurses and aides
MPA - work	Vincent 2016	Protective services	156.000	125.320	186.680	15.653	Blank
			43.225	31.942	54.508	5.757	

Mean and 95% CI

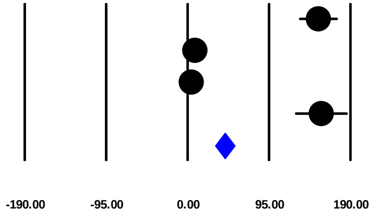

MPA during work time (min/day)

Supplemental figure 4f. Funnel plot for MPA (min/day) at work across all occupations

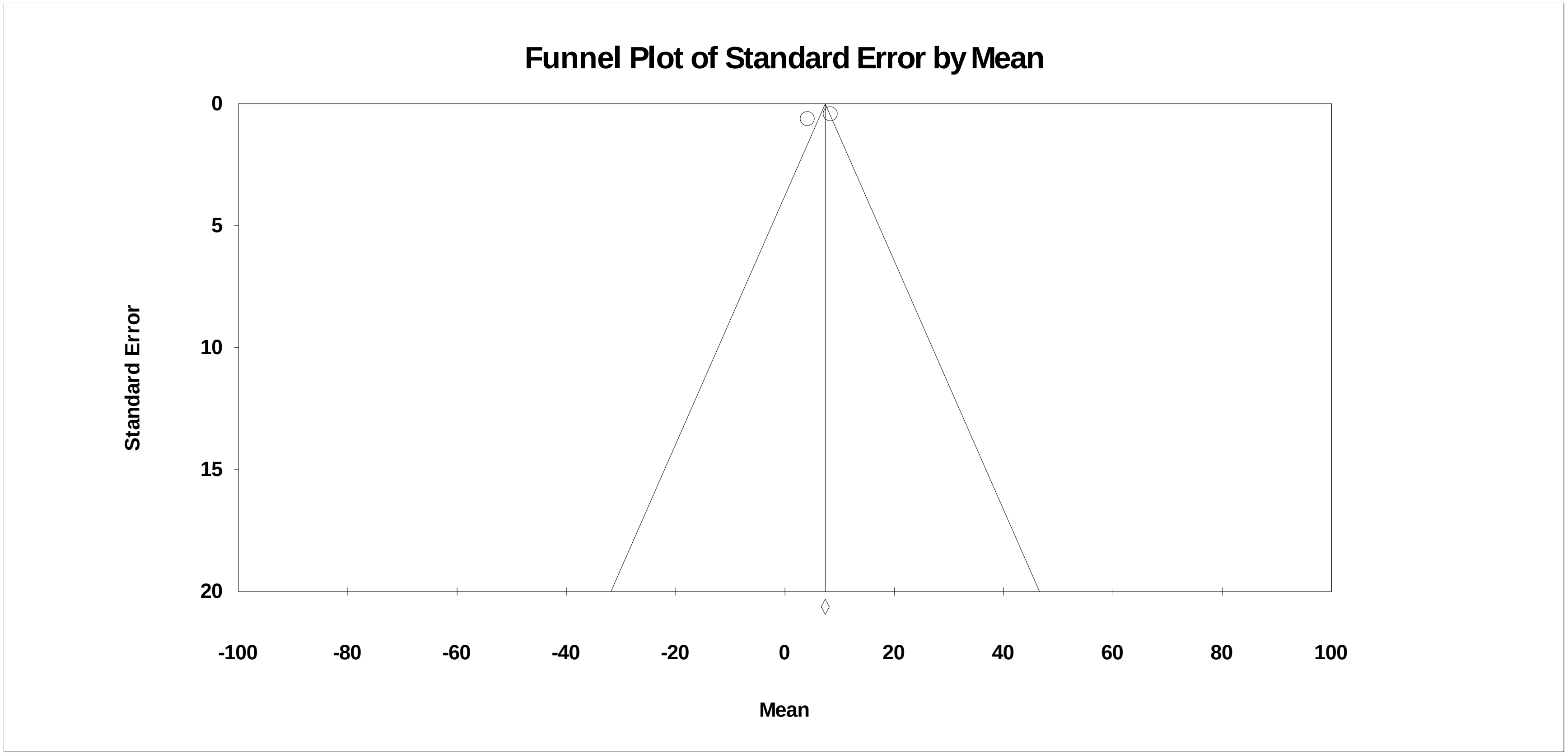

Supplemental figure 4g. Minutes/day of MPA during wake time across all occupations

Outcome	Study name	Comparison	Mean	Lower limit	Upper limit	Standard error	Subgroup within study
MPA - wake	Arias, 2015	Laborers	243.000	209.172	276.828	17.260	Blank
MPA - wake	Chitkara, 2014	Call centre workers	91.600	71.411	111.789	10.301	Blank
MPA wake	Finkelstein, 2015	Office workers	43.581	42.243	44.919	0.683	Combined
MPA - wake	Jancey 2016	Office workers	36.130	32.038	40.222	2.088	Blank
MPA - wake	Peavler 2012	Office workers	15.668	9.274	22.062	3.262	Combined
MPA - wake	Reed 2018 a	Healthcare workers	38.000	36.258	39.742	0.889	Nurses
MPA - wake	Umukoro 2013	Healthcare workers	23.571	19.732	27.411	1.959	Nurses and aides
MPA - wake	Weiler 2015	Athletes	74.100	63.085	85.115	5.620	Blank
			52.582	43.427	61.738	4.671	

Mean and 95% CI

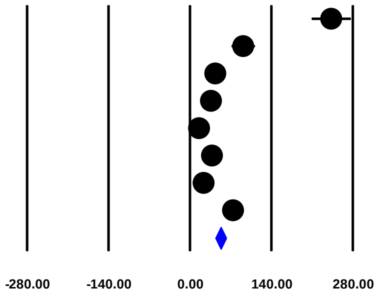

MPA during wake time (min/day)

Supplemental figure 4h. Funnel plot for MPA (min/day) during wake time across all occupations

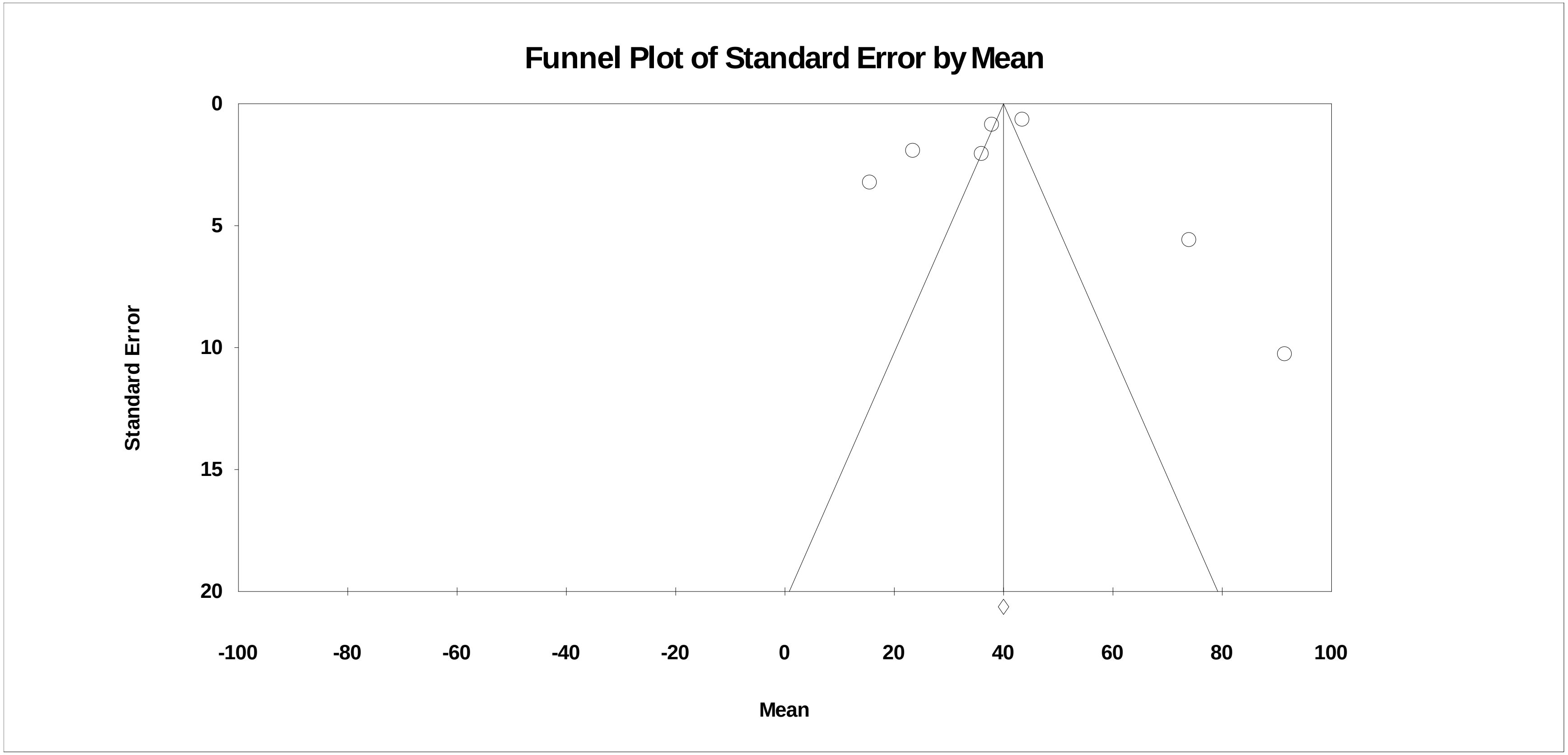

Supplemental figure 4i. Minutes/day in MPA during wake time in office workers

Outcome	Study name	Comparison	Statistics for each study				Subgroup within study
			Mean	Lower limit	Upper limit	Standard error	
MPA wake	Finkelstein, 2015	Office workers	43.581	42.243	44.919	0.683	Combined
MPA - wake	Jancey 2016	Office workers	36.130	32.038	40.222	2.088	Blank
MPA - wake	Peavler 2012	Office workers	15.668	9.274	22.062	3.262	Combined
			32.148	18.914	45.382	6.752	

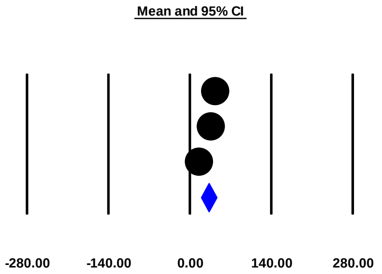

MPA during wake time (min/day)

Supplemental figure 4j. Percentage of time in MPA during wake time across all occupations except office workers

Outcome	Study name	Comparison	Statistics for each study				Subgroup within study
			Mean	Lower limit	Upper limit	Standard error	
MPA - wake	Arias, 2015	Laborers	243.000	209.172	276.828	17.260	Blank
MPA - wake	Chitkara, 2014	Call centre workers	91.600	71.411	111.789	10.301	Blank
MPA - wake	Reed 2018 a	Healthcare workers	38.000	36.258	39.742	0.889	Nurses
MPA - wake	Umukoro 2013	Healthcare workers	23.571	19.732	27.411	1.959	Nurses and aides
MPA - wake	Weiler 2015	Athletes	74.100	63.085	85.115	5.620	Blank
			82.045	60.929	103.160	10.773	

Mean and 95% CI

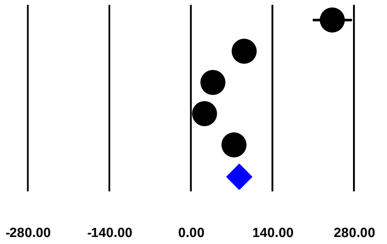

MPA during wake time (min/day)

Supplemental figures 5a-h

Vigorous intensity physical activity (VPA)

Supplemental figure 5a. Percentage of time in VPA at work across all occupations

Outcome	Study name	Comparison	Mean	Lower limit	Upper limit	Standard error	Subgroup within study
VPA % work	van Dommelen 2016 - A	Academics	0.431	0.328	0.534	0.053	Combined
VPA % work	van Dommelen 2016 - F	Financial services	0.538	0.327	0.749	0.108	Combined
VPA % work	Vincent 2016	Protective services	0.100	-0.117	0.317	0.111	Blank
			0.364	0.146	0.581	0.111	

Mean and 95% CI

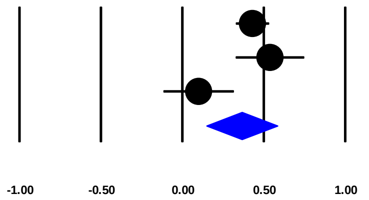

VPA during work time (%)

Supplemental figure 5b. Funnel plot for VPA (%) at work across all occupations

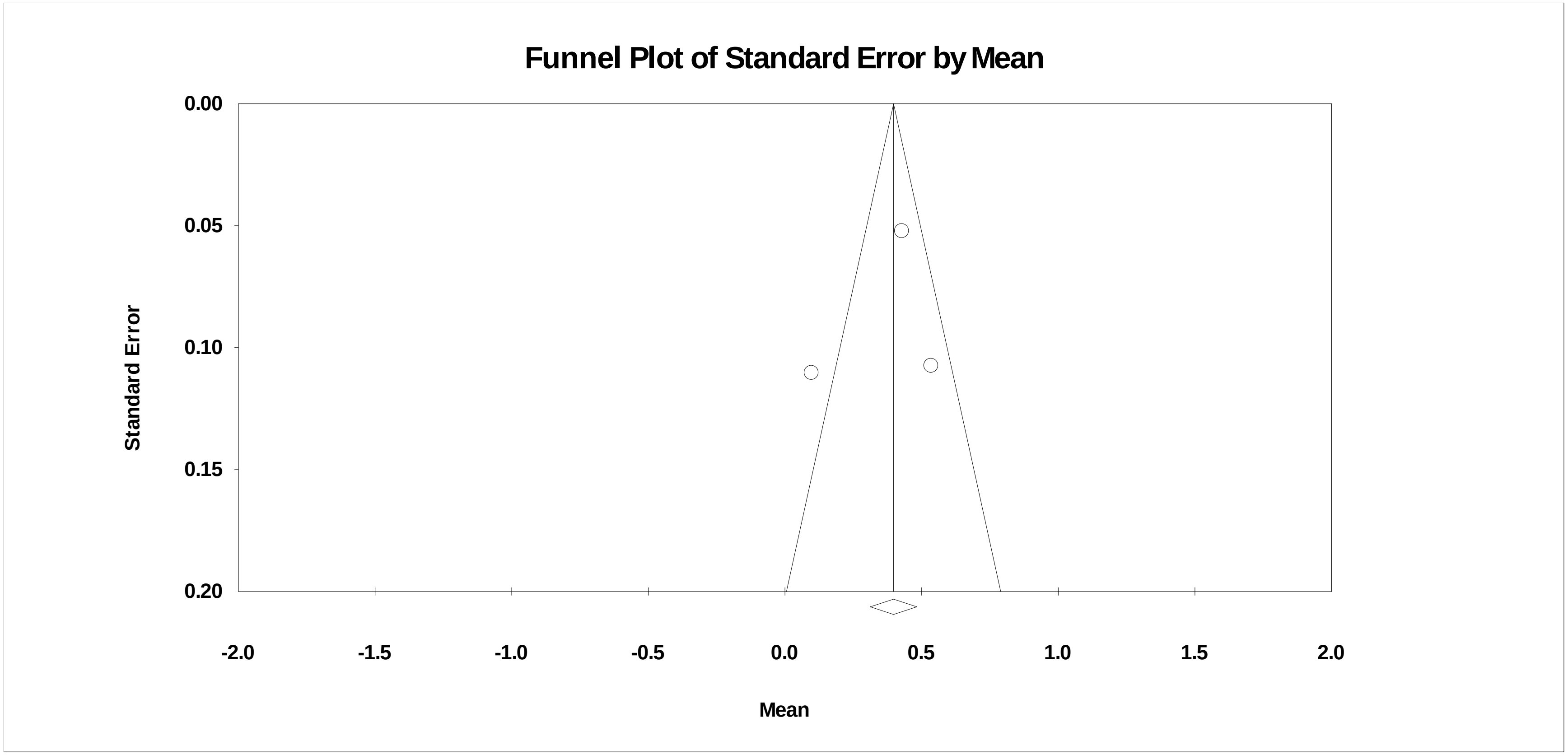

Supplemental figure 5c. Percentage of time in VPA during wake time across all occupations

Outcome	Study name	Comparison	Statistics for each study				Subgroup within study
			Mean	Lower limit	Upper limit	Standard error	
VPA%wake	Peavler 2012	Office workers	0.176	0.047	0.305	0.066	Combined
VPA%wake	van Dommelen 2016 - A	Academics	0.147	0.049	0.244	0.050	Combined
VPA%wake	van Dommelen 2016 - F	Financial services	0.331	0.214	0.447	0.060	Combined
VPA%wake	van Dommelen 2016 - L	Laborers	0.100	0.005	0.195	0.049	Men
			0.185	0.088	0.281	0.049	

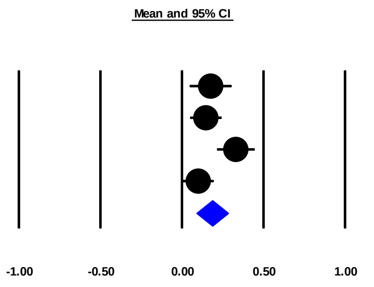

VPA during wake time (%)

Supplemental figure 5d. Funnel plot for VPA (%) during wake time across all occupations

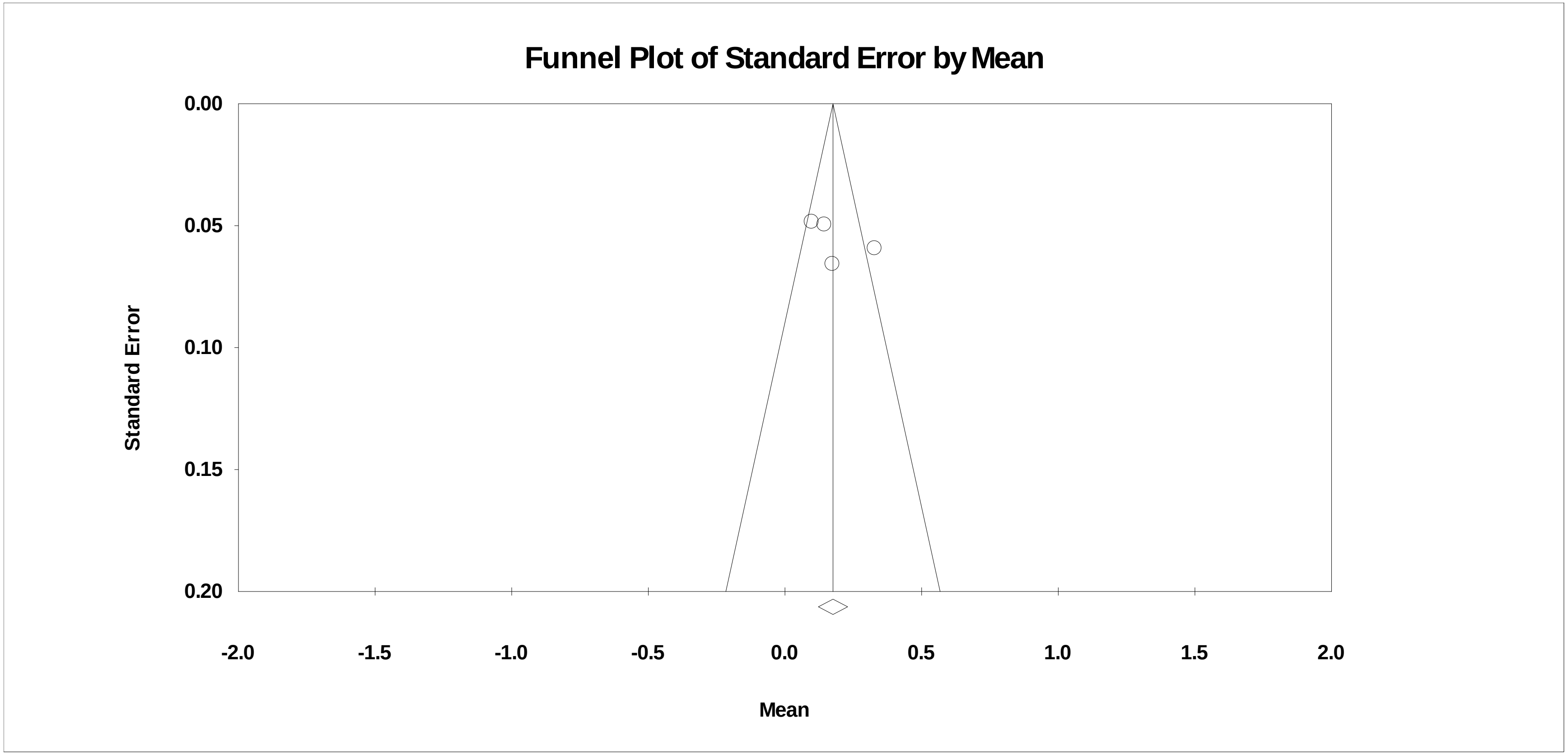

Supplemental figure 5e. Minutes/day of VPA at work across all occupations

Outcome	Study name	Comparison	Statistics for each study: Mean	Lower limit	Upper limit	Standard error	Subgroup within study
VPA - work	Chappel, 2016	Protective services	0.200	0.032	0.368	0.086	Blank
VPA wake	Finkelstein, 2015	Office workers	2.814	2.460	3.169	0.181	Combined
VPA - work	Jirathananuwat 2017	Healthcare workers	0.010	0.001	0.019	0.005	Blank
VPA - work	Umukoro 2013	Healthcare workers	0.057	-0.003	0.118	0.031	Nurses and aides
VPA - work	Vincent 2016	Protective services	1.000	-0.549	2.549	0.791	Blank
			0.662	0.340	0.984	0.164	

Mean and 95% CI

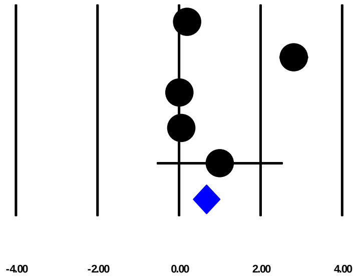

VPA during work time (min/day)

Supplemental figure 5f. Funnel plot for VPA (min/day) at work across all occupations

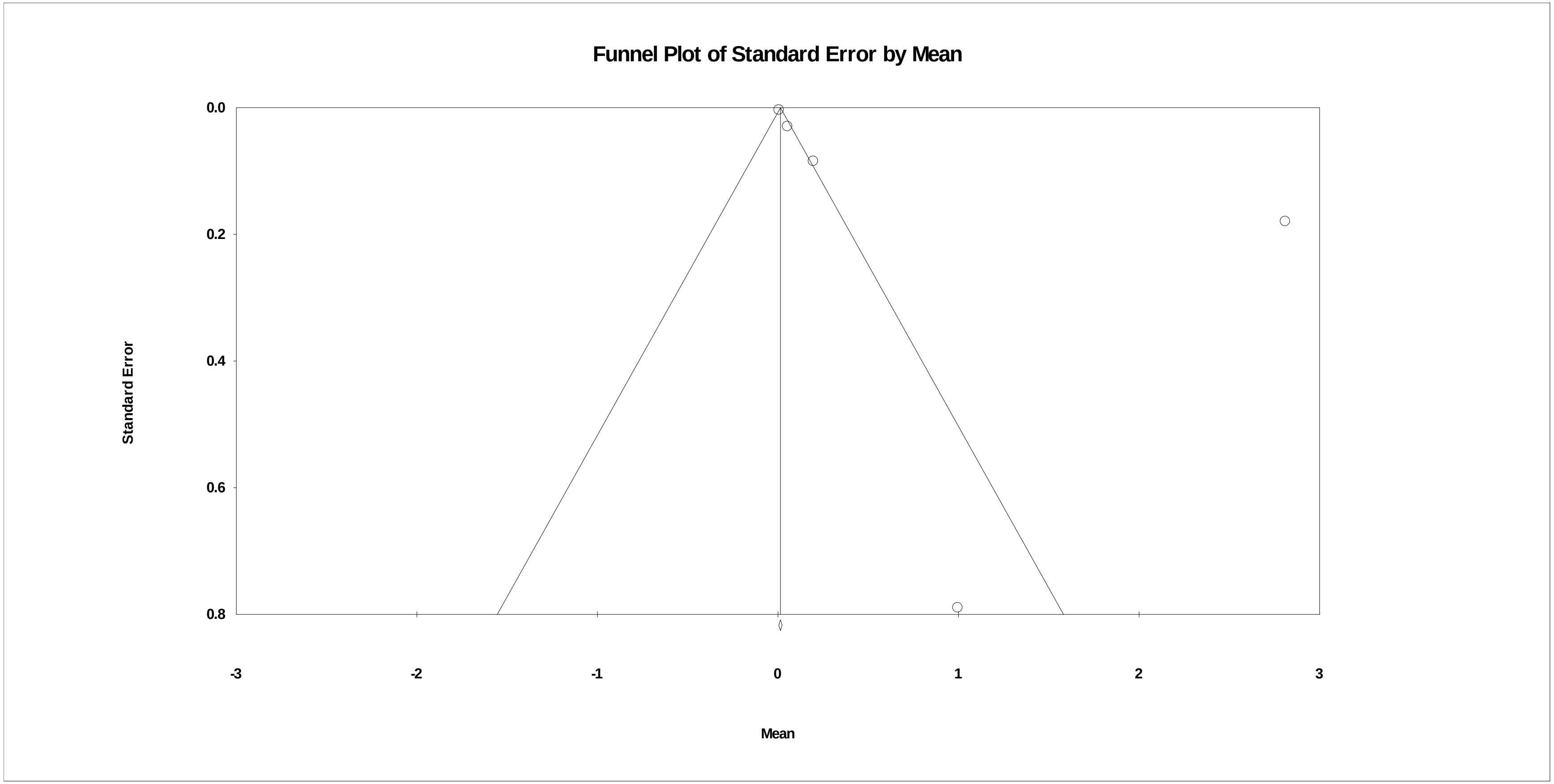

Supplemental figure 5g. Minutes/day of VPA during wake time across all occupations

Outcome	Study name	Comparison	Mean	Lower limit	Upper limit	Standard error	Subgroup within study
VPA - wake	Arias, 2015	Laborers	2.000	0.414	3.586	0.809	Blank
VPA - wake	Chitkara, 2014	Call centre workers	0.900	-0.584	2.384	0.757	Blank
VPA wake	Finkelstein, 2015	Office workers	2.814	2.460	3.169	0.181	Combined
VPA - wake	Jancey 2016	Office workers	0.330	0.139	0.521	0.097	Blank
VPA - wake	Peavler 2012	Office workers	1.474	0.356	2.591	0.570	Combined
VPA - wake	Reed 2018 a	Healthcare workers	3.000	2.516	3.484	0.247	Nurses
VPA - wake	Umukoro 2013	Healthcare workers	2.571	0.793	4.350	0.907	Nurses and aides
VPA - wake	Weiler 2015	Athletes	2.100	0.571	3.629	0.780	Blank
			1.895	0.732	3.059	0.594	

Mean and 95% CI

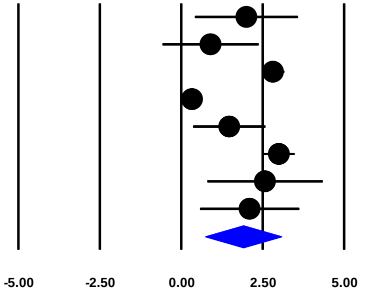

VPA during work time (min/day)

Supplemental figure 5h. Funnel plot for VPA (min/day) during wake time across all occupations

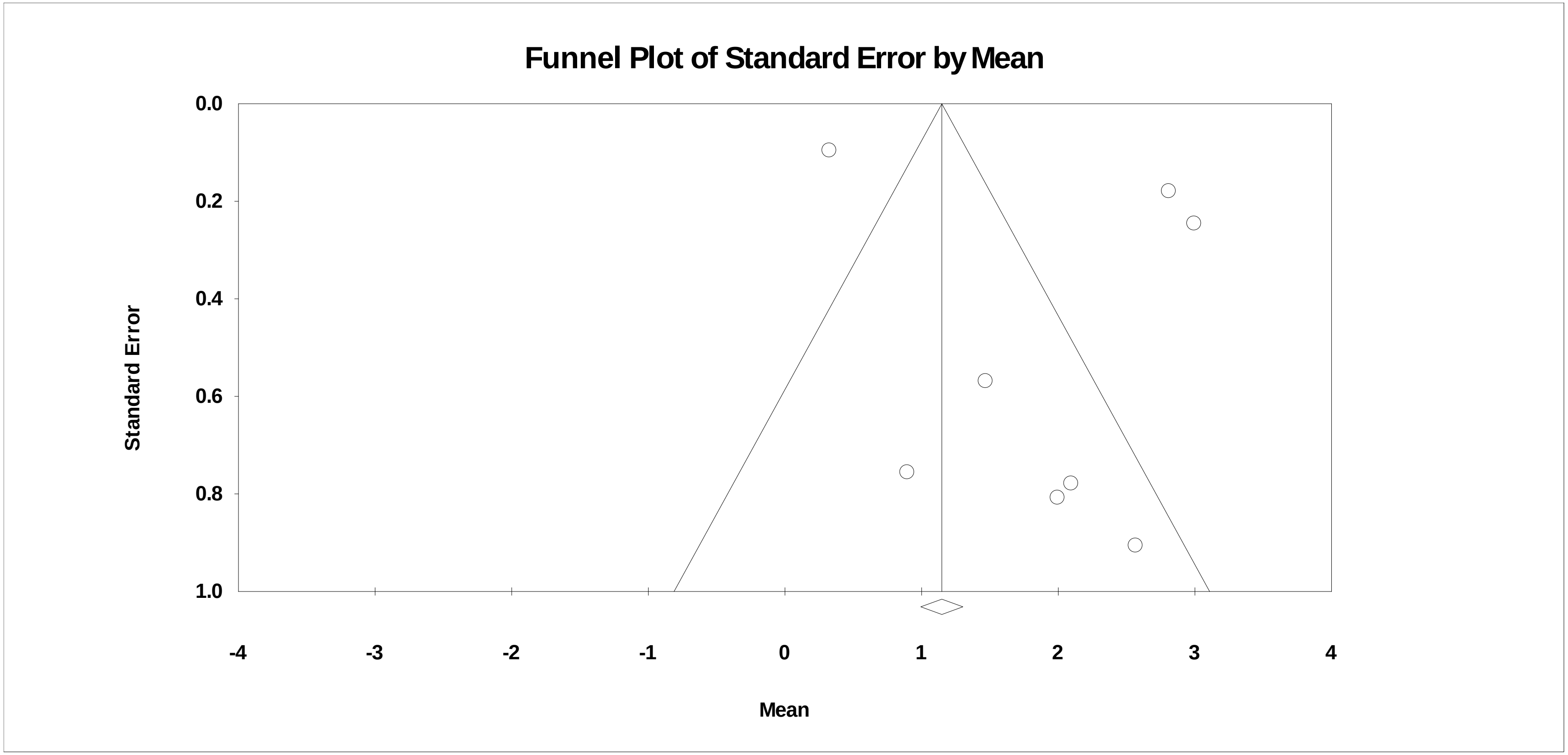

Supplemental figures 6a-g

Steps

Supplemental figure 6a. Steps per day at work across all occupations

Outcome	Study name	Comparison	Mean	Lower limit	Upper limit	Standard error	Subgroup within study
Steps - work	Atkinson, 2005	Healthcare workers	7429.444	7226.711	7632.177	103.437	Combined
Steps - work	Badland 2004	Office workers	4833.304	4463.874	5202.735	188.488	Combined
Steps - work	Brakenridge, 2016	Office workers	2286.800	2164.109	2409.491	62.598	Blank
Steps - work	Cheung 2008	School teachers	7223.000	6630.752	7815.248	302.173	Blank
Steps - work	Cheung, 2012	School teachers	7394.000	6937.397	7850.603	232.965	Blank
Steps - work	Copeland, 2017	Healthcare workers	8362.500	7382.560	9342.440	499.978	Blank
Steps - work	Fisher 2018	Office workers	3412.000	3083.385	3740.615	167.664	Blank
Steps - work	Julin 2011	Laborers	9820.000	8495.100	11144.900	675.982	Blank
Steps - work	Li 2017	Office workers	4347.969	3743.041	4952.898	308.643	Combined
Steps - work	Neuhaus 2014	Office workers	1742.000	1509.756	1974.244	118.494	Blank
Steps - work	Rafferty 2016	Office workers	3564.000	2949.375	4178.625	313.590	Blank
Steps - work	Schofield 2005 - A	Academics	4422.000	3994.341	4849.659	218.197	Blank
Steps - work	Schofield 2005 - allied uni	Allied university	4790.000	4213.457	5366.543	294.160	Blank
Steps - work	Schofield 2005 - H	Healthcare workers	5446.000	3846.295	7045.705	816.191	Blank
Steps - work	Schofield 2005 - L	Laborers	10334.000	6706.107	13961.893	1851.000	Blank
Steps - work	Schofield 2005 - O	Office workers	5380.000	4705.875	6054.125	343.948	Blank
Steps - work	Schofield 2005 CS	Customer service - hospitality	8384.000	6448.380	10319.620	987.579	Blank
			5697.919	4484.446	6911.393	619.130	

Mean and 95% CI

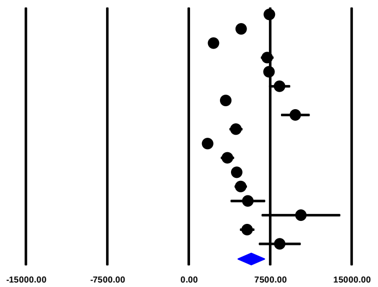

Steps per day during work time

Supplemental figure 6b. Steps per day at work in office workers

Outcome	Study name	Comparison	Statistics for each study				Subgroup within study
			Mean	Lower limit	Upper limit	Standard error	
Steps - work	Badland 2004	Office workers	4833.304	4463.874	5202.735	188.488	Combined
Steps - work	Brakenridge, 2016	Office workers	2286.800	2164.109	2409.491	62.598	Blank
Steps - work	Fisher 2018	Office workers	3412.000	3083.385	3740.615	167.664	Blank
Steps - work	Li 2017	Office workers	4347.969	3743.041	4952.898	308.643	Combined
Steps - work	Neuhaus 2014	Office workers	1742.000	1509.756	1974.244	118.494	Blank
Steps - work	Rafferty 2016	Office workers	3564.000	2949.375	4178.625	313.590	Blank
Steps - work	Schofield 2005 - O	Office workers	5380.000	4705.875	6054.125	343.948	Blank
			3626.147	2735.818	4516.477	454.258	

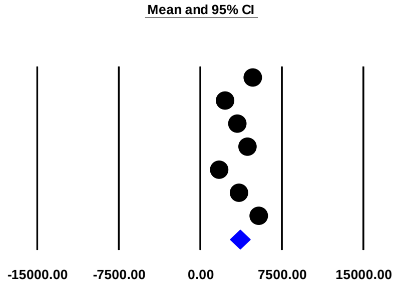

Steps per day during work time

Supplemental figure 6c. Steps per day at work in healthcare workers

Outcome	Study name	Comparison	Statistics for each study				Subgroup within study
			Mean	Lower limit	Upper limit	Standard error	
Steps - work	Atkinson, 2005	Healthcare workers	7429.444	7226.711	7632.177	103.437	Combined
Steps - work	Copeland, 2017	Healthcare workers	8362.500	7382.560	9342.440	499.978	Blank
Steps - work	Schofield 2005 - H	Healthcare workers	5446.000	3846.295	7045.705	816.191	Blank
			7281.945	6183.473	8380.417	560.455	

Mean and 95% CI

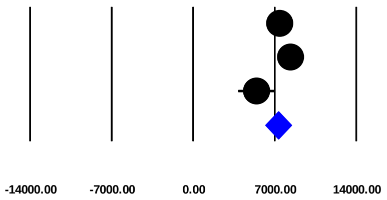

Steps per day during work time

Supplemental figure 6d. Steps per day at work across all occupations except office workers

Outcome	Study name	Comparison	Mean	Lower limit	Upper limit	Standard error	Subgroup within study
Steps - work	Atkinson, 2005	Healthcare workers	7429.444	7226.711	7632.177	103.437	Combined
Steps - work	Cheung 2008	School teachers	7223.000	6630.752	7815.248	302.173	Blank
Steps - work	Cheung, 2012	School teachers	7394.000	6937.397	7850.603	232.965	Blank
Steps - work	Copeland, 2017	Healthcare workers	8362.500	7382.560	9342.440	499.978	Blank
Steps - work	Julin 2011	Laborers	9820.000	8495.100	11144.900	675.982	Blank
Steps - work	Schofield 2005 - A	Academics	4422.000	3994.341	4849.659	218.197	Blank
Steps - work	Schofield 2005 - allied uni	Allied university	4790.000	4213.457	5366.543	294.160	Blank
Steps - work	Schofield 2005 - H	Healthcare workers	5446.000	3846.295	7045.705	816.191	Blank
Steps - work	Schofield 2005 - L	Laborers	10334.000	6706.107	13961.893	1851.000	Blank
Steps - work	Schofield 2005 CS	Customer service - hospitality	8384.000	6448.380	10319.620	987.579	Blank
			7123.701	6107.120	8140.281	518.673	

Mean and 95% CI

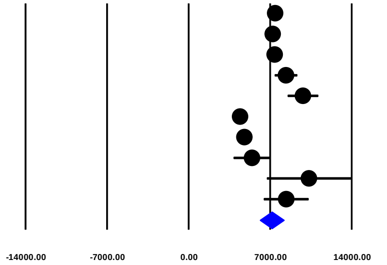

Steps per day during work time

Supplemental figure 6e. Steps per day during wake time across all occupations

Outcome	Study name	Comparison	Statistics for each study: Mean	Lower limit	Upper limit	Standard error	Subgroup within study
Steps - wake	Abdt, 2012	Healthcare workers	1999.000	1258.351	2739.649	377.889	Blank
Steps - wake	Badland 2004	Office workers	9035.452	8272.671	9798.233	389.181	Combined
Steps - wake	Bassey, 1983	Factory workers	11600.000	10620.019	12579.981	500.000	Blank
Steps - wake	Bergman 2018	Office workers	8988.357	8346.887	9629.827	327.287	Combined
Steps - wake	Brakenridge, 2016	Office workers	5004.000	4777.205	5230.795	115.714	Blank
Steps - wake	Brett, 2017 - A	Academics	9264.200	7310.997	11217.403	996.551	Blank
Steps - wake	Brett, 2017 - O	Office workers	10554.760	8188.290	12921.230	1207.405	Blank
Steps - wake	Brewer, 2016	Healthcare workers	3764.619	3415.000	4114.238	178.380	Combined
Steps - wake	Chae, 2015	Office workers	5403.670	4982.221	5825.119	215.029	Blank
Steps - wake	Chan 2018	Healthcare workers	10620.000	9740.536	11499.464	448.714	Blank
Steps - wake	Chastin, 2009 - O	Office workers	11007.000	9751.618	12262.382	640.513	Blank
Steps - wake	Chastin, 2009 - PD	Postal workers - delivery	17065.000	15809.618	18320.382	640.513	Blank
Steps - wake	Chau, 2016	Call centre workers	1642.252	1142.552	2141.952	254.954	Combined
Steps - wake	Cheung 2008	School teachers	11399.000	10733.095	12064.905	339.754	Blank
Steps - wake	Cheung, 2012	School teachers	11351.000	10853.691	11848.309	253.734	Blank
Steps - wake	Chitkara, 2014	Call centre workers	5361.000	4232.837	6489.163	575.604	Blank
Steps - wake	Choi 2010	Protective services	3220.635	3195.839	3245.430	12.651	Combined
Steps - wake	Clemes, 2014	Office workers	3742.000	3166.157	4317.843	293.803	Blank
Steps - wake	Croteau 2017	Healthcare workers	8446.000	7452.900	9439.100	506.693	Blank
Steps - wake	Finkelstein, 2015	Office workers	8012.106	7845.084	8179.128	85.217	Combined
Steps - wake	Gany 2014	Drivers	3731.900	3250.061	4213.739	245.841	Blank
Steps - wake	Gilson 2010a	Office workers	7919.000	7120.478	8717.522	407.417	Blank
Steps - wake	Gilson 2010b	Office workers	7473.000	6446.592	8499.408	523.687	Blank
Steps - wake	Gilson 2012	Office workers	5892.000	5674.489	6109.511	110.977	Blank
Steps - wake	Healy 2013	Office workers	1952.909	1755.804	2150.014	100.566	Combined
Steps - wake	James 2009	Healthcare workers	10865.000	9684.870	12045.130	602.118	Blank
Steps - wake	Jancey 2016	Office workers	3238.200	2841.958	3634.442	202.168	Blank
Steps - wake	Jirathananuwat 2017	Healthcare workers	11416.500	10943.181	11889.819	241.494	Blank
Steps - wake	Ju 2011	Laborers	8000.900	7443.974	8557.826	284.151	Blank
Steps - wake	Julin 2011	Laborers	15085.000	13300.033	16869.967	910.714	Blank
Steps - wake	Korshoj 2013	Laborers	20198.000	18170.165	22225.835	1034.629	Blank
Steps - wake	Kozey-Keadley 2012	Office workers	6417.000	4941.813	7892.187	752.660	Blank
Steps - wake	Mansi 2015 - F	Factory workers	5885.221	5575.930	6194.512	157.804	Combined
Steps - wake	Miller 2004 - A	Academics	10147.000	9395.839	10898.161	383.253	Scientists
Steps - wake	Miller 2004 - BC	Blue-collar workers	11784.000	9312.050	14255.950	1261.222	Blank
Steps - wake	Miller 2004 - O	Office workers	7580.590	7041.318	8119.863	275.144	Combined
Steps - wake	Miller 2004 - tech	Technicians	10731.000	9936.037	11525.963	405.601	Blank
Steps - wake	Mitsui 2010	Office workers	5236.000	4611.512	5860.488	318.622	Blank
Steps - wake	Pontt 2015 - L	Laborers	11296.000	9859.101	12732.899	733.125	Blank
Steps - wake	Pontt 2015 - O	Office workers	8886.000	7766.107	10005.893	571.385	Blank
Steps - wake	Rafferty 2016	Office workers	11008.000	9855.243	12160.757	588.152	Blank
Steps - wake	Reed 2018 a	Healthcare workers	8176.000	7948.433	8403.567	116.108	Nurses
Steps - wake	Reed 2018b	Healthcare workers	8885.000	8076.535	9693.465	412.489	Nurses
Steps - wake	Schnebly 2017	Healthcare workers	9369.700	8471.845	10267.555	458.098	Blank
Steps - wake	Schofield 2005 - A	Academics	9702.000	8732.331	10671.669	494.738	Blank
Steps - wake	Schofield 2005 - allied uni	Allied university	4988.000	4103.383	5872.617	451.344	Blank
Steps - wake	Schofield 2005 - H	Healthcare workers	6691.000	4574.803	8807.197	1079.712	Blank
Steps - wake	Schofield 2005 - L	Laborers	14535.000	11027.971	18042.029	1789.333	Blank
Steps - wake	Schofield 2005 - O	Office workers	9200.000	8219.679	10180.321	500.173	Blank
Steps - wake	Schofield 2005 CS	Customer service - hospitality	13919.000	11628.238	16209.762	1168.778	Blank
Steps - wake	Smith 2002 - A	Academics	7138.537	6174.220	8102.854	492.007	Combined
Steps - wake	Smith 2002 - eng	Professional engineers	7947.000	6729.862	9164.138	621.000	Blank
Steps - wake	Smith 2002 - H	Healthcare workers	9564.000	8048.349	11079.651	773.306	Blank
Steps - wake	Smith 2002 - house	Housewives	5674.000	4158.058	7189.942	773.454	Blank
Steps - wake	Smith 2002 - O	Office workers	5923.141	5458.364	6387.919	237.136	Combined
Steps - wake	Smith 2002 - PD	Postal workers - delivery	12713.000	11629.214	13796.786	552.962	Blank
Steps - wake	Smith 2002 - S	School teachers	7285.000	6362.912	8207.088	470.462	Blank
Steps - wake	Smith 2002 tech	Technicians	8866.000	7308.938	10423.062	794.434	Blank
Steps - wake	Smith 2015	Office workers	9737.000	9198.732	10275.268	274.632	Blank
Steps - wake	Steeves 2018 - ADE	Blank	6552.000	6361.799	6742.201	97.043	Arts, design, entertainment, sports, media
Steps - wake	Steeves 2018 - Arch	Blank	7119.000	6968.083	7269.917	77.000	Architecture, engineering
Steps - wake	Steeves 2018 - BF	Blank	7215.000	7122.067	7307.933	47.416	Business, financial operations
Steps - wake	Steeves 2018 - C	Customer service - hospitality	9388.000	9225.043	9550.957	83.143	Food preparation, serving
Steps - wake	Steeves 2018 - CM	Blank	6199.000	6056.598	6341.402	72.655	Computer, mathematical
Steps - wake	Steeves 2018 - CSS	Blank	5684.000	5463.307	5904.693	112.600	Community, social services
Steps - wake	Steeves 2018 - D	Drivers	9960.000	9879.729	10040.271	40.955	Transportation, material moving
Steps - wake	Steeves 2018 - Educ	Blank	7890.000	7804.315	7975.685	43.718	Education, training, library
Steps - wake	Steeves 2018 - H	Healthcare workers	6813.577	6739.430	6887.724	37.831	Combined
Steps - wake	Steeves 2018 - IMR	Blank	9004.000	8790.679	9217.321	108.839	Installation, maintenance, repair
Steps - wake	Steeves 2018 - L	Laborers	9596.625	9519.391	9673.860	39.406	Combined
Steps - wake	Steeves 2018 - Legal	Blank	5804.000	5200.865	6407.135	307.728	Legal
Steps - wake	Steeves 2018 - LPSS	Blank	6196.000	5966.680	6425.320	117.002	Life, physical, social science
Steps - wake	Steeves 2018 - O	Office workers	6831.041	6800.858	6861.224	15.400	Combined
Steps - wake	Steeves 2018 - P	Protective services	7109.000	6953.277	7264.723	79.452	Protective service
Steps - wake	Steeves 2018 - PCS	Blank	8339.000	8113.935	8564.065	114.831	Personal care service
Steps - wake	Steeves 2018 - Prod	Blank	8151.000	8103.084	8198.916	24.447	Production
Steps - wake	Steeves 2018 - Sales	Blank	7243.000	7182.661	7303.339	30.786	Sales & related
Steps - wake	Swartz 2014	Office workers	3574.000	3520.105	3627.895	27.498	Blank
Steps - wake	Talbot 2011	Protective services	6699.573	6033.613	7365.533	339.782	Combined
Steps - wake	Taylor 2016	Office workers	6484.271	6009.836	6958.706	242.063	Combined
Steps - wake	Tigbe 2011 - O	Office workers	10853.000	10088.481	11617.519	390.068	Blank
Steps - wake	Tigbe 2011 - PD	Postal workers - delivery	18554.000	17321.446	19786.554	628.866	Blank
Steps - wake	Tobin 2016	Office workers	2453.000	2122.406	2783.594	168.673	Blank
Steps - wake	Torquati 2018	Healthcare workers	8496.000	7773.270	9218.730	368.747	Nurses
Steps - wake	Wieters 2009	Office workers	4596.155	4376.794	4815.515	111.921	Combined
			8123.512	7586.290	8660.735	274.098	

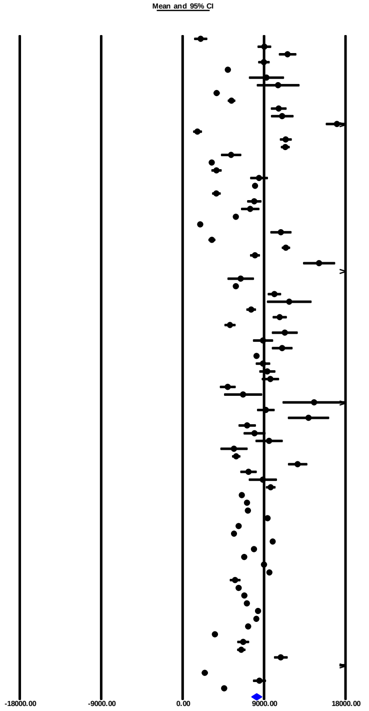

Steps per day during wake time

Supplemental figure 6f. Steps per day during wake time in office workers

Outcome	Studyname	Comparison	Statistics for each study				Subgroup within study
			Mean	Lower limit	Upper limit	Standard error	
Steps - wake	Badland 2004	Office workers	9035.452	8272.671	9798.233	389.181	Combined
Steps - wake	Bergman 2018	Office workers	8988.357	8346.887	9629.827	327.287	Combined
Steps - wake	Brakenridge, 2016	Office workers	5004.000	4777.205	5230.795	115.714	Blank
Steps - wake	Brett, 2017 - O	Office workers	10554.760	8188.290	12921.230	1207.405	Blank
Steps - wake	Chae, 2015	Office workers	5403.670	4982.221	5825.119	215.029	Blank
Steps - wake	Chastin, 2009 - O	Office workers	11007.000	9751.618	12262.382	640.513	Blank
Steps - wake	Clemes, 2014	Office workers	3742.000	3166.157	4317.843	293.803	Blank
Steps - wake	Finkelstein, 2015	Office workers	8012.106	7845.084	8179.128	85.217	Combined
Steps - wake	Gilson 2010a	Office workers	7919.000	7120.478	8717.522	407.417	Blank
Steps - wake	Gilson 2010b	Office workers	7473.000	6446.592	8499.408	523.687	Blank
Steps - wake	Gilson 2012	Office workers	5892.000	5674.489	6109.511	110.977	Blank
Steps - wake	Healy 2013	Office workers	1952.909	1755.804	2150.014	100.566	Combined
Steps - wake	Jancey 2016	Office workers	3238.200	2841.958	3634.442	202.168	Blank
Steps - wake	Kozey-Keadley 2012	Office workers	6417.000	4941.813	7892.187	752.660	Blank
Steps - wake	Miller 2004 - O	Office workers	7580.590	7041.318	8119.863	275.144	Combined
Steps - wake	Mitsui 2010	Office workers	5236.000	4611.512	5860.488	318.622	Blank
Steps - wake	Pontt 2015 - O	Office workers	8886.000	7766.107	10005.893	571.385	Blank
Steps - wake	Rafferty 2016	Office workers	11008.000	9855.243	12160.757	588.152	Blank
Steps - wake	Schofield 2005 - O	Office workers	9200.000	8219.679	10180.321	500.173	Blank
Steps - wake	Smith 2002 - O	Office workers	5923.141	5458.364	6387.919	237.136	Combined
Steps - wake	Smith 2015	Office workers	9737.000	9198.732	10275.268	274.632	Blank
Steps - wake	Steeves 2018 - O	Office workers	6831.041	6800.858	6861.224	15.400	Combined
Steps - wake	Swartz 2014	Office workers	3574.000	3520.105	3627.895	27.498	Blank
Steps - wake	Taylor 2016	Office workers	6484.271	6009.836	6958.706	242.063	Combined
Steps - wake	Tigbe 2011 - O	Office workers	10853.000	10088.481	11617.519	390.068	Blank
Steps - wake	Tobin 2016	Office workers	2453.000	2122.406	2783.594	168.673	Blank
Steps - wake	Wieters 2009	Office workers	4596.155	4376.794	4815.515	111.921	Combined
			6857.366	6022.916	7691.816	425.748	

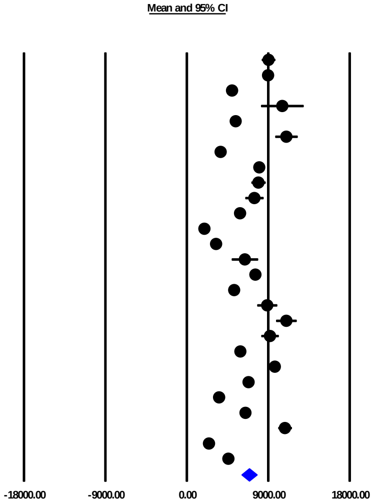

Steps per day during wake time

Supplemental figure 6g. Steps per day during wake time in all workers except office workers

Outcome	Study name	Comparison	Statistics for each study: Mean	Lower limit	Upper limit	Standard error	Subgroup within study
Steps - wake	Abdt, 2012	Healthcare workers	1999.000	1258.351	2739.649	377.889	Blank
Steps - wake	Bassey, 1983	Factory workers	11600.000	10620.019	12579.981	500.000	Blank
Steps - wake	Brett, 2017 - A	Academics	9264.200	7310.997	11217.403	996.551	Blank
Steps - wake	Brewer, 2016	Healthcare workers	3764.619	3415.000	4114.238	178.380	Combined
Steps - wake	Chan 2018	Healthcare workers	10620.000	9740.536	11499.464	448.714	Blank
Steps - wake	Chastin, 2009 - PD	Postal workers - delivery	17065.000	15809.618	18320.382	640.513	Blank
Steps - wake	Chau, 2016	Call centre workers	1642.252	1142.552	2141.952	254.954	Combined
Steps - wake	Cheung 2008	School teachers	11399.000	10733.095	12064.905	339.754	Blank
Steps - wake	Cheung, 2012	School teachers	11351.000	10853.691	11848.309	253.734	Blank
Steps - wake	Chitkara, 2014	Call centre workers	5361.000	4232.837	6489.163	575.604	Blank
Steps - wake	Choi 2010	Protective services	3220.635	3195.839	3245.430	12.651	Combined
Steps - wake	Croteau 2017	Healthcare workers	8446.000	7452.900	9439.100	506.693	Blank
Steps - wake	Gany 2014	Drivers	3731.900	3250.061	4213.739	245.841	Blank
Steps - wake	James 2009	Healthcare workers	10865.000	9684.870	12045.130	602.118	Blank
Steps - wake	Jirathananuwat 2017	Healthcare workers	11416.500	10943.181	11889.819	241.494	Blank
Steps - wake	Ju 2011	Laborers	8000.900	7443.974	8557.826	284.151	Blank
Steps - wake	Julin 2011	Laborers	15085.000	13300.033	16869.967	910.714	Blank
Steps - wake	Korshoj 2013	Laborers	20198.000	18170.165	22225.835	1034.629	Blank
Steps - wake	Mansi 2015 - F	Factory workers	5885.221	5575.930	6194.512	157.804	Combined
Steps - wake	Miller 2004 - A	Academics	10147.000	9395.839	10898.161	383.253	Scientists
Steps - wake	Miller 2004 - BC	Blue-collar workers	11784.000	9312.050	14255.950	1261.222	Blank
Steps - wake	Miller 2004 - tech	Technicians	10731.000	9936.037	11525.963	405.601	Blank
Steps - wake	Pontt 2015 - L	Laborers	11296.000	9859.101	12732.899	733.125	Blank
Steps - wake	Reed 2018 a	Healthcare workers	8176.000	7948.433	8403.567	116.108	Nurses
Steps - wake	Reed 2018b	Healthcare workers	8885.000	8076.535	9693.465	412.489	Nurses
Steps - wake	Schnebly 2017	Healthcare workers	9369.700	8471.845	10267.555	458.098	Blank
Steps - wake	Schofield 2005 - A	Academics	9702.000	8732.331	10671.669	494.738	Blank
Steps - wake	Schofield 2005 - allied uni	Allied university	4988.000	4103.383	5872.617	451.344	Blank
Steps - wake	Schofield 2005 - H	Healthcare workers	6691.000	4574.803	8807.197	1079.712	Blank
Steps - wake	Schofield 2005 - L	Laborers	14535.000	11027.971	18042.029	1789.333	Blank
Steps - wake	Schofield 2005 CS	Customer service - hospitality	13919.000	11628.238	16209.762	1168.778	Blank
Steps - wake	Smith 2002 - A	Academics	7138.537	6174.220	8102.854	492.007	Combined
Steps - wake	Smith 2002 - eng	Professional engineers	7947.000	6729.862	9164.138	621.000	Blank
Steps - wake	Smith 2002 - H	Healthcare workers	9564.000	8048.349	11079.651	773.306	Blank
Steps - wake	Smith 2002 - house	Housewives	5674.000	4158.058	7189.942	773.454	Blank
Steps - wake	Smith 2002 - PD	Postal workers - delivery	12713.000	11629.214	13796.786	552.962	Blank
Steps - wake	Smith 2002 - S	School teachers	7285.000	6362.912	8207.088	470.462	Blank
Steps - wake	Smith 2002 tech	Technicians	8866.000	7308.938	10423.062	794.434	Blank
Steps - wake	Steeves 2018 - ADE	Blank	6552.000	6361.799	6742.201	97.043	Arts, design, entertainment, sports, media
Steps - wake	Steeves 2018 - Arch	Blank	7119.000	6968.083	7269.917	77.000	Architecture, engineering
Steps - wake	Steeves 2018 - BF	Blank	7215.000	7122.067	7307.933	47.416	Business, financial operations
Steps - wake	Steeves 2018 - C	Customer service - hospitality	9388.000	9225.043	9550.957	83.143	Food preparation, serving
Steps - wake	Steeves 2018 - CM	Blank	6199.000	6056.598	6341.402	72.655	Computer, mathematical
Steps - wake	Steeves 2018 - CSS	Blank	5684.000	5463.307	5904.693	112.600	Community, social services
Steps - wake	Steeves 2018 - D	Drivers	9960.000	9879.729	10040.271	40.955	Transportation, material moving
Steps - wake	Steeves 2018 - Educ	Blank	7890.000	7804.315	7975.685	43.718	Education, training, library
Steps - wake	Steeves 2018 - H	Healthcare workers	6813.577	6739.430	6887.724	37.831	Combined
Steps - wake	Steeves 2018 - IMR	Blank	9004.000	8790.679	9217.321	108.839	Installation, maintenance, repair
Steps - wake	Steeves 2018 - L	Laborers	9596.625	9519.391	9673.860	39.406	Combined
Steps - wake	Steeves 2018 - Legal	Blank	5804.000	5200.865	6407.135	307.728	Legal
Steps - wake	Steeves 2018 - LPSS	Blank	6196.000	5966.680	6425.320	117.002	Life, physical, social science
Steps - wake	Steeves 2018 - P	Protective services	7109.000	6953.277	7264.723	79.452	Protective service
Steps - wake	Steeves 2018 - PCS	Blank	8339.000	8113.935	8564.065	114.831	Personal care service
Steps - wake	Steeves 2018 - Prod	Blank	8151.000	8103.084	8198.916	24.447	Production
Steps - wake	Steeves 2018 - Sales	Blank	7243.000	7182.661	7303.339	30.786	Sales & related
Steps - wake	Talbot 2011	Protective services	6699.573	6033.613	7365.533	339.782	Combined
Steps - wake	Tigbe 2011 - PD	Postal workers - delivery	18554.000	17321.446	19786.554	628.866	Blank
Steps - wake	Torquati 2018	Healthcare workers	8496.000	7773.270	9218.730	368.747	Nurses
			8745.190	7980.788	9509.592	390.008	

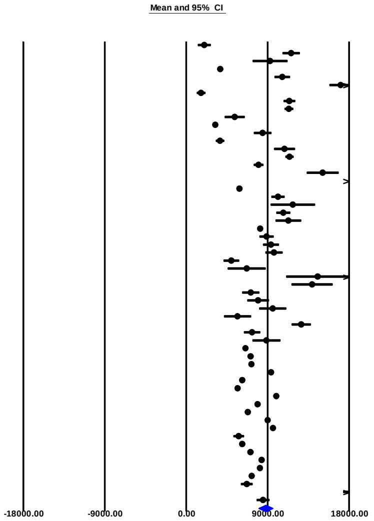

Steps per day during wake time

Supplemental figure 6h. Steps per day during wake time in healthcare workers

Outcome	Study name	Comparison	Statistics for each study				Subgroup within study
			Mean	Lower limit	Upper limit	Standard error	
Steps - wake	Abdt, 2012	Healthcare workers	1999.000	1258.351	2739.649	377.889	Blank
Steps - wake	Brewer, 2016	Healthcare workers	3764.619	3415.000	4114.238	178.380	Combined
Steps - wake	Chan 2018	Healthcare workers	10620.000	9740.536	11499.464	448.714	Blank
Steps - wake	Croteau 2017	Healthcare workers	8446.000	7452.900	9439.100	506.693	Blank
Steps - wake	James 2009	Healthcare workers	10865.000	9684.870	12045.130	602.118	Blank
Steps - wake	Jirathananuwat 2017	Healthcare workers	11416.500	10943.181	11889.819	241.494	Blank
Steps - wake	Reed 2018 a	Healthcare workers	8176.000	7948.433	8403.567	116.108	Nurses
Steps - wake	Reed 2018b	Healthcare workers	8885.000	8076.535	9693.465	412.489	Nurses
Steps - wake	Schnebly 2017	Healthcare workers	9369.700	8471.845	10267.555	458.098	Blank
Steps - wake	Schofield 2005 - H	Healthcare workers	6691.000	4574.803	8807.197	1079.712	Blank
Steps - wake	Smith 2002 - H	Healthcare workers	9564.000	8048.349	11079.651	773.306	Blank
Steps - wake	Steeves 2018 - H	Healthcare workers	6813.577	6739.430	6887.724	37.831	Combined
Steps - wake	Torquati 2018	Healthcare workers	8496.000	7773.270	9218.730	368.747	Nurses
			8071.589	6952.029	9191.149	571.215	

Mean and 95% CI

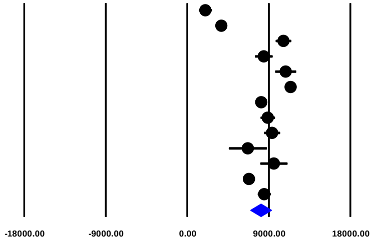

Steps per day during wake time

Supplemental figure 6i. Steps per day during wake time in academic workers

Outcome	Study name	Comparison	Statistics for each study				Subgroup within study
			Mean	Lower limit	Upper limit	Standard error	
Steps - wake	Brett, 2017 - A	Academics	9264.200	7310.997	11217.403	996.551	Blank
Steps - wake	Mller 2004 - A	Academics	10147.000	9395.839	10898.161	383.253	Scientists
Steps - wake	Schofield 2005 - A	Academics	9702.000	8732.331	10671.669	494.738	Blank
Steps - wake	Smith 2002 - A	Academics	7138.537	6174.220	8102.854	492.007	Combined
			9062.121	7562.422	10561.819	765.167	

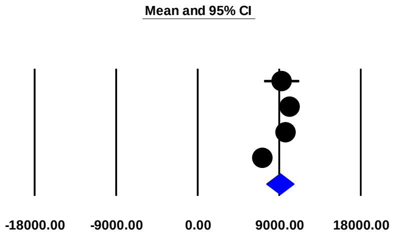

Steps per day during wake time

Supplemental figure 6j. Steps per day during wake time in school teachers

Outcome	Study name	Comparison	Statistics for each study				Subgroup within study
			Mean	Lower limit	Upper limit	Standard error	
Steps - wake	Cheung 2008	School teachers	11399.000	10733.095	12064.905	339.754	Blank
Steps - wake	Cheung, 2012	School teachers	11351.000	10853.691	11848.309	253.734	Blank
Steps - wake	Smith 2002 - S	School teachers	7285.000	6362.912	8207.088	470.462	Blank
			10043.056	7851.082	12235.030	1118.375	

Mean and 95% CI

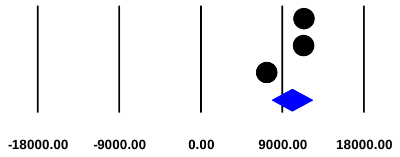

Steps per day during wake time

Supplemental figure 6k. Steps per day during wake time in laborers

Outcome	Study name	Comparison	Mean	Lower limit	Upper limit	Standard error	Subgroup within study
Steps - wake	Ju 2011	Laborers	8000.900	7443.974	8557.826	284.151	Blank
Steps - wake	Julin 2011	Laborers	15085.000	13300.033	16869.967	910.714	Blank
Steps - wake	Korshoj 2013	Laborers	20198.000	18170.165	22225.835	1034.629	Blank
Steps - wake	Pontt 2015 - L	Laborers	11296.000	9859.101	12732.899	733.125	Blank
Steps - wake	Schofield 2005 - L	Laborers	14535.000	11027.971	18042.029	1789.333	Blank
Steps - wake	Steeves 2018 - L	Laborers	9596.625	9519.391	9673.860	39.406	Combined
			12796.040	10782.968	14809.112	1027.096	

Mean and 95% CI

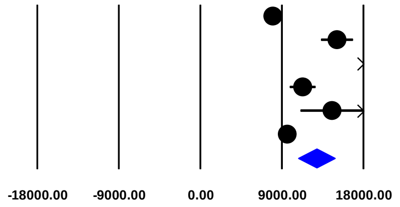

Steps per day during wake time

Supplemental figure 6l. Steps per day during wake time in postal delivery workers

Outcome	Study name	Comparison	Mean	Lower limit	Upper limit	Standard error	Subgroup within study
Steps - wake	Chastin, 2009 - PD	Postal workers - delivery	17065.000	15809.618	18320.382	640.513	Blank
Steps - wake	Smith 2002 - PD	Postal workers - delivery	12713.000	11629.214	13796.786	552.962	Blank
Steps - wake	Tigbe 2011 - PD	Postal workers - delivery	18554.000	17321.446	19786.554	628.866	Blank
			16099.971	12526.337	19673.605	1823.316	

Statistics for each study

Mean and 95% CI

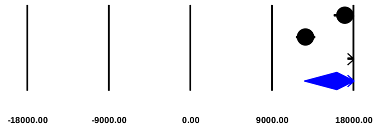

Steps per day during wake time

Supplemental figures 7a-j

Body mass index (BMI)

Supplemental figure 7a. BMI across all occupations

Study name	Subgroup within study	Comparison	Outcome	Mean	Standard error	Variance	Lower limit	Upper limit	Z-Value	p-Value
Aandstad 2018	Home guard soliders	Protective services	BMI - OM	26.100	0.183	0.033	25.742	26.458	143.008	0.000
Abd 2012	Physicians	Healthcare	BMI - SR	25.100	0.416	0.173	24.285	25.915	60.371	0.000
Alkajah 2012	Combined	Office	BMI - OM	22.160	0.477	0.228	21.225	23.095	46.452	0.000
Andersen 2011	Taxi drivers	Drivers	BMI - OM	27.800	0.299	0.089	27.214	28.386	93.049	0.000
Arias 2017	Patient care workers	Healthcare	BMI - unclear	26.000	0.583	0.340	24.856	27.144	44.562	0.000
Arias 2015	Construction workers	Laborers	BMI - unclear	29.000	0.539	0.291	27.943	30.057	53.767	0.000
Brakenridge 2016	Blank	Office	BMI - SR	24.600	0.275	0.076	24.061	25.139	89.496	0.000
Brewer 2016	Combined	Healthcare	BMI - unclear	25.684	0.607	0.369	24.494	26.874	42.298	0.000
Brown 2013	Combined	Office	BMI - OM	26.570	0.420	0.177	25.747	27.394	63.228	0.000
Carr 2016 a	Blank	Office	BMI - OM	30.100	1.103	1.217	27.938	32.262	27.287	0.000
Carr 2016 b	Blank	Office workers with sit-stand desks	BMI - OM	31.000	1.149	1.321	28.747	33.253	26.969	0.000
Chae 2015	Blank	Office	BMI - OM	23.260	0.403	0.162	22.471	24.049	57.747	0.000
Chan 2018	Blank	Healthcare workers	BMI - SR	23.300	0.414	0.172	22.488	24.112	56.241	0.000
Chappel 2016	Fire fighters	Protective services	BMI - SR	26.300	0.583	0.340	25.157	27.443	45.104	0.000
Chastin 2009 - PD	Blank	Postal workers - delivery	BMI - SR	27.000	0.641	0.410	25.745	28.255	42.154	0.000
Chastin 2009 - O	Postal workers - office	Office	BMI - SR	27.000	0.480	0.231	26.058	27.942	56.205	0.000
Chau 2016	Blank	Call centre workers	BMI - SR	26.800	0.988	0.976	24.864	28.736	27.130	0.000
Cheung 2008	Combined	School teachers	BMI - OM	21.638	0.441	0.194	20.774	22.503	49.079	0.000
Cheung 2012	Blank	School teachers	BMI - OM	22.300	0.451	0.203	21.416	23.184	49.448	0.000
Chitkara 2014	Blank	Call centre workers	BMI - OM	29.900	1.491	2.224	26.977	32.823	20.051	0.000
Clemes 2014a	Blank	Office	BMI - OM	24.000	0.412	0.170	23.192	24.808	58.185	0.000
Clemes 2014b	Blank	Office	BMI - OM	24.000	0.291	0.085	23.429	24.571	82.348	0.000
Croteau 2018	Blank	Healthcare workers	BMI - unclear	27.400	0.917	0.841	25.603	29.197	29.887	0.000
De Jong 2018	Blank	Office	BMI - OM	30.400	0.115	0.013	30.175	30.625	265.021	0.000
Dollman 2016 - O	Blank	Office	BMI - OM	28.390	0.610	0.373	27.194	29.586	46.510	0.000
Dollman 2016 - L	Farmers	Laborers	BMI - OM	29.870	0.813	0.662	28.276	31.464	36.725	0.000
Donath 2015	Combined	Office workers with sit-stand desks	BMI - unclear	24.216	0.786	0.618	22.676	25.756	30.813	0.000
Evans 2012	Combined	Office	BMI - unclear	23.650	0.588	0.346	22.498	24.802	40.232	0.000
Finkelstein 2015	Combined	Office	BMI - OM	24.134	0.148	0.022	23.843	24.424	162.819	0.000
Fisher 2018	Blank	Office	BMI - OM	25.640	0.392	0.154	24.871	26.409	65.359	0.000
French 2007	Transit workers	Drivers	BMI - OM	32.200	0.221	0.049	31.767	32.633	145.762	0.000
Gao 2016	Blank	Office	BMI - OM	23.700	0.748	0.560	22.233	25.167	31.670	0.000
Gilson 2012a	Blank	Office	BMI - unclear	27.100	0.303	0.092	26.507	27.693	89.508	0.000
Gilson 2012b	Combined	Office	BMI - SR	25.964	1.073	1.151	23.861	28.066	24.202	0.000
Gilson 2016	Total	Office	BMI - OM	28.000	0.662	0.439	26.702	29.298	42.279	0.000
Gilson 2017	Truck drivers	Drivers	BMI - OM	31.200	1.055	1.114	29.132	33.268	29.565	0.000
Hallman 2017	Blank	Blue collar workers (cleaning, transportation, manufacturing)	BMI - OM	27.600	0.196	0.038	27.216	27.984	140.816	0.000
Hallman 2018	Combined	Office	BMI - SR	25.410	0.422	0.178	24.584	26.236	60.261	0.000
Healy 2013	Blank	Office	BMI - OM	26.800	0.823	0.678	25.186	28.414	32.544	0.000
Heay 2016	Blank	Office	BMI - OM	28.610	0.400	0.160	27.826	29.394	71.519	0.000
Jirathananuwat 2017	Nurses	Healthcare	BMI - unclear	22.480	0.243	0.059	22.004	22.956	92.533	0.000
Kirk 2016	Blank	Office	BMI - unclear	25.800	0.761	0.580	24.308	27.292	33.887	0.000
Kloster 2017	Blank	Office	BMI - OM	26.000	0.275	0.076	25.461	26.539	94.473	0.000
Korshoj 2013	Cleaners	Laborers	BMI - OM	25.800	1.118	1.250	23.609	27.991	23.076	0.000
Kozey-Keadle 2012	Blank	Office	BMI - unclear	33.700	1.252	1.568	31.246	36.154	26.913	0.000
Li 2017	Blank	Office	BMI - NR	25.000	0.894	0.800	23.247	26.753	27.951	0.000
Lunde 2017 - H	Blank	Healthcare	BMI - SR	25.100	0.479	0.229	24.162	26.038	52.428	0.000
Lunde 2017 - L	Construction workers	Laborers	BMI - SR	25.700	0.423	0.179	24.872	26.528	60.825	0.000
Mansi 2015	Combined	Laborers	BMI - OM	28.967	3.039	9.234	23.011	34.922	9.532	0.000
Mansoubi 2016	Combined	Office	BMI - OM	25.020	0.688	0.473	23.672	26.368	36.381	0.000
Mitsui 2010	Blank	Office	BMI - OM	24.000	0.481	0.231	23.058	24.942	49.913	0.000
Neil-Sztramko 2017	Physiotherapists	Healthcare	BMI - SR	22.300	0.470	0.221	21.378	23.222	47.402	0.000
Neuhaus 2014	Blank	Office	BMI - OM	26.000	0.769	0.591	24.493	27.507	33.817	0.000
Parry 2013a	Blank	Office	BMI - unclear	24.700	0.580	0.336	23.564	25.836	42.599	0.000
Parry 2013b	Blank	Office	BMI - OM	28.000	0.813	0.661	26.407	29.593	34.449	0.000
Peavler 2012	Combined	Office	BMI - OM	32.338	0.749	0.560	30.870	33.805	43.198	0.000
Pedersen 2016	Blank	Office	BMI - unclear	26.000	0.404	0.163	25.208	26.792	64.320	0.000
Reed 2018	Combined	Healthcare	BMI - OM	27.134	0.267	0.071	26.610	27.657	101.561	0.000
Reed 2018b	Combined	Healthcare	BMI - OM	27.555	0.643	0.414	26.295	28.816	42.844	0.000
Ryan 2011 - O	Administrators	Office	BMI - unclear	27.000	1.045	1.091	24.952	29.048	25.845	0.000
Ryan 2011 - T	Blank	Technicians	BMI - unclear	23.500	0.907	0.823	21.722	25.278	25.906	0.000
Ryan 2011 - A	Combined	Academics	BMI - unclear	24.300	0.446	0.199	23.425	25.175	54.426	0.000
Ryde 2013	Blank	Office	BMI - OM	26.100	0.381	0.145	25.354	26.846	68.576	0.000
Sawyer 2017	Blank	Office	BMI - OM	25.800	0.420	0.176	24.978	26.622	61.483	0.000
Schwartz 2016	Blank	Office	BMI - OM	29.200	0.802	0.643	27.628	30.772	36.403	0.000
Smith 2018	Blank	Office	BMI - OM	25.820	0.419	0.175	24.999	26.641	61.661	0.000
Straker 2013	Blank	Call centre workers	BMI - unclear	24.500	0.349	0.122	23.815	25.185	70.104	0.000
Swartz 2014	Office workers - clerical	Office	BMI - OM	28.500	0.955	0.913	26.628	30.372	29.832	0.000
Talbot 2011	Combined	Protective services	BMI - OM	29.187	0.467	0.218	28.272	30.102	62.507	0.000
Tigbe 2011 - PD	Blank	Postal workers - delivery	BMI - unclear	26.300	0.401	0.161	25.514	27.086	65.604	0.000
Tigbe 2011	Postal workers - office	Office	BMI - unclear	27.400	0.535	0.286	26.352	28.448	51.261	0.000
Toomingas 2012	Blank	Call centre workers	BMI - SR	24.400	0.319	0.102	23.776	25.024	76.579	0.000
Torquati 2018	Nurses	Healthcare	BMI - OM	28.000	0.875	0.766	26.285	29.715	31.993	0.000
Urda 2016	Blank	Office	BMI - OM	30.500	1.236	1.528	28.077	32.923	24.672	0.000
Varela Mato 2017	Bus drivers	Drivers	BMI - OM	28.500	0.737	0.543	27.055	29.945	38.669	0.000
Varela Mato 2016	Lorry drivers	Drivers	BMI - OM	28.000	0.570	0.325	26.883	29.117	49.140	0.000
Vincent 2016	Fire fighters	Protective services	BMI - SR	26.800	0.743	0.552	25.343	28.257	36.063	0.000
Weiler 2015	Blank	Professional football players	BMI - unclear	23.900	0.260	0.068	23.390	24.410	91.923	0.000
Wick 2016	Blank	Office workers with sit-stand desks	BMI - OM	23.900	0.681	0.464	22.565	25.235	35.078	0.000
Wong 2014	Transport drivers	Drivers	BMI - unclear	33.700	1.641	2.693	30.484	36.916	20.536	0.000
Zhu 2018	Combined	Office	BMI - OM	25.800	0.801	0.642	24.230	27.370	32.212	0.000
				26.519	0.044	0.002	26.433	26.605	604.997	0.000

Mean and 95% CI

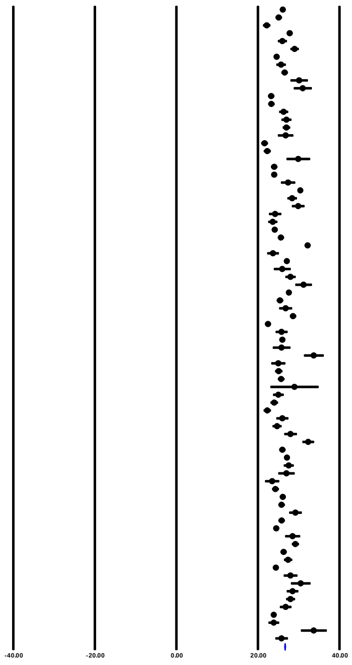

BMI

Supplemental figure 7b. Funnel plot for BMI across all occupations

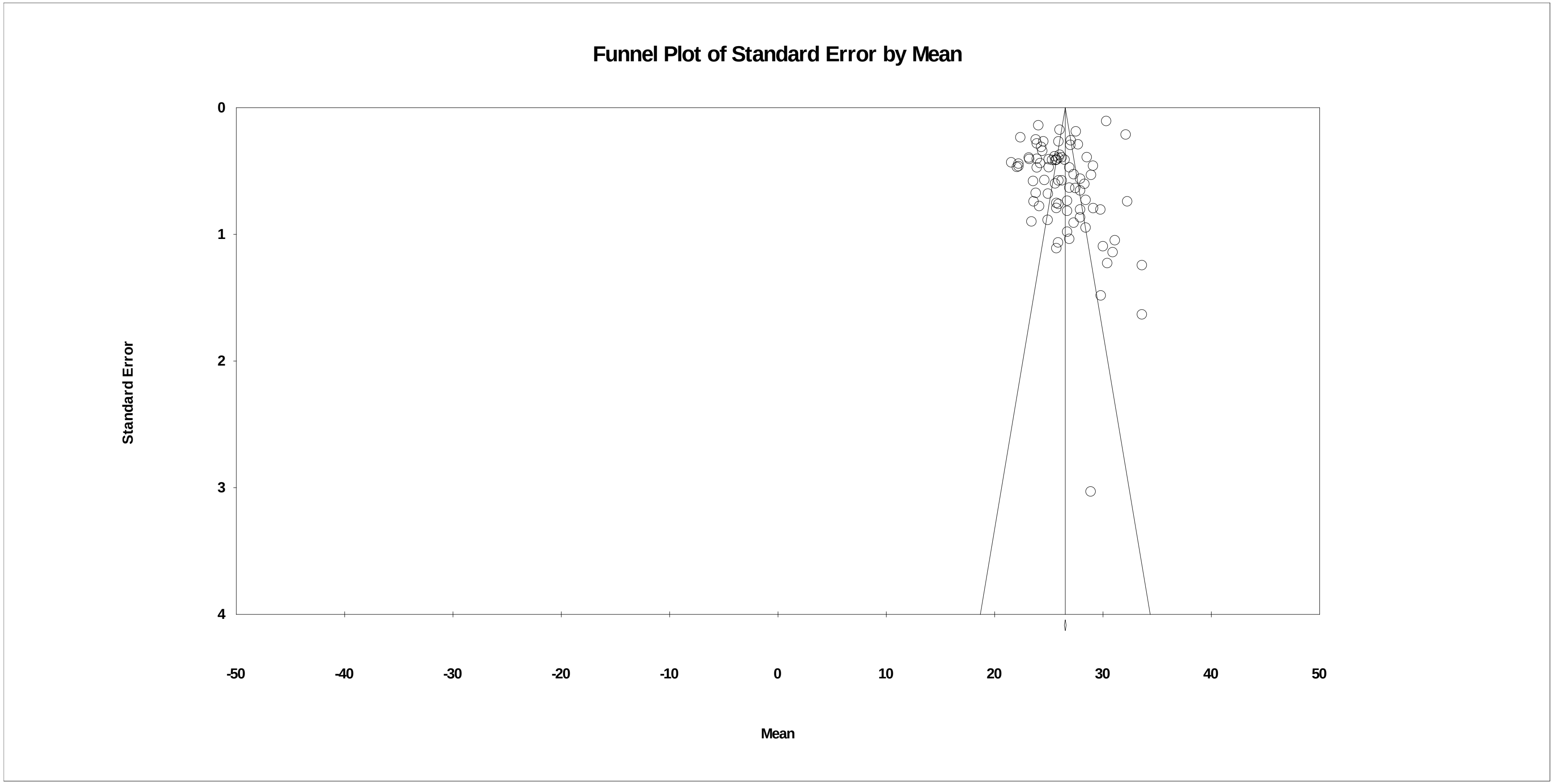

Supplemental figure 7c. BMI in drivers

Study name	Subgroup within study	Comparison	Outcome	Statistics for each study						
				Mean	Standard error	Variance	Lower limit	Upper limit	Z-Value	p-Value
Andersen 2011	Taxi drivers	Drivers	BMI - OM	27.800	0.299	0.089	27.214	28.386	93.049	0.000
French 2007	Transit workers	Drivers	BMI - OM	32.200	0.221	0.049	31.767	32.633	145.762	0.000
Gilson 2017	Truck drivers	Drivers	BMI - OM	31.200	1.055	1.114	29.132	33.268	29.565	0.000
Varela Mato 2016	Lorry drivers	Drivers	BMI - OM	28.000	0.570	0.325	26.883	29.117	49.140	0.000
Varela Mato 2017	Bus drivers	Drivers	BMI - OM	28.500	0.737	0.543	27.055	29.945	38.669	0.000
Wong 2014	Transport drivers	Drivers	BMI - unclear	33.700	1.641	2.693	30.484	36.916	20.536	0.000
				30.080	1.147	1.315	27.832	32.327	26.226	0.000

Mean and 95% CI

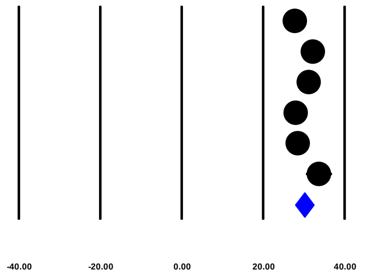

BMI

Supplemental figure 7d. BMI in protective service workers

Study name	Subgroup within study	Comparison	Outcome	Statistics for each study						
				Mean	Standard error	Variance	Lower limit	Upper limit	Z-Value	p-Value
Aandstad 2018	Home guard soliders	Protective services	BMI - OM	26.100	0.183	0.033	25.742	26.458	143.008	0.000
Chappel 2016	Fire fighters	Protective services	BMI - SR	26.300	0.583	0.340	25.157	27.443	45.104	0.000
Talbot 2011	Combined	Protective services	BMI - OM	29.187	0.467	0.218	28.272	30.102	62.507	0.000
Vincent 2016	Fire fighters	Protective services	BMI - SR	26.800	0.743	0.552	25.343	28.257	36.063	0.000
				27.095	0.795	0.631	25.538	28.652	34.102	0.000

Mean and 95% CI

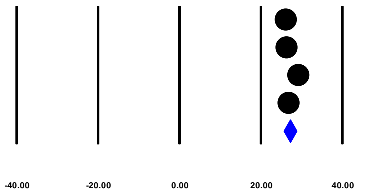

BMI

Supplemental figure 7e. BMI in call centre workers

Study name	Subgroup within study	Comparison	Outcome	Mean	Standard error	Variance	Lower limit	Upper limit	Z-Value	p-Value
Chau 2016	Blank	Call centre workers	BMI - SR	26.800	0.988	0.976	24.864	28.736	27.130	0.000
Chitkara 2014	Blank	Call centre workers	BMI - OM	29.900	1.491	2.224	26.977	32.823	20.051	0.000
Straker 2013	Blank	Call centre workers	BMI - unclear	24.500	0.349	0.122	23.815	25.185	70.104	0.000
Toomingas 2012	Blank	Call centre workers	BMI - SR	24.400	0.319	0.102	23.776	25.024	76.579	0.000
				25.670	0.694	0.481	24.310	27.029	37.014	0.000

Statistics for each study

Mean and 95% CI

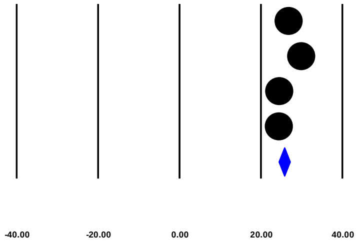

BMI

Supplemental figure 7f. BMI in office workers

Study name	Subgroup within study	Comparison	Outcome	Mean	Standard error	Variance	Lower limit	Upper limit	Z-Value	p-Value
Alkajah 2012	Combined	Office	BMI - OM	22.160	0.477	0.228	21.225	23.095	46.452	0.000
Brakenridge 2016	Blank	Office	BMI - SR	24.600	0.275	0.076	24.061	25.139	89.496	0.000
Brown 2013	Combined	Office	BMI - OM	26.570	0.420	0.177	25.747	27.394	63.228	0.000
Carr 2016 a	Blank	Office	BMI - OM	30.100	1.103	1.217	27.938	32.262	27.287	0.000
Chae 2015	Blank	Office	BMI - OM	23.260	0.403	0.162	22.471	24.049	57.747	0.000
Chastin 2009 - O	Postal workers - office	Office	BMI - SR	27.000	0.480	0.231	26.058	27.942	56.205	0.000
Clemes 2014a	Blank	Office	BMI - OM	24.000	0.412	0.170	23.192	24.808	58.185	0.000
Clemes 2014b	Blank	Office	BMI - OM	24.000	0.291	0.085	23.429	24.571	82.348	0.000
De Jong 2018	Blank	Office	BMI - OM	30.400	0.115	0.013	30.175	30.625	265.021	0.000
Dollman 2016 - O	Blank	Office	BMI - OM	28.390	0.610	0.373	27.194	29.586	46.510	0.000
Evans 2012	Combined	Office	BMI - unclear	23.650	0.588	0.346	22.498	24.802	40.232	0.000
Finkelstein 2015	Combined	Office	BMI - OM	24.134	0.148	0.022	23.843	24.424	162.819	0.000
Fisher 2018	Blank	Office	BMI - OM	25.640	0.392	0.154	24.871	26.409	65.359	0.000
Gao 2016	Blank	Office	BMI - OM	23.700	0.748	0.560	22.233	25.167	31.670	0.000
Gilson 2012a	Blank	Office	BMI - unclear	27.100	0.303	0.092	26.507	27.693	89.508	0.000
Gilson 2012b	Combined	Office	BMI - SR	25.964	1.073	1.151	23.861	28.066	24.202	0.000
Gilson 2016	Total	Office	BMI - OM	28.000	0.662	0.439	26.702	29.298	42.279	0.000
Hallman 2018	Combined	Office	BMI - SR	25.410	0.422	0.178	24.584	26.236	60.261	0.000
Healy 2013	Blank	Office	BMI - OM	26.800	0.823	0.678	25.186	28.414	32.544	0.000
Heay 2016	Blank	Office	BMI - OM	28.610	0.400	0.160	27.826	29.394	71.519	0.000
Kirk 2016	Blank	Office	BMI - unclear	25.800	0.761	0.580	24.308	27.292	33.887	0.000
Kloster 2017	Blank	Office	BMI - OM	26.000	0.275	0.076	25.461	26.539	94.473	0.000
Kozey-Keadle 2012	Blank	Office	BMI - unclear	33.700	1.252	1.568	31.246	36.154	26.913	0.000
Li 2017	Blank	Office	BMI - NR	25.000	0.894	0.800	23.247	26.753	27.951	0.000
Mansoubi 2016	Combined	Office	BMI - OM	25.020	0.688	0.473	23.672	26.368	36.381	0.000
Mitsui 2010	Blank	Office	BMI - OM	24.000	0.481	0.231	23.058	24.942	49.913	0.000
Neuhaus 2014	Blank	Office	BMI - OM	26.000	0.769	0.591	24.493	27.507	33.817	0.000
Parry 2013a	Blank	Office	BMI - unclear	24.700	0.580	0.336	23.564	25.836	42.599	0.000
Parry 2013b	Blank	Office	BMI - OM	28.000	0.813	0.661	26.407	29.593	34.449	0.000
Peavler 2012	Combined	Office	BMI - OM	32.338	0.749	0.560	30.870	33.805	43.198	0.000
Pedersen 2016	Blank	Office	BMI - unclear	26.000	0.404	0.163	25.208	26.792	64.320	0.000
Ryan 2011 - O	Administrators	Office	BMI - unclear	27.000	1.045	1.091	24.952	29.048	25.845	0.000
Ryde 2013	Blank	Office	BMI - OM	26.100	0.381	0.145	25.354	26.846	68.576	0.000
Sawyer 2017	Blank	Office	BMI - OM	25.800	0.420	0.176	24.978	26.622	61.483	0.000
Schwartz 2016	Blank	Office	BMI - OM	29.200	0.802	0.643	27.628	30.772	36.403	0.000
Smith 2018	Blank	Office	BMI - OM	25.820	0.419	0.175	24.999	26.641	61.661	0.000
Swartz 2014	Office workers - clerical	Office	BMI - OM	28.500	0.955	0.913	26.628	30.372	29.832	0.000
Tigbe 2011	Postal workers - office	Office	BMI - unclear	27.400	0.535	0.286	26.352	28.448	51.261	0.000
Urda 2016	Blank	Office	BMI - OM	30.500	1.236	1.528	28.077	32.923	24.672	0.000
Zhu 2018	Combined	Office	BMI - OM	25.800	0.801	0.642	24.230	27.370	32.212	0.000
				26.495	0.455	0.207	25.603	27.388	58.178	0.000

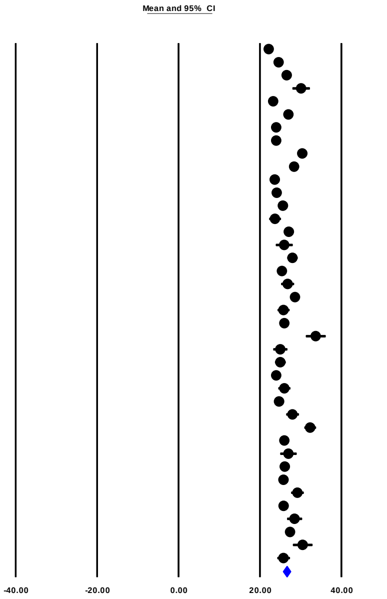

BMI

Supplemental figure 7g. BMI in office workers with sit-stand desks

Study name	Subgroup within study	Comparison	Outcome	Statistics for each study						
				Mean	Standard error	Variance	Lower limit	Upper limit	Z-Value	p-Value
Carr 2016 b	Blank	Office workers with sit-stand desks	BMI - OM	31.000	1.149	1.321	28.747	33.253	26.969	0.000
Donath 2015	Combined	Office workers with sit-stand desks	BMI - unclear	24.216	0.786	0.618	22.676	25.756	30.813	0.000
Gao 2016	Blank	Office workers with sit-stand desks	BMI - OM	23.200	1.075	1.156	21.093	25.307	21.578	0.000
Wick 2016	Blank	Office workers with sit-stand desks	BMI - OM	23.900	0.681	0.464	22.565	25.235	35.078	0.000
				25.502	1.493	2.228	22.577	28.428	17.086	0.000

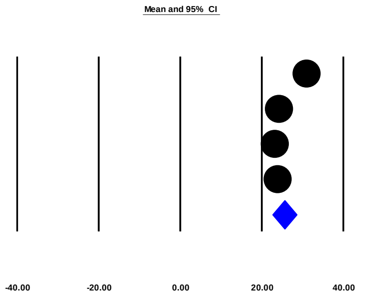

BMI

Supplemental figure 7h. BMI in laborers

Study name	Subgroup within study	Comparison	Outcome	Statistics for each study						
				Mean	Standard error	Variance	Lower limit	Upper limit	Z-Value	p-Value
Arias 2015	Construction workers	Laborers	BMI - unclear	29.000	0.539	0.291	27.943	30.057	53.767	0.000
Dollman 2016 - L	Farmers	Laborers	BMI - OM	29.870	0.813	0.662	28.276	31.464	36.725	0.000
Korshoj 2013	Cleaners	Laborers	BMI - OM	25.800	1.118	1.250	23.609	27.991	23.076	0.000
Lunde 2017 - L	Construction workers	Laborers	BMI - SR	25.700	0.423	0.179	24.872	26.528	60.825	0.000
Mansi 2015	Combined	Laborers	BMI - OM	28.967	3.039	9.234	23.011	34.922	9.532	0.000
				27.729	1.061	1.127	25.648	29.809	26.124	0.000

Mean and 95% CI

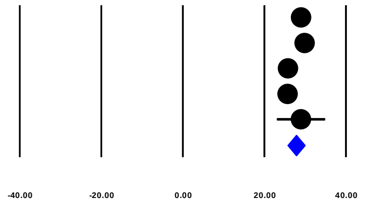

BMI

Supplemental figure 7i. BMI in healthcare workers

Study name	Subgroup within study	Comparison	Outcome	Statistics for each study						
				Mean	Standard error	Variance	Lower limit	Upper limit	Z-Value	p-Value
Abd 2012	Physicians	Healthcare	BMI - SR	25.100	0.416	0.173	24.285	25.915	60.371	0.000
Arias 2017	Patient care workers	Healthcare	BMI - unclear	26.000	0.583	0.340	24.856	27.144	44.562	0.000
Brewer 2016	Combined	Healthcare	BMI - unclear	25.684	0.607	0.369	24.494	26.874	42.298	0.000
Chan 2018	Blank	Healthcare workers	BMI - SR	23.300	0.414	0.172	22.488	24.112	56.241	0.000
Croteau 2018	Blank	Healthcare workers	BMI - unclear	27.400	0.917	0.841	25.603	29.197	29.887	0.000
Jirathananuwat 2017	Nurses	Healthcare	BMI - unclear	22.480	0.243	0.059	22.004	22.956	92.533	0.000
Lunde 2017 - H	Blank	Healthcare	BMI - SR	25.100	0.479	0.229	24.162	26.038	52.428	0.000
Neil-Sztramko 2017	Physiotherapists	Healthcare	BMI - SR	22.300	0.470	0.221	21.378	23.222	47.402	0.000
Reed 2018	Combined	Healthcare	BMI - OM	27.134	0.267	0.071	26.610	27.657	101.561	0.000
Reed 2018b	Combined	Healthcare	BMI - OM	27.555	0.643	0.414	26.295	28.816	42.844	0.000
Torquati 2018	Nurses	Healthcare	BMI - OM	28.000	0.875	0.766	26.285	29.715	31.993	0.000
				24.740	0.127	0.016	24.491	24.988	194.812	0.000

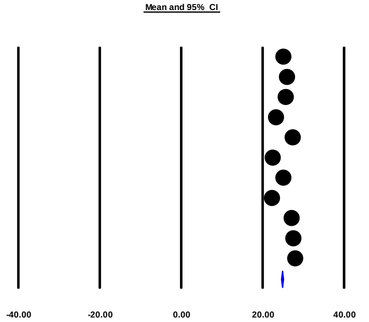

BMI

Supplemental figure 7j. Funnel plot for BMI across healthcare workers

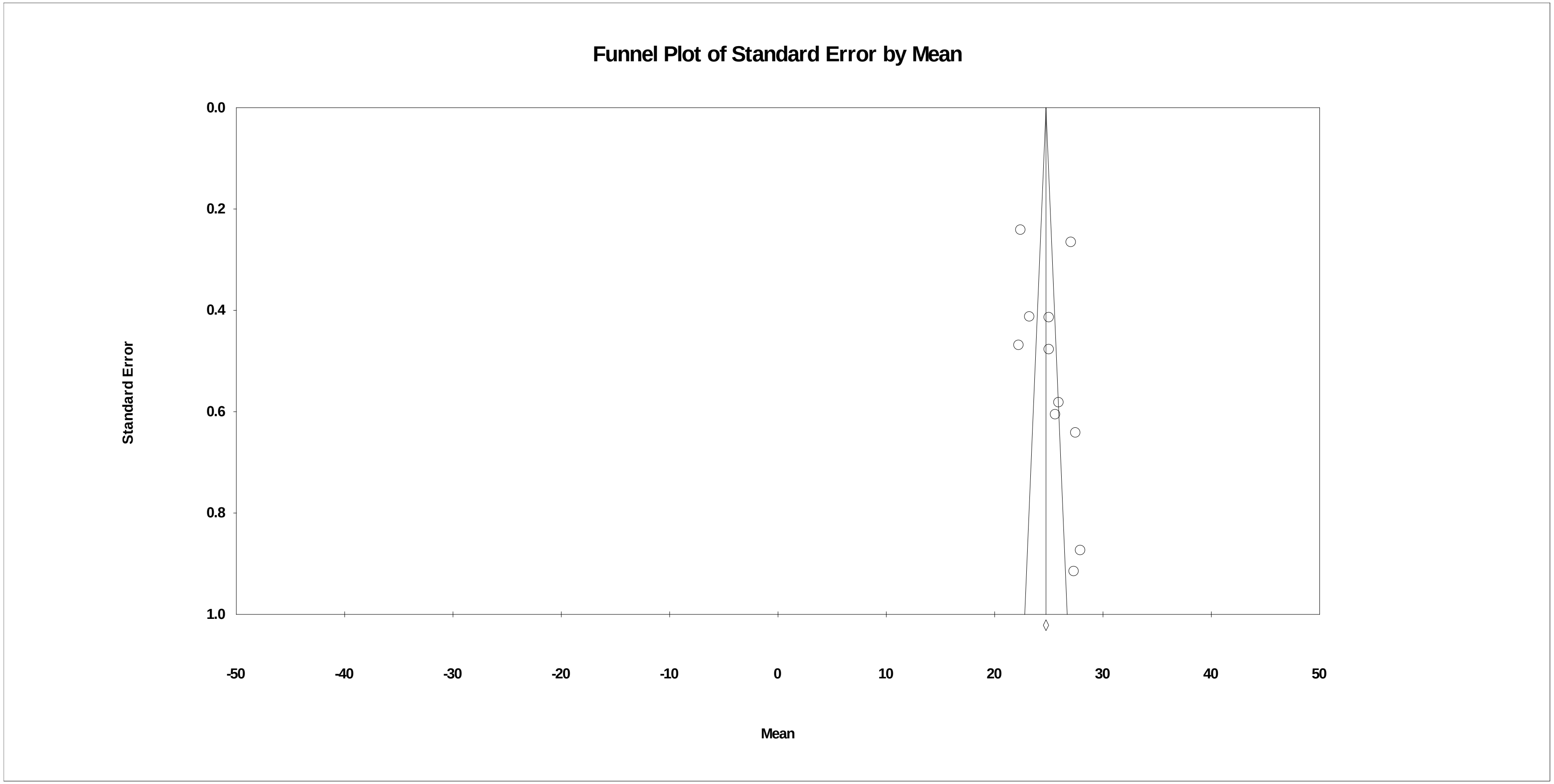

Supplemental figures 8a-d

Waist circumference

Supplemental figure 8a. Waist circumference across all occupations

Study name	Subgroup within study	Comparison	Outcome	Statistics for each study: Mean	Standard error	Variance	Lower limit	Upper limit	Z-Value	p-Value
Abd 2012	Physicians	Healthcare	WC - SR	89.410	1.344	1.805	86.776	92.044	66.542	0.000
Alkajah 2012	Combined	Office	WC - OM	81.820	1.609	2.588	78.667	84.973	50.858	0.000
Andersen 2011	Taxi drivers	Drivers	WC - OM	100.000	0.815	0.664	98.403	101.597	122.726	0.000
Brown 2013	Combined	Office	WC - OM	84.211	1.488	2.213	81.295	87.127	56.607	0.000
Carr 2016 a	Blank	Office	WC - OM	97.800	2.417	5.842	93.063	102.537	40.462	0.000
Carr 2016 b	Blank	Office workers with sit-stand desks	WC - OM	100.600	3.251	10.568	94.228	106.972	30.946	0.000
Croteau 2018	Blank	Healthcare workers	WC - unclear	89.662	2.840	8.065	84.096	95.228	31.573	0.000
Gilson 2016	Combined	Office	WC - OM	96.070	1.890	3.571	92.367	99.774	50.841	0.000
Gilson 2017	Truck drivers	Drivers	WC - OM	109.000	2.294	5.263	104.504	113.496	47.512	0.000
He 2013	Combined	Healthcare	WC - OM	98.696	2.453	6.019	93.888	103.505	40.228	0.000
Koepp 2013	Blank	Office	WC - OM	95.000	3.167	10.028	88.793	101.207	30.000	0.000
Korshoj 2013	Cleaners	Laborers	WC - OM	85.500	3.332	11.101	78.970	92.030	25.662	0.000
Lagersted-Olsen 2014	Combined	Office	WC - OM	83.831	2.188	4.787	79.543	88.119	38.316	0.000
Mansi 2015	Combined	Laborers	WC - OM	95.750	2.527	6.386	90.797	100.703	37.890	0.000
Mansoubi 2016	Combined	Office	WC - OM	80.220	1.726	2.980	76.837	83.603	46.471	0.000
Reed 2018	Combined	Healthcare	WC - OM	80.799	0.582	0.339	79.659	81.940	138.867	0.000
Reed 2018b	Combined	Healthcare	WC - OM	84.187	1.445	2.087	81.355	87.018	58.274	0.000
Ryde 2013	Blank	Office	WC - OM	83.800	1.113	1.238	81.619	85.981	75.324	0.000
Torquati 2018	Nurses	Healthcare	WC - OM	87.000	1.896	3.596	83.283	90.717	45.880	0.000
Waters 2016	Combined	Office	WC - OM	83.149	2.041	4.168	79.147	87.150	40.730	0.000
				90.204	1.956	3.824	86.372	94.037	46.128	0.000

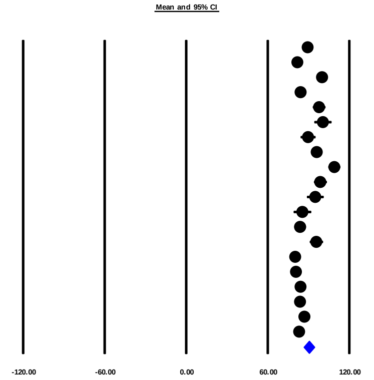

BMI

Supplemental figure 8b. Funnel plot for waist circumference across all occupations

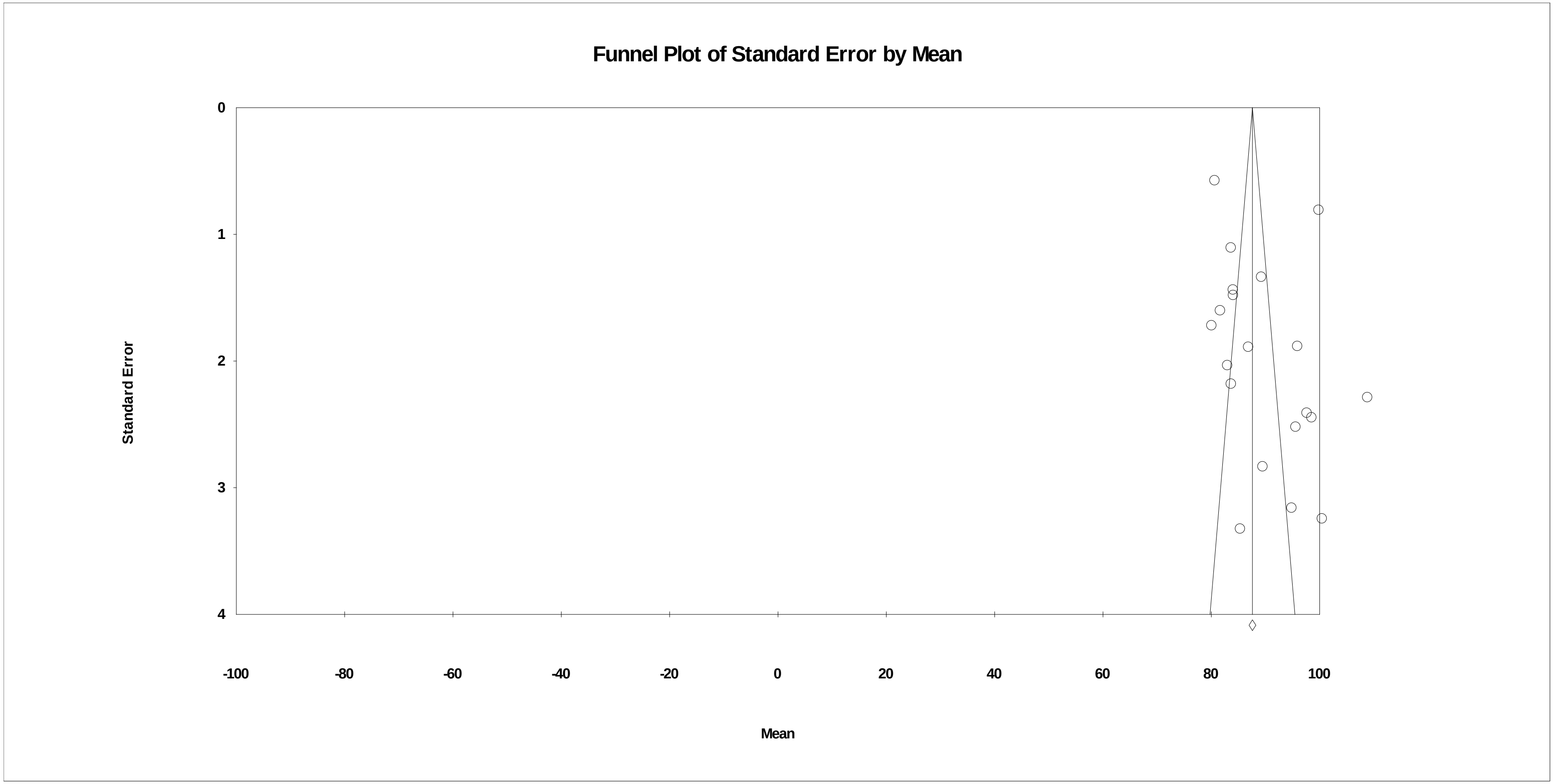

Supplemental figure 8c. Waist circumference across all healthcare workers

Study name	Subgroup within study	Comparison	Outcome	Statistics for each study						
				Mean	Standard error	Variance	Lower limit	Upper limit	Z-Value	p-Value
Abd 2012	Physicians	Healthcare	WC - SR	89.410	1.344	1.805	86.776	92.044	66.542	0.000
Croteau 2018	Blank	Healthcare workers	WC - unclear	89.662	2.840	8.065	84.096	95.228	31.573	0.000
He 2013	Combined	Healthcare	WC - OM	98.696	2.453	6.019	93.888	103.505	40.228	0.000
Reed 2018	Combined	Healthcare	WC - OM	80.799	0.582	0.339	79.659	81.940	138.867	0.000
Reed 2018b	Combined	Healthcare	WC - OM	84.187	1.445	2.087	81.355	87.018	58.274	0.000
Torquati 2018	Nurses	Healthcare	WC - OM	87.000	1.896	3.596	83.283	90.717	45.880	0.000
				88.020	2.469	6.094	83.181	92.858	35.655	0.000

Mean and 95% CI

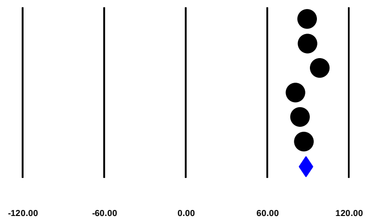

Waist circumference

Supplemental figure 8d. Waist circumference across office workers

Study name	Subgroup within study	Comparison	Outcome	Mean	Standard error	Variance	Lower limit	Upper limit	Z-Value	p-Value
Alkajah 2012	Combined	Office	WC - OM	81.820	1.609	2.588	78.667	84.973	50.858	0.000
Brown 2013	Combined	Office	WC - OM	84.211	1.488	2.213	81.295	87.127	56.607	0.000
Carr 2016 a	Blank	Office	WC - OM	97.800	2.417	5.842	93.063	102.537	40.462	0.000
Gilson 2016	Combined	Office	WC - OM	96.070	1.890	3.571	92.367	99.774	50.841	0.000
Koepp 2013	Blank	Office	WC - OM	95.000	3.167	10.028	88.793	101.207	30.000	0.000
Lagersted-Olsen 2014	Combined	Office	WC - OM	83.831	2.188	4.787	79.543	88.119	38.316	0.000
Mansoubi 2016	Combined	Office	WC - OM	80.220	1.726	2.980	76.837	83.603	46.471	0.000
Ryde 2013	Blank	Office	WC - OM	83.800	1.113	1.238	81.619	85.981	75.324	0.000
Waters 2016	Combined	Office	WC - OM	83.149	2.041	4.168	79.147	87.150	40.730	0.000
				87.058	1.964	3.856	83.209	90.906	44.336	0.000

Mean and 95% CI

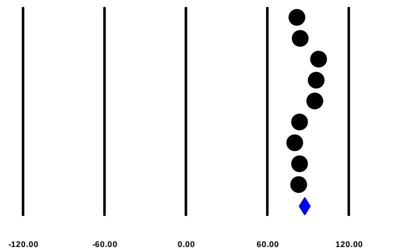

Waist circumference

Supplemental figures 9a-d

Wais-to-hip ratio (WHR)

Supplemental figure 9a. WHR across all occupations

Study name	Subgroup within study	Comparison	Outcome	Statistics for each study							Mean and 95% CI
				Mean	Standard error	Variance	Lower limit	Upper limit	Z-Value	p-Value	
Chae 2015	Blank	Office	WHR	0.860	0.007	0.000	0.846	0.874	119.921	0.000	
Cheung 2008	Women	School teachers	WHR	0.800	0.016	0.000	0.769	0.831	51.225	0.000	
Cheung 2012	Blank	School teachers	WHR	0.790	0.009	0.000	0.771	0.809	83.208	0.000	
Gao 2016	Blank	Office	WHR	0.840	0.018	0.000	0.805	0.875	46.910	0.000	
Korshoj 2013	Cleaners	Laborers	WHR`	0.820	0.025	0.001	0.772	0.868	33.338	0.000	
Lagersted-Olsen 2014	Combined	Office	WHR`	0.813	0.014	0.000	0.786	0.841	57.996	0.000	
				0.821	0.015	0.000	0.791	0.850	54.748	0.000	

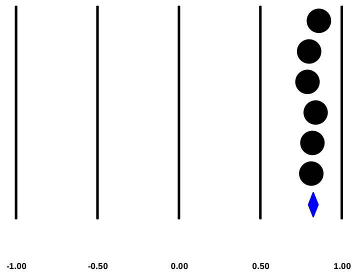

WHR

Supplemental figure 9b. Funnel plot for WHR across all occupations

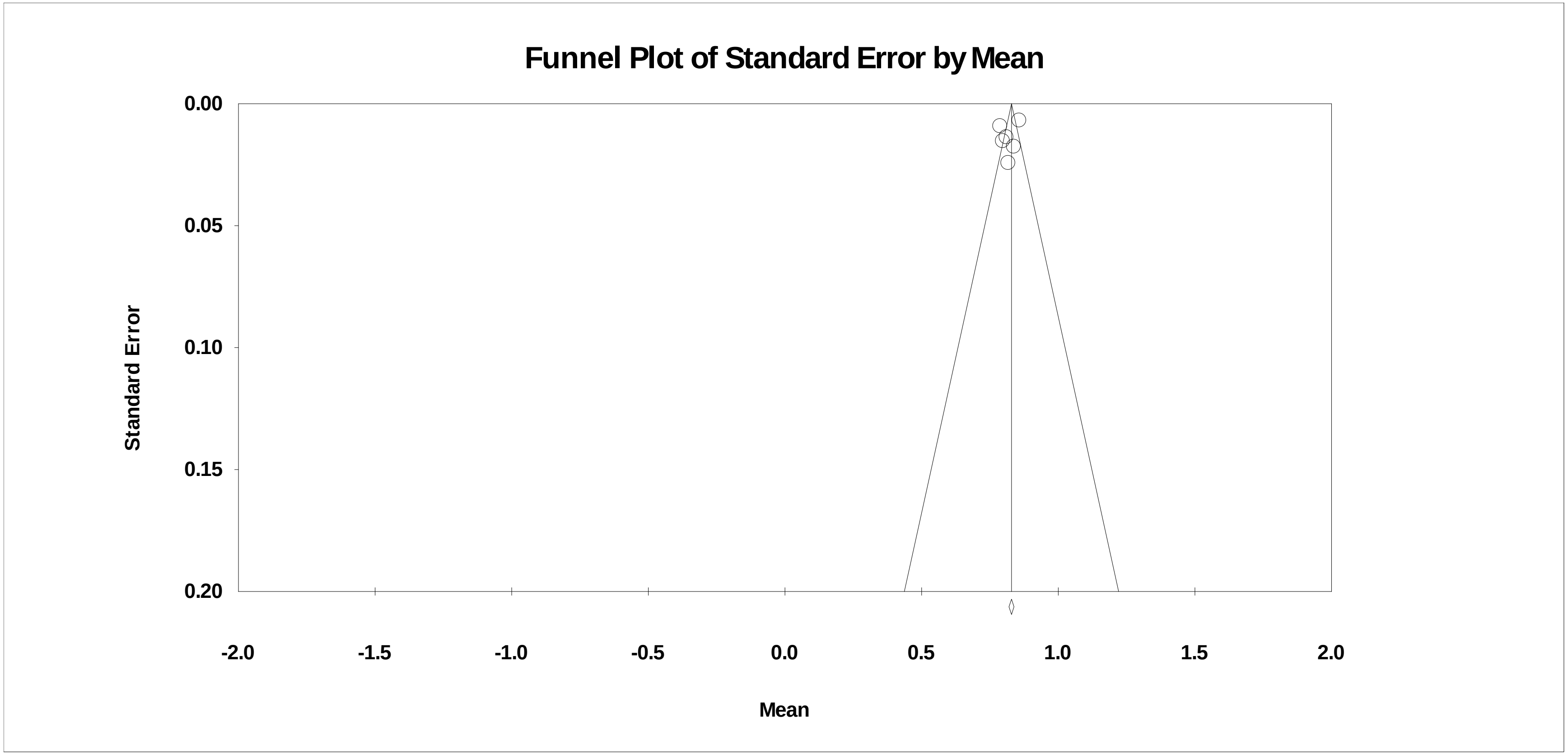

Supplemental figure 9c. WHR in office workers

Study name	Subgroup within study	Comparison	Outcome	Statistics for each study						
				Mean	Standard error	Variance	Lower limit	Upper limit	Z-Value	p-Value
Chae 2015	Blank	Office	WHR	0.860	0.007	0.000	0.846	0.874	119.921	0.000
Gao 2016	Blank	Office	WHR	0.840	0.018	0.000	0.805	0.875	46.910	0.000
Lagersted-Olsen 2014	Combined	Office	WHR`	0.813	0.014	0.000	0.786	0.841	57.996	0.000
				0.839	0.016	0.000	0.809	0.870	53.504	0.000

Mean and 95% CI

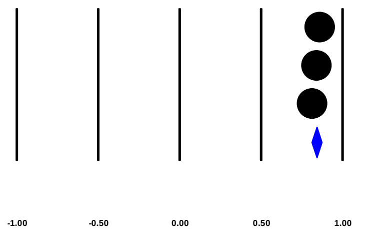

WHR

Supplemental figure 9d. WHR across all occupations except office workers

Study name	Subgroup within study	Comparison	Outcome	Statistics for each study						
				Mean	Standard error	Variance	Lower limit	Upper limit	Z-Value	p-Value
Cheung 2008	Women	School teachers	WHR	0.800	0.016	0.000	0.769	0.831	51.225	0.000
Cheung 2012	Blank	School teachers	WHR	0.790	0.009	0.000	0.771	0.809	83.208	0.000
Gao 2016	Blank	Office workers with sit-stand desks	WHR	0.800	0.032	0.001	0.738	0.862	25.298	0.000
Korshoj 2013	Cleaners	Laborers	WHR`	0.820	0.025	0.001	0.772	0.868	33.338	0.000
				0.796	0.007	0.000	0.781	0.810	106.290	0.000

Mean and 95% CI

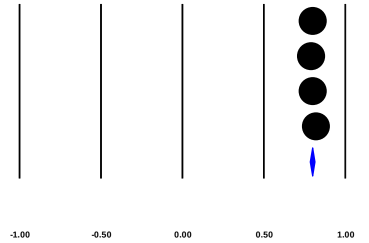

WHR

Supplemental figures 10a-d

Body fat percentage (BF%)

Supplemental figure 10a. BF% across all occupations

Study name	Subgroup within study	Comparison	Outcome	Mean	Standard error	Variance	Lower limit	Upper limit	Z-Value	p-Value
Carr 2016 a	Blank	Office	% BF	36.700	1.606	2.579	33.552	39.848	22.852	0.000
Carr 2016 b	Blank	Office workers with sit-stand desks	% BF	39.200	1.419	2.013	36.419	41.981	27.627	0.000
Cheung 2008	Combined	School teachers	% BF	24.710	0.475	0.225	23.780	25.640	52.070	0.000
Cheung 2012	Blank	School teachers	% BF	25.800	0.854	0.730	24.125	27.475	30.194	0.000
De Jong 2018	Blank	Office	% BF	35.600	1.698	2.882	32.273	38.927	20.970	0.000
Donath 2015	Combined	Office workers with sit-stand desks	% BF	28.252	1.383	1.913	25.540	30.963	20.424	0.000
Gorman 2013	Blank	Office	% BF	26.600	1.713	2.934	23.243	29.957	15.530	0.000
Koepp 2013	Blank	Office	% BF	31.400	1.317	1.734	28.819	33.981	23.848	0.000
Lagersted-Olsen 2014	Combined	Office	% BF	23.485	1.772	3.139	20.012	26.957	13.255	0.000
Mansi 2015	Combined	Laborers	% BF	28.492	1.391	1.936	25.765	31.219	20.479	0.000
Mansoubi 2016	Combined	Office	% BF	27.605	1.263	1.595	25.130	30.080	21.858	0.000
Schwartz 2016	Blank	Office	BF %	29.700	1.655	2.739	26.456	32.944	17.945	0.000
				29.741	1.383	1.914	27.029	32.452	21.498	0.000

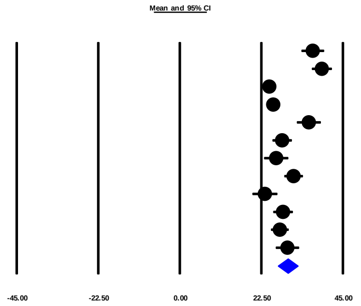

BF%

Supplemental figure 10b. Funnel plot for BF% across all occupations

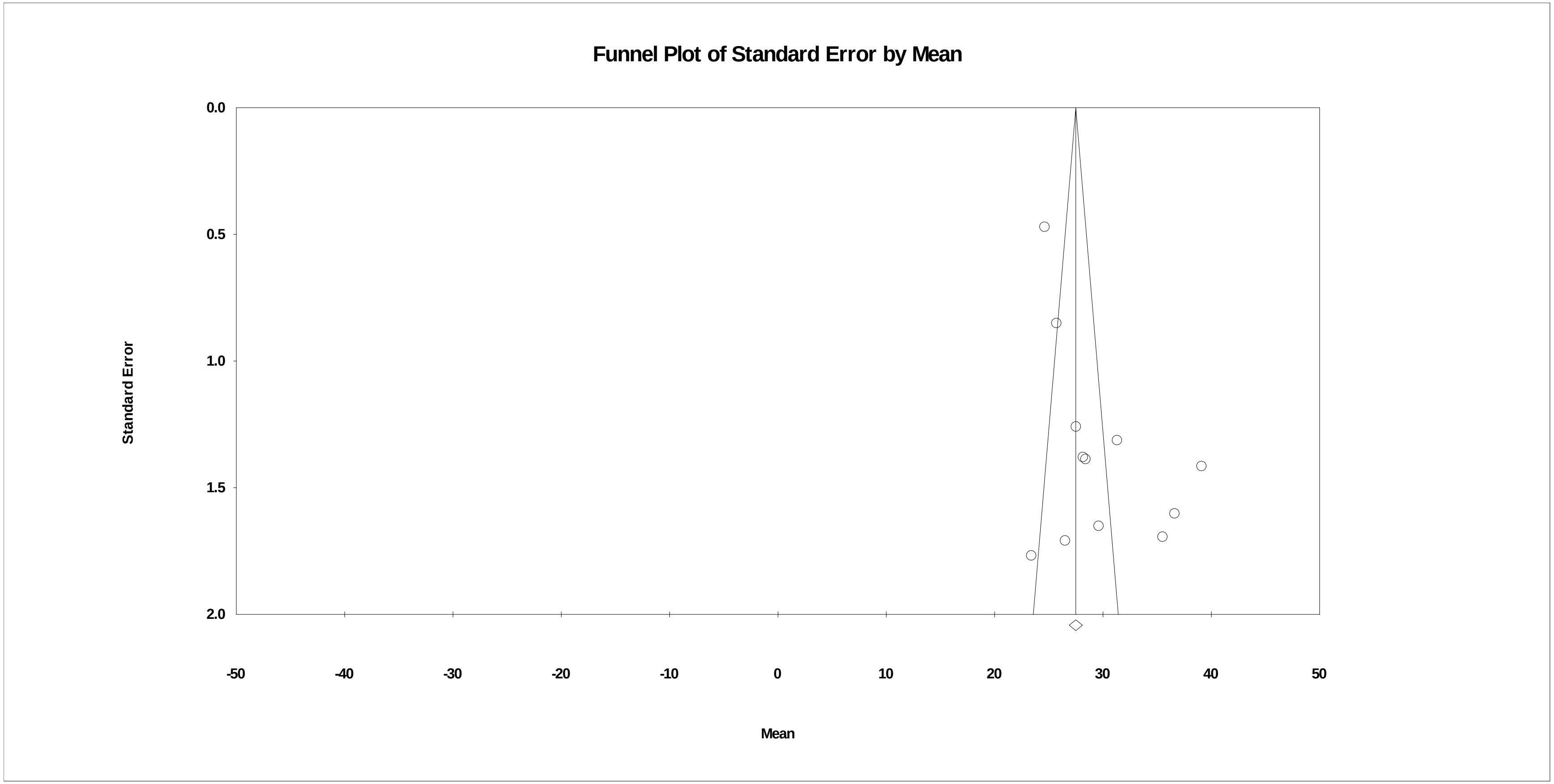

Supplemental figure 10c. BF% in office workers

Study name	Subgroup within study	Comparison	Outcome	Mean	Standard error	Variance	Lower limit	Upper limit	Z-Value	p-Value
				Statistics for each study						
Carr 2016 a	Blank	Office	% BF	36.700	1.606	2.579	33.552	39.848	22.852	0.000
De Jong 2018	Blank	Office	% BF	35.600	1.698	2.882	32.273	38.927	20.970	0.000
Gorman 2013	Blank	Office	% BF	26.600	1.713	2.934	23.243	29.957	15.530	0.000
Koepp 2013	Blank	Office	% BF	31.400	1.317	1.734	28.819	33.981	23.848	0.000
Lagersted-Olsen 2014	Combined	Office	% BF	23.485	1.772	3.139	20.012	26.957	13.255	0.000
Mansoubi 2016	Combined	Office	% BF	27.605	1.263	1.595	25.130	30.080	21.858	0.000
Schwartz 2016	Blank	Office	BF %	29.700	1.655	2.739	26.456	32.944	17.945	0.000
				30.168	1.699	2.887	26.838	33.498	17.755	0.000

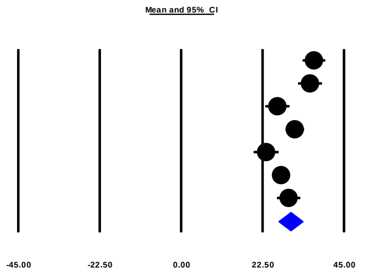

BF%

Supplemental figure 10d. BF% across all occupations except office workers

Study name	Subgroup within study	Comparison	Outcome	Statistics for each study						
				Mean	Standard error	Variance	Lower limit	Upper limit	Z-Value	p-Value
Carr 2016 b	Blank	Office workers with sit-stand desks	% BF	39.200	1.419	2.013	36.419	41.981	27.627	0.000
Cheung 2008	Combined	School teachers	% BF	24.710	0.475	0.225	23.780	25.640	52.070	0.000
Cheung 2012	Blank	School teachers	% BF	25.800	0.854	0.730	24.125	27.475	30.194	0.000
Donath 2015	Combined	Office workers with sit-stand desks	% BF	28.252	1.383	1.913	25.540	30.963	20.424	0.000
Mansi 2015	Combined	Laborers	% BF	28.492	1.391	1.936	25.765	31.219	20.479	0.000
				29.179	2.161	4.669	24.944	33.414	13.504	0.000

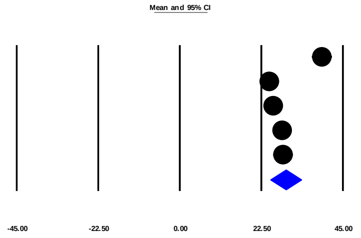

BF%

Supplemental figures 11a-b

Systolic blood pressure (SBP)

Supplemental figure 11a. SBP across all occupations

Study name	Subgroup within study	Comparison		Mean	Standard error	Variance	Lower limit	Upper limit	Z-Value	p-Value
Carr 2016 a	Blank	Office	SBP	124.000	1.136	1.289	121.774	126.226	109.198	0.000
Carr 2016 b	Blank	Office workers with sit-stand desks	SBP	120.000	2.155	4.645	115.776	124.224	55.678	0.000
Finkelstein 2015	Combined	Office	SBP	114.769	0.575	0.331	113.642	115.897	199.471	0.000
Gilson 2016	Total	Office	SBP	128.000	1.722	2.965	124.625	131.375	74.337	0.000
Gilson 2017	Truck drivers	Drivers	SBP	142.000	2.753	7.579	136.604	147.396	51.580	0.000
Koepp 2013	Blank	Office	SBP	132.000	2.167	4.694	127.753	136.247	60.923	0.000
Korshoj 2013	Cleaners	Laborers	SBP	115.000	2.460	6.050	110.179	119.821	46.754	0.000
Mansi 2015	Combined	Laborers	SBP	123.525	2.723	7.416	118.188	128.862	45.359	0.000
Reed 2018b	Combined	Healthcare	SBP - OM	115.026	1.473	2.171	112.138	117.914	78.066	0.000
Talbot 2011	Combined	Protective services	SBP	122.891	1.658	2.750	119.641	126.142	74.107	0.000
Torquati 2018	Nurses	Healthcare	SBP	115.000	2.188	4.787	110.712	119.288	52.560	0.000
Zhu 2018	Combined	Office	SBP	119.000	2.492	6.210	114.116	123.884	47.755	0.000
				122.474	2.088	4.360	118.381	126.567	58.653	0.000

Statistics for each study

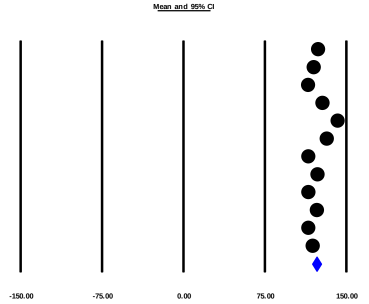

SBP

Supplemental figure 11b. Funnel plot for SBP across all occupations

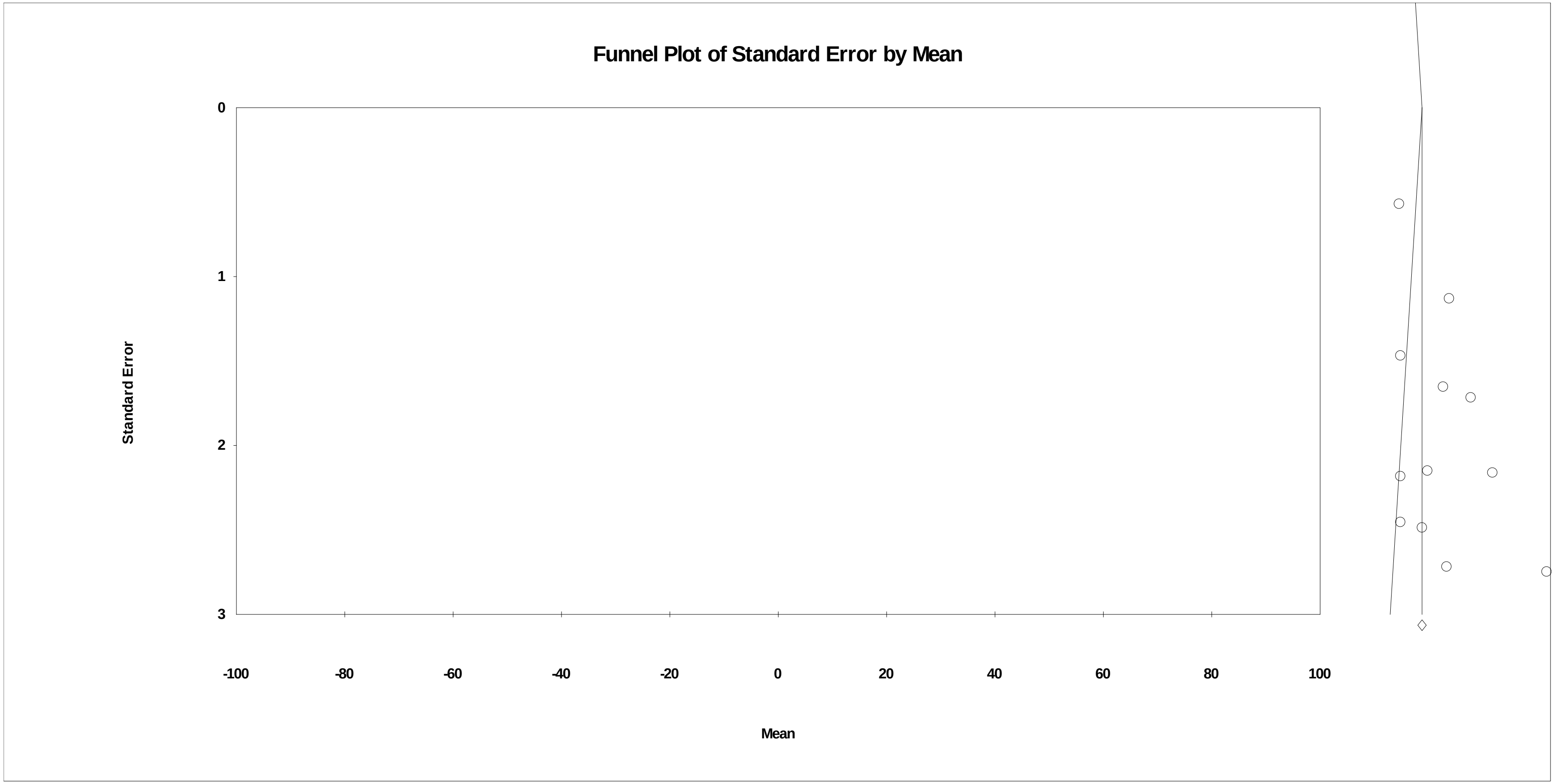

Supplemental figures 12a-b

Diastolic blood pressure (DBP)

Supplemental figure 12a. DBP across all occupations

Study name	Subgroup within study	Comparison	Outcome	Mean	Standard error	Variance	Lower limit	Upper limit	Z-Value	p-Value
Abd 2012	Physicians	Healthcare	DBP - SR	88.900	1.153	1.329	86.641	91.159	77.117	0.000
Carr 2016 a	Blank	Office	DBP	77.000	1.298	1.684	74.456	79.544	59.332	0.000
Carr 2016 b	Blank	Office workers with sit-stand desks	DBP	73.000	1.796	3.226	69.480	76.520	40.645	0.000
Finkelstein 2015	Combined	Office	DBP	71.471	0.371	0.137	70.745	72.197	192.881	0.000
Gilson 2016	Total	Office	DBP	86.000	1.192	1.421	83.664	88.336	72.143	0.000
Gilson 2017	Truck drivers	Drivers	DBP	87.000	2.524	6.368	82.054	91.946	34.475	0.000
Koepp 2013	Blank	Office	DBP	89.000	3.000	9.000	83.120	94.880	29.667	0.000
Korshoj 2013	Cleaners	Laborers	DBP	77.000	1.789	3.200	73.494	80.506	43.044	0.000
Mansi 2015	Combined	Laborers	DBP	75.542	1.625	2.642	72.356	78.727	46.478	0.000
Reed 2018b	Combined	Healthcare	DBP - OM	75.184	0.916	0.839	73.389	76.980	82.072	0.000
Talbot 2011	Combined	Protective services	DBP	76.070	1.148	1.318	73.820	78.320	66.262	0.000
Torquati 2018	Nurses	Healthcare	DBP	78.000	1.459	2.128	75.141	80.859	53.474	0.000
Zhu 2018	Combined	Office	DBP	76.133	1.724	2.971	72.755	79.512	44.169	0.000
				79.094	1.814	3.291	75.539	82.650	43.602	0.000

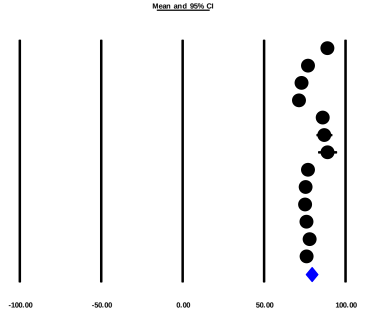

DBP

Supplemental figure 12b. Funnel plot for DBP across all occupations

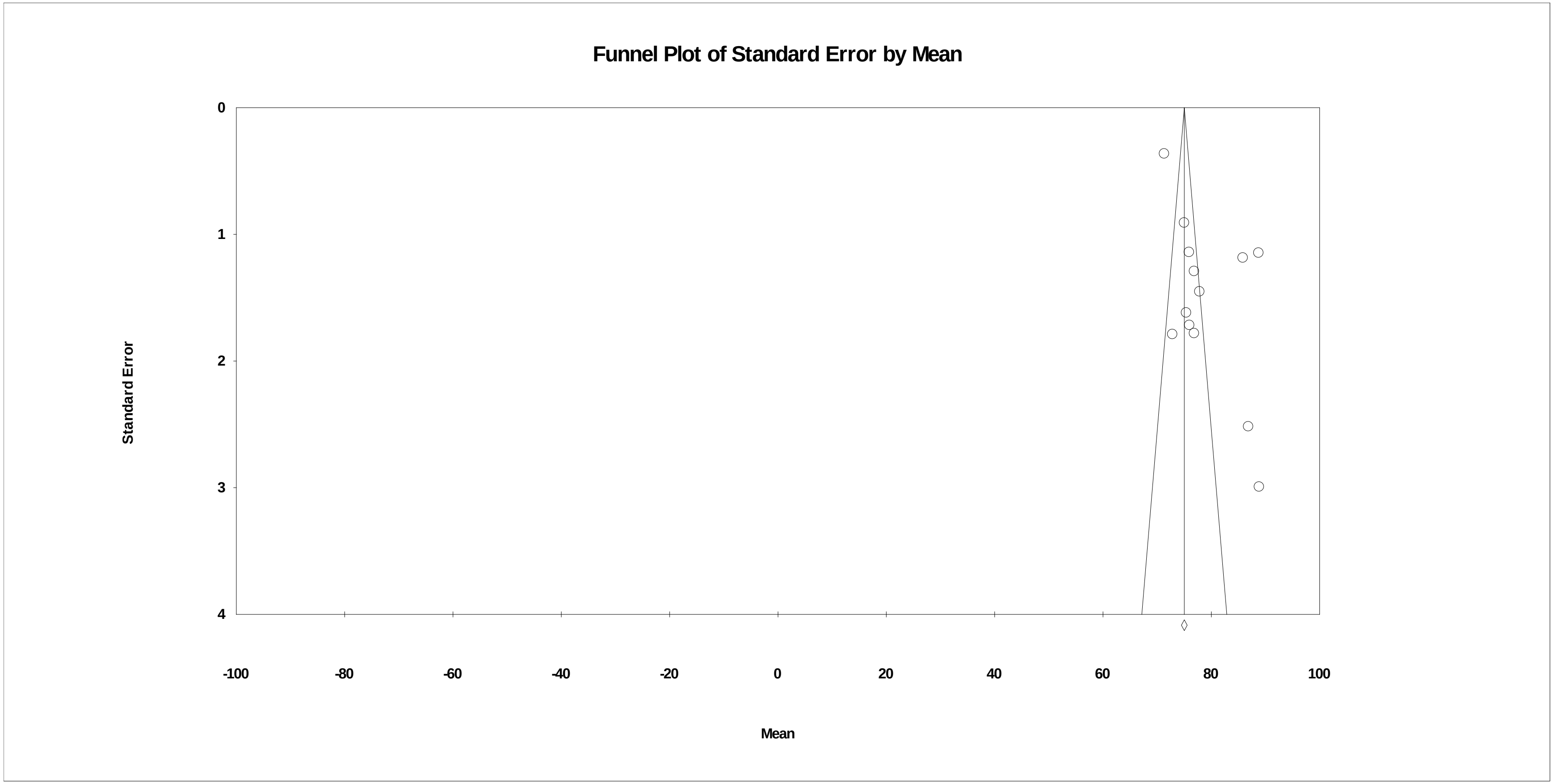

Supplemental figures 13a-b

Total cholesterol

Supplemental figure 13a. Total cholesterol across all occupations

Study name	Subgroup within study	Comparison	Outcome	Statistics for each study						
				Mean	Standard error	Variance	Lower limit	Upper limit	Z-Value	p-Value
Alkajah 2012	Combined	Office	TC	4.527	0.184	0.034	4.166	4.887	24.615	0.000
Koepp 2013	Blank	Office	TC	5.000	0.133	0.018	4.739	5.261	37.500	0.000
Talbot 2011	Combined	Protective services	TC	4.751	0.093	0.009	4.568	4.934	50.944	0.000
Zhu 2018	Combined	Office	TC	4.720	0.131	0.017	4.463	4.977	35.984	0.000
				4.769	0.083	0.007	4.606	4.932	57.458	0.000

Mean and 95% CI

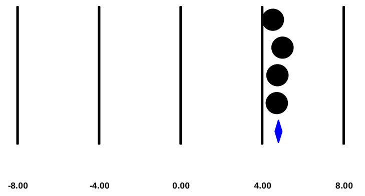

Total cholesterol

Supplemental figure 13b. Funnel plot for total cholesterol across all occupations

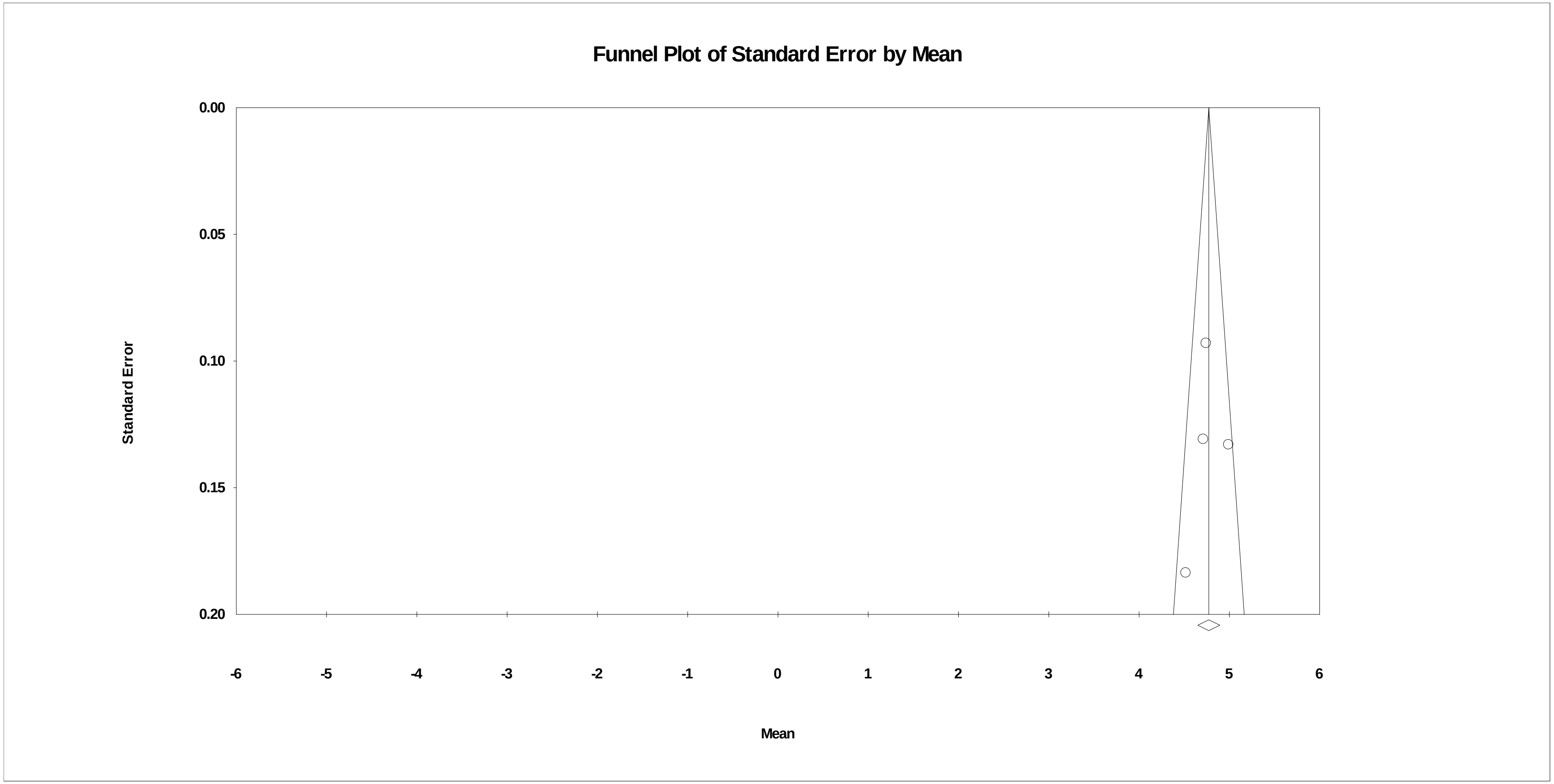

Supplemental figures 14a-b

Triglycerides

Supplemental figure 14a. Triglycerides across all occupations

Study name	Subgroup within study	Comparison	Outcome	Mean	Standard error	Variance	Lower limit	Upper limit	Z-Value	p-Value
Alkajah 2012	Combined	Office	TG	0.917	0.165	0.027	0.594	1.239	5.567	0.000
Andersen 2011	Taxi drivers	Drivers	TG	2.400	0.208	0.043	1.992	2.808	11.526	0.000
Gorman 2013	Blank	Office	TG	0.800	0.077	0.006	0.649	0.951	10.392	0.000
Koepp 2013	Blank	Office	TG	1.500	0.167	0.028	1.173	1.827	9.000	0.000
Zhu 2018	Combined	Office	TG	1.100	0.095	0.009	0.915	1.285	11.634	0.000
				1.317	0.219	0.048	0.887	1.747	6.002	0.000

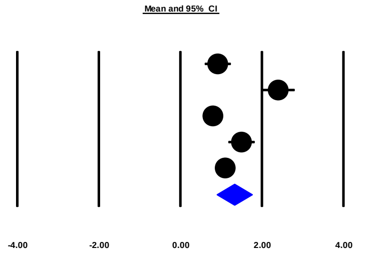

Triglycerides

Supplemental figure 14b. Funnel plot for triglycerides across all occupations

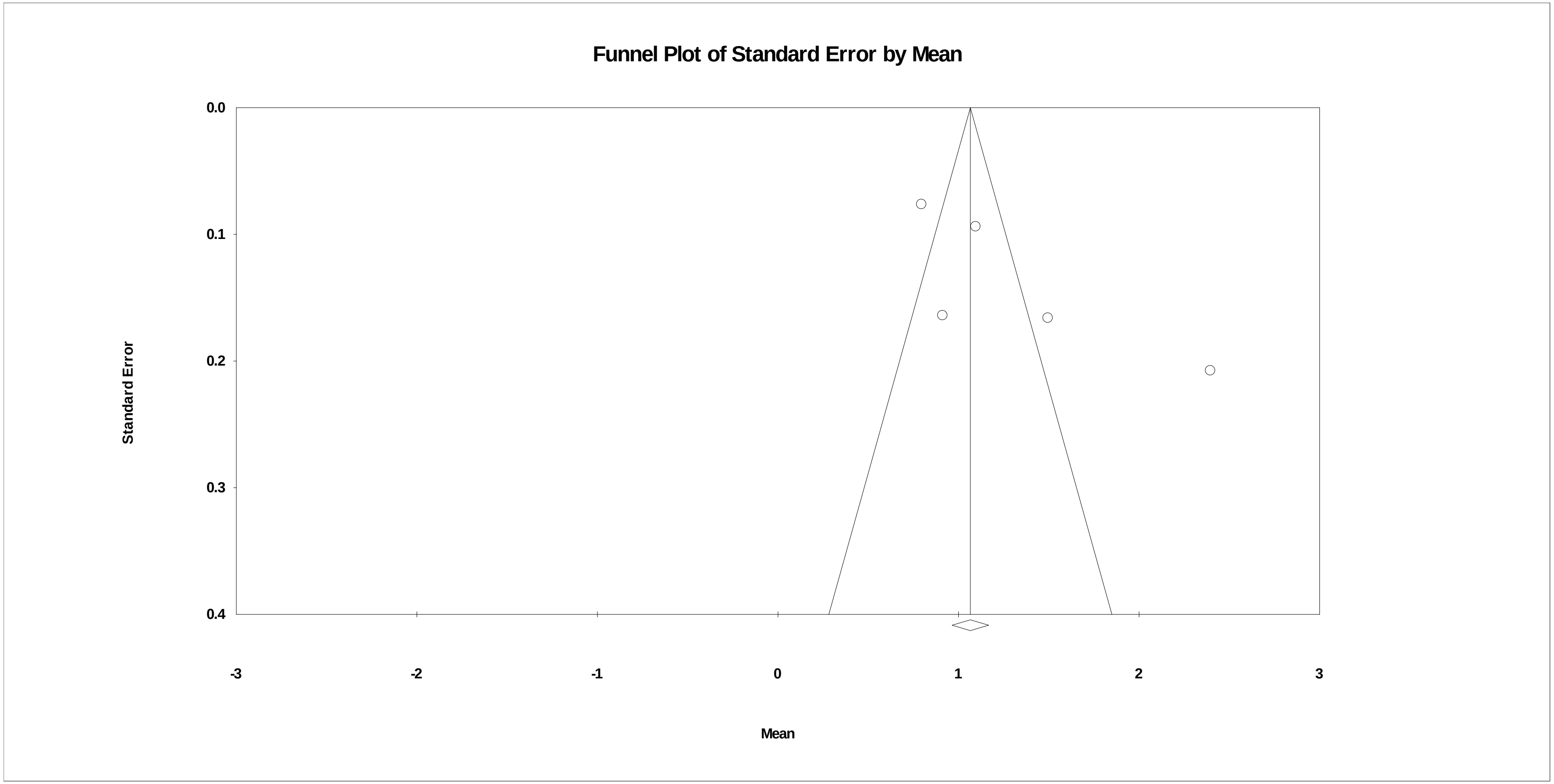

Supplemental figures 15a-b

High density lipoprotein (HDL)

Supplemental figure 15a. HDL across all occupations

Study name	Subgroup within study	Comparison	Outcome	Mean	Standard error	Variance	Lower limit	Upper limit	Z-Value	p-Value
Alkajah 2012	Combined	Office	HDL - C	1.460	0.080	0.006	1.302	1.618	18.168	0.000
Andersen 2011	Taxi drivers	Drivers	HDL - C	1.000	0.018	0.000	0.965	1.035	55.227	0.000
Gorman 2013	Blank	Office	HDL - C	1.600	0.077	0.006	1.449	1.751	20.785	0.000
Koepp 2013	Blank	Office	HDL - C	1.400	0.083	0.007	1.237	1.563	16.800	0.000
Talbot 2011	Combined	Protective services	HDL - C	1.302	0.038	0.001	1.228	1.376	34.422	0.000
				1.346	0.121	0.015	1.108	1.584	11.104	0.000

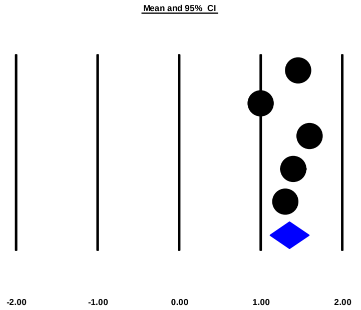

HDL (mmol/L)

Supplemental figure 15b. Funnel plot for HDL across all occupations

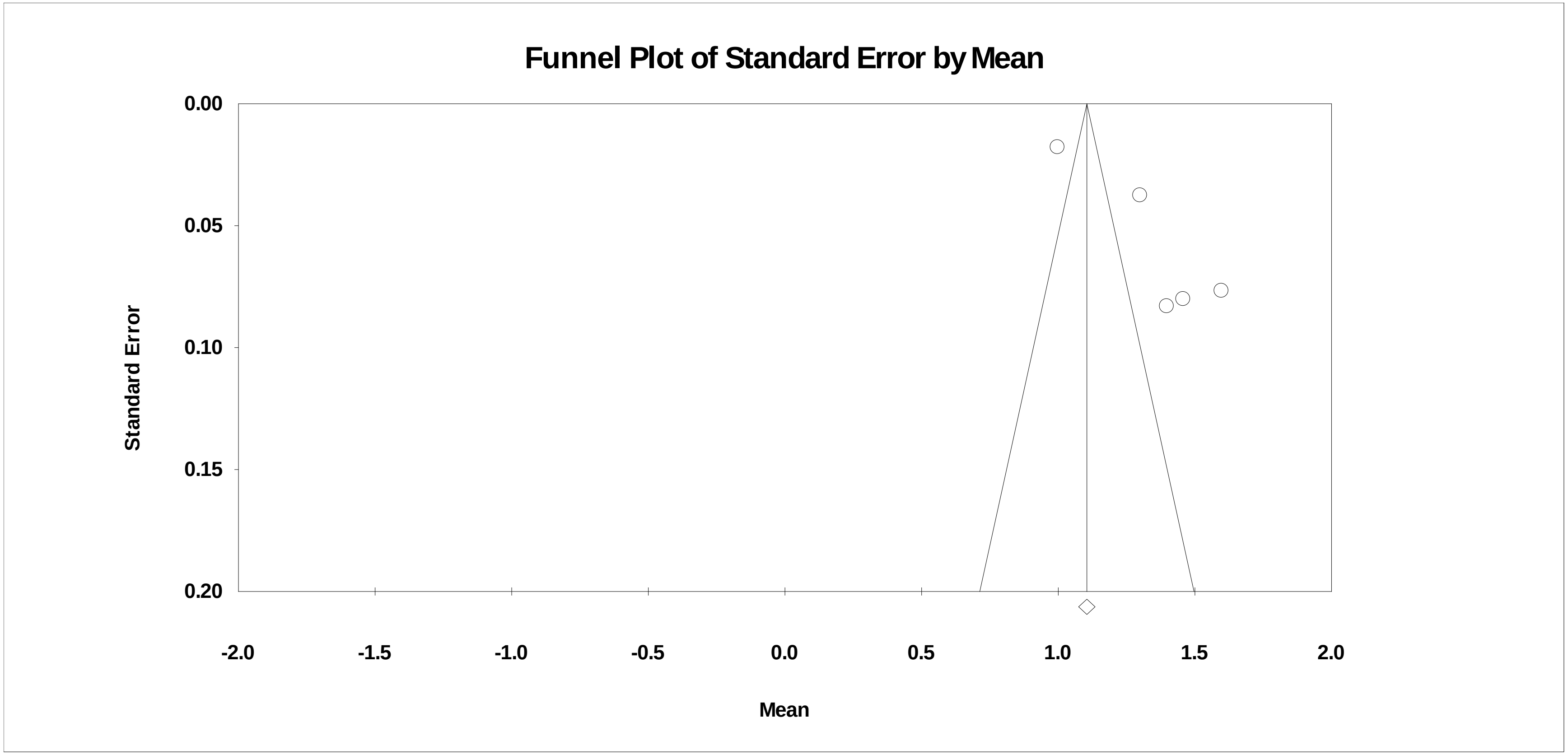

Supplemental figures 16a-b

Low density lipoprotein (LDL)

Supplemental figure 16a. LDL across all occupations

Study name	Subgroup within study	Comparison	Outcome	Mean	Standard error	Variance	Lower limit	Upper limit	Z-Value	p-Value
Andersen 2011	Taxi drivers	Drivers	LDL - C	3.500	0.063	0.004	3.376	3.624	55.227	0.000
Koepp 2013	Blank	Office	LDL - C	2.900	0.133	0.018	2.639	3.161	21.750	0.000
Talbot 2011	Combined	Protective services	LDL - C	2.849	0.093	0.009	2.666	3.032	30.548	0.000
Zhu 2018	Combined	Office	LDL - C	2.927	0.121	0.015	2.690	3.163	24.241	0.000
				3.051	0.193	0.037	2.674	3.428	15.844	0.000

Mean and 95% CI

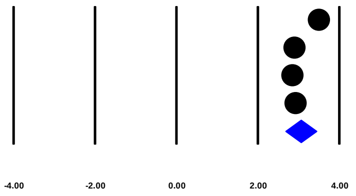

LDL - C

Supplemental figure 16b. Funnel plot for LDL across all occupations

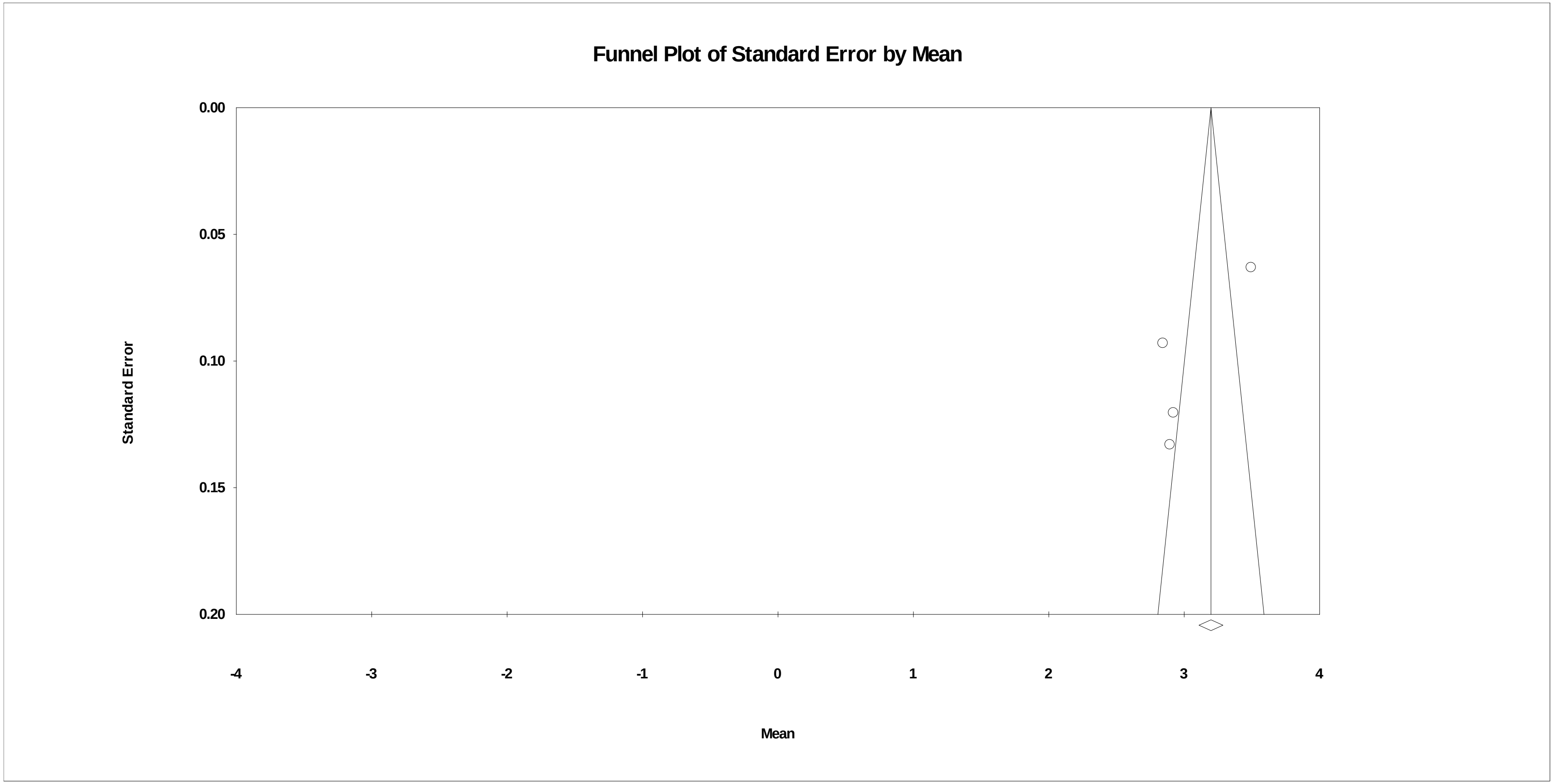

Supplemental figures 17a-b

Blood glucose

Supplemental figure 17a. Blood glucose across all occupations

Study name	Subgroup within study	Comparison	Outcome	Mean	Standard error	Variance	Lower limit	Upper limit	Z-Value	p-Value
Alkajah 2012	Combined	Office	Glucose	4.870	0.074	0.005	4.725	5.015	65.928	0.000
Andersen 2011	Taxi drivers	Drivers	Glucose	5.500	0.109	0.012	5.287	5.713	50.625	0.000
De Jong 2018	Blank	Office	Glucose	5.000	0.094	0.009	4.816	5.184	53.157	0.000
Gorman 2013	Blank	Office	Glucose	4.800	0.058	0.003	4.687	4.913	83.138	0.000
Koepp 2013	Blank	Office	Glucose	5.211	0.213	0.045	4.794	5.628	24.469	0.000
Zhu 2018	Combined	Office	Glucose	5.367	0.125	0.016	5.122	5.612	42.913	0.000
				5.108	0.118	0.014	4.876	5.340	43.178	0.000

(Statistics for each study)

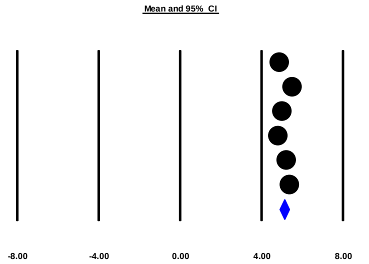

Glucose

Supplemental figure 17b. Funnel plot for blood glucose across all occupations

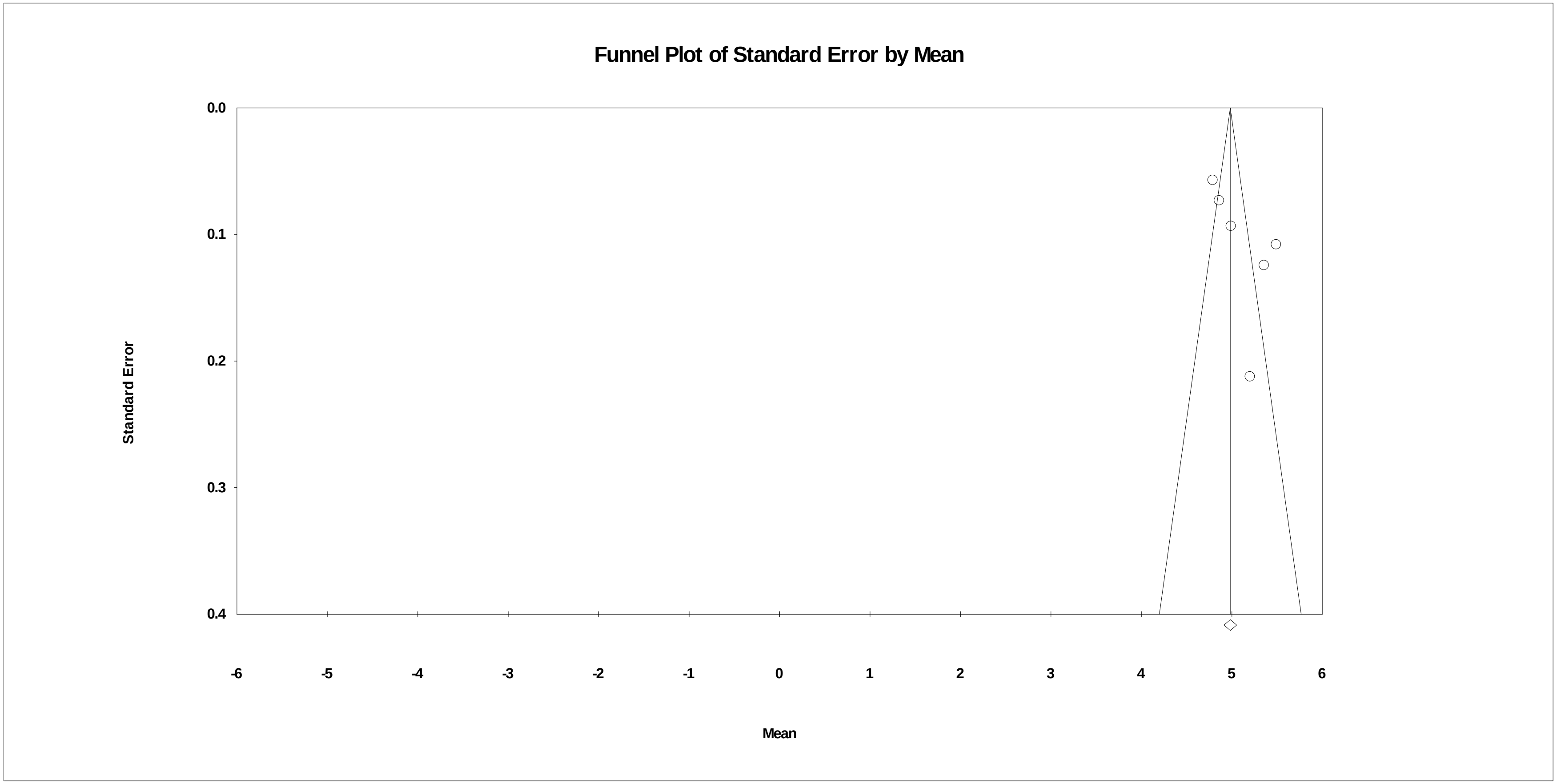

Supplemental figures 18a-b

Peak VO_2

Supplemental figure 18a. Peak VO_2 across all occupations

Study name	Subgroup within study	Comparison	Outcome	Statistics for each study						
				Mean	Standard error	Variance	Lower limit	Upper limit	Z-Value	p-Value
Andersen 2011	Taxi drivers	Drivers	Peak VO2	32.200	0.344	0.118	31.526	32.874	93.595	0.000
Carr 2016 a	Blank	Office	Peak VO2	30.700	1.314	1.727	28.125	33.275	23.364	0.000
Carr 2016 b	Blank	Office workers with sit-stand desks	Peak VO2	30.800	1.473	2.169	27.913	33.687	20.913	0.000
Finkelstein 2015	Combined	Office	Peak VO2	35.343	0.165	0.027	35.019	35.666	214.079	0.000
Korshoj 2013	Cleaners	Laborers	Peak VO2	34.000	2.012	4.050	30.056	37.944	16.895	0.000
				32.710	1.128	1.273	30.499	34.922	28.992	0.000

Mean and 95% CI

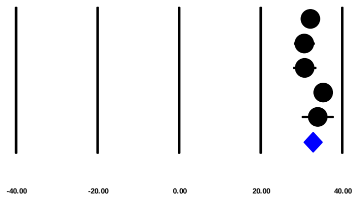

Peak VO2

Supplemental figure 18b. Funnel plot for peak VO_2 across all occupations

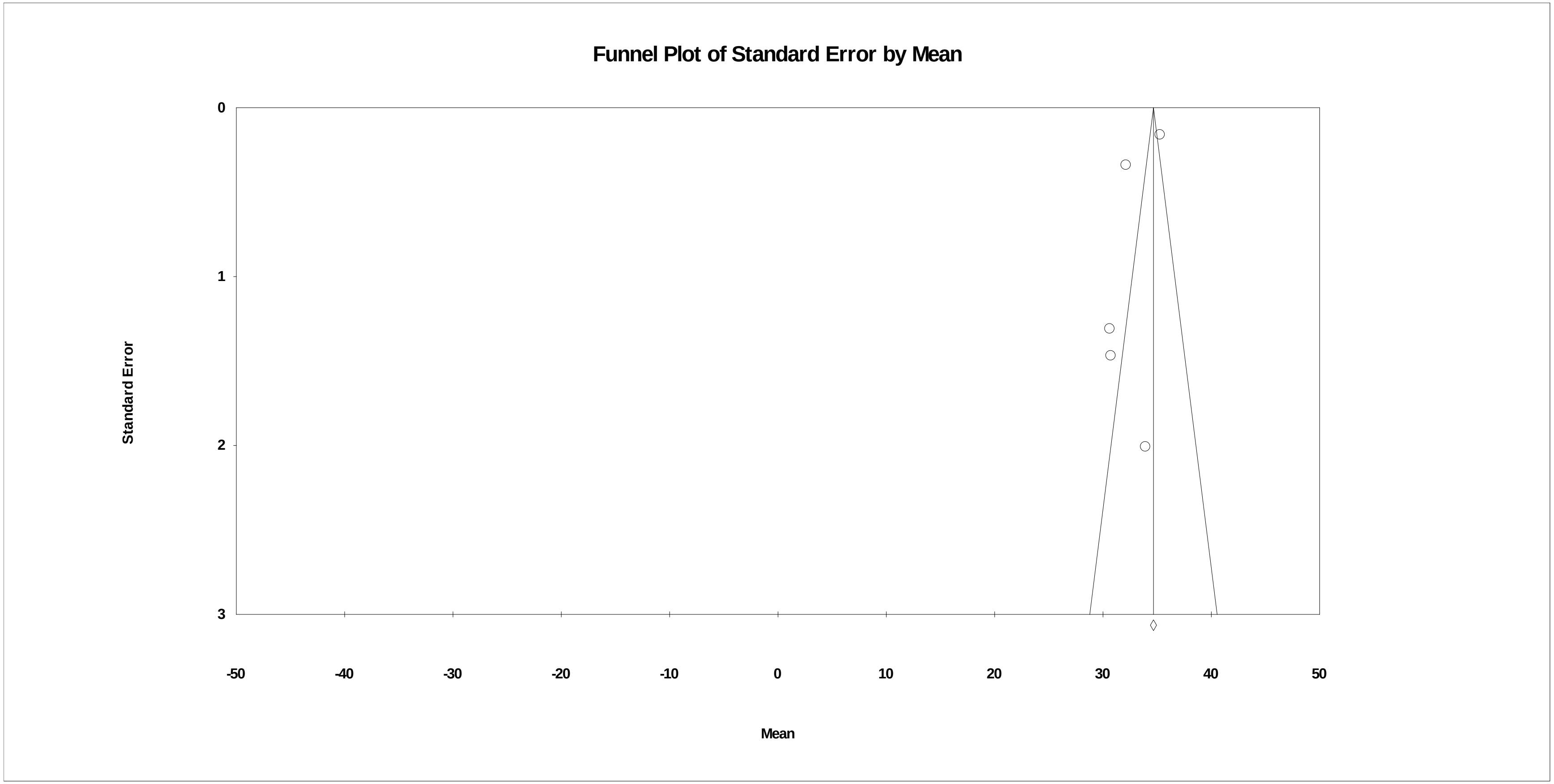

Supplement: Supplementary file 4 — Figures S1–S18. Individual meta-analyses and funnel plots for behaviour, cardiometabolic and fitness outcomes. (PDF 658 kb) [file 12966_2019_790_MOESM4_ESM.pdf]
