# Supplementary material for: Device-measured physical activity, sedentary behaviour and cardiometabolic health and fitness across occupational groups: a systematic review and meta-analysis
Source: Int J Behav Nutr Phys Act. 2019 Apr 2;16:30. doi: 10.1186/s12966-019-0790-9 (PMC6444868; doi:10.1186/s12966-019-0790-9)
Supplement: Supplementary file 5 — Table S4. Cardiometabolic and fitness outcomes by occupation group. (DOCX 54 kb) [file 12966_2019_790_MOESM5_ESM.docx]

Table S4. Cardiometabolic and fitness outcomes by occupation group

| **First Author, year** | **Occupation group (s)** | **Sample size analyzed (N or %)** | | | | **SR or OM** | **Body mass (kg)** | **BMI (kg/m^2^)** | **WC (cm)** | **Body Fat (%)** | **BP (mmHG)** | **Other** |
| --- | --- | --- | --- | --- | --- | --- | --- | --- | --- | --- | --- | --- |
|  |  | **Women** | | **Men** | **Total** |  |  |  |  |  |  |  |
| **Academics** | | | | | | | | | | | | |
| Ryan, 2011 | Lecturers, researchers | |  |  | Lecturers 27  Researchers 27 | NR | Lecturers: 70 (12) Researchers 71 (11) | Lecturers 24.5 (3.5) Researchers  24.1 (3.1) | - | - | - | - |
| **Call centre workers** | | | | | | | | | | | | |
| Chau, 2016 | Call center workers | | 14 (45%) | 17 (55%) | 31 | SR | - | 26.8 (5.5) | - | - | - | - |
| Chitkara, 2014 | Call center workers | | 15 (79%) | 4 (21%) | 19 | OM | - | 29.9 (6.5) | - | - | - | - |
| Clark, 2011 | Call center workers | | 10 (42%) | NR | 24 | OM | - | 27.8 (22.9-35.8)** | - | - | - | - |
| Straker, 2013 | Call center workers | | 68 |  | 131 | NR | - | 24.5 (4.0) | - | - | - | - |
| **Drivers** | | | | | | | | | | | | |
| Andersen, 2011 | Taxi drivers | 0 (0%) | | 71 (100%) | 71 | OM | - | 27.8 (3.3) | 100 (9) | - | - | **HDL (mmol/L):** 1 (0.2)  **LDL (mmol/L)**: 3.5 (0.7)  **TG (mmol/L):** 2.4 (2.3)  **Glucose**: **(mmol/L)** 5.5 (1.2)  **Glucose2h (mmol/L)**: 7.7 (3.3)  **HOMA-IR:** 5.1 (2.7)  **VO (ml/kg/min)_2_:** 32.2 (3.8) |
| French, 2007 | Transit workers | 22% | | 78% | 158 | OM | 97.6 (0.7) | 32.2 (7.3) | - | - | - | - |
| Gilson, 2017 | Truck drivers | 0 (0%) | | 19 (100%) | 19 | OM | - | 31.2 (4.6) | 109 (10) | - | **SBP**: 142 (12)  **DBP:** 87 (11) | - |
| Varela-Mato, 2016a | Bus drivers | NR | | NR | 28 | OM | - | 28.1 (5.8)  ** | 101.5 (21)  ** | 26 (9)** | SBP: 137 (14)**  DBP: 88 (11)** | **RHR:** 72 (12)** |
| Varela-Mato, 2016b | Truck drivers | 0 (0%) | | 77 (100%) | 77 | OM | - | 28 (5) | - | - | - | - |
| Varela-Mato, 2017a | Bus drivers | 0 (0%) | | 28 (100%) | 28 | OM | - | 28.5 (3.9) | - | - | - | - |
| Varela-Mato, 2017b | Truck drivers | 0 (0%) | | 87 (100%) | 87 | OM | - | 27.7 (19.6, 43.4) ** | 100.9 (77.5, 141.0)** | 24.8 (12.2; 43.3)** | **SBP:** 129.0 (108.5, 155.0)**  **DBP:** 81.0 (65.0, 104.0)** | **WHR:** 0.93 (0.8-1.1)**  **RHR:** 61.0 (42.0, 89.0)**  **TC (mmol/L):** 5.1 (2.6. 7.3)**  **HDL (mmol/L):** 1.4 (0.9, 1.7)**  **LDL (mmol/L):** 3.2 (1.0, 5.4)**  **TG (mmol/L):** 1.5 (0.7, 4.3)**  **Fasting glucose (mmol/L):** 5.1 (3.7, 12.7)** |
| Wong, 2014 | Transportation drivers | 0 (0%) | | 23 (100%) | 23 | NR | - | 33.7 (7.9) | - | - | - | - |
| **Factory** | | | | | | | | | | | | |
| Bassey, 1983 | Factory workers | | NR | NR | 59 | NR | Men: 72.5 (1.4)  Women: 63.8 (1.6)  [mean (SE)] | - | - | - | - | - |
| **Healthcare** | | | | | | | | | | | | |
| Abd, 2012 | Physicians (general cardiologists, cardiothoracic surgeons, procedural cardiologists, cardiac anesthes-iologists) | 2 (7%) | | 26 (93%) | 28 | SR | - | 25.1 (2.2) | 89.4 (7.1) | - | **DBP:** 88.9 (6.1) | **RHR:** 65.1 (9.1) |
| Arias, 2017 | Patient care workers | 41 (87%) | | 6 (13%) | 47 | NR | - | 26 (4) | - | - | - | - |
| Brewer, 2016 | Physical therapists | Outpatient: 11 (18%); inpatient: 27 (44%) | | Out-patient20 (33%); inpatient: 3 (5%) | 61 (outpatient: 31; inpatient 30) | NR | Outpatient: 77.9 (14.4)  Inpatient: 67.3 (14.1) | Outpatient: 26.2 (3.7)  Inpatient: 25.1 (5.7) | - | - | - | - |
| Croteau, 2017 | Nurses and nursing support staff | 19 (95%) | | 1 (5%) | 20 | NR | - | 27.4 (4.1) | 89.7 (12.7) | - | - | - |
| He, C. 2013 | Nursing aids | 22 (100%) | | 0 (0%) | 22 | OM | 8h day: 76.1 (17.2); 8h evening: 79.2 (18.9); 8hr night: 88.1 (20.3); 12h day: 70.2 (11.1); 12h night: 86.2 (17.9) | - | 8h day: 96.3 (14.5); 8h evening: 96.0 (16.8); 8h night: 110.0 (20.1); 12h day: 87.6 (12.7); 12h night: 104.6 (20.3) | - | - | - |
| Jirathananuwat, 2017 | Nurse practitioners (NP) and nurse managers (NM) | 283 (97.9%) (NP: 136 (95.7%); NM: 147 (100%) | | 6 (2%) (NP: 6 (2%); NM: 0 (0%)) | 289 | OM | - | All: 22.5 (4.1); NP: 21.2 (3.4); NP 23.7 (4.4) | - | - | - | - |
| Lunde, 2017* | Healthcare workers | 73% | | 22% | 63 | SR | - | 25.1 (3.8) | - | - | - | - |
| Martinez, 2013 | Obstetricians | 11 (61%) | | 7 (39%) | 18 | NR | - | 22.6** (18.5-27.9 [min-max] | - | - | - | - |
| Neil-Sztramko, 2017 | Physiotherapist | 36 (95%) | | 2 (5%) | 38 | SR | - | All participant: 22.3 (2.9) Public practice PTs: 22.9 (3.0)  Private practice PTs: 21.1 (2.1) | - | - | - | - |
| Reed, 2018a | Nurses | 23 (6%) | | 387 (94%) | 410 | OM | All: 73.3 (15.0); Women: 72.7 (15.0); Men: 84.0 (10.2) | All: 27.1 (5.4); Women: 27.1 (5.5); Men: 27.7 (3.6) | All: 80.8 (11.8); Women: 80.3 (11.8); Men: 89.2 (7.7) | - | **SBP:**  All: 114 (12); Women: 114 (12); Men: 119 (14)  **DBP:**  All: 74 (9); Women: 74 (9); Men: 78 (12) | **RHR (bpm):**  All: 69 (10)  Women: 69 (10)  Men: 64 (9) |
| Reed, 2018b | Nurses | 74 | | 2 | 76 | OM | Men: 73.0 (25.5)  Women: 75.4 (16.4) | Men: 22.2 (3.1)  Women: 27.7 (5.6) | Men: 83.7 (17.5)  Women: 84.2 (12.6) | - | **SBP:**  Men: 116 (6)  Women: 115 (13)  **DBP:**  Men: 82 (4)  Women: 75 (8) | **RHR (bpm):**  Men: 69 (9)  Women: 68 (9) |
| Torquati, 2018 | Nurses | 41 (87%) | | 6 (13%) | 47 | OM | 76 (17) | 28 (6) | 87 (13) | - | **SBP:** 115 (15)  **DBP:** 78 (10) | **-** |
| **Laborers** | | | | | | | | | | | | |
| Arias, 2015 | Construction workers | 0 (%) | | 55 (100%) | 55 | NR | - | 29 (4) | - | - | - | - |
| Dollman, 2016* | Farmers | 0 (0%) | | 29 (100%) | 29 | OM | - | 29.9 (4.4) | - | - | - | - |
| Julin, 2011 | Construction workers | 2 (8%) | | 25 (92%) | 27 | NR | - | 25.6 (NR) | - | - | - | - |
| Korshoj, 2013 | Cleaners | 20 (100%) | | 0 (0%) | 20 | OM | 70.9 (15.3) | 25.8 (5) | 85.5 (14.9) | - | **SBP:** 115 (11)  **DBP:** 77 (9) | **WHR:** 0.8 (0.1)  **VO_2_ (ml/kg/min):** 34 (9) |
| Lunde, 2017* | Construction workers | 2% | | 98% | 51 | SR |  | 25.7 (3.3) | - | - | - | - |
| Mansi, 2015 | Meat processing workers | I: 19 (65.5%); C: 15 (51.7%) | | I: 10 (34.5%)C: 14 (48.3%) | 58 (I: 29; C: 29) | OM | I: 80.2 (16.9)  C: 76.9 (13.9) | I: 29.9 (16.9)  C: 28.3 (13.9) | I: 98.9 (12.7)  C: 93.5 (12.1) | I: 29.6 (6.9)  C: 27.7 (6.9) | **SBP:**  I: 125.1 (16.3)  C: 122.4 (11.3)  **DBP:**  I: 76.3 (9.6)  C: 75.0 (6.9) | - |
| Pontt, 2015* | Farmers | 0 (0%) | | 29 (100%) | 29 | NR | - | 29.9 (NR) | - | - | - | - |
| **Office workers (traditional desks)** | | | | | | | | | | | | |
| Alkhajah, 2012 | Office workers | 29 (91%) | | 3 (9%) | 32 | OM | I: 64.8 (10)  C: 58.3 (8.3) | I: 22.6 (2.6) C: 21.5 (2.6) | I: 83.3 (9)  C: 79.2 (8.4) | - | - | **TC (mmol/L):**  I: 4.5 (1); C: 4.5 (0.7)  **HDL (mmol/L)**:  I: 1.4 (0.4); C: 1.6 (0.4)  **Glucose (mmol/L):**  I: 4.8 (0.3); C: 4.9 (0.4)  **TG (mmol/L)**:  I: 1.1 (0.9), C = 0.7 (0.4) |
| Bird, 2015 | University staff | NR | | NR | 10 | SR | - | 26.5 (NR) | - | - | - | - |
| Brakenridge 2016 | Office workers | 70 (46%) | | 83 (54%) | 153 | OM | - | 24.6 (3.4) | - | - | - | - |
| Brown, 2013 | Office workers | 76 (70%) | | 32 (30%) | 108 | NR | - | Total: 26.5 (4.4)  Women: 25.8 (4.1)  Men: 28.4 (4.5) | Total: 84.2 (12.4) Women: 79.5 (21)  Men: 95.4 (9.1) | - | - | - |
| Carr, 2016 | Office workers | 74% | | 26% | 38 | OM | 87.3 (21.4) | 30.1 (6.8) | 97.8 (14.9) | 36.7 (9.9) | **SBP:** 124 (7)  **DBP:** 77 (8) | **HR (bpm):** 74 (10)  **VO_2_ (ml/kg/min):** 30.7 (8.1) |
| Chae, 2015 | Office workers | 35 (50%) | | 35 (50%) | 70 | OM | - | 23.4 (3.4) | - | - | - | **WHR:** 0.9 (0.1) |
| Chastin, 2009* | Postal workers (office) | 6 (15%) | | 33 (85%) | 39 | SR | 82 (12) | 27 (3) | - | - | - | - |
| Chau, 2014 | Office workers | 36 (86%) | | 6 (14%) | 38 | SR | 64 (13) | - | - | - | - | - |
| Clark, 2011 | Office workers | 54 (66%) | | NR | 82 | OM | - | 25.5 (23.7-28.9)** | - | - | - | - |
| Clemes, 2014a | Office workers | 60% | | 40% | 72 | OM | - | 24 (3.5) | - | - | - | - |
| Clemes, 2014b | Office workers | 70% | | 30% | 170 | OM | - | 24.5 (3.8) | - | - | - | - |
| De Jong, 2018 | Office workers | 12 | | 10 | 22 | OM | - | 30.4 (0.5) | - | 35.6 (7.4) | - | **Glucose (mmol/L):** 5.00 (0.41) |
| Dollman, 2016* | Office workers | 0 (0%) | | 28 (100%) | 28 | OM | - | 28.4 (3.2) | - | - | - | - |
| Evans, 2012 | Office workers | 22 (79%) | | 6 (21%) | 28 | NR | - | I: 23.6 (2.8); C: 23.7 (3.5) | - | - | - | - |
| Finkelstein, 2015 | Office workers | Cash: 113 (57.4%) Charity: 105 (52.8%) Control: 112 (55.7%) Fitbit Only: 100 (49.3%) | | Cash: 84 (42.6%)Charity: 94 (47.2%) Control: 89 (44.3%)Fitbit Only: 103 (50.7%) | 800 (Cash: 197; Charity: 199; Control: 201; Fitbit only: 203) | OM | Cash: 64.5 (13.6); Charity: 66.5 (13.4); Control: 66.4 (14.1); Fitbit only: 66.9 (15.2) | Cash: 24.0 (4.3); Charity: 24.2 (3.8)  Control: 24.2 (4.2); Fitbit only: 24.2 (4.5) | - | - | **SBP:** Cash: 115.4 (17.1); Charity: 114.5 (15.8); Control: 115.7 (16.6); Fitbit only: 113.5 (15.6)  **DBP:** Cash: 71.9 (10.6); Charity: 71.4 (10.3); Control: 72.0 (11.2); Fitbit only: 70.6 (9.8) | **VO_2_ (ml/kg/min):**  Cash: 35.4 (4.8)  Charity: 35.3 (4.6)  Control: 35.0 (4.8)  Fitbit only: 35.7 (4.5) |
| Fisher, 2018 | Office workers | 54% | | 42% | 131 | OM | - | 25.6 (4.5) | - | - | - | - |
| Foley, 2016 | Office workers | 38 (43%) | | 50 (57%) | 56 | SR | - | 25.7 (range: 17.4-42.8) | - | - | - | - |
| Gao, 2016 | Office workers | 8 | | 6 | 14 | OM | 69.6 (10.8) | 24.0 (2.5) | - | - | - | **WHR:** 0.9 (0.1) |
| Gilson, 2012a | Office workers | 288 (69%) | | 102 (31%) | 330 | NR | - | 27.1 (5.5) | - | - | - | - |
| Gilson, 2012b | Office workers | 7 (64%) | | 4 (36%) | 11 | SR | - | Total: 26.8 (3.9)  Women: 26.8 (3.9)  Men: 24.5 (2.7) | - | - | - | - |
| Gilson, 2016 | Office workers | 11 (20%) | | 46 (80%) | 57 | OM | - | 28 (5) | Men: 98 (14) Women: 88 (13) | - | **SBP:** 128 (13)  **DBP:** 86 (9) | - |
| Gorman, 2013 | Office workers | 75% | | 25% | 27 | OM | 67.8 (12) | - | - | 26.6 (8.9) | - | **HDL (mmol/L)**: 1.6 (0.4)  **TG (mmol/L)**: 0.8 (0.4)  **Glucose (mmol/L)**: 4.8 (0.3) |
| Hallman, 2018 | Office workers | 50 | | 60 | 110 | SR | - | I = 25.1 (4.3)  C = 26.2 (4.7) | - | - | - | - |
| Healy*,* 2013 | Office workers | 23 (56%) | | 19 (44%) | 43 | OM | - | 26.8 (5.4) | - | - | - | - |
| Healy, 2016 | Office workers | 158 (68%) | | 73 (32%) | 229 | OM | - | 28.6 (6.1) | - | - | - | - |
| Kirk, 2016 | Office workers | 27 (93%) | | 2 (7%) | 29 | NR | - | 25.8 (4.1) | - | - | - | - |
| Kloster, 2017 | Office workers | 201 (63%) | | 102 (37%) | 317 | OM | - | 26. (4.9) | Men 97.7 (88-106**  Women: 86.3 (77.8-97.4) ** | - | - | - |
| Koepp, 2013 | Office workers | 25 (69%) | | 11 (31%) | 36 | OM | 86.9 (26.6) | - | 95 (19) | 31.4 (7.9) | **SBP:** 132 (13) **DBP:** 89 (18) | **TC (mmol/L):** 5.0 (0.8)  **HDL (mmol/L):** 1.4 (0.5)  **LDL (mmol/L)**: 2.9 (0.8)  **TG (mmol/L):** 1.5 (1.0)  **Glucose (mmol/L):** 5.2 (1.3)  **HbA1c (%)**: 5.3 (0.9) |
| Kozey-Keadle, 2012 | Office workers | 15 (75%) | | 5 (25%) | 20 | NR | - | 33.7 (5.6) | - | - | - | - |
| Lagersted-Olsen, 2014 | office workers | 12 (46%) | | 14 (54%) | 26 | OM | Men: 79.8 (10.0); Women: 71.5 (15.3) | 25.2 (4.1) | Men: 86.6 (8.2); Women: 80.6 (13.5) | Men: 17.9 (4.8); Women: 30.0 (8.5) | **SBP:**124.1 (10.2)  **DBP:** 82.3 (7.5) | **WHR:**  Men: 0.9 (0.1;  Women: 0.8 (0.1)  **VO_2_ (ml/kg/min):** 43.8 (12) |
| Li, 2017 | Office workers | 20 (77%) | | 6 (23%) | 26 | NR | 70 (16) | 25 (4) | - | - | - | - |
| Mansoubi, 2016 | Office workers | 22 (55%) | | 18 (45%) | 40 | OM | Men: 81.5 (12)  Women: 66.6 (15.1) | Men: 25.9 (3.5)  Women: 24.3 (4.9) | Men: 85.5 (8.7)  Women: 75.9 (10.8) | Men: 25.9 (3.5)  Women: 29 (10.2) | - | - |
| Mitsui, 2010 | Office workers | 0 (0%) | | 50 (100%) | 50 | OM | 69.4 (9.9) | 24 (3.4) | - | - | - | - |
| Neuhaus, 2014 | Office workers | 37 (84%) | | 7 (16%) | 44 | OM | - | 26 (5.1) | - | - | - | - |
| Parry, 2013a | Office workers | 42% | | 58% | 50 | NR | - | 24.7 (4.1) | - | - | - | - |
| Parry*,* 2013b | Office (clerical workers, call centre, data processing) | 50 (80.6%) | | 12 (19.4%) | 62 | OM | - | 28 (6.4) | - | - | - | - |
| Peavler, 2012 | Desk-based workers | I: 86.9%; C: 94.1% | | I: 13.1%; C: 5.9% | 40 (I: 23; C: 17) | OM | I: 88.1 (15.8) C: 91.4 (13.7) | I: 31.7 (4.9)  C: 33.2 (4.5) | - | - | - | - |
| Pedersen, 2016 | Office workers | 100 (74%) | | 63 (26%) | 153 | NR | - | 26 (5) | - | - | - | - |
| Pontt, 2015* | Office workers | 0 (0%) | | 29 (100%) | 29 | NR | - | 29.9 (NR) | - | - | - | - |
| Ryan, 2011 | Office workers | 17 (76%) | | 5 (24%) | 22 | NR | Administrators: 77 (17) | Administrators: 27.0 (4.9) | - | - | - | - |
| Ryde, 2013 | Office workers | 68 (65%) | | 37 (35%) | 105 | OM | 0 | 26.1 (3.9) | 83.8 (11.4) | - | - | - |
| Sawyer, 2017 | Office workers | 61 (54%) | | 52 (46%) | 115 | OM | - | 25.8 (4.5) | - | - | - | - |
| Schwartz, 2016 | Office workers | 89% | | 11% | 97 | OM | - | 29.2 (7.9) | - | 29.7 (16.3) | - | - |
| Swartz, 2014 | Office workers | 68% | | 32% | 60 | OM | 83.4 (25) | 28.5 (7.4) | - | - | - | - |
| Taylor, 2016 | Office workers | 82% | | 18% | 175 | OM | - | **Usual breaks:** 29.8 (95% CI: 27.7, 31.8) **Computer prompts:**  28.3 (95% CI: 26.8, 29.9) **Booster breaks:**  32.2 (95% CI: 30.2, 34.3) | **Usual breaks:** 100.1 (95% CI: 94.5, 105.7) **Computer prompts:**  93.7 (95% CI: 90.2, 97.3) **Booster breaks:**  101.4 (95% CI: 95.8, 106.9) | - | **SBP:** 116 ± 15.4  **DBP:** Usual breaks: 70 (95% CI: 67, 73)  Computer prompts: 70 (95% CI: 67, 73)  Booster breaks: 71 (95% CI: 69, 73) | **TC (mmol/L):** 4.92 ± 0.91  **TG (mmol/L):** 1.37 ± 0.10  **Glucose (mmol/L):** 5.33 ± 1.54 |
| Tigbe, 2011* | Postal workers (office) | 10 | | 46 | 56 | NR | - | 27.4 (4) | - | - | - | - |
| Toomingas, 2012 | Call center operators | 97 (69%) | | 43 (31%) | 140 | SR | 71.4 (13.5) | 24.4 (3.8) | - | - | - | - |
| Urda, 2016 | Office workers | 44 (100%) | | 0 (0%) | 44 | OM | - | 30.5 (8.2) | - | - | - | - |
| Waters, 2016 | Office workers | 24 (60%) | | 13 (40%) | 37 | OM | - | 25 (5) | Men: 92.1 (11.2) Women: 78.3 (10.3) | - | - | - |
| Zhu, 2018 | Office workers | 9 (25%) | | 27 (75%) | 36 | OM | Intervention: 75.0 (20.4)  Comparison: 73.2 (12.0) | Intervention: 26.0 (5.4)  Comparison: 25.4 (3.5) | - | - | **SBP:**  Intervention: 119.1 (16.4)  Comparison: 118.8 (12.2)  **DBP:**  Intervention: 75.6 (10.3)  Comparison: 77.2 (10.8) | **TC (mmol/L):**  Intervention: 4.52 (0.75)  Comparison: 5.12 (0.73)  **HDL-C (mmol/L):**  Intervention: 1.59 (0.51)  Comparison: 1.72 (0.48)  **LDL-C (mmol/L):**  Intervention: 2.81 (0.74)  Comparison: 3.16 (0.66)  **TG (mmol/L):**  Intervention: 1.01 (0.57)  Comparison: 1.28 (0.54)  **Glucose (mmol/L):**  Intervention: 5.54 (0.85)  Comparison: 5.02 (0.29) |
| **Office workers with sit-stand desks** | | | | | | | | | | | | |
| Carr, 2016 | Office workers with access to sit-stand desks | | 74% | 26% | 31 | OM | 89.4 (22.2) | 31.0 (6.4) | 100.6 (18.1) | 39.2 (7.9) | **SBP:** 120 (12)  **DBP:** 73 (10) | **HR (bpm):** 76 (7)  **VO_2_ (ml/kg/min):** 30.8 (8.2) |
| Danquah, 2017 | Office workers with access to sit-stand desk | | 210 (66%) | 107 (44%) | 317 (I: 173; C: 144) | OM | - | 26 (4.9) | I : 92.3 (1.1)  C: 90.8 (1.1)  Men: 97.9 Women: 88.4 | I: 29.2 (0.73) C: 31.4 (0.85)  Men: 22.1  Women: 34.2 | - | I: fat mass = 23.4 (0.8); fat-free mass = 54.3 (0.9)  C: fat mass = 24.5 (0.8); fat-free mass: 52.3 (0.9) |
| Donath, 2015 | Office workers with access to sit-stand desk | | 23 (74%) | 8 (26%) | 31 | OM | I: 65.8 (12.9); C: 70.4 (15.7) | I: 23.7 (3.7); C: 24.7 (5) | - | I: 28.2 (7.3); C:28.3 (8.3) | - | - |
| Gao, 2016 | Office workers with sit-stand desks | | 2 | 8 | 10 | OM | 67.4 (10.7) | 23.2 (3.4) | - | - | - | **WHR:** 0.8 (0.1) |
| Wick, 2016 | Office workers with access to sit-stand desks | | 30 (79%) | 8 (21%) | 38 | OM | - | 23.9 (4.2) | - | - | - | - |
| **Postal delivery** | | | | | | | | | | | | |
| Chastin, 2009* | Postal workers (delivery) | | 5 (13%) | 34 (87%) | 39 | SR | 79 (13) | 27 (4) | - | - | - | - |
| Tigbe, 2011* | Postal workers (delivery) | | 5 (9%) | 51 (91%) | 56 | NR | - | 26.3 (3) | - | - | - | - |
| **Protective services** | | | | | | | | | | | | |
| Aandstad, 2016 | Home guard soldiers | | 0 | 411 (100%) | 411 | OM | 84.8 (13.1) | 26.1 (3.7) | - | - | - | - |
| Chappel, 2016 | Firefighters | | 8 (24%) | 26 (76%) | 34 | SR | 82.2 (13.7) | 26.3 (3.4) | - | - | - | - |
| Cuddy, 2015 | Firefighters | | 3 (20%) | 12 (80%) | 15 | OM | 78.3 (8.6) | - | - | - | - | - |
| Talbot, 2011 | National Guard Personnel | | I: 15 (31%); C: 9 (19%) | I: 33 (68%); C: 37 (80%) | 94 | OM | - | I: 28.6 (5.1) C: 29.8 (3.8) | - | - | **SBP:**  I: 120.2 (16.5); C: 125.7 (15.3)  **DBP:**  I: 74.7 (11.9); C: 77.5 (10.2) | **TC (mmol/L):**  I: 4.8 (1.0); C: 4.7 (0.8)  **HDL (mmol/L):**  I: 1.4 (0.4); C: 1.2 (0.3) **LDL (mmol/L):**  I: 2.8 (1.0); C: 2.9 (0.8) |
| Vincent, 2016 | Firefighters | | 9 (23%) | 31 (77%) | 40 | SR | - | 26.8 (4.7) | - | - | - | - |
| **School teachers** | | | | | | | | | | | | |
| Cheung, 2008 | School teachers | 41 (79%) | | 11 (21%) | 52 (I: 38; C: 14) | OM | Men: 67.5 (8.9)  Women: 52.2 (7.5) | Men: 22.9 (3.3)  Women: 21.3 (3.1) | - | Men: 19.9 (5.1)  Women: 26.0 (0.6) | - | **WHR:**  Men: 0.9 (0.0)  Women: 0.8 (0.1) |
| Cheung, 2012 | School teachers | NR | | NR | 71 (PE teacher: 18; non-PE: 53) | OM | - | **Total:** 22.3 (3.8)  **PE teachers:** 22.1 (3.1) **Non-PE teachers:** 22.4 (3.9) | - | **Total:** 25.8 (7.2)  **PE teachers:** 25.8 (8.9)  **Non-PE teachers:** 25.8 (6.6) | - | **WHR:**  **Total:** 0.79 (0.08)  **PE teachers:** 0.78 (0.07)  **Non-PE teachers:** 0.79 (0.08) |
| **Other occupations** | | | | | | | | | | | | |
| Clark, 2011 | Customer service | 9 (60%) | | NR | 15) | OM | - | 24.8 (23.1-28.3)** | - | - | - | - |
| Hallman, 2017* | Workers from cleaning, transportation and manufacturing sectors | cleaning = 24% manufacturing = 61% transportation = 97% | | cleaning = 86% manufacturing = 39% transportation = 3% | 625 | OM | - | Total: 27.6 (4.9) | - | - | - | - |
| Ryan, 2011 | Technicians | 3 (43%) | | 4 (57%) | 7 | NR | 71 (12) | 23.5 (2.4) | - | - | - | - |
| Weiler, 2015 | Professional football players | 0 (0%) | | 25 (100%) | 25 | NR | 81.9 (7.8) | 23.9 (1.3) | - | - | - | - |

Data presented as sample size (%) or mean (standard deviation) unless otherwise stated

* indicates studies with two or more occupational data sets

**represents median and (interquartile range)

Abbreviations: %BF – body fat percentage, BM – body mass, BMI – body mass index, BP – blood pressure, C - control group, DBP – diastolic blood pressure, HbA1c – haemoglobin A1c, HDL-C – high density lipoprotein cholesterol, HOMA-IR – homeostatic model assessment insulin resistance, I - intervention group, LDL-C – low density lipoprotein cholesterol, NR – not reported, OM – objectively measured, PE - physical education, PT - physiotherapist, RHR – resting heart rate, SBP – systolic blood pressure, SD – standard deviation, SE - standard error, SR – self-reported, TC – total cholesterol, TC-HDL – total cholesterol to HDL ratio, TG – triglycerides, WC – waist circumference, WHR – waist to hip ratio, VO_2max_ – maximum rate of oxygen consumption,
